# The genome of cultivated peanut provides insight into legume karyotypes, polyploid evolution and crop domestication

**DOI:** 10.1038/s41588-019-0402-2

**Published:** 2019-05-01

**Authors:** Weijian Zhuang, Hua Chen, Meng Yang, Jianping Wang, Manish K. Pandey, Chong Zhang, Wen-Chi Chang, Liangsheng Zhang, Xingtan Zhang, Ronghua Tang, Vanika Garg, Xingjun Wang, Haibao Tang, Chi-Nga Chow, Jinpeng Wang, Ye Deng, Depeng Wang, Aamir W. Khan, Qiang Yang, Tiecheng Cai, Prasad Bajaj, Kangcheng Wu, Baozhu Guo, Xinyou Zhang, Jingjing Li, Fan Liang, Jiang Hu, Boshou Liao, Shengyi Liu, Annapurna Chitikineni, Hansong Yan, Yixiong Zheng, Shihua Shan, Qinzheng Liu, Dongyang Xie, Zhenyi Wang, Shahid Ali Khan, Niaz Ali, Chuanzhi Zhao, Xinguo Li, Ziliang Luo, Shubiao Zhang, Ruirong Zhuang, Ze Peng, Shuaiyin Wang, Gandeka Mamadou, Yuhui Zhuang, Zifan Zhao, Weichang Yu, Faqian Xiong, Weipeng Quan, Mei Yuan, Yu Li, Huasong Zou, Han Xia, Li Zha, Junpeng Fan, Jigao Yu, Wenping Xie, Jiaqing Yuan, Kun Chen, Shanshan Zhao, Wenting Chu, Yuting Chen, Pengchuan Sun, Fanbo Meng, Tao Zhuo, Yuhao Zhao, Chunjuan Li, Guohao He, Yongli Zhao, Congcong Wang, Polavarapu Bilhan Kavikishor, Rong-Long Pan, Andrew H. Paterson, Xiyin Wang, Ray Ming, Rajeev K. Varshney

**Affiliations:** 1 Fujian Provincial Key Laboratory of Plant Molecular and Cell Biology, Oil Crops Research Institute, State Key Laboratory of Ecological Pest Control for Fujian and Taiwan Crops, Fujian Agriculture and Forestry University, Fuzhou, China; 2 Nextomics Biosciences Institute, Wuhan, China; 3 Haixia Institute of Science and Technology, Fujian Agriculture and Forestry University, Fuzhou, China; 4 Agronomy Department, University of Florida, Gainesville, FL USA; 5 Center of Excellence in Genomics & Systems Biology, International Crops Research Institute for the Semi-Arid Tropics (ICRISAT), Hyderabad, India; 6 College of Biosciences and Biotechnology, National Cheng Kung University, Tainan, Taiwan; 7 Graduate Program in Translational Agricultural Sciences, National Cheng Kung University and Academia Sinica, Taipei, Taiwan; 8 Guangxi Academy of Agricultural Sciences, Nanning, China; 9 Biotechnology Research Center, Shandong Peanut Research Institute, Shandong Academy of Agricultural Sciences, Shandong, China; 10 North China University of Science and Technology, Tangshan, China; 11 The University of Western Australia, Perth, Western Australia Australia; 12 USDA-ARS, Crop Protection and Management Research Unit, Tifton, GA USA; 13 Henan Academy of Agricultural Sciences, Zhengzhou, China; 14 Oil Crops Research Institute of the Chinese Academy of Agricultural Sciences, Wuhan, China; 15 Zhongkai University of Agriculture and Engineering, Guangzhou, China; 16 College of Crop Sciences, Fujian Agriculture and Forestry University, Fuzhou, China; 17 School of Life Science, TsingHua University, Beijing, China; 18 Guangdong Provincial Key Laboratory for Plant Epigenetics, College of Life Sciences and Oceanography, Shenzhen University, Shenzhen, China; 19 Tuskegee University, Tuskegee, AL USA; 20 Osmania University, Hyderabad, India; 21 College of Life Science, National Tsing Hua University, Hsin Chu, Taiwan; 22 Plant Genome Mapping Laboratory, University of Georgia, Athens, GA USA; 23 Department of Plant Biology, University of Illinois of Urbana-Champaign, Urbana, IL USA

**Keywords:** Genomics, DNA sequencing

## Abstract

High oil and protein content make tetraploid peanut a leading oil and food legume. Here we report a high-quality peanut genome sequence, comprising 2.54 Gb with 20 pseudomolecules and 83,709 protein-coding gene models. We characterize gene functional groups implicated in seed size evolution, seed oil content, disease resistance and symbiotic nitrogen fixation. The peanut B subgenome has more genes and general expression dominance, temporally associated with long-terminal-repeat expansion in the A subgenome that also raises questions about the A-genome progenitor. The polyploid genome provided insights into the evolution of *Arachis hypogaea* and other legume chromosomes. Resequencing of 52 accessions suggests that independent domestications formed peanut ecotypes. Whereas 0.42–0.47 million years ago (Ma) polyploidy constrained genetic variation, the peanut genome sequence aids mapping and candidate-gene discovery for traits such as seed size and color, foliar disease resistance and others, also providing a cornerstone for functional genomics and peanut improvement.

## Abstract (web summary)

High-quality genome sequence of cultivated peanut comprising 2.54 Gb with 20 pseudomolecules and 83,709 protein-coding gene models provides insights into genome evolution and the genetic mechanisms underlying seed size and leaf resistance in peanut.

## Main

Cultivated peanut or groundnut (*A. hypogaea* L.) is among the most important oil and food legumes, grown on 25 million ha between latitudes 40° N and 40° S with annual production of ~46 million tons (http://www.fao.org/faostat/en/#home). It presumably was domesticated in South America ~6,000 years ago and then was widely distributed in post-Columbian times^1^. Combining richness in seed oil (~46–58%) and protein (~22–32%), peanut is important in fighting malnutrition and ensuring food security. A ‘longevity fruit’^2^, peanut also offers health benefits such as richness in heart-healthy oleic and linoleic acid; resveratrol, fiber and folic acid; and easily digested protein. In Asia and Africa, more peanut is produced than any other grain legume including soybean^1^ (http://www.fao.org/faostat/en/#home). In China, peanut accounts for >46% of total output of all oil crops, ranking fourth after rice, wheat and corn in market value. A nitrogen-fixing *Fabaceae* plant with geocarpy, peanut can grow on arid and marginal land^3^. With yield averaging 3,649 kg ha^−1^ in China in recent years and especially with the advent of high oleic acid cultivars, peanut is increasingly important as an oil and protein source.

The *Arachis* genus contains 81 species, mostly diploids (2*n* = 2*x* = 20). Genetic, cytogenetic, phylogeographic and molecular evidence suggested that hybridization between diploids *A. duranensis* (AA genome) and *A. ipaensis* (BB) may have formed the allotetraploid *A. hypogaea*^4–9^ (AABB, 2*n* = 4*x* = 40) (refs. ^1,4,5,10–13^). Genomic in situ hybridization suggests that *A. monticola* may be the immediate wild ancestor of *A. hypogaea*^6^. Although agronomic traits differ dramatically between cultivated peanut and its wild progenitors, cytogenetic and genetic studies^8,9,14^ suggest few changes in the A and B subgenomes since polyploidization.

The complexity resulting from closely related subgenomes, repetitive sequence abundance and large genome size (2.7 Gb) complicates peanut genome assembly^1^. We present a reference genome sequence of cultivated peanut to facilitate understanding of genome architecture and accelerate crop improvement. Resequencing of 30 allotetraploid accessions of various ecotypes, 18 wild species and four synthetic tetraploids provides insights into peanut genome architecture, trait biology, evolution and domestication.

## Results

### Sequencing, assembly and annotation

A reference genome assembly was developed for *A. hypogaea* var. Shitouqi (zh.h0235, a well-known Chinese cultivar and breeding parent belonging to subspecies *fastigiata*, botanical type *vulgaris* and agronomic type Spanish with heterozygosity only 1/6,537 nucleotides on average) (Supplementary Note 1.1). First, assembly of 100× single-molecule real-time sequences^15^ yielded contigs totaling 2.54 Gb, 94% of estimated peanut genome size^1^, with N50 (the shortest contig length at 50% of the total assmbled genome accumulated from the longest one) of 1.51 Mb (Table 1; Supplementary Note 1.3). Approximately 90% of the assembly was contributed by just 1,804 contigs (Table 1; Supplementary Note 1.1). Assembly thresholds of overlapping reads >96% and overlapping length >2,000 base pairs (bp) were used. Second, chromosome conformation capture (Hi-C) sequencing produced 31,734,151 valid paired-end reads that covered 99.6% of assembly length. This allowed assembly of PacBio contigs into 20 chromosome-scale scaffolds with N50 of 129.8 Mb, accounting for 95.5% of assembled sequences (Table 1; Supplementary Fig. 1; Supplementary Dataset 1) after dissociating 297 mistakenly assembled contigs by three-dimensional proximity information^16–18^ and/or genetic mapping.

**Table 1:** Peanut genome assembly statistics

	*A. duranensis* (2*n* = 2*x* = 20)		*A. ipaensis* (2*n* = 2*x* = 20)		*A. hypogaea* (2*n* = 4*x* = 40)		
	Illumina	Illumina + Linkage Map	Illumina	Illumina + Linkage Map	PacBio^a^	PacBio + Hi-C	PacBio + Hi-C + Linkage Map
Total assembly size of contigs (bp)	1,211,482,656		1,512,089,950		2,538,408,906		
Number of contigs	765,406		869,435		7,232		
N50 contig length (bp)	22,293		23,492		1,509,423		
N90 contig length (bp)	NA		NA		342,540		
L50 contig count	12,992		15,898		505		
L90 contig count	NA		NA		1,804		
Longest contig (bp)	221,145		250,973		8,550,813		
Total assembly size of scaffolds (bp)	1,074,450,206	1,041,781,911	1,388,638,929	1,342,408,530		2,424,161,010	2,506,735,760
Number of scaffolds	635,392	10	759,499	10		20	20
N50 scaffold length (bp)	947,955	110,037,037	5,343,284	136,175,642		129,846,058	135,108,068
N90 scaffold length (bp)	NA	94,617,824	NA	126,351,151		104,681,234	109,264,827
L50 scaffold count	334	5	86	5		10	9
L90 scaffold count	NA	8	NA	9		17	17
Missing bases (%)^b^	11.3	3.0	8.2	3.3		4.5	1.3

NA, not available; L50, smallest number of contigs whose length sum makes up half of the assembled genome; L90, smallest number of contigs whose length sum makes up 90% of the assembled genome. ^a^With HiSeq clean data 1,350 Gb for quivering.

^b^Missing bases (%) = Gap length / total assembly size × 100.

To support chromosome assembly, we integrated two new genetic maps with two existing genetic maps^19,20^ through ALLMAPS^21^, yielding 14,619 loci in 20 linkage groups covering 3,264.4 cM (Supplementary Fig. 2; Supplementary Table 1; Supplementary Datasets 2 and 3). Finally, 20 pseudomolecules were created by anchoring 6,289 contigs to the genetic and Hi-C scaffold maps through ALLMAPS together with minor manual adjustment of five Hi-C assembled error scaffolds based on genetic maps. The 2.51 Gb total size of pseudomolecules is 98.75% of total assembly length (chromosomes are designated Chr01–Chr20, corresponding to A01–A10 and B01–B10 of the diploid A and B chromosomes^1^, respectively). The remaining contigs (32.3 Mb) were designated chromosome_00 (Table 1; Supplementary Dataset 3). High co-linearity between the assemblies and published BACs^22^ and 1,576 *A. hypogaea* BAC paired-end sequences (GenBank accession numbers FI498696.1 to FI503143.1; https://www.ncbi.nlm.nih.gov/nucgss?term=Bactierial+artificial+chromosome+of+Arachis+hypogaea) (Supplementary Fig. 3; Supplementary Dataset 4a), low consensus error rate and high contig read depth (Supplementary Dataset 4b,c; Supplementary Note 1.8) all indicated high assembly quality.

A total of 83,709 protein-coding genes (Supplementary Table 2a) were inferred from the assembly using ab initio prediction with supporting evidence of RNA-sequencing (RNA-seq) data from 39 tissues/conditions and PacBio isoform sequencing (Iso-Seq) (Supplementary Table 2c; Supplementary Dataset 5a; Supplementary Fig. 4a,c). On average, the genes encode transcripts of 1,589.5 bp with 6.8 exons and proteins of 403 amino acids, comparable to other legumes but longer than the diploid A and B genomes^1^ (Supplementary Table 2a,b). Among coding gene models, 62,781 were annotated with annotation edit distance^23^ values ≤0.38 (Supplementary Fig. 4d). Approximately 76.6% of predicted genes were assigned functional annotations (Supplementary Table 2d). We identified 39,127 non-coding RNA (ncRNA) including 4,723 transfer RNAs (tRNAs), 3,107 ribosomal RNAs (rRNAs), 480 microRNAs (miRNAs) and 30,817 small nuclear RNAs (Supplementary Table 3a; Supplementary Dataset 5b). A total of 1.97 Gb (77.65%) of genome sequences was repetitive, including 1.67 Gb (64.74%) of retrotransposons and 114 Mb (4.49%) of DNA transposons (Supplementary Tables 3b and 5). Gypsy and nonautonomous long-terminal repeat (LTR) retrotransposons comprised 40.59% and 27.14% of the genome, respectively. Identification of 93.1% of the 1,440 genes in the Plantae BUSCO dataset^24^ (Supplementary Table 4) indicated high quality of genome assembly and annotation.

**Fig. 1:**
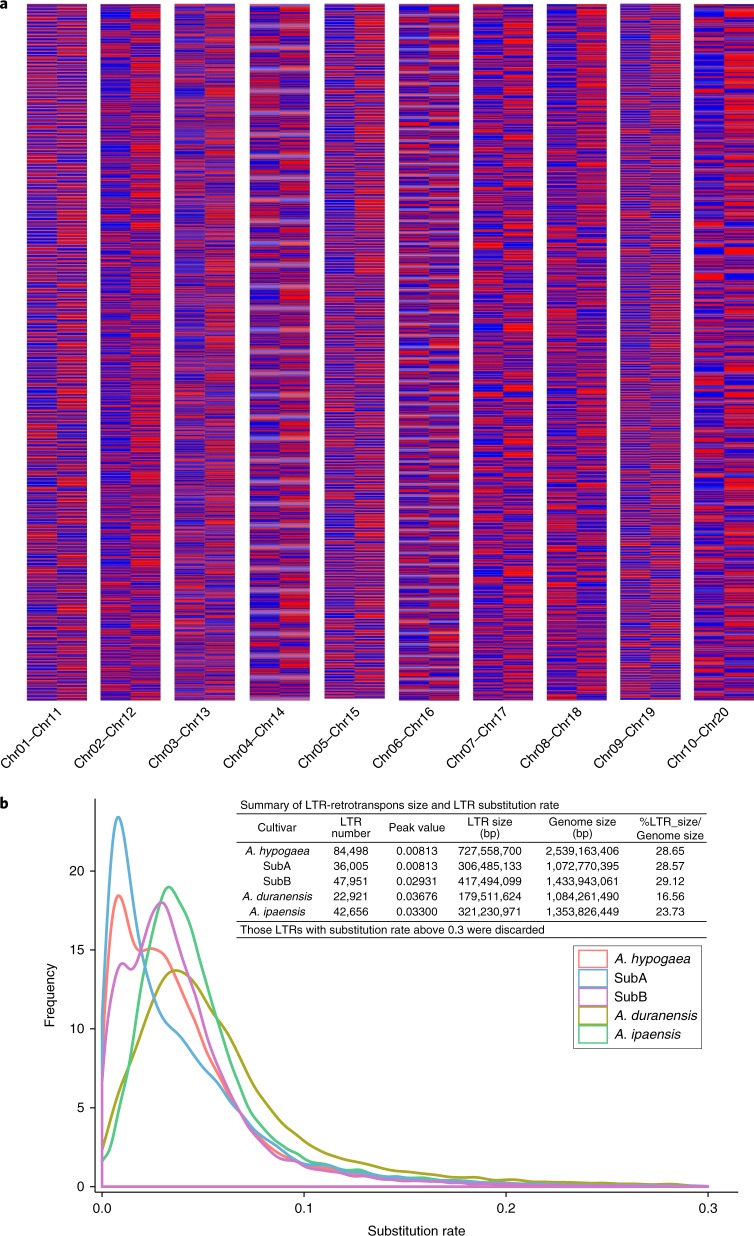
Expression differentiation of paired homeologous genes between peanut subgenomes and repeat expansion among peanut and diploid ancestor genomes. **a**, Widespread expression differentiation of homeologous gene pairs between two subgenomes is shown. Homeologous chromosomes are indicated at the bottom of the figure. **b**, Density distribution of substitution rates using the paired-end sequences of LTR retrotransposons in the *A. hypogaea*, *A. hypogaea*-SubA, *A. hypogaea*-SubB, *A. duranenesis* and *A. ipaensis* genomes. The LTR in *A. hypogaea* and *A. hypogaea*-SubA exhibited rapid expansion ~246,700 years ago, but those of *A. hypogaea*-SubB, *A. duranensis* and *A. ipaensis* did so about 0.8922, 1.1206 and 1.0049 Ma, respectively, based on the formula *T* = *S*/2 µ (where *T* is the evolution time, *S* is the substitution rate here and µ is the 1.64 × 10^−8^ substitution rate per year; Supplementary Note 3.3.5). The number of LTR retrotransposons and the peak substitution rate for each part are embedded in the figure.

Peanut genome complexity combines allotetraploidy with other gene duplication mechanisms. A total of 30,596 nonredundant peanut genes including 24,208 (79.12%) with and 6,338 (20.88%) without homeologs (Supplementary Table 6) were identified. Among the 6,388 genes without homeologs, 2,421 appear to have formed since tetraploidy, and some might be false annotations because 47% (1,140) of tetraploid-specific genes had an annotation edit distance larger than 0.4. We also detected 27,913 duplicated genes with 10,590 and 17,323 in subgenomes A and B (Supplementary Dataset 5c), including 2,402 tandem (consecutive) and 25,511 dispersed duplications (on different chromosomes or apart in the same chromosome) (Supplementary Table 6). In 29 RNA samples, the 24,208 homeologous pairs showed widespread differential expression (Fig. 1a), with dominant expression more frequent among B than A subgenome homeologs (Fig. 1a).

### Characterization of subgenome structure

**Fig. 2:**
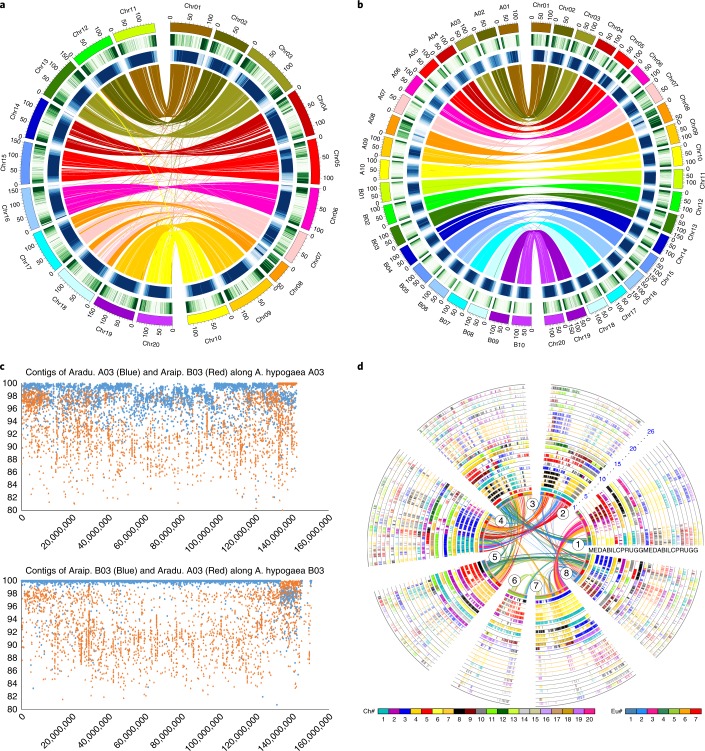
Characterization of the peanut genome and chromosomes. **a**, Circos diagram depicting relationships of A and B subgenome chromosomal pseudomolecules. The scale for the chromosomes (outer bars) is megabases; colors represent the density of nonautonomous LTR retrotransposons and Ty3-gypsy elements (blue) and genes (green). Homeologous blocks of ≥30 gene pairs between Chr01–Chr10 and Chr11–Chr20 (A01–A10 and B01–B10, respectively) are connected with lines. **b**, Syntenic comparisons between peanut subgenomes and diploid A and B genomes. The outer three circles are chromosomes, density of genes and of Ty3-gypsy and nonautonomous LTR retrotransposons (as shown in **a**). Colored lines connect blocks with ≥30 orthologous gene pairs between the A and B subgenomes and *A. duranensis* and *A. ipaensis* genomes, respectively, based on BLASTN. **c**, Alignment of diploid peanut A03 and B03 contigs to corresponding tetraploid chromosomes, with parameters: -a 8 -p blastn -m 9 -e 1e-10. The best hits with alignment length ≥2,000 bp were plotted. Translocation between chromosomes A03 and B03 is evident in cultivated peanut. **d**, Eleven-genome alignment using co-linear genes, each mapped onto the barrel medic chromosomes. A, *A. hypogaea* A; B, *A. hypogaea* B; C, *C. cajan*; D, *A. duranensis*; E, *C. arietinum*; G, *G. max*; I, *A. ipaensis*; L, *L. japonicus*; M, *M. truncatula*; P, *P. vulgaris*; R, *V. radiata*; U, *V. angularis*.

The B subgenome is more similar to *A. ipaensis* than the A subgenome is to *A. duranensis*^1^ (also shown in Supplementary Dataset 7) with 2,543 (1,408.4 Mb, 55.49% of contig length, >93.28% identity) anchored B genome contigs, versus 2,477 (1,085.7 Mb, 42.77%, >92.82% identity) A genome contigs (Supplementary Dataset 6; Supplementary Note 3.3.1). The diploid A genome and tetraploid A subgenome shared 34,266 co-linear genes, whereas the diploid B genome and tetraploid B subgenome shared 38,417, also indicating better preservation of gene co-linearity (Fig. 2a,b; Supplementary Table 7; Supplementary Fig. 5a). There was also better co-linearity of the B subgenome with other legumes (Supplementary Table 7), reflected by 2,067 gene pairs in 301 co-linear blocks with more than 4 homeologous gene pairs in the A subgenome and 2,283 gene pairs in 300 blocks in the B subgenome (Supplementary Table 7). A total of 629 genes (1.8% of 35,576) might have been affected by gene conversion, with 369 (58.7%) A subgenome genes converted by their B subgenome counterparts and 230 (41.3%) vice versa (Supplementary Fig. 6; Supplementary Note 3.2). Well-preserved sequence homology after tetraploidization may facilitate inter-subgenome recombination and rearrangement^25^.

Unbalanced structural rearrangements occurred in the peanut A (sub)genome before and after divergence. Reciprocal comparisons of corresponding diploid and tetraploid chromosomes (Supplementary Note 3.2) identified at least six exchanges or substitutions with clearly defined boundaries between A and B subgenomes, including a 10-Mb translocation between chromosomes 3 and 13 (Supplementary Dataset 7; Fig. 2c). Inversions affecting ≥3 Mb in the A (sub)genome (Supplementary Figs. 7 and 8) included 11 inter-subgenome incongruent chromosomal regions, 11 inter-A (sub)genome regions, and 4 inter-B (sub)genome regions. These comprise 23 independent events, 21 (91.3%) occurring in the A lineage (*Χ*^2^ test *P* = 7.4 × 10^−5^). Interestingly, B-genome-specific crossover occurred in the B (but not A) lineage to make chromosomes 7 and 8 (Fig. 2a; Supplementary Figs. 5b and 9e), and the changes were retained in the A genome^1^ to A subgenome as clearly shown in chromosome 8, which demonstrated irregular gene density distribution patterns (Supplementary Fig. 7b).

Most transposable elements expanded after tetraploidization, especially the Gypsy and unclassified LTRs (Supplementary Table 3b). B subgenome LTRs are derived from the progenitor B genome, but most A subgenome LTRs formed after polyploidization (Fig. 1b; Supplementary Note 3.3.5). Base substitution rates between paired-end sequences showed that the A subgenome and *A. hypogaea* experienced rapid LTR expansion after tetraploidization (~0.25 Ma), but LTRs of the B subgenome and the two diploids expanded before tetraploidization (0.89, 1.12 and 1.00 Ma), respectively. A subgenome LTR expansion may relate to the prevalence of dysfunctional expression or loss of A subgenome homeologous genes in polyploid peanut^26^ (Fig. 1b), and also raised questions about whether the sequenced *A. duranensis* was representative of the A subgenome progenitor.

**Fig. 3:**
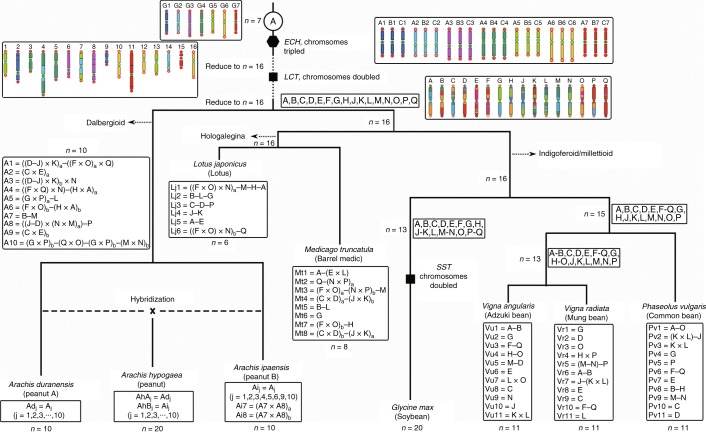
Legume karyotype evolution. The 16 ancestral legume chromosomes (called Lu, denoted by capital letters A–Q), were reconstructed by using corresponding common bean genes and compared with extant legume genomes. By using dot plots between Lu and each legume genome, and between close legume relatives, we reconstruct the origin of peanut chromosomes, including *A. duranensis* and *A. ipaensis*.

Traces of legume-common tetraploidy (LCT) ~59 Ma (ref. ^27^) and core-eudicot-common hexaploidy (ECH) ~130 Ma remain in the peanut genome. Post-ECH genome structure has been well preserved in *V. vinifera*^28^, and post-LCT structure in *P. vulgaris* (Supplementary Note 3.4.1; Supplementary Fig. 9a,b; Supplementary Dataset 8). The A subgenome conserved 1,289 co-linear gene pairs from LCT and 1,198 from ECH, accounting for 6.9% and 6.5% of gene content, and the B subgenome contained 1,508 and 1,372 from LCT and ECH (6.6% and 6.1% of its gene content), respectively. Peanut often preserves ancestral gene arrangements in comparison with other legumes^26^ (Figs. 2d and 3; Supplementary Figs. 9 and 10; Supplementary Dataset 8).

The peanut karyotype formed largely independently from those of other legumes (Fig. 3; Supplementary Note 3.5). ‘Top-down’ grape–legume comparison (Supplementary Fig. 9a,b; Fig. 10) identified 5 independent chromosome fusions, including 3 nested chromosome fusions and 2 end–end joins, producing 16 basic legume ancestral chromosomes before the LCT (Fig. 3; Supplementary Fig. 9c). A bottom-up approach found 5 common bean chromosomes and 11 chromosomal blocks largely preserved in different legumes (Fig. 3; Supplementary Fig. 9d; Supplementary Dataset 8), identifying 16 post-LCT ancestral chromosomes. This means that after doubling in the LCT, the original chromosome number was restored after genome repatterning, resembling maize^29^. Comparison with the 16 post-LCT chromosomes (called Lu), reconstructed by using common bean genes (Supplementary Dataset 8d), revealed peanut ancestral chromosomes A1, A3, A4, A5, A6 and A7 to be composed of segments originated from Lu chromosomes via six fusions resulting in chromosome number reduction. Chromosomes A2, A8, A9 and A10 were produced by two crossovers of Lu chromosomes (Supplementary Fig. 9; Fig. 3; Supplementary Dataset 8). After splitting from the A genome, crossing over in the peanut B genome produced its specific chromosomes 7 and 8 (Supplementary Fig. 9e).

### Changes of subgenome content

**Fig. 4:**
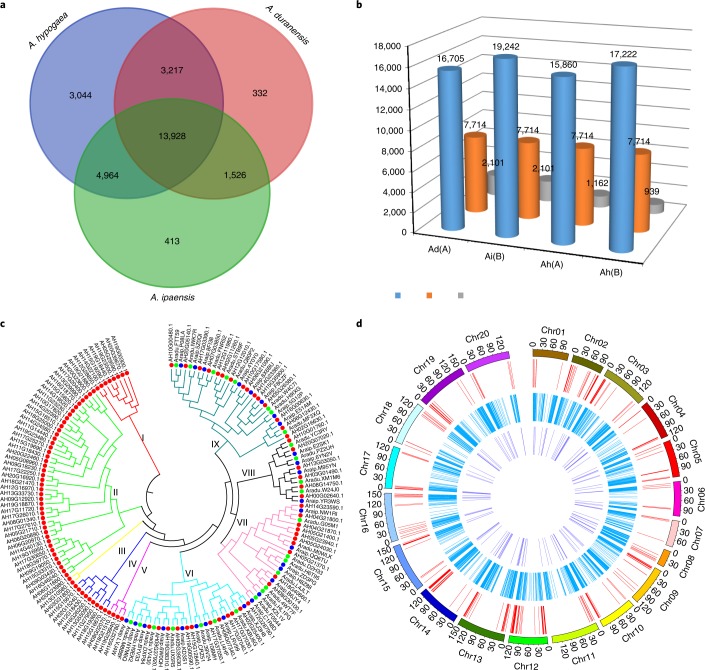
Peanut gene retention after tetraploidization. **a**, Numbers of shared and unique orthologous protein-coding gene clusters in peanut (AHAB), *A. duranensis* (Aradu) and *A. ipaensis* (Araip). **b**, The number of single-copy gene sets is presented (blue), retained as a single copy (orange) or lost (gray) in the peanut A or B subgenomes. **c**, Maximum likelihood tree of ARF gene family, with 114, 28 and 28 members in peanut, *A. duranensis*, and *A. ipaensis*, respectively. Branch values represent the percentage of 1,000 bootstrap replicates supporting the topology. Scale bar represents substitutions per site. **d**, Chromosome distributions of genes involved in fatty acid metabolism, symbiotic nitrogen fixation pathways and biotic stress resistance in cultivated peanut, from outer to inner circles representing chromosomes, R genes, acyl-lipid-related and nodulation-related genes.

The peanut A (37,059 genes) and B (46,650 genes) subgenomes, respectively, showed 0.88% and 12.46% expansion in gene content compared with *A. duranensis* (A) and *A. ipaensis* (B)^1^, supporting dominance of the B subgenome. Compared with related legumes^27^ and *Arabidopsis*, the cultivated peanut genome shared 9,614 orthologous groups/families of genes (53.45% of the total identified) with soybean^30^, common bean^31^, *Medicago*^32^ and *Arabidopsis* (Supplementary Table 8; Supplementary Dataset 9). Of 24,380 orthologous gene families identified in A and B diploid genomes, 22,109 (90.68%) were retained in peanut after tetraploidization (Fig. 4a; Supplementary Fig. 11). Among ortholog gene sets with only one copy found in all three *Arachis* species^1^, 1,162 and 939 genes were lost from the peanut A and B subgenomes, respectively (Fig. 4b). Furthermore, 7,714 genes that are single copy in each diploid remained single copy in both peanut subgenomes (Fig. 4b).

A total of 10,974 gene families had 3–51 copies in tetraploid peanut (Supplementary Dataset 5c). For example, peanut, *A. duranensis* and *A. ipaensis*, respectively, have 114, 28 and 28 members of ‘Auxin response factor’ (ARF), which regulates plant growth and development^33^ (Fig. 4c). The ARF genes group into nine clusters, with I–V including only copies from cultivated peanut, perhaps related to large seed size and organ evolution. Peanut contains three copies of cytochrome P450 78A6 genes (CYP78A6) with two duplicated members compared with just single copies in the diploid B genome, associated with large seed size. Among 3,044 orthologous families of 10,339 genes specific to peanut (Supplementary Table 8), many have functional gene ontology relating to nucleic acid-binding proteins, transcription factors, ATP–NADH-related or ARFs.

The total of 661 genes detected in tetraploid peanut with nucleotide-binding site (NBS) domains characteristic of biotic stress resistance were fewer than the sum of those in *A. duranensis* (385) and *A. ipaensis* (428) (ref. ^1^), and mostly located in terminal regions of chromosomes, particularly Chr02 and Chr04 (Supplementary Dataset 10; Fig. 4d). These genes comprised three groups: coiled coil (CC)-NBS-leucine-rich repeat (LRR) (CNL), Toll/interluekin-1 receptor (TIR)-NBS-LRR (TNL) and (albeit few) resistance to powdery mildew8 (RPW8)-NBS-LRR (RNL) (Supplementary Fig. 12). Many CNL were absent in peanut, suggesting some losses during domestication, although retained TNL numbers were comparable with wild species (Supplementary Fig. 12b,c).

Seed oil content and quality are primary targets for peanut breeding programs^34^. We identified a total of 1,944 acyl-lipid orthologs in peanut, with 1,347 and 1,324 in *A. duranensis* and *A. ipaensis*^1^, respectively (Supplementary Dataset 11a; Fig. 4d; Supplementary Table 9). These genes grouped into 727 gene families. In 50 gene families, the single-copy gene of wild peanut was duplicated at least once in cultivated peanut including six families of 23 genes with more than two duplicates, responsible for fatty acid synthesis, lipid signaling and triacylglycerol (TAG) biosynthesis (Supplementary Table 10). At least 426 genes (Supplementary Dataset 11a) were located within 125 published quantitative trait locus (QTL) regions^35^, comprising candidates for future cloning and oil improvement. We constructed a genome-scale acyl-lipid metabolic network for peanut based on RNA co-expression patterns of four embryo developmental stages (Supplementary Fig. 13; Supplementary Dataset 11b,c), which may facilitate improvement of oil quality and content.

Peanut uses a unique *Rhizobium* infection mechanism for nitrogen fixation^36^, which may be more transferrable to nonlegume species than other mechanisms^37^. We identified a total of 119 orthologous families of nodulation-related genes in 13 legume species (Supplementary Note 4.4) with 81 (68.07%) conserved in all 13 (Supplementary Dataset 11d). Peanut retained 95 families of 169 genes with 4 families missing and 40 experiencing more gene loss (29 families) or gain (11 families) than the sequenced diploids during evolution (Supplementary Dataset 11e). These contained all genes required by other legume species for symbiotic nitrogen fixation. A phylogenetic tree of three ubiquitous symbiosis signaling pathway genes (Supplementary Fig. 14) showed that genes in the *Arachis* species were distinct from, and ancestral to, those in other legumes, perhaps associated with the unique nodulation mechanism (Supplementary Note 4.4).

### The origin and domestication of peanut

**Fig. 5:**
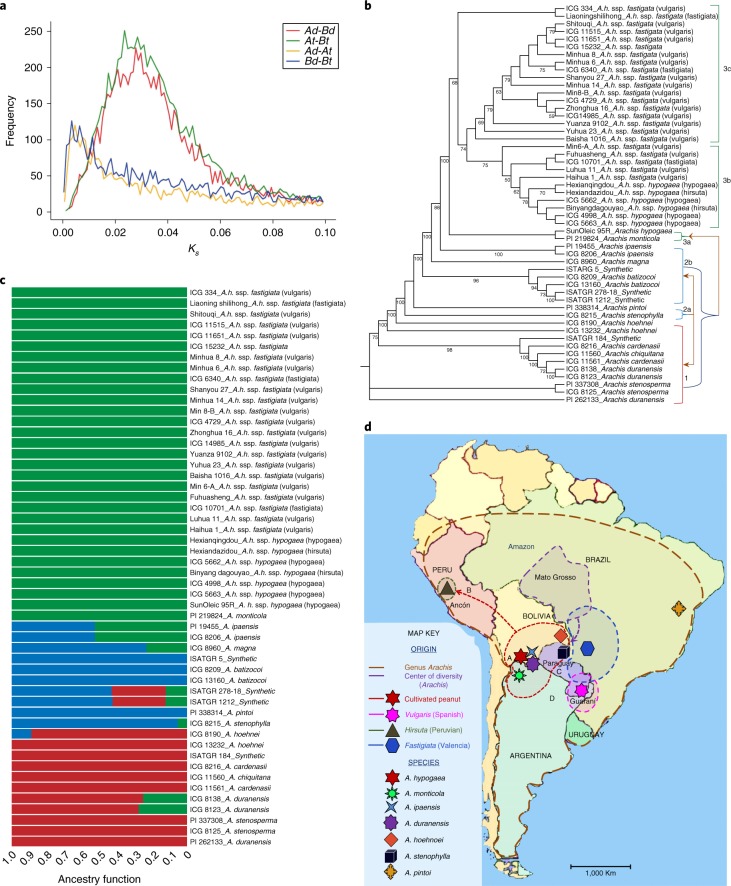
Evolutionary history of peanut. **a**, *K*_s_ distributions of gene pairs in each species. Diploid A (Ad) and B (Bd) genomes diverged from one another about 2.6 Ma, and from their corresponding subgenomes ~0.42–0.47 Ma based on a mutation rate of 8.21 × 10^−9^ *K*_s_ yr^−1^ (ref. ^38^). **b**, Maximum likelihood tree of 52 varieties generated from 17.16 million SNPs. Color brackets indicate different groups. Topologies are supported by percentages of 1,000 bootstrap replicates indicated by branch values. Scale bar represents substitutions per site. **c**, Pattern of admixture analysis of the 52 accessions when *K* = 3. Of the three major groups detected, accessions from A-genome, Pr-genome and one synthetic, ISATGR 184, clustered together as group 1 (red bars). All of the accessions belonging to the B-genome, K-genome, C-genome and E-genome clustered together with three synthetics (ISATGR 5, ISATGR 1212 and ISATGR 278-18) as group 2 (blue bars). The largest group was group 3 (green bars), consisting of all the tetraploids, except the synthetics. **d**, Evolutionary relationships and distribution of *Arachis* species, showing the hypothesized hybridization producing tetraploid *A. monticola* and the subsequent evolution of peanut into two subspecies and four (later six) varieties or ecotypes. Dashed line arrows A–D show the original *A. hypogaea* varieties were moved and domesticated independently to form var. *hypogaea* in Bolivia; Peruvian type (var. *hirsuta*) in Ancon, Peru; Valencia type (*fastigiata*) in Paraguay-central Brazil; and Spanish type (*vulgaris*) in the Guarani area (Paraguay–Argentina–Brazil), respectively^5,40^. Accessions are shown based on collection site. *A.h*., *Arachis hypogaea*; ssp., subspecies; var., variant.

Peanut resulted from a single hybridization between A and B genome species in South America^9^, believed to be *A. duranensis* and *A. ipaensis*^5,6^. Comparison with the sequenced A and B genomes^1^ supports *A. ipaensis* or a close relative as the peanut B subgenome progenitor with average >99.5% identity, but much greater divergence (~97% identity) from *A. duranensis* (Supplementary Datasets 6 and 7; Fig. 2c). By characterizing *K*_s_ values between all co-linear genes based on 8.21 × 10^−9^ *K*_s_ yr^−1^ (Nei–Gojobori approach^38^, Bertioli et al.^1^) (Fig. 5a; Supplementary Note 5.2), we found that the divergence of diploid and tetraploid A or B genomes was dated to ~ 0.42–0.47 Ma and therefore was more ancient than previously thought, thus falsifying the possibility of human involvement in polyploidization^1,39^. The split of A and B subgenomes was estimated at ~ 2.6 Ma, as previously reported^1^. Estimation of the splitting dates of 41 single-copy genes with BEAST2 again confirmed the earlier inference (Supplementary Note 5.2.2).

Among 81 species of nine sections in *Arachis*, peanut evolved from the biggest section, but its exact origin and domestication are still unclear^40^. We resequenced 52 accession of 12 species including *A. duranensis* and *A. ipaensis*, as well as the wild tetraploid *A. monticola*, and 30 diverse peanut samples (Supplementary Dataset 12). Phylogeny, admixture and principal component analysis (PCA) clustered the 52 accessions into three classes using SNP (legends of Fig. 5b,c; Supplementary Figs. 15a and 16a,b). Fst (diversity paramteter) values between groups 1 and 3 (0.76094) or 2 and 3 (0.79683) suggested higher genetic distances and low genome exchange associated with ploidy difference (Supplementary Fig. 16).

Phylogenetic analysis places *A. monticola* as basal after tetraploidization, with subsequent divergence of two subspecies and four (later considered six) varieties (Fig. 5b). The origin of *A. monticola* from *A. ipaensis* and some *A. duranensis* accessions (Fig. 5b,d, earth map showing locations) was supported by similar levels of SNPs and InDels in *A. ipaensis*, *A. monticola* and many peanut varieties, but not in *A. duranensis* (Supplementary Dataset 13; Supplementary Fig. 15). Peanut was predicted to have been domesticated from *A. monticola* in northern Argentina^5^; however, our phylogenetic and sequencing data find *A. monticola* basal to subspecies *hypogaea* and *fastigiata* ecotypes. This indicated that peanut may have started from diverse subspecies *hypogaea* and been domesticated independently in different locations^40^, for example, to the northwest evolving Peruvian ecotypes adaptable to drought (Fig. 5d, arrow B) and to the southeast deriving Valencia and Spanish ecotypes independently (Fig. 5b,d, arrows C and D), which later spread worldwide^40^ (Supplementary Dataset 14). The phylogenetic tree classified most cultivated peanut accessions into two groups corresponding to the two subspecies, but showed clearly that subspecies *hypogaea* intermingled with modern vulgaris-type cultivars (Fig. 5b; Supplementary Dataset 12) bred from inter-subspecies crosses, an approach used to breed widely adaptable, high-yielding cultivars in China.

The sequences of four synthetic tetraploids illustrate opportunities to diversify the peanut gene pool. Phylogenetic analysis grouped synthetics with diploids, indicating high genetic distance from natural tetraploids (Fig. 5b). Synthetic tetraploids ISATGR 278 [*A. duranensis* (ICG 8138) × *A. batizocoi* (ICG 13160)] and ISATGR 5 [*A. magna* (ICG 8960) × *A. batizocoi* (ICG 8209)] seemed to undergo whole-genome duplication. Two other synthetics derived from reciprocal crosses contained 1.23- and 5.93-fold more genome content of *A. duranensis* than the B genome based on read mapping (Supplementary Table 11; Fig. 5b; Supplementary Fig. 17a; Supplementary Note 5.3.2). The A genome enrichment presumably resulted from non-random retention of parent chromosomes in offspring because of incompatibility, which further supports the emerging hypothesis that a species other than *A. duranensis*, which is more compatible with the B genome, is the A genome donor. A total of 17.16 million non-redundant SNPs and 4.52 million non-redundant InDels were identified from the 52 Arachis accessions (Supplementary Dataset 13c,d). Synthetics contained higher numbers of SNPs and InDels than natural tetraploids (Supplementary Dataset 13a,b; Supplementary Fig. 15), offering rich diversity in functional genes and neutral DNA markers.

A finding warranting further investigation is that *A. stenophylla* (EE) and *A. pintoi* (CC) showed diverse SNP patterns, mapped on both subgenomes with low read numbers and grouped between A and B genome accessions (Fig. 5b,c; Supplementary Fig. 17b; Supplementary Table 11; Supplementary Dataset 12). We hypothesize that diploids with E or C genomes might have separately evolved into diploid A and B genomes, which, in turn, hybridized to form peanut (Fig. 5b).

### Impact on peanut improvement

**Fig. 6:**
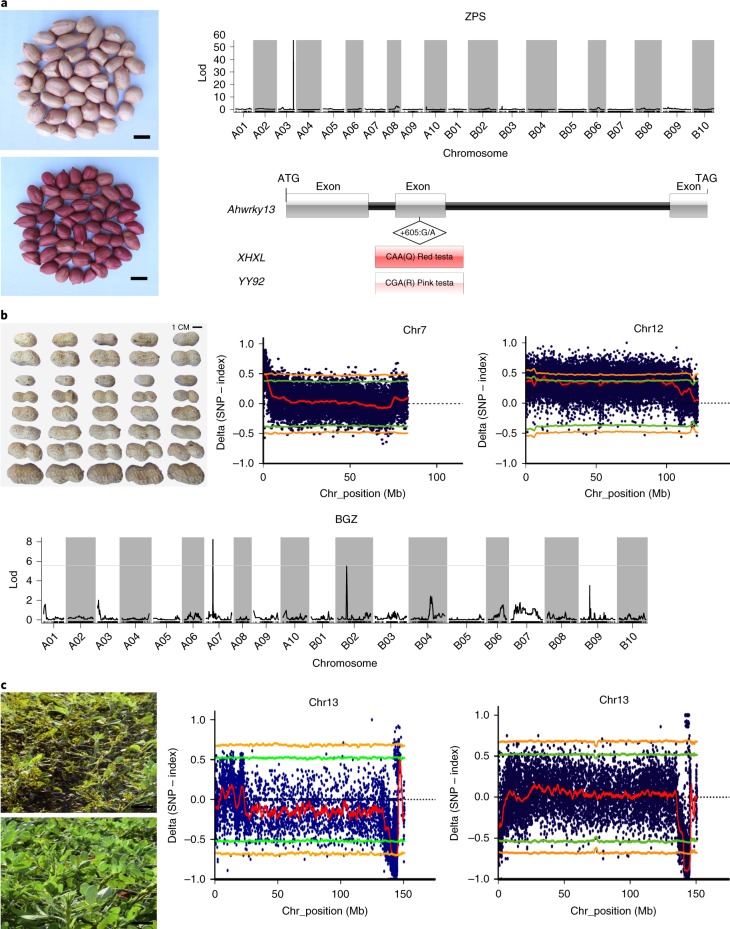
Candidate genes underlying seed size and color and foliar disease resistances. **a**, Seeds with red and pink testa color, linkage mapping and the candidate-gene model with SNPs. Scale bars indicate 1 cm. **b**, Phenotypes of RILs with pod size segregation, BSA mapping by resequencing and QTL mapping of pod size (100 pod weight in RILs (Yueyou 92 × Xinhuixiaoli)). Seed size QTLs were mapped on Chr07 (A07) and Chr12 (B02) using genetic mapping and QTL-seq approaches. Scale bar indicates 1 cm. **c**, Phenotypes of LLS-susceptible and LLS-resistant RILs from TAG 24 × GPBD 4. A Chr13 (B03) genomic region was mapped for both LLS and rust resistance. Scale bars indicate 5 cm.

The genome reveals candidate genes for many agronomically important peanut traits that have been genetically mapped (Supplementary Data 15). Through BLAST analysis using flanking DNA markers, 40 quantitative traits such as seed size, yield and quality, resistance and plant characters were mapped to pseudomolecules (Supplementary Fig. 18; Supplementary Dataset 15), revealing candidate genes. For example, red testa controlled by a single dominant gene was mapped to a region of 0.905 cM on chromosome 3 (Fig. 6a; Supplementary Dataset 16; Supplementary Dataset 17b). Candidate genes WRKYs (including WRKY13 with cosegregated R202Q; Fig. 6a), MYB and bHLH family and cytochrome 450 genes relating to anthocyanidin biosynthesis^41,42^ and anthocyanidin reductase and flavonoid 3′-monooxygenase of the anthocyanidin biosynthesis^41^ were found in the locus (Supplementary Note 6.2.4). Upregulation of anthocyanin synthesis genes (Supplementary Dataset 17c–e; Supplementary Notes 6.2.3 and 6.2.4) may cause red seed color.

Functional analysis of candidate genes promises new information on the regulation of peanut seed size, an important yield component. Fine QTL mapping and bulk segregant analysis (BSA) using a recombinant inbred line (RIL) population, Yueyou 92 and Xinhuixiaoli (Supplementary Notes 6.1.5 and 6.2.2), identified the same candidate regions on Chr07 (0.87–1.95 Mb in contigs 000199F) and Chr02 (4.41–5.91 Mb in 000164F) (Fig. 6b; Supplementary Dataset 18a,b; Supplementary Figs. 19 and 20), including 99 and 97 candidate genes, respectively. These 99 Chr07 genes included 19 candidates such as ABC transporters, oligopeptide transporter 5, histidine kinase 2, amino acid permease 3 and transcriptional regulator STERILE APETALA (SAP), which regulates seed development and seed size identity^43–45^. Histidine kinase 2 and SAP in *Arabidopsis* control shoot and seed growth and seed size^44,45^. Oligopeptide transporter 5, relating to embryo development and seed size, contained four missense SNPs between the two parents (Supplementary Data 18d; Supplementary Note 6.2.2). Expression of the SAP and nearby F-box genes were upregulated substantially in the embryos or pods of the large seeded parent and RILs by RNA-seq (Supplementary Dataset 18c; Supplementary Note 6.1.7). These 97 Chr12 genes on contig 000164 included an auxin transcription factor (ARF2) and three CYP78A6 playing key roles in seed size^46,47^, with CYP78A6 tandemly duplicated in the same region and ARF2 upregulated substantially in both the large seeded parent and RILs (Supplementary Dataset 18b,c). One SNP and one InDel differentiate the promoter region of CYP78A6 between large and small seeded parents (Supplementary Dataset 18e).

Resistance to two globally important foliar fungal diseases, leaf rust (caused by *Puccinia arachidis*) and late leaf spot (LLS) (*Cercosporidium personatum*), colocalizes to a common genomic region. A total of 1.73 billion high-quality reads of two parents (TAG 24 and GPBD 4, resistant and susceptible to both rust and LLS, respectively) and four resistant and susceptible pools from recombination inbred lines (RILs) revealed overlapping regions on Chr13 for rust (140.40–144.88 Mb) and LLS (140.80–144.71 Mb; Supplementary Table 12). This region harbored 216 (rust) and 171 (LLS) genes (Supplementary Dataset 19b,c), including TIR-NBS-LRR, pentatricopeptide repeat, glutathione *S*-transferase, serine/threonine kinase, and mitogen-activated protein kinase and calcium‐dependent protein kinase pathway genes. An R-gene cluster with two conserved Tir-NBS-LRR genes includes one (AH13G54010.1) with resistance co-segregating SNPs between resistant and susceptible parents and bulks (G143854163A, G143855518A for rust; C143855539T, G143855898C for LLS), tracing to *A. cardenasii* via resistant variety GPBD 4, through ICGV 86855 (interspecific derivative). Because no missense SNP was closely related to resistance, AH13G54010.1 might be a candidate for resistance to both diseases. This genomic region seems to be translocated from Chr03 to Chr13 after tetraploidization (Fig. 6c) because QTL-seq analysis using the *A. duranensis* genome assembly identified rust and LLS resistance on Aradu.A03 (ref. ^48^).

High oleic acid in seeds, contributing to better flavor and longer storage life of peanut products and benefitting human cardiovascular health, results from mutations in homeologous genes. We developed mutant lines with ~80% oleic acid from genetic backgrounds with only ~40% oleic acid (Supplementary Table 13), and resequenced two Min6-A from EMS treatment of Minhua 6 and Min8-A from γ-ray radiation of Minhua 8 (Supplementary Table 13; Supplementary Dataset 20). Both mutants differed from wild type by mutations in homeologous microsomal oleoyl-PC desaturase genes, ahFAD2A (dysfunction mutation on AH09G33970 at 114,779,221 bp of Chr09, G673A/D225N for FAD2A) and ahFAD2B (frameshift on AH19G43590 at 154464257bp of Chr19), which confer high oleate^49^ in Min8-B (Supplementary Dataset 20a,b). The mutations were experimentally validated by both near-infrared spectrum and chemical analysis and Sanger sequencing of another mutant AOM7a513 (Supplementary Fig. 21; Supplementary Table 13). Locations of ahFAD2A and ahFAD2B on Chr09 and Chr19 of the tetraploid genome explain that both happened simultaneously, leading to high oleic peanuts.

## Discussion

High oil and protein content and drought resilience (geocarpy) make peanut important for global food security. This high-quality genome assembly will accelerate breeding objectives, including improved yield and oil quality, and resilience to disease and abiotic stresses. Identification of mutations underlying large seeds and high oleate, as well as candidate genes or genomic regions for other important traits, provides insights into high-yield and quality formation and expedites breeding. The research community can now better capitalize on the value of peanut as a model for polyploid genome evolution and its contributions to improved yield, quality and resistance.

## Methods

A full description of the methods can be found in the Supplementary Information. No statistical methods were used to predetermine sample size. The genome-associated experiments were not randomized, and the investigators were carefully allocated during experiments and outcome assessment.

### Genome sequencing and assembly

#### Sequencing

DNA was extracted from leaf tissues of a single plant of *A. hypogaea* cv. Shitouqi (ssp. *A. h. fastigiata* var. *vulgaris*, the most widely cultivated peanut ecotype in the world) following a previously published protocol^50^ and purified with Beckman Coulter Genomics AMPure XP magnetic beads. DNA quality was assessed by agarose gel electrophoresis and NanoDrop 2000c spectrophotometry, followed by Thermo Fisher Scientific Qubit fluorometry. A total of 204 single-molecule real-time cells were run on the PacBio RS II system, and 14 cells on the Sequel system, with P6/C4 chemistry (Supplementary Note 1.2), thus producing 270.5-Gb subreads with a coverage of 100× of the peanut genome.

#### Assembly

De novo assembly was developed on a large-scale Tanhe computer using the diploid assembly FALCON (https://github.com/PacificBiosciences/FALCON)^51^, including PacBio raw read correction, preassembly and contigs construction. The draft assembly contigs were followed by error correction using PacBio reads with the quiver algorithm^52^. DNA was also sequenced using an Illumina HiSeq 2000 machine, and the quivered contigs were further polished by Illumina reads. Finally, potential contaminations were screened against National Center for Biotechnology Information bacteria, virus database and human genome to form the final contig assembly.

#### Three-dimensional chromatin conformation capture sequencing

To generate physical scaffolds for genome assembly, we generated Hi-C sequencing data by adapting published procedures^53^ (Supplementary Note 1.4.1). In brief, freshly harvested leaves were cut into 2- to 3-mm pieces and infiltrated in 2% formaldehyde, and crosslinking was stopped by adding glycine. The tissue was ground to powder and suspended in nuclei isolation buffer to obtain a nuclei suspension. Nuclei were digested with *Hind*III as previously described^18^, marked by incubating with Klenow enzyme and biotin-14-dCTP^17^ generating blunt-end-repaired DNA strands, and ligated by T4 DNA polymerase. The extracted DNA was mechanically sheared to 200–300 bp sizes by ultrasound followed by size fractionation using AMPure XP beads. DNA fragments of 150–300 bp were blunt-end repaired and A-tailed, followed by purification through biotin–streptavidin-mediated pulldown^18^. PCR amplification was performed after adapters were ligated to the Hi-C products. The PCR products were purified with AMPure XP beads, and the Hi-C libraries were quantified by quantitative PCR for Illumina HiSeq X-ten PE150 sequencing^17^.

#### Scaffolding the PacBio assemblies with LACHESIS

Hi-C unique paired-end sequence data were used to scaffold the PacBio assembly contigs using a software pipeline LACHESIS^24^. The Hi-C sequences were aligned to the draft contig assemblies. The separations of Chicago read pairs mapped within draft contigs were clustered by agglomerative hierarchical clustering producing chromosomic groups. The contigs within the groups were constructed as trunks based on interaction strength among contigs, by selecting the most dependable trunks as roots for adding the rest contigs into suitable positions and producing a group with correct contigs order. Finally, the orientations of contigs within chromosomal groups were determined using weighted directed acyclic graph (WDAG)^18^ based on interaction strength between two contig directions.

#### RIL population mapping and marker analysis

Two RIL mapping populations of 978 F_9_ lines and 343 F_12_ lines were developed from the same crosses of Yueyou 92 (*A. hypogaea* var. *vulgaris*) and Xinhuixiaoli (*A. hypogaea* var. *fastigiata*) at different times by single-seed descent starting from F_2_ generation. Specific locus-amplified fragment sequencing was performed with DNA from the parents and randomly chosen 314 RIL_12_ lines and 267 RIL_9_ lines using the specific locus-amplified fragment SNP calling method^54^ (Biomarker Company), and sequencing reads were mapped to the reference genome (http://peanutbase.org/) using SOAP^55^. SNPs were called in the parents and in the RIL lines. Genotype calls were generated for every line of the two populations by summing up read counts. Markers were assigned to linkage groups by HighMap^56^. The order of the markers was determined using the maximum likelihood algorithm. Regression mapping in HighMap was used to determine the centimorgan distances per genetic linkage group.

#### Integrated linkage maps

The two dense SNP maps described earlier were integrated with two previously published peanut linkage maps^19,20^ (one 1,619-SNP linkage map and one refined integrated map containing 1,954 simple sequence repeat (SSR) or transposon markers after removing some contradictory markers) by ALLMAP software^21^ based on assembling the contig sequences of the STQ genome. The maps of Yueyou 92 × Xinhuixiaoli were set as the highest priority in the integration. The assumption is that at the 100-kb bin level recombination should essentially be zero. On this level, a regression of centimorgan position on both maps yielded *R*^2^ values > 0.85 and often >0.9, so the regression line could easily be used for interpolating the positions of the alternative map toward the corresponding position on the Yueyou 92 × Xinhuixiaoli map. All Yueyou 92 × Xinhuixiaoli markers went into the integrated map on their original position.

#### Constructing chromosome pseudomolecules

The construction of pseudomolecules followed an automated procedure by the integration of the following datasets: (1) sequence assemblies of 7,232 contigs, (2) the high-density integrated linkage map with 14,619 markers as described earlier, and (3) Hi-C data with valid pair-end reads of 31,734,151 covering more than 99.6% of the total length of contigs sequences. Specifically, Hi-C data were used to map the contigs and clustered the contigs into scaffolds using the software LACHESIS^24^. Then using the Hi-C alone assembled map and the integrated linkage maps, we assembled the whole chromosomes by ALLMAP software^21^ with a priority of Hi-C assembled map versus integrated genetic map being 1:1. The Hi-C assembled chromosomal scaffolds were optimized for the arrangements and orientation of contig trunks in this step together with manual adjustment. Subsequently, the pseudomolecules were generated by concatenating the adjacent contig sequences with 100 ‘N’s, and were oriented and numbered in accordance with previously published maps^1^. Finally, all contig sequences not anchored to chromosomes were constructed with 100 ‘N’s as linkers following the order of contig sizes.

#### Validation of *A. hypogaea* genome assemblies with BACs

To validate the genome assembly, we downloaded a total of 1,576 public BAC end sequence (BES) records of *A. hypogaea* in GenBank GSS database (FI498696.1 to FI503143.1) for analysis. These BESs were aligned to the reference genome through BLASTN with the criterion of >95% aligning identity, >90% aligned coverage for BESs, not located in pseudochromosome Chr00 and the insert size lower than 200 kb. The insertion lengths between a matched pair of BAC end sequences within the genome are about a 110-kb span on average (Supplementary Note 1.8). We also performed all-to-all alignment (-minIdentity = 80–99, -minScore = 100, --fastMap) of three available peanut BACs released in the GenBank^22^ and the assemblies using BLAT.

#### Annotation of genome and transcribed regions

Gene models were predicted using EuGene 4.2 embedded in a fully automated pipeline^57^. The annotation of the peanut genome assemblies was based on four datasets that included: (1) RNA-seq data (Supplementary Dataset 5a); (2) reference protein predictions from *Arachis ipaensis*^1^, *Arachis duranensis*^1^, *Glycine max*^30^, *Medicago truncatula* and *Phaseolus vulgaris*, as well as A*rabidopsis thaliana*^58^ from Phytozome^8^; (3) previously released transcriptome 454 sequencing data (complementary DNA) sequences (SRR1367372, SRR1368960, SRR1371390, SRR1377239); and (4) newly generated peanut PacBio Iso-Seq data. The RNA-seq datasets were derived from a total of 29 different tissues and conditions (Supplementary Notes 2.1 and 2.2). The full-length transcriptome data were derived from the 29 evenly mixed previously described RNA samples and were generated by the Iso-Seq method (Supplementary Note 2.3) for supporting annotation.

AUGUSTUS, SNAP and GeneMark^59^ were used for ab initio gene prediction, using model training based on coding sequences from *A. ipaensis*, *A. duranensis*, *G. max* and *A. thaliana*. RNA-seq and Iso-Seq reads were mapped onto the reference genome using TopHat^60^ and Bowtie 2 (ref. ^61^), respectively. Hints with locations of potential intron–exon boundaries were generated from the alignment files with the software package BAM2hints in the MAKER package^62^. MAKER with AUGUSTUS was then used to predict genes in the repeat-masked reference genome. Genes were characterized for their putative function in the UniProt and KEGG databases. Completeness of gene spaces was evaluated with the BUSCO pipeline^24^.

Genome-wide prediction of ncRNAs, such as rRNA, small nuclear RNA and miRNA, was performed in Rfam^63^. tRNA and rRNA were identified using tRNAscan-SE, and RNAmmer and miRNA were predicted using miRanda version 3.0 (http://www.microrna.org).

#### Annotation of repeat region

Conserved BLASTN search in Repbase and de novo prediction were performed to annotate repeat sequences. Repeat families were first de novo identified independently and classified using RepeatModeler^64^ (see Supplementary Note 2.4). RepeatMasker^65^ was used to search and identify the repeats within the genomes. Repeats annotation in Repbase was also performed by RepeatMask, RepeatProteinMasker and TRF software and merged with de novo annotation.

#### Gene differential expression analysis

The normalized counts of gene expression were estimated using Cufflinks package based on the TopHat^60^ output results of the 29 samples’ RNA-seq data analysis as described earlier. The fragments per kilobase per million mapped reads (FPKM) values of expression genes were calculated. The differential expression between homeologous genes was identified by FPKM if their fold change (FPKMa/FPKMb) was greater than 2 and the false discovery rate was ≤0.01.

#### Syntenic analysis of peanut and its wild diploid genomes

To identify chromosome structural changes between tetraploid peanut and two wild diploids, we analyzed subgenome synteny by plotting the positions of homeologous pairs of A- and B-subgenome within the context of the 20 chromosomes using Circos^66^/MCScanX^67^ with at least five syntenic genes. Synteny of the A- and B-subgenomes versus diploid A and B genomes was compared, respectively, using the same software. To differentiate chromosome recombinations within the two subgenomes after tetraploidization, we also performed synteny of the two diploid genomes for comparison (Fig. 2b).

#### Orthologous regions between peanut and the diploid species

To determine the variations of chromosome insertions, deletions or substitutions, we identified orthologous regions in cultivated peanut and the two diploid species (https://peanutbase.org) by BLASTN searches of the peanut genome using the Molecula of contigs from each diploid genome individually. The similarity between the peanut subgenomes and the diploid species *A. duranensis* and *A. ipaensis* was presented in dot figures proportional to chromosome sizes (Supplementary Dataset 7; Supplementary Note 3.3.4). Segmental relationships along chromosomes were identified by reciprocal comparisons.

#### Genomic comparison of *A. hypogaea* with other legume species and *V. vinifera*

To investigate the origin and evolution of peanut genome, the evolutionary relationships of peanut, we compared its diploid ancestors and other genome-sequenced legume species including *G. max*, *P. vulgaris* and *M. truncatula*, as well as *V. vinifera*. We identified homologous proteins between *A. hypogaea* and five other legume genomes using BLASTP^68^ (E value 1 × 10^−5^) and scanned syntenic blocks consisting of homologous genes among the 11 genomes including *V. vinifera* using MCScanX^67^ with at least five syntenic genes (Supplementary Fig. 10; Fig. 3; Supplementary Table 7). To reconstruct the chromosomal evolution model of *A. hypogaea*, we inferred 16 legume basic chromosomes before LCT from *V. vinifera*, then constructed 16 legume common chromosomes (called Lu) after LCT from *P. vulgaris*, and then reconstructed the ancestral A and B genomes chromosome from Lu, which hybridized and evolved to 20 *A. hypogaea* chromosomes using the precise analysis of co-linear relationships.

#### Identification of orthologous genes

Orthologous gene families among peanut, two wild species (*A. ipaensis* and *A. duranensis*), and several other plant species including *P. vulgaris*, *M. truncatula* and *G. max*, as well as *A. thaliana*, were identified using the OrthoMCL pipeline^69^. The longest protein prediction from each gene was selected. Pairwise sequence similarities between all input protein sequences were calculated using BLASTP^68^ with an E value cutoff of 10^−5^. Markov clustering of the resulting similarity matrix was used to define the ortholog cluster structure of the proteins. Comparative analysis of gene families and the copy numbers was performed among peanut and the other species for visualization with InteractiVenn using Custom Perl scripts^70^ (Fig. 4; Supplementary Fig. 11). Individual gene trees were then constructed using the maximum likelihood method using Mega^71^. Changes of three important peanut gene families or pathways, such as fatty acyl metabolism, R gene and nitrogen symbiosis fixation, were analyzed in greater detail using BLAST searches, as well as GenomeThreader mappings to the peanut reference genome.

#### Identification of nonredundant and duplicated genes in allotetraploid peanut genome

Nonredundant genes in the cultivated peanut genome were identified based on the BLAST results of protein-coding genes between the two subgenomes. In brief, protein sequences extracted from A subgenome were aligned using BLAST against proteins from B subgenome, and vice versa. The best matches were retained and formatted to a two-column table of homeolog pairs. Duplicated genes were classified into two categories: (1) tandem duplicated genes if the multiple copies were consecutively located in the neighborhood, and (2) dispersed duplicated genes if not tandem.

#### Acyl-lipid genes in cultivated peanut genome

The protein sequences of all the annotated gene models from peanut and the two ancestors (https://peanutbase.org) were aligned to two acyl-lipid gene datasets (885 *A. thaliana* and 829 soybean acyl-lipid genes; http://aralip.plantbiology.msu.edu/, https://www.soybase.org/) by BLASTP with an E value < 10^−6^ and matching length ≥50%. Oil-related QTLs^34,35,72^ in peanut were also searched to find orthologs within those QTL regions. The identified acyl-lipid orthologs of three peanut species, also those from soybean, oil palm and rapeseed, were assigned to gene families by OrthoMCL (inflation value, 1.5) with default parameters^69^. The orthologous acyl-lipid genes in peanut were associated with RNA-seq expression data, and a weighted gene coexpression network analysis^73^ was performed using R software (https://horvath.genetics.ucla.edu/html/CoexpressionNetwork/). Results were visualized by using Cytoscape software^74^. To investigate enriched functions of identified coexpression modules, we used FatiGO to perform gene ontology enrichment analysis^75^.

#### NBS-LRR encoding genes

NBS-encoding R genes in genomes of *A. hypogaea*, *A. duranensis* and *A. ipensis*, and also soybean, were screened using the Hidden Markov Model (HMMER3.0) and BLASTP. HMMER3.0 (ref. ^76^) was used to search for the Pfam NBS (NB-ARC) family PF00931 domain (cutoff E value < 1 × 10^−10^). BLASTP^77^ was used to search TIR or no-TIR domain-containing NB-ARC R genes for class discrimination. Statistics were made in Excel to tell the changes and differentiation (Supplementary Dataset 10). The classification and the evolution were predicted by phylogenetic analysis as following description. The localization of R genes was mapped among the reference genome using Circos^66^.

#### Nitrogen symbiosis-related gene

Nodulation-related genes were collected from two recent studies^78,79^ in *M. truncatula*, *L. japonicus*, and *G. max*. The protein sequences of nodulation-related genes were retrieved from 12 legume species (National Center for Biotechnology Information: https://www.ncbi.nlm.nih.gov) and peanut. The orthologs were first determined by using BLASTP, BBH and OrthoMCL^69^ (inflation value of 1.5 and other settings default).

#### Resequencing

Fifty-two accessions were chosen for DNA resequencing covering cultivated peanut, wild species and artificial tetraploids (Supplementary Dataset 12; Supplementary Note 5.1.1). All sequencing was performed with a HiSeq 2500 machine (Illumina), using 150-bp paired-end libraries. The raw reads from 52 accessions were filtered using trimmomatic v.0.36 and mapped to the reference genome using BWA-MEM^80^. Variants were called using HaplotypeCaller and GenotypeGVCFs of Genome Analysis tool kit (GATK) v.3.8. The obtained SNPs were filtered using GATK filters^81^ followed by HAPLOSWEEP v.1.0 to remove homeologous SNPs. The identified InDels were filtered using GATK filters. For phylogenetic analysis, the phylogenetic tree was constructed by SNPhylo^82^ (maximum likelihood method and 1,000 bootstraps) using the filtered SNPs. Admixture and PCA were performed for the 52 accessions (Supplementary Notes 5.3 and 5.4).

#### Phylogenetic analysis of ARF

To identify ARF homologs, we used the protein sequence from the *A. hypogaea* ARF gene as a BLAST query. Filtering for hits with an E value < 1 × e^−5^, identity of 50% with RNA-seq evidence resulted in the identification of 114 peanut proteins, with 28 and 28 proteins from AA subgenome and BB subgenome, respectively. For the construction of the phylogenetic tree, protein sequences from these 114 peanut ARF homologs were aligned using Clustal Omega^83^ along with the above diploid gene models. Phylogenetic analysis was performed with MEGA^84^ (v.6.06). The final tree was estimated using the maximum likelihood method with a bootstrap value of 1,000 replicates.

#### Phylogenetic analysis of R genes

The alignment of NBS domains of R gene was performed with Clustal Omega^83^, using released sequences in NCBI (accessions FI498696.1 to FI503143.1). There are 661 R genes in the peanut reference genome. MEGA^84^ software (v.6.06) was used to perform phylogenetic analysis. The maximum likelihood method was used to infer the phylogeny based on the Jones–Taylor–Thornton (JTT) matrix-based model^71^.

#### Phylogenetic analysis of nodulation genes

A phylogenetic tree was constructed by MEGA6 software^84^ using four nodulation genes found in all species for nodulation evolution and phylogenetic analysis. The best model was selected from Model Selection using the maximum likelihood method with 1,000 bootstrap replications.

#### Integrating main QTLs to the reference genome

Scores of previously published reports were searched for QTLs involving 40 traits covering peanut economically favorable traits and plant growth and development (Supplementary Dataset 15). Their specific positions were identified by BLASTN alignments using the flanking markers of QTLs. A total of 136 main QTLs with ~8%–71% of phenotype variance explanation were mapped to the peanut genome assembly. A 4 Mb sequence was considered at the flanks of the mapped markers to define the QTL coordinates and assess colocalization with candidate genes (Supplementary Dataset 15).

### Seed sizes and testa color gene analyses

#### QTL mapping

A population was developed by crossing Yueyou 92 (pink testa, big seeds) and Xinhuixiaoli (red testa, small seeds) as mentioned earlier. Real hybrids were identified in F_1_ plants with red testa, a dominant trait. First phenotyping of seed color was performed on 752 individual plants in the F_2_ generation. Phenotyping of seed size and testa color were characterized in the RIL population of 267 lines containing 20 plants each, three replications for at least 2 years (Nature Research Reporting Summary). The QTLs for seed size and testa color were mapped to the reference genome based on the genetic maps with 7,134 SNP markers derived from the population of 267 lines using the composite interval mapping (CIM) method of QTL IciMapping^85^. Candidate genes were searched by flanking DNA markers of QTLs (Supplementary Note 6).

#### Candidate-gene evaluation

Candidate genes for seed size and testa color within QTL regions were fine-mapped and evaluated based on QTL-seq in BSA and RNA-seq (Supplementary Notes 6.2.3 and 6.1.7), together with bioinformatics analysis. We mapped key genes by sequencing comparison with both randomly chosen RIL lines with big and small seeds, and natural varieties with big and small seeds. We also identified a key gene using RNA-seq analysis. Genes differentially expressed between RILs with big and small seeds or pink and red testa were selected.

### Bulk segregation analysis for seed size and foliar disease resistance

The QTL-seq analysis was conducted from two RIL populations using the multiseason phenotyping data for three traits, namely, pod weight, rust resistance and LLS resistance. In brief, the bulks were made by pooling DNA from selected RILs with extreme phenotypes for these traits. For pod weight, the DNA from 54 RILs possessing low pod weight and 54 RILs with high pod weight were pooled from the population (Yueyou 92 × Xinhuixiaoli). Similarly, DNA from 25 RILs each for resistance and susceptible RILs (TAG 24 × GPBD 4) were pooled to constitute four bulks, that is, resistant bulk for rust and LLS, and susceptible bulk for rust and LLS, respectively. The resistance parent GPBD 4 was an interspecific derivative of *A. cardenasii*, that is, the resistance source for both of the diseases. Together with four parents, a total of ten DNA samples were sequenced on Illumina HiSeq 2500. The sequencing data were analyzed using the QTL-seq pipeline^86^ (http://genome-e.ibrc.or.jp/home/bioinformatics-team/mutmap) for calculating the SNP-index using the tetraploid genome assembly developed and reported in this article. The ∆SNP-index for each trait was then calculated by subtracting the SNP-index of one bulk from that of another bulk. The candidate-gene discovery was performed in the genomic regions with the highest ∆SNP-index.

### Reporting Summary

Further information on research design is available in the Nature Research Reporting Summary linked to this article.

## Online content

Any methods, additional references, Nature Research reporting summaries, source data, statements of code and data availability and associated accession codes are available at 10.1038/s41588-019-0402-2.

## Supplementary information

Supplementary Information Supplementary Notes 1–7, Supplementary Figs. 1–21, Supplementary Tables 1–13 and Supplementary Datasets 1, 2, 7, 8 and 14

Reporting Summary

Supplementary Dataset 3 Integrated linkage map information.

Supplementary Dataset 4 Validation of pseudomolecules.

Supplementary Dataset 5 RNA-seq, miRNA and duplicated genes.

Supplementary Dataset 6 Contig assignment.

Supplementary Dataset 9 Orthologous gene cluster.

Supplementary Dataset 10 R-gene statistics.

Supplementary Dataset 11 Acyl-lipid and nodulation-related genes.

Supplementary Dataset 12 Resequencing genotypes.

Supplementary Dataset 13 Distribution of SNP and InDel of 52 accessions.

Supplementary Dataset 15 QTL mapping regions of 40 traits.

Supplementary Dataset 16 Construction of YX-map with high density of SNP markers.

Supplementary Dataset 17 Testa color phenotype and candidate genes data.

Supplementary Dataset 18 Seed size phenotype and candidate genes within mapping region.

Supplementary Dataset 19 LLR and RR candidate regions.

Supplementary Dataset 20 SNPs and InDels of original and high oleate mutant variety.

Supplementary Dataset 21 Peanut accession IDs and URLs for access to datasets.

## Data Availability

Genome assemblies and resequencing data were available in BioProject of GenBank under accession numbers PRJNA480120 and SRR7617992, etc. (see Supplementary Data 21), respectively. The genome assemblies and annotations, transcriptome and PacBio Iso-Seq reads can also be accessed at http://peanutgr.fafu.edu.cn and http://peanutgr.fafu.edu.cn/Download.php. All materials and other data in this study are available upon reasonable request.
