# Supplementary material for: The genome of cultivated peanut provides insight into legume karyotypes, polyploid evolution and crop domestication
Source: Nat Genet. 2019 May 1;51(5):865–76. doi: 10.1038/s41588-019-0402-2 (PMC7188672; doi:10.1038/s41588-019-0402-2)
Supplement: Supplementary file 1 — Supplementary Notes 1–7, Supplementary Figs. 1–21, Supplementary Tables 1–13 and Supplementary Datasets 1, 2, 7, 8 and 14 [file 41588_2019_402_MOESM1_ESM.pdf]

nature genetics
SUPPLEMENTARY INFORMATION
https://doi.org/10.1038/s41588-019-0402-2

In the format provided by the authors and unedited.

The genome of cultivated peanut provides insight into legume karyotypes, polyploid evolution and crop domestication

Weijian Zhuang[1,24,25]*, Hua Chen[1,24], Meng Yang[2,24], Jianping Wang[3,4,24], Manish K. Pandey[5], Chong Zhang[1], Wen-Chi Chang[6,7], Liangsheng Zhang[3], Xingtan Zhang[3], Ronghua Tang[8], Vanika Garg[5], Xingjun Wang[9], Haibao Tang[3], Chi-Nga Chow[6,7], Jinpeng Wang[10], Ye Deng[1], Depeng Wang[2], Aamir W. Khan[5,11], Qiang Yang[1], Tiecheng Cai[1], Prasad Bajaj[5], Kangcheng Wu[1,3], Baozhu Guo[1,12], Xinyou Zhang[13], Jingjing Li[2], Fan Liang[2], Jiang Hu[2], Boshou Liao[14], Shengyi Liu[14], Annapurna Chitikineni[5], Hansong Yan[3], Yixiong Zheng[1,15], Shihua Shan[9], Qinzheng Liu[1], Dongyang Xie[1], Zhenyi Wang[10], Shahid Ali Khan[1], Niaz Ali[1], Chuanzhi Zhao[9], Xinguo Li[9], Ziliang Luo[4], Shubiao Zhang[1,16], Ruirong Zhuang[1], Ze Peng[4], Shuaiyin Wang[1], Gandeka Mamadou[1], Yuhui Zhuang[1,17], Zifan Zhao[4], Weichang Yu[18], Faqian Xiong[8], Weipeng Quan[2], Mei Yuan[9], Yu Li[1,16], Huasong Zou[1], Han Xia[9], Li Zha[1], Junpeng Fan[2], Jigao Yu[10], Wenping Xie[1], Jiaqing Yuan[10], Kun Chen[1], Shanshan Zhao[1], Wenting Chu[1], Yuting Chen[1], Pengchuan Sun[10], Fanbo Meng[10], Tao Zhuo[1], Yuhao Zhao[10], Chunjuan Li[9], Guohao He[19], Yongli Zhao[19], Congcong Wang[15], Polavarapu Bilhan Kavikishor[20], Rong-Long Pan[1,21], Andrew H. Paterson[10,22], Xiyin Wang[10,25]*, Ray Ming[3,23,25]* and Rajeev K. Varshney[5,11,25]*

[1]Fujian Provincial Key Laboratory of Plant Molecular and Cell Biology, Oil Crops Research Institute, State Key Laboratory of Ecological Pest Control for Fujian and Taiwan Crops, Fujian Agriculture and Forestry University, Fuzhou, China. [2]Nextomics Biosciences Institute, Wuhan, China. [3]Haixia Institute of Science and Technology, Fujian Agriculture and Forestry University, Fuzhou, China. [4]Agronomy Department, University of Florida, Gainesville, FL, USA. [5]Center of Excellence in Genomics & Systems Biology, International Crops Research Institute for the Semi-Arid Tropics (ICRISAT), Hyderabad, India. [6]College of Biosciences and Biotechnology, National Cheng Kung University, Tainan, Taiwan. [7]Graduate Program in Translational Agricultural Sciences, National Cheng Kung University and Academia Sinica, Taipei, Taiwan. [8]Guangxi Academy of Agricultural Sciences, Nanning, China. [9]Biotechnology Research Center, Shandong Peanut Research Institute, Shandong Academy of Agricultural Sciences, Shandong, China. [10]North China University of Science and Technology, Tangshan, China. [11]The University of Western Australia, Perth, Western Australia, Australia. [12]USDA-ARS, Crop Protection and Management Research Unit, Tifton, GA, USA. [13]Henan Academy of Agricultural Sciences, Zhengzhou, China. [14]Oil Crops Research Institute of the Chinese Academy of Agricultural Sciences, Wuhan, China. [15]Zhongkai University of Agriculture and Engineering, Guangzhou, China. [16]College of Crop Sciences, Fujian Agriculture and Forestry University, Fuzhou, China. [17]School of Life Science, TsingHua University, Beijing, China. [18]Guangdong Provincial Key Laboratory for Plant Epigenetics, College of Life Sciences and Oceanography, Shenzhen University, Shenzhen, China. [19]Tuskegee University, Tuskegee, AL, USA. [20]Osmania University, Hyderabad, India. [21]College of Life Science, National Tsing Hua University, Hsin Chu, Taiwan. [22]Plant Genome Mapping Laboratory, University of Georgia, Athens, GA, USA. [23]Department of Plant Biology, University of Illinois of Urbana-Champaign, Urbana, IL, USA. [24]These authors contributed equally: Weijian Zhuang, Hua Chen, Meng Yang, Jianping Wang. [25]These authors jointly supervised this work: Weijian Zhuang, Rajeev K. Varshney, Ray Ming, Xiyin Wang. *e-mail: weijianz@fafu.edu.cn; wangxiyin@vip.sina.com; rayming@illinois.edu; r.k.varshney@cgiar.org

Supplementary Notes

S. Note 1. Reference genome sequencing and assembly 3
S1.1 Plant material 3
S1.2 PacBio sequencing and assembly 3
S1.3 Illumina sequencing and quivering 4
S1.4 HiC library preparation and sequencing 4
S1.5 Scaffolding the PacBio assembly with the Hi-C information 6
S1.6 Integrating genetic maps using ALLMAPS 6
S1.7. Improving the Hi-C scaffolds with integrated genetic mapping 7
S1.8. Validating the reference genome assembly 8
S. Note 2. Transcriptome sequencing and reference genome annotation 9
S2.1. Plant growth and RNA extraction 9
S2.2 Transcriptome sequencing 10
S2.3 PacBio Isoseq data generation and correction 11
S2.4 Ab initio gene model prediction 12
S2.5 Characterization of repetitive sequences 13
S2.6 Validation of gene models with BUSCO 14
S2.7 Non-coding RNAs prediction 14
S2.8 Analysis of homeologs in an allotetraploid peanut genome 14
S. Note 3. Comparative genomic analysis 16
S3.1 Methods 16
S3.2 Genome comparison between the A and B sub-genome of *A. hypogaea* 17
S3.3 Genome comparison of *A. hypogaea* with diploid *A. duranensis* and *A. ipaensis* 18
S3.4 Genomic comparison of *A. hypogaea* with other legume species 20
S3.5 Reconstruction of *A. hypogaea* chromosomes from an inferred legume common ancestor 20
S. Note 4. Gene family analysis 22
S4.1 Clustering of gene families by OrthoMCL 22
S4.2 Analysis of the R-gene super family 23
S4.3 Analysis of Acyl metabolic related genes 24
S4.4 Analysis of nodulation related genes 26
S. Note 5. Re-sequencing and phylogenetic analysis 27
S5.1 Plant material and Illumina sequencing 27
S5.2 History of peanut origin 28
S5.3 Phylogenetic analyses 30
S5.4 Circos and other analysis of peanut accessions 31
S. Note 6. Linkage mapping, BSA and QTL discovery 31
S6.1 Yueyou 92 × Xinhuixiaoli populations 31
S6.2 Pooled-sequencing based candidate gene discovery 35
S. Note 7. Mutant analysis and candidate gene discovery for high oleic acid 37
S7.1 Plant material 38
S7.2 Methodology of mutant analysis 38
S7.3 Candidate gene discovery by analyzing high oleic acid mutants 39

Supplementary Note 1 Reference genome sequencing and assembly

S1.1 Plant material

We sequenced cultivated tetraploid peanut (*Arachis hypogaea*) var. Shitouqi (accession zh.h0235; subspecies *fastigiata* and *vulgaris* botanical type), the most widely cultivated peanut ecotype in the world. All genome analyses herein were performed using accession zh.ho235, Shitouqi, unless noted otherwise. This accession is a well-known cultivar and parent in China, publicly available from the Chinese Crop Germplasm Resources Information System (http://www.cgris.net/) of the Chinese Agriculture Academy, Peoples Republic of China. Nearly 70% of Chinese bred cultivars have consanguinity with Shitouqi. (Accession IDs and URLs for access to datasets see **Supplementary Data Set 21**)

S1.2 PacBio sequencing and assembly

DNA was extracted from young leaf tissues of a single plant with dark treatment for 24 h before harvest. DNA was prepared as described in the "Procedure & Checklist - Preparing Genomic DNA for Large Insert SMRTbell® Libraries from Arabidopsis" protocol (https://www.pacb.com/wp-content/uploads/Procedure-Checklist-Preparing-Genomic-DNA-for-Large-Insert-SMRTbell-Libraries-from-Arabidopsis.pdf). DNA was purified with Beckman Agencourt AMPure XP magnetic beads and assessed by PFGE and Invitrogen Qubit Fluorometry. A total of 219 Single-Molecule Real-Time (SMRT) cells (205 from PacBio RSII and 14 from PacBio Sequel) were run with the P6-C4 chemistry (RSII) and Sequel DNA Polymerase 2.0 as well as Sequel Sequencing Plate 2.0 (Sequel) by NextOmics Biosciences Co., Ltd (Wuhan, China). A total of 29,234,185 PacBio post-filtered reads were generated from the 218 SMRT cells. This produced a total of 270,461,987,014 bp single-molecule sequencing data, with an average read length of 10.25 Kb. De novo assembly was conducted using the PacBio diploid assembler FALCON (https://github.com/PacificBiosciences/FALCON) with the setting GENOME_SIZE = 2,700,000,000. FALCON started with the step of filtering SMRT reads with parameter (Options--filter='MinReadScore=0.80, MinSRL= 500, MinRL=100'), and then performing error correction and preassembly and contig construction with parameters (the length_cutoff and length_cutoff_pr set to 13 kb and 12 kb, respectively). The draft assembled genome size, contig N50, and contig number were 2,515,629,439, 1,509,423 bp and 7,232,

respectively. The draft assembly was then improved to obtain the final assembly using the quiver algorithm as follows.

S1.3 Illumina sequencing and quivering

For heterozygosity estimation and PacBio genome assembly quivering, high quality genomic DNA for WGS sequencing was extracted using leaf tissue from the same single plant using CTAB (Cetyltrimethyl ammonium bromide) method[86]. The 400-bp paired-end (PE) libraries were prepared using the NEBNext Ultra DNA Library Prep Kit. Sequencing was performed through HiSeq 2500 at WuXi NextCODE (Shanghai, China). The adapter sequences, leading and trailing bases were removed with Trimmomatic (v0.35)[87]. To further correct FALCON assemblies, two steps were included: first, using the pbalign and Quiver software from PacBio (https://github.com/PacificBiosciences), the corrected PacBio reads were aligned to the assembled genome for correction; second, Illumina NGS reads were aligned to the genome by BWA[88] and then Pilon[89] software was used to polish the genome and get an improved version. The final assembled genome size is 2,538,408,906 bp, 94% of the peanut genome size estimated from K-mer analysis (**Supplementary Note Fig. 1.3**), with contig N50 and contig number as 1,509,423 bp and 7,232, respectively **(Table 1)**.

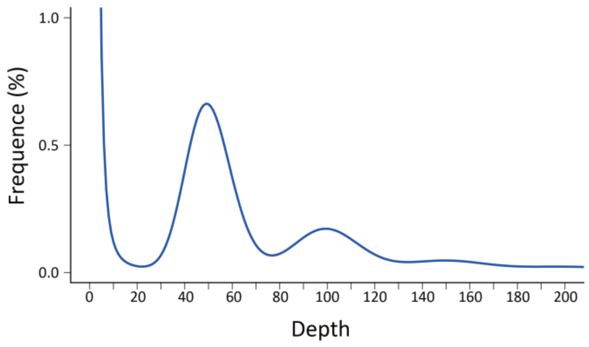

Supplementary Note Figure 1.3. Genome size estimation from K-mer analysis for *A. hypogaea*. The 17K-mer frequency distribution of *A. hypogaea* is showed about. The secondary peak at twice the main peak depth is indicative of high repeat content. Genome size is estimated from the depth of the main peak as below. The genome size is estimated using the formula: Genome Size = K-mer_num/Peak_depth. Here the K-mer number is 135,325,292,080, K-mer is 17, Peak depth is 50, and therefore, the estimated genome size is 2,706,505,842 bp.

S1.4 HiC library preparation and sequencing

S1.4.1 Plant material and chromatin crosslinking. Peanut plants (*A. hypogaea* cv. Shitouqi) were grown in a greenhouse for 10-14 days to 3-4 leaves. Freshly harvested three leaf samples (2-3 g, each) were cut into 0.1x1 cm pieces, transferred into a 50 ml tube with 2% formaldehyde solution, vacuum infiltrated and supplemented crosslinking for 15 mins. The crosslinking was stopped by adding 2 M glycine to 0.125 M for 5

min at 20 ℃, and then putting on ice for 15 mis to fix the crosslinking, filtering off water and keeping at -70 ℃ for DNA extraction.

S1.4.2 Hi-C fragment preparation. The materials were ground in liquid nitrogen, and used for nuclei isolation. Isolated nuclei were purified and checked for quality and quantity. The nuclei mixture was dissolved, the chromatin was digested with *Hind*III and marked with biotin-14-dCTP (Thermo Fisher Scientific), and the DNA molecules were made blunt-end. The blunt-end DNA was ligated for the Hi-C library and the subsequent DNA was purified. The concentration of the ligation products was determined using a Qubit Fluorometer (Life Technologies). Ligation of filled-in *Hind*III sites (AAGCTT) results in the creation of sites for the restriction enzyme *Nhe*I (GCTAGC). The ligated DNA were subjected to the exonuclease activity of T4 DNA polymerase to remove the biotin-14-dCTP from non-ligated DNA ends, and the DNA was purified by phenol: CHCl3 (1:1) extraction, precipitated and washed. The purified DNA was fragmented using a Covaris S220 device (Covaris Ltd.) and was size fractionated using standard 2 % agarose gel electrophoresis to obtain fragments in the range of 200-300 bp. The size of the DNA was fractionated using different ratios of sample volumes and AMPure XP beads. The size between 150 and 300 bp was used for Illumina adapter ligation. Blunt-end repair, A-tailing and biotin pull-down were performed to get the biotin labeled Hi-C ligation products.

S1.4.3 Illumina adapter ligation, PCR amplification and sequencing.

Adapters were ligated to Hi-C DNA by T4 DNA ligase (Fermentas). The ligation products were purified. Test PCR reactions were performed to find the optimal PCR cycles to produce sufficient library for sequencing, without generating unwanted byproducts. Then, amplification of the DNA was carried out by PCR reactions in large-scale with the forward and reverse primer pair: 5`- AAT GAT ACG GCG ACC ACC GAG AT-3` and 5`-CAA GCA GAA GAC GGC ATA CGA -3`. In order to select the PCR cycles, size distribution and quantity of the PCR products were analyzed using the Agilent Bioanalyzer High Sensitivity DNA Kit. To estimate the fraction with biotinylated junctions, 100 ng of the library were digested with NheI. The Hi-C library was quantified using Real-Time PCR and sequenced (paired-end, 2 x 100 cycles) using the HiSeq X-ten Illumina system as described for the PE150. We have done three samples for Hi-C sequencing, producing 600 Gb raw data and developed valid paired-end reads of 31,734,151 covering over 99.6% of the total length of contig sequences.

S1.5 Scaffolding the PacBio assembly with the Hi-C information

The Hi-C sequence data was used to scaffold preliminary assemblies. First, the cleaned paired-end reads (in FASTQ format) generated by Illumina HiSeq from Hi-C library were aligned to the assemblies by HiC-Pro software[90] with default parameters, resulting in 39.05% unique mapped reads out of the total 645.77 Mb pairs of clean reads. After considering the map position and orientation of these unique reads, 14,782,854 pairs (5.86%) were filtered as validated pairs. Second, LACHESIS software[18], which uses hierarchical agglomerative clustering to group contigs that are likely to derive from the same chromosome according to the validated Hi-C dataset, grouped 4,329 out of 7,232 contigs into 20 groups with N50 of 135,691,394 bp and total length of 2,492,481,929 (98.19% of total assembled length). In each group, contigs were linearly ordered by making use of the higher Hi-C link densities expected between closely located contigs. Each group generated a graph with vertices representing contigs and edge weights corresponding to the inverse of the normalized Hi-C linkage density between pairs of contigs, and a weighted directed acyclic graph (WDAG) was built representing all possible ways to orient the contigs in each chromosome group, given the predicted order[18]. The process also contains an error-correction step, which broke 297 contigs into 631 contigs, and finally, 3,664 high-confident contigs were anchored to a total of 20 chromosomes accounting for 2,424,161,010 bp (95.5% of all assembly length) and leaving 3,902 contigs unanchored totaling 114,247,896 bp **(Table 1)**.

S1.6 Integrating genetic maps using ALLMAPS

S1.6.1 Method. Four high density genetic maps were utilized for integrated map construction including map R314 with 5,019 SNP markers, map YX267 with 6,523 SNP markers, map 1623 with 1,592 SNP markers[19], and map 1954 with 1,836 SSR markers (http://marker.kazusa.or.jp/Peanut/). Map R314 and YX267 are from the same RIL population derived from the same parents as shown in **Supplementary Note 6**. To obtain consistent orientations of the four maps, the marker sequences of linkage group in the map were BLASTed against the diploid A and B genome from *A. duranensis* and *A. ipaensis*, respectively[1]. Based on the markers' sequences and linkage positions, the four maps were integrated into one map using ALLMAPS[21] with 7,566 contigs assembled from Hi-C sequencing as the reference. The priority level followed the hierarchical order: R314>YX267>1623>1954. The resulting integrated map was manually investigated to check whether any marker that was unable to be integrated by ALLMAPS could be integrated based on a perfect match to a

contig from Hi-C assembly. This integrated map was used as a reference for whole genome assembly. For the final integrated map, the linkage map position was projected based on marker recombination frequencies, which were calculated in a sliding window of 3 Mb based on the original linkage map's genetic and physical locations on the final genome assembly. The length of each integrated linkage group was checked to make sure that it was greater than the longest length of the corresponding linkage group from four maps.

S1.6.2 Results. The integrated map contained a total of 14,619 markers covering 3,264 cM with 4,899 (97.6%), 6,369 (97.6%), 1,575 (98.9%), and 1,776 (96.7%) markers from the four linkage maps of 314, 267, 1623, and 1954, respectively **(Supplementary Data Set 2; Supplementary Data Set 3a-d)**. A total of 4,826 and 9,793 markers were integrated on the A and B genomes of the integrated map, respectively **(Supplementary Table 1)**. The mapped markers for each integrated linkage group were derived from the corresponding linkage group identified from their original map (**Supplementary Data Set 3b,** pink highlighted cells, and **Supplementary Fig. 2)**. However, notable amounts of markers in the integrated map also came from homeologous linkage groups in the original map (**Supplementary Data Set 3b** orange highlighted and **Supplementary Fig. 2)** or from other unrelated linkage groups specifically for the chromosomes in B genome (**Supplementary Fig. 2** red highlighted cells and un-highlighted cells). These 14,619 markers had matches with 2,312 of 7,566 assembled contigs from Hi-C sequencing results, with a total summed physical length of 2.2 Gb (86.68% of total length of assemblies). The size of the integrated contigs ranged from 2,904 bp to 8.5 Mb with an average of 0.96 Mb.

S1.7. Improving the Hi-C scaffolds with integrated genetic mapping

The construction of pseudomolecules followed an automated procedure by the integration of the following datasets: (1) sequence assemblies of 7232 contigs; (2) the high density integrated linkage map with 14619 markers as described above; (3) Hi-C data with 31,734,151 valid paired-end reads covering over 99.6% of the total length of contigs. Specifically, Hi-C data were used to map the contigs and cluster the contigs into scaffolds, producing twenty big scaffolds of over 49 Mb using the software LACHESIS[18]. Hi-C showed advantages in clustering contigs belonging to a chromosome into the same scaffolds and in contig orientation within scaffolds. Then, using the Hi-C assembled map and the integrated linkage maps, the whole chromosomes were assembled by ALLMAPS software[21] with a priority of Hi-C assembled map vs. integrated genetic map being 1:1. The Hi-C assembled chromosomal scaffolds were optimized for the arrangements and

orientation of contig trunks in this step, together with manual adjustment. Five error scaffolds assembled by Hi-C alone were found through ALLMAPS and rearranged by ALLMAPS together with manual correction. Subsequently, the pseudomolecules were generated by concatenating the adjacent contig sequences with 100 "N"s, and were oriented and numbered in accordance with previously published diploid genomes[1]. Finally, the adjacent anchored contigs were connected by 100 bp Ns, and the unanchored contigs were ordered according to size with the largest on the top and the smallest on the bottom, also using 100 bp Ns as connection between adjacent contigs, forming a pseudo chromosome named Chr00. The final genome size is 2,539,163,406 bp with N50 of 135,085,854 bp, and the anchored and unanchored contigs are 6,289 and 1,277, respectively **(Table 1; Supplementary Table 4)**.

S1.8. Validating the reference genome assembly

The assembly was first validated by comparing the Illumina short-reads described above (100 X coverage) to the reference assembly using SAMtools (version 1.6)[91]. Of the 803,264,808 Illumina short reads generated, 99.59% were successfully mapped back to the final assembly, with 97.44% properly paired.

To further validate the genome assembly, a total of 4,448 public bacterial artificial chromosome end sequences (BESs) of *A. hypogaea* in Genbank GSS database (accession number from FI498696.1 to FI503143.1) were downloaded for analysis. After removing 420 BACs with single-end sequences, 2014 pairs of BESs ((4448-420)/2=2014) were aligned to the reference genome through BLASTn and 1,987 (98.66%) pairs have significant matches, leaving only 20 with one end matching to the genome and 7 with no match. Of the 1987 BESs, 1,969 (99.1%) aligned to the same contigs with both ends. A total of 1576 BAC end paired sequences met the criterion: (1) > 95% alignment identity; (2) > 90% alignment coverage for BESs; (3) not located in pseudochromosome Chr00; (4) insert size lower than 200 Kb. The insertion lengths between matched pair of BAC end sequences within the genome are about 110 kb on average **(Supplementary Fig. 3a; Supplementary Data Set 4).**

Besides BESs, three public peanut full-length BACs (HF937574.1, HQ637178.1, and HQ637177.1) were also used to validate the genome assembly using BLAT software[92]. The best hit of BLAT results shows all three BACs are well in line with the genome assembly (**Supplementary Fig. 3b-c**).

We also estimated the whole ConsensusCallErrors in the genome with Illumina DNA short reads, the sample_4 data of Shitouqi (not used in the Pilon correction) together with other 4 data from four peanut

samples (**Supplementary Data Set 4b**). Both SNPs and INDELs were counted the homozygous variant calls and estimated the consensus error calls to be 46.3 per 1 Mb, which is much higher than the quality standard (99.99% accuracy) of human genome (Jeremy Schmutz, et.al, 2004, Nature) and rice genome (International Rice Genome Sequencing Project, 2005, Nature).

In addition, we mapped the PB reads to assembled contigs and found only 0.77% of contig length that were assembled into chromosomes but supported by less than 5 reads. For the total assembly, only 19.55 Mb were supported with less than 5 reads. That shows that our genome assembly is of high quality (**Supplementary Data Set 4c**).

Supplementary Note 2. Transcriptome sequencing and reference genome annotation

S2.1. Plant growth and RNA extraction

Peanut plants were grown in the greenhouse or the field to different growth stages and a total of 29 samples across tissues and different circumstances (as described follows) were prepared and kept in -80℃ via snap-freezing in liquid nitrogen. RNA was extracted from the following 29 samples (**Supplementary Data Set 21**): roots (including main root of germinating seed for 3 days, and roots of plants with 4, 8 and 12 leaves), root tips (from 4 day seedlings); root-stem (from 7 day seedlings and plants with 4 and 8 leaves), root nodules (from plants with 8 and 12 leaves), leaves (from plants with two leaves, 8 leaf plants, 12 leaf plants of different leaf growth stages), cotyledons (from germinating seeds after 3 days, germinate and plants with 4 and 8 leaves), stem tips (from seedlings/plants of 2, 4, and 8 leaves), inflorescence (the whole inflorescence including flower buds of different stages and opening flowers), pegs (from 12-leaf plants including different stages of peg), pericarps (from plants of pod development stage and mature stages including 10, 30 and 50 days after pegging), testa (from plants of pod development and mature stages, including two stages of 20 and 40 days after pegging), embryos (from plants of pod development and mature stages, including four stages of 10, 20, 30 and 50 days after pegging). All the above samples were taken from the plants or geminating seeds planted on the field at a temperature of 20-30℃ under natural length of light cycles with well humid condition. RNA was also isolated from leaves of seedlings with drought or low temperature treatments at different time points and their control plants, and from leaves of seedlings treated with five hormones by

spraying [salicylic acid (3 mM), ethylene (10 mM), abscisic acid (10 μg/mL), brassinosteroid (0.1 mg/L), paclobutrazol (150 mg/L)] at different time points and their control plants. Plants of drought treatments are sampled at 2, 4, and 8 days after treatments, those of cold and five hormones treatments at 3, 6, 12, and 24 hours after treatments. For all the treatments, plants were grown in well-watered conditions excepting drought treatments, at a temperature of 28 ℃, in a light cycle of 14 hours of light (200 mmol $s^{-1}m^{-2}$) and 10 hours of darkness in a green house. RNA was extracted from the isolated samples individually and then equally mixed to form the 29 RNA samples. Frozen tissue from all samples was ground using mortar and pestle, and RNA was isolated using the TIANGEN RNAprep Pure Kit (for Polysaccharide and Polyphenolic-rich plants) (Cat. no. DP441). RNA quality was assessed using an Agilent 2100 BioAnalyzer. In addition, 14 other RNA sequence data released by us were also integrated to aid genome annotation.

S2.2 Transcriptome sequencing

Sequencing libraries were prepared using the Illumina TruSeq RNA Sample Prep Kit. 150-bp PE sequencing was performed using an Illumina HiSeq X Ten machine at WuXi NextCODE (Shanghai, China). Raw data were preprocessed through the NGS QC Toolkit (v2.3.3)[93] to remove adaptors, low quality bases, and reads containing more than 10% unknown bases ("N"). The remaining reads were then evaluated using FastQC (http://www.bioinformatics.babraham.ac.uk/projects/fastqc/). Finally, the clean reads were employed for the genome protein-coding gene prediction and verification of the prediction results (**Supplementary Data Set 5b**), which supported the gene model annotions with accuracy.

Supplementary Note Table 2.2 Summary of clean data for 29 tissue samples.

Sample	Read Length (bp)	Clean reads	Clean Bases (bp)	Q30 Rate
10PC	150	51,471,632	7,501,202,492	0.92
10PE	150	52,426,204	7,627,699,947	0.93
20PE	150	49,749,208	7,232,930,202	0.94
20PT	150	55,393,532	8,081,073,567	0.93
30PC	150	59,653,256	8,714,709,335	0.93
30PE	150	55,999,512	8,063,560,969	0.88
40PT	150	55,984,122	8,162,428,252	0.92
50PC	150	52,824,026	7,705,352,070	0.93
50PE	150	44,547,974	6,424,291,952	0.88
ABA	150	56,158,908	8,200,644,936	0.92
BR	150	54,420,908	7,900,835,966	0.91
Cot	150	46,142,872	6,690,016,746	0.93

Dry	150	49,668,370	7,245,813,100	0.92
DryCK	150	54,167,142	7,925,477,607	0.93
ETH	150	48,271,300	7,061,736,048	0.91
F	150	60,549,898	8,753,327,090	0.94
HorCK	150	46,392,810	6,774,176,798	0.91
Leaf	150	56,427,020	8,214,021,033	0.93
LT	150	56,518,092	8,192,470,047	0.92
LTCK	150	60,934,146	8,876,574,417	0.92
PAC	150	42,028,158	6,018,784,289	0.88
Peg	150	53,699,150	7,787,703,102	0.92
Rnod1	150	57,022,988	8,279,521,494	0.92
Root	150	61,466,640	8,940,488,791	0.93
RS	150	49,488,042	7,157,421,555	0.92
Rtip1	150	60,296,952	8,737,698,739	0.92
SA	150	52,750,796	7,612,265,798	0.89
Stem	150	56,603,108	8,217,581,159	0.93
Stip	150	58,328,826	8,476,499,895	0.93

S2.3 PacBio Isoseq data generation and correction

S2.3.1 Material and method. The above 29 RNA samples were mixed equally and the quality of mixed RNA was assessed with standard agarose gel electrophoresis, Thermo Fisher Scientific Qubit Fluorometer, and Agilent 2100 BioAnalyzer. RNA was fractionated into three libraries consisting of differently sized RNA (1-2 kb, 2-3 kb, and 3-6 kb). A total of five SMRT cells for each RNA sample were run on the PacBio Sequel system with the Sequel DNA Polymerase 2.0 and Sequel Sequencing Plate 2.0 by NextOmics Biosciences Co., Ltd (Wuhan, China). Sequencing reads were processed with IsoSeq_SA3nUP pipeline in SMRT Link (v4.0)[94] using the following steps:

S2.3.2 Isoseq reads preprocess. A total of 1,172,600 reads covering 21,207,188,734 bp were produced by the Sequel system for the three RNA libraries and the adaptor and low-quality sequences were automatically removed in the internal process of the sequencer. These polymerase reads are composed of one or more subreads, which represent the sequenced single molecular length. Different subreads from the same polymerase read generate circular consensus sequences (CCS). After using the "ccs" command in the IsoSeq_SA3nUP pipeline, only the polymerase reads with at least one subread were considered, and these processes left 813,825 CCSs (1,890,380,326 bp).

S2.3.3 Isoseq classification, cluster, and correction. The CCSs were then classified as full-length,

non-chimeric and non-full-length by identifying and using 5'-primer, 3'-primer, and poly A/T tails. These three parts were removed in the final output. For the total of 813,825 CSSs, 83.17% are full-length and non-chimeric. These sequences were then clustered through an Iterative Clustering and Error correction (ICE) algorithm incorporated in the IsoSeq_SA3nUP pipeline. The input and output file of ICE is the full-length, non-chimeric reads, and the unpolished consensus isoforms, respectively. These consensus isoforms were further polished by using the non-full-length reads and the raw bam file with --quiver parameter. These processes generated 356,891 non-redundant, non-chimeric CCSs, comprising a total of 784,294,264 bp. These sequences were finally polished with Illumina RNA-seq data from the combined 29 tissues through LoRDEC (v0.3) software[95].

S2.3.4 Filtration, fusion gene and redundancy. A fusion gene is a hybrid gene formed from two previously separate genes. We used "fusion_finder.py" (https://github.com/Magdoll/cDNA_Cupcake/wiki) to detect fusion genes in the polished isoseq. Reads were firstly aligned to the peanut genome using GMAP[96], and according to the alignment results, isoseqs were considered fusion genes if satisfying the following criterions: (1) two or more genome regions were aligned by one isoseq read; (2) the coverage of each aligned site for one isoseq is at least 10; (3) at least 99% region of the isoseq was covered; and (4) the whole alignment length is at least 10 kb. Finally, 11,474 fusion genes were found, which contained 12,260 isoseq reads and 344,631 corrected isoforms. These isoforms are still redundant: for example, there are more than one read aligned to the same position of the genome, so these redundant sequences were further filtered by using the script "collapse_isoforms_by_sam.py" in the PacBio SMRT-link package, with parameters "--min-identity 0.9 and --min-coverage 0.85", and after filtration, 120,685 isoforms remain, comprising 273,023,489 bp. These isoforms were used for the genome protein-coding gene annotation in the following analyses.

S2.4 Ab initio gene model prediction

AUGUSTUS[60], SNAP[97], and GeneMark[98] were employed as the main *ab initio* prediction softwares for the genomes. Coding sequences from *A. duranensis and A. ipaensis* were concatenated to create a master list of genes. Fifty percent of the genes from the master list with high scores were used to train the AUGUSTUS model, and the remaining genes were used for validation. Two rounds of prediction optimization were performed with the software package provided by AUGUSTUS. Next, RNA-Seq clean reads from 29 different tissues and from 14 integrated samples with biotic stresses, were mapped onto the reference genome using

HISAT2[99] and assembled into transcripts using StringTie[100] and Cuffmerge[101] to determine locations of potential intron-exon boundaries. These transcripts, together with full-length transcripts generated from Iso-Seq using the mixture of 29 RNA samples, and Tifrunner transcripts downloaded from PeanutBase (https://peanutbase.org/files/genomes/Arachis_hypogaea/transcriptomes/Arahy.Tifrunner.sSQ6/) were used as the input files of the MAKER software package[102]. Thirdly, a set of homologous proteins from other species were used for homology evidence. The species include: *A. duranesis* (V14167; PeanutBase), *A. ipaensis* (K30076; PeanutBase), *G. max* (Wm82.a2.v； Phytozome12), *M. truncatula* (Mt4.0v1; Phytozome12), and *P. vulgaris* (v2.1; Phytozome 12). Finally, MAKER was used with AUGUSTUS model trained above to predict genes in the repeat-masked reference genome with default parameters except alt_splice=0 and always_complete=1. To assess the quality of gene prediction, the best supported gene models were chosen using the AED (Annotation Edit Distance), and only genes with AED<=0.75 were left as the final predicted results **(Supplementary Fig. 4d)**. The final set contains a total of 83,709 genes. The average gene length is 5,076 bp with an average coding sequence length of 1,589.77bp **(Supplementary Table 2a, b)**. Alignments using the RNA-seq clean reads from 29 different tissues supported 64,513 genes, accounting for 76.64% of the total genes **(Supplementary Table 2c)**. Functional assignments of the peanut predicted genes were made with BLAST (version 2.2.28+) by aligning their protein-coding regions to sequences in public protein databases, including KEGG (81.0)[103], SwissProt (release 2017_03)[104], and TrEMBL (release 2017_03). Secondary annotation or domain searches were based on the InterProScan (5.21-60.0)[105] databases. A total of 64,179 genes were functionally annotated, comprising 76.67% of the 83,709 gene models **(Supplementary Table 2d)**.

S2.5 Characterization of repetitive sequences

Repeat families in the genome assemblies of *A. hypogaea* were first *de novo* independently identified and classified using the software RepeatModeler (https://github.com/rmhubley/RepeatModeler) (version 1.0.11). Following the classification process, the output data files from RepeatModeler for the genome assemblies were used as a custom repeat library by the RepeatMasker program (http://www.repeatmasker.org/) (version 4.0.7) to discover and identify repeats within the peanut genome. To make comparisons, the published two diploid ancestors, *A. duranensis* and *A. ipaensis*[1], were also used to identify repetitive elements using the same method. The results of repeat annotation are summarized in **Supplementary Table 3b**. The peanut genome

contains a total of 77.65% repetitive elements.

S2.6 Validation of gene models with BUSCO

Genome assembly and annotation completeness were assessed using the 1,440 conserved genes of embryonic plants in BUSCO[25] with the BLAST E-value cutoff set to 1e10-5 **(Supplementary Table 5).** The total results for sequencing, assembly and annotation were summarized in **Supplementary Table 4.**

S2.7 Non-coding RNAs prediction

Non-coding RNAs, including miRNA, snRNA, and rRNA, were identified by using INFERNAL[106] to search the Rfam database. RNAmmer was additionally used to identity rRNA in more detailed subclasses. The tRNA genes were searched by tRNAscan-SE[107]. In total, we identified 480 microRNAs, 4723 transfer RNAs, 2,808 ribosomal RNAs and 30,817 small nuclear RNAs in the peanut genome (**Supplementary Table 3a**).

S2.8 Analysis of homeologs in an allotetraploid peanut genome

S2.8.1 Methods. Non-redundant genes in the cultivated peanut genome were identified based on BLAST of protein-coding genes between the two subgenomes. Briefly, protein sequences extracted from A sub-genome were BLASTed against proteins from B sub-genome, and vice versa. The best matches were retained and formatted as a two-column table of homeolog-pairs. Duplicated genes were identified by aligning genes that are absent on the table against homeolog-pairs and classified as tandem duplicated or dispersed duplicated genes if there is significant similarity with other genes present on the table. The remaining genes were classified as genes without homeologs.

S2.8.2 Identification of homeologous genes. Detailed annotation of homeologous genes in allopolyploid genomes will improve the accuracy of non-redundant gene numbers in the genome and enhance the precision of gene expression analysis. Comparative analysis of the two subgenomes identified a total of 30,596 non-redundant genes, including 24,208 (79.12%) with and 6,388 (20.88%) without homeologs **(Supplementary Table 6)**. We further investigated whether the genes without homeologs have orthologs in the ancestral diploid genomes. Among the 6,388 genes, 2174 (34.03%) and 2814 (44.05%) have orthologs in subgenomes A and B, respectively, and 1,400 are unique in allotetraploid peanut. Among the 1,400 unique genes, 244 have homologs in GenBank, suggesting that their orthologs in ancestral diploid genomes are either mis-annotated or lost. The remaining 1,156 non-homeologous genes may be either mis-annotated or of de

novo origin and species specific.

Sequence identity of homeologous genes within the tetraploid genome and orthologous genes between the cultivated peanut genome and its ancestral diploid genomes was determined based on reciprocal BLAST. Within the tetraploid genome, the sequence identity between homeologous genes of the two subgenomes ranged from 40.25% to 100%, with an average of 86.72%. The sequence identity of orthologous genes between subgenome A and its putative ancestral diploid genome ranged from 40.05% to 100% with an average at 85.32%. The sequence identity of orthologous genes between subgenome B and its putative ancestral diploid genome ranged from 40.09% to 100% with an average at 85.64%. Overall, the sequence identity of 76118 ortholog pairs ranged from 40.05% to 100% with an average of 85.51% and a vast majority (70.36%) of ortholog-pairs having >80% sequence identity (**Supplementary Note Fig. S2.8.2**). Noting that peanut shares several earlier genome duplications with other eudicots, some of the lowest sequence identities could represent mis-classification of homoeologs as recent when they actually trace to earlier polyploidizations.

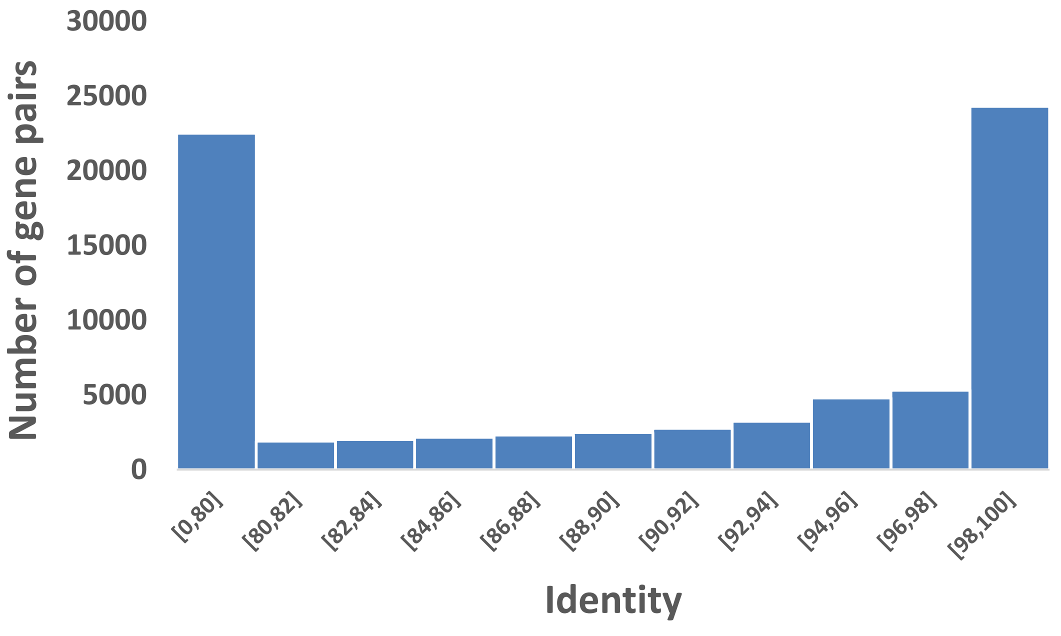

Supplementary Note Figure 2.8.2. The distribution of sequence identity of orthologs between cultivated peanuts and its ancestors.

S2.8.3 Identification of non-redundant genes. In addition, we also detected a large number of duplicated genes (27,913), including 25,511 dispersed duplicated genes and 2,402 tandem duplicated genes

(Supplementary Table 6). It is no surprise that there are as many as ~28 k duplicated genes in the allotetraploid peanut genome. The ancestral diploid genomes contain 36,734 genes in *A. duranensis* and 41,840 genes in *A. ipaensis*. As stated in previous research, the elevated gene numbers in A. ipaensis arisen from locale duplications[1]. In the tetraploid peanut genome, a total of 83,709 genes are annotated. This number is more than the sum of the two diploid genomes (78,574), suggesting there are many species-specific duplicated genes in the tetraploid genome. Among the 27,913 duplicated genes, 10,590 are in subgenome A and 17,323 are in subgenome B. Meanwhile, 25,165 genes (90.15%) have orthologs in the two diploid ancestral genomes.

S2.8.4 Characterization of expression of homeolog pairs. Global expression patterns of these homeolog-pairs revealed prevalent differential expression between the two subgenomes (**Supplementary Table 6; Supplementary Data Set 5c; Fig.1**). We compared the expression of 24,208 homeolog-pairs from 29 RNA samples. A total of 3,638 genes have dominant effects in all the tested samples. In addition, expression of 11,832 genes are higher than their counterparts in at least 2/3 investigated samples, showing a large proportion (48.88%) of homeolog-pairs to have partial dominance effects.

Supplementary Note 3. Comparative genomic analysis

S3.1 Methods

S3.1.1 Inferring gene collinearity. With annotated genes as input, chromosomes from within a genome or between different genomes were compared. Firstly, by performing BLASTp[87], protein sequences were searched against one another to find potentially homologous genes (E-value < 1e10-5). A smaller E-value may involve more-diverged homologous genes and help find ancient duplicated genes. Secondly, information about gene homology was used as input for the software ColinearScan[108] to locate homologous gene pairs in colinearity. The key parameter, the maximum gap, was set to be 50 intervening genes, as adopted in previous genomics research[27]. Large gene families with 30 or more copies in a genome were removed from inferring colinearity to avoid false positive associations.

S3.1.2 Inferring genomic homology. To infer chromosomal homology in legumes, we used the grape genome as an outgroup reference, which provided chromosome homology information transitively. The grape genome preserves the ancestral genome structure before and after the ECH that was common to most eudicot

plants[29,109] much better than other sequenced eudicot genomes, which are often affected by further polyploidization(s). The grape genome was important to reveal and distinguish paralogous blocks within legume genomes that were produced by the ECH event or otherwise. Due to the ECH, any one grape genomic region often has 2 paralogous regions within grape itself, and more in legume genomes. Dot plots of homology between genomes produced by our custom software were used to help distinguish orthologous and outparalogous regions between different genomes. We produced dot plots between each pair of grape and legumes.

S3.2 Genome comparison between the A and B sub-genome of *A. hypogaea*

By performing BLASTp to visualize the chromosomal locations of all homeologous gene pairs, we found 35576 colinear genes in 3539 blocks of ≥ 4 gene pairs preserved in A and B subgenomes with 63 homeologous blocks covering 13583 gene pairs **(Fig. 2a; Supplementary Table 7)**, indicating high colinearity between the two subgenomes. However, this may allow intra-subgenome recombination,[26] as a total of 2067 gene pairs of 301 blocks > 4 homeologous gene pairs were found in colinearity in the A subgenome and 2283 gene pairs of 300 blocks in B subgenome **(Supplementary Table 7)**. We also found evidence of gene conversion for 629 genes (1.8% of 35576), with 369 (58.7%) A subgenome genes being converted by their B subgenome counterparts and 230 (41.3%) vice versa (Chi-square test P-value = 1.4 x 10^{-8}). These were supported by prominent recombinations **(Fig. 2a; Supplementary Fig. 6; Supplementary Fig. 10)** and by translocations and substitutions as shown by BLASTn in each A and B subgenomes using contig sequences from corresponding chromosomes of diploid A and B genome separately **(Supplementary Data Set 6)**. Furthermore, except for chromosome pairs of 2 versus 12 and 8 versus 18, homeologous chromosome pairs between A and B sub-genomes all have one to several inversions, as demonstrated by syntenic block analysis of over 5 gene pairs **(Supplementary Fig. 5a)**. By comparison of density distribution patterns of both genes and abundant repeat sequences, we found that the A sub-genome experienced significant change but the B sub-genome remained more stable **(Supplementary Fig. 5b)**. In partial summary, the peanut genome underwent both intra- and inter-subgenomic chromosome recombinations and rearrangements with the Bt subgenome being dominant in preserving ancestral information.

S3.3 Genome comparison of *A. hypogaea* with diploid *A. duranensis* and *A. ipaensis*

S3.3.1 Assignment of contigs to A and B subgenomes

Using splited 10-Kb window of each contigs of peanut genome, we run BLAST against the two ancestry genomes (A merge version of AA and BB genomes), filtered the 10-Kb parts with extreme GC content (lower than 20% or greater than 80%). 5,702 contigs (2,519,836,999 bp, 99.27% of the total contig length) aligned to the two diploids **(Supplementary Data Set 6)**. Contigs were assigned to the A or B sub-genome if >70% were covered by one diploid and <25% by the other diploid. Then, 2477 (1,085,725,279 bp) and 2543 (1,408,447,132 bp) contigs were assigned to A and B subgenomes with the identity of > 92.82% and > 93.28%, and taking 42.77% and 55.49% of total contig length, respectively. The BB genome contributed 322721853 bp (29.72%) more than the AA genome **(Supplementary Data Set 6)**. These indicate that B subgenome was more similar to *A. ipaensis*[1] than A subgenome to *A. duranensis*.

S3.3.2 Preservation of homologous inheritance information. Between the diploid A genome and tetraploid A subgenome, we revealed 2310 orthologous blocks with >= 4 colinear genes, in total containing 34266 colinear genes, while between the B genome and B subgenome, more blocks (2405) and more genes (38417) were inferred **(Supplementary Table 7)**. For blocks with >= 50 colinear genes, the B genome and B subgenome has ~2800 more colinear genes in total. The longest B and sub-B genome block is from their chromosome 7, containing 1553 colinear genes, as compared to the longest A and subA genome block in their chromosome 3 with 1058 colinear genes. These findings show that the B subgenome is more similar to the sequenced B genome diploid, than the A subgenome is to the A genome diploid.

S3.3.3 Tetraploidization enhanced peanut chromosome changes. Reciprocal syntenic comparisons of subgenomes with diploid A and B genomes, helped to find dozens of large inversions and crossing-over regions **(Supplementary Figs. 6, 8)**, For inversions >= 3 Mb, we identified 11 A and B subgenome incongruent chromosomal regions, 11 A and A subgenome regions, and 4 B and B subgenome regions, resulting from 23 independent events. The majority of these events (20 of 23, or 87%) occurred during the divergence of A and A subgenome lineages, consistent with either the A diploid not being the progenitor, or with more rapid evolution of the A subgenome after tetraploidization. We found at least 6 chromosomal exchanges or substitutions between A and B subgenomes and there was a 10 Mb translocation between chromosome 3 and 13, respectively **(Supplementary Data Set 7; Fig. 2c)**.

S3.3.4 Comparison of contigs from diploid A and B genomes to the tetraploid A and B subgenomes. The

published diploid A and B genomes[1] were divided into contigs at every gap with 100 Ns, which were aligned to the corresponding A and B chromosomes in tetraploid peanut with parameters "-a 8 -p blastn -m 9 -e 1e-10". The best hit with alignment length >= 2,000 bp were selected to be plotted in the dot plots.

The majority of the ancestral diploid A and B genomes matched their corresponding tetraploid genomes with more than 99.5% sequence identity, particularly the diploid B genome and the corresponding B genome in the tetraploid. The A genome in *A. duranensis* was much more diverged from the A subgenome in *A. hypogaea* than the B genome in *A. ipaensis* from the B subgenome in *A. hypogaea*. For example, chromosome A08 of *A. duranensis* was significantly diverged from A08 of *A. hypogaea*, while chromosome B08 of *A ipaensis* was nearly identical to the A08 of *A. hypogaea*. The A and B genome sequences were relatively similar at the ends of the chromosomes, with greater divergence in the middle part of the chromosomes. Particularly at the end of chromosomes B02, A03, B03, B04, B06, and A07, significant stretches of A and B genome swaps were identified, which suggested homeologous chromosomes interacting, pairing, and exchanging during meiosis after polyploidization specifically at the chromosome ends.

S3.3.5 Comparison of repeat element between peanut and its progenitors. TE expansions on the shape of the polyploid genome evolution were studied. Most transposable elements were found to have experienced expansion after tetraploidization, especially the Gypsy and unclassified elements of LTRs expanded for 154.2 Mb and 192.8 Mb **(Supplementary Table 3b)**. We used the LTR_FINDER results, and by employing the PAML software to calculate the substitution rate between the pair end sequences of LTR-retrotransposons. The main peak of substitution rates for *A. hypogaea*, *A. hypogaea-subA, A. hypogaea-subB, A. duranensis*, and *A .ipaensis* are 0.0081, 0.0081, 0.0293, 0.0368, and 0.0330, respectively. These indicate a much more recent LTR expansion in *A. hypogaea*, which is mainly caused by Sub-A genome. Relatively, the LTR expansions of Sub-B genome is much moderately in recent evolution compared to Sub-A genome, and is comparable to the LTR expansions of *A. duranensis* and *A. ipaensis* genome in time which is earlier than Sub-A. If we used a Ks value of **8.21×10^{-9}** Ks/year and usually, the evolution rate is two times of repeat regions as the coding region[125], so the evolution rate is 1.64×10^{-8} ks/year in repeat region. Using a formula $T=S/2\mu$[126], where T is the evolution time, S is the substitution rate here, and μ is the substitution rate, the LTR expansion time is 0.2467, 0.2467, 0.8922, 1.1206, and 1.0049 million years, respectively.

S3.4 Genomic comparison of *A. hypogaea* with other legume species

S3.4.1 Syntenic comparison. Peanut shares a whole-genome duplication ~59 million years ago (MYA) with other legumes, and a whole-genome triplication ~130 MYA with other core eudicots[27,29,109]. By gene colinearity analysis, we found the legume genome duplication has 1289 and 1508 colinear genes in A and B subgenomes, accounting for 6.9% and 6.6% of their respective gene content. The eudicot genome triplication preserved 1198 and 1372 colinear genes in A and B subgenomes, accounting for 6.5% and 6.1% of their respective gene content. This indicates traces of ancient polyploidization remaining in the extant subgenomes (**Fig. 2d; Supplementary Figs. 7, 9; Supplementary Data Set 8**).

S3.4.2 Multiple genome alignment. With the *M. truncatula* (barrel medic) genome as the reference, the other 11 legume genomes were aligned by using colinear genes as anchor points. The alignment is displayed in a circular manner, with each genome shown in patches mapped onto the barrel medic chromosomes. Due to lineage-specific tetraploidy (SST), the soybean genome is displayed in two neighboring circles **(Fig. 2d)**. Genes met by a half-line starting from the center of the circle are homologous, being orthologs (if the best hit), outparalogs (if a secondary hit between genomes) or paralogs if within a genome. Furthermore, to show genome structure changes after the ECH, with the grape genome as the reference, 12 legume genomes were aligned. To reflect the LCT and the SST, the circular alignment has 1 + 11x2 + 4 = 27 circles of homologous chromosomal patches **(Fig. 2d)**. We found peanut genome generally preserved more primitive status than other legumes.

S3.5 Reconstruction of *A. hypogaea* chromosomes from an inferred legume common ancestor

S3.5.1 Method. By comparing relative genomes, ancestral karyotypes were inferred by inferring merging events, crossing-overs, and commonly-preserved chromosomes or chromosomal regions. We deduced karyotypes and their changes from the eudicot common ancestor to extant legumes by a bi-directional strategy: top-down and bottom-up.

S3.5.2 Inferring ancestral legume basic chromosomes top-down. We took a top-down approach to infer the ancestral legume karyotype before the legume-common whole-genome duplication. There is thought to have been 7 ancestral haploid chromosomes in the common ancestor of core eudicots before the whole-genome triplication **(Fig. 3).** After the event, the chromosome number tripled to 21, and the corresponding karyotype has been largely preserved in the grape genome. By comparing the grape genome to legume genomes using

homologous gene dotplots **(Supplementary Fig. 9a,b),** , we inferred ancestral chromosome fusions during evolution. A fusion of two chromosomes could be identified by the repetitive co-occurrence of their broken or duplicated segments in different extant legume chromosomes. For example, two ancestral chromosomes, corresponding to grape chromosomes 6 and 13, were inferred to have fused before the legume-common whole-genome duplication, in that their respective chromosomal segments were found to co-occur four times in peanut and other legume genomes. In total, 5 independent chromosome fusions, including 3 nested chromosome fusions (NCF) and 2 chromosome end-end joining (CEJ) fusions, were identified and therefore the karyotype was inferred to have 16 ancestral chromosomes, which were duplicated by the LCT **(Fig. 3; Supplementary Fig. 9c)**. Theoretically, during the process, 5 satellite or B chromosomes might have been produced and their loss resulted in chromosome number reduction[110]. The basic framework gene content of the 16 ancestral chromosomes was reconstructed by using corresponding common bean genes.

S3.5.3 Inferring ancestral legume common chromosomes bottom-up. We also took a bottom-up approach to infer the ancestral karyotype before the split of the legumes under consideration. With common bean (*Phaseolus vulgaris*, n=11) as the reference, the genomes of diploid peanut (*A. duranensis,* n=10), barrel medic (*Medicago truncatula*, n=8), soybean (*Glycine max*, n=20), pigeonpea (*Cajanus cajan*, n=11), and Adzuki bean (*Vigna angularis*, n=11) were compared to find independent chromosomes and chromosomal blocks. The integrity of each of 5 common bean chromosomes (Pv4, Pv5, Pv7, Pv10, and Pv11) is largely preserved in peanut and other legumes, showing their ancestral nature **(Supplementary Fig. 9d; Supplementary Data Set 8a-e; Fig. 3)**. Another 11 chromosomal blocks were also identified, which independently and repetitively appeared in inter-genomic dot plots, suggesting their ancestral integrity. Eventually, 16 ancestral chromosomes were identified. The above findings mean that before the LCT, there had been 16 chromosomes, which duplicated in the event, and then were reduced to 16 chromosomes again before the split of legumes.

S3.5.4. Reconstruction of peanut chromosomes. The 16 ancestral legume chromosomes after the LCT (called *Lu*), referred to by alphabetical letters, were reconstructed by using corresponding common bean genes, and then were compared to extant legume genomes. By using dot plots between *Lu* and each legume genome, and between close legume relatives, we inferred shared chromosomes and changes along each lineage **(Fig. 3; Supplementary Fig. 9d, e; Supplementary Data Set 8f-k)**. Along the peanut lineage, the 16 chromosomes reduced to 10 by 6 chromosome fusions, accompanied by 2 cross-overs, and another cross-over in the peanut B genome to produce its specific chromosomes 7 and 8, as shown above **(Supplementary Fig. 9d,e)**. The

peanut karyotype formed largely independently of those of other legume lineages. Indeed, the peanut and Hologalegina lineages (*L. japonicus* and barrel medic) share only one cross-over event (*Lu*: FxO). The 16 *Lu* chromosomes reduced to n=6 in *L. japonicus*, and n=8 in barrel medic; to 15 in the common ancestor of common bean and *Vigna* legumes (1 CEJ); to 13 in *Vigna* ancestor (2 CEJs), and 11 in each *Vigna* species but with different fusions; to 11 in common bean (4 CEJs); to 13 in the Glycine genus (3 CEJs) then duplicated to 26, further reducing to 20 in soybean.

Supplementary Note 4. Gene family analysis

S4.1 Clustering of gene families by OrthoMCL

S4.1.1 Detection of gene families from peanut genome using OrthoMCL

The following species: *A. duranensis* (V14167; PeanutBase), *A. ipaensis* (K30076; PeanutBase), *Glycine max* (Wm82.a2.v1； Phytozome12), *Medicago truncatula* (Mt4.0v1; Phytozome12), *Phaseolus vulgaris* (v2.1; Phytozome 12) and *Arabidopsis thaliana* (TAIR10; Phytozome 12) were used to lead the gene family analyses. Protein coding genes were compared against each other in an all-to-all way by using BLASTp software[87]. The OrthoMCL program[80] was employed to cluster the alignment results into family groups with default parameters. In a reciprocal alignment, to make the domain search more accurate, only the result that the overlap length is greater than 50% of any two sequences were considered. For comparison between peanut and its wild ancestral species, the parameter was set as: E-value 1e10-10 and alignment identity 30%; and comparison among peanut with other legume species as the above; and for comparison with Arabidopsis, the parameter was set to E-value 1e10-5 and alignment identity 30%.

S4.1.2 Gene family comparison of *A. hypogaea, A. duranensis,* and *A. ipaensis*

Gene content comparison. Compared to their diploid A and B genomes with 36,734 and 41,480 genes, both tetraploid A and B subgenomes show respectively 0.88% and 12.46% expansion in their gene content (contain 37,059 and 46,650 protein-coding genes). The B subgenome has played a dominant role in gene expansion, implying active gene duplications or recruitment after tetraploidization.

Gene family comparison. Analysis of two ancestral wild diploid species, *A. duranensis* and *A. ipaensis,* and tetraploid peanut using OrthoMCL showed that among 83,709 protein-coding genes predicted for *A. hypogaea*, 62,794 were clustered in a total of 25,153 families **(Supplementary Table 8)**. Of the three genomes, a total of

27,424 orthologous families were identified with 22,109 (91.72%) retained in the cultivated peanut genomes after tetraploidization, including 13,928 retained from both ancestors' genomes, 3,217 retained specifically from *A. duranensis*, and 4,964 from *A. ipaensis* **(Fig. 4a, Supplementary Data Set 9)**. The number of species-specific gene families for *A. hypogaea*, *A. duranensis*, *A. ipaensis* were 3,044, 332 and 413, which contained 10,339, 898 and 1,068 genes, respectively **(Supplementary Table 8)**.

Of the gene models in the genomes of cultivated peanut and its ancestors, *A. duranensis* and *A. ipaensis*, totals of 14,648 16,705 and 19,242 genes were identified as single copy, respectively **(Figure 4b; Supplementary Data Set 9)**. Among those single copy genes, 7,714 remained as single copy in both sub-genomes of cultivated peanut **(Fig. 4b)**, thus can be considered as a core set of single copy genes retained from ancestors. However, 1,162 and 939 single copy genes were lost from the cultivated peanut A and B sub-genomes, respectively, for a total of 2,101 single copy genes lost.

S4.1.3 Gene family comparison of *A. hypogaea, G. max, M. truncatula, P. vulgaris* and *A. thaliana.*

The comparisons were further enlarged to other related legumes (soybean, common bean, *M. truncatula*) and also to *A. thaliana*. Among the 83,709 protein-coding genes predicted for peanut, 72,036 were clustered in a total of 17,986 families **(Supplemental Table 8)**. A total of 9,614 gene families (36.70% of the total) were shared among these five species **(Supplementary Fig. 11)**. The number of species-specific gene families for *A. hypogaea*, *A. thaliana*, *G. max, P. vulgaris,* and *M. truncatula* are 5054, 929, 730, 181 and 1866 respectively **(Supplementary table 9)**. These again showed the peanut species specialization from other legume species, perhaps related to being a geocarpic plant. In comparison with two diploids, peanut A and B subgenomes shared 11646 orthologous groups with the two species and contained respective 340 and 780 specific families **(Supplementary Fig. 11b)**, indicating speciation of cultivated peanut diverged from the two wild ancestors.

S4.2 Analysis of the R-gene super family

To identify NBS encoding genes, predicted ORFs from each genome were screened using the raw Hidden Markov Model (HMM) HMMER3.0 to search for the Pfam NBS (NB-ARC) family PF00931 domain (E-value < 1e10-5). Of all the gene models annotated in the genomes, a total of 661 genes contained NBS domains in cultivated peanut **(Supplementary Data Set 10)**, versus 385 and 428 in the genomes of *A. duranensis* and *A. ipaensis*, respectively. These genes with the NBS domain mainly localized in the terminal

regions of chromosomes with more genes on chr02 and chr12 **(Fig. 4d)**, and they could be further divided into three categories **(Supplementary Fig. 12a)** including CNL (CC-NBS-LRR) as the largest category, TNL (TIR-NBS-LRR) as the second largest category, and RNL (RPW8-NBS-LRR) as the smallest category. Among the CNLs, the numbers of genes in wild species were much higher than in cultivated peanut due to gene loss in cultivated peanut **(Supplementary Fig. 12b)**. Of the TNL category, the gene numbers in wild ancestors' genomes were comparable to those in cultivated peanut, indicating the adaptability of TNL to cultivated peanut. Although the difference in numbers was minimal, each branch was not a one-to-one correspondence between wild and cultivated peanuts, indicating that the resistance genes were differentiated, with loss/gain between the cultivated and wild species.

S4.3 Analysis of Acyl metabolic related genes

S4.3.1 Methods. Two acyl-lipid gene datasets were utilized to explore orthologous genes in cultivated peanut: (1) ARABIDOPSIS ACYL-LIPID METABOLISM database (http://aralip.plantbiology.msu.edu/) that included 885 *Arabidopsis thaliana* acyl-lipid genes; and (2) Soybean genetics and genomics database (SoyBase, https://www.soybase.org/), which contained 829 soybean (*Glycine max*) acyl-lipid genes. The protein sequences of all the annotated gene models from genomes of the three *Arachis* species, together with major oilseed crops of *Glycine max*, *Elaeis guineensis* and *Brassica napus*, were aligned to both acyl-lipid gene datasets by BLASTp **(Supplementary Table 9)**. The hits with an E value < 10^{-6} and the percentage of matching length ≥30% for both genes were adopted, where the percentage of matching length = (alignment length × percentage of identity) / gene length. In addition, oil-related quantitative trait loci (QTLs) discovered in peanut were searched and summarized[36, 82, 111] to find peanut orthologs that are located within those QTL regions. The sequences of flanking markers of those QTLs were aligned to the cultivated peanut genome by using either Bowtie (-f -a -v 2 -I 80 -X 2000)[112] or BLASTn.

To identify key acyl-lipid genes during peanut embryo growth, the orthologous acyl-lipid genes in peanut were associated with RNA-seq data of four embryo growth time points (10PE, 20PE, 30PE, 50PE). The Short Time-series Expression Miner (STEM) software[113] was used to identify genes with different expression patterns. Furthermore, FPKM values from 29 different tissue samples were used as input data for a weighted gene co-expression network analysis (WGCNA)[83]. The FPKM values were $\log_2$ (x+1) transformed. The top 20,000 variant genes, the peanut orthologs of acyl-lipid genes identified in this section, were included for

WGCNA analysis using R software following the tutorial at https://labs.genetics.ucla.edu/horvath/CoexpressionNetwork/Rpackages/WGCNA/Tutorials/index.htm. To investigate enriched functions of identified co-expression modules, FatiGO was used to perform GO enrichment analysis[85].

S4.3.2 Acyl lipid metabolic genes. A total of 1,944 orthologous acyl-lipid genes were identified in cultivated peanut, of which 426 **(Supplementary Data Set 11)** were located within 125 QTL regions, thus could serve as good candidates for future mapping and gene cloning studies. Totals of 1,137 and 1,142 orthologous acyl-lipid genes were identified in *A. duranensis* and *A. ipaensis*, respectively **(Supplementary Table 9)**. These genes involved in acyl-lipid metabolism in these three species were assigned into 727 gene families with 465 *A. duranensis* and 487 *A. ipaensis* single copy genes distributed in 552 gene families, while 400 gene families have single copies in both species. For 50 of the 552 families, the single copy gene of wild peanut was duplicated at least once in cultivated peanut genomes. Among them, six families showed more than two duplicates in cultivated peanut involving 23 duplicated genes, perhaps playing an important role in acyl-lipid pathways. These 23 acyl-lipid genes were annotated to be important in acyl-lipid pathways, as nine were responsible for fatty acid synthesis, three for lipid signaling, and four for triacylglycerol (TAG) biosynthesis **(Supplementary Table 10)**. Besides, at least 205 single copy genes from the diploid species, with various functions, lost one copy or more after peanut tetraploidization.

S4.3.3 Regulatory network of acyl-lipid genes.

To identify genes that are in the regulatory network of acyl-lipid genes, a co-expression network was constructed using 20,858 genes, including top variant genes, peanut orthologs of acyl-lipid genes, and nodulation-related genes (in next section). A total of 30 modules were obtained with module size ranging from 43 (*saddlebrown*) to 4,820 genes (*turquoise* module) **(Supplementary Data Set 11b)**. Several modules contained large numbers of orthologs of acyl-lipid genes, such as *turquoise* (416 orthologs) and *blue* (202), mostly due to their large module size. Regarding both the percentage and number of orthologs of acyl-lipid genes, the *tan* module (358 genes) contained a high percentage (41.34%) and number (148) of acyl-lipid genes **(Supplementary Data Set 11b).** The GO enrichment analysis **(Supplementary Data Set 11c)** revealed that the *tan* module was enriched for lipid metabolic process, phospholipid-translocating ATPase activity, phospholipid transport, fatty acid biosynthetic process, fatty acid biosynthetic process, lipid binding, lipid transport as well as other functions or processes important for acyl-lipid biosynthesis **(Supplementary Data Set 11c)**. Genes in the *tan* module were involved in TAG, fatty acid synthesis, cuticular wax synthesis,

Omega- hydroxylation of laurate, lipid signaling, and lipase synthesis **(Supplementary Fig. 13)**. These discoveries provided important resources for exploring the gene networks of acyl-lipid biosynthesis in cultivated peanut. Further studies on genes in the networks may facilitate improvement of peanut oil quality and content.

S4.4 Analysis of nodulation related genes

S4.4.1 Methods. To find orthologous nodulation-related genes in the genomes of cultivated peanut and other legume species with reference genomes, the nodulation-related genes were collected from two recent, comprehensive searches for nodulation-related genes in *Medicago truncatula*, *Lotus japonicus*, and soybean (*Glycine max*)[114,115]. The protein sequences of nodulation-related genes were either retrieved as described below or downloaded from National Center for Biotechnology Information (NCBI, https://www.ncbi.nlm.nih.gov). In addition to *A. hypogaea*, the protein sequences of 12 other legume species were obtained, including *M. truncatula*, *L. japonicus*, *G. max*, *Phaseolus vulgaris* (Phytozome, https://phytozome.jgi.doe.gov), *Arachis duranensis*, *Arachis ipaensis* (PeanutBase, https://peanutbase.org), *Cicer arietinum*, *Cajanus cajan* (GigaDB, http://gigadb.org/dataset/100076), *Lupinus angustifolius*, *Trifolium pratense*, *Vigna angularis*, and *Vigna radiata* (LIS, https://legumeinfo.org/download). The orthologs of nodulation-related genes in these 13 species were firstly determined by using a combination of BLASTp reciprocal and bi-directional best hits (BBH) (E-value < 1e10-6)[66], and OrthoMCL (Inflation value 1.5 and other settings default)[116].

To investigate the evolution of nodulation and phylogenetic relationships of these 13 legumes species, a phylogenetic tree was constructed by using concatenated sequences of three nodulation genes whose orthologs were found in all species using both BBH and OrthoMCL methods. MEGA6 software[76] was used for phylogenetic tree construction. The best model was selected using a maximum likelihood method with 1000 bootstrap replications.

S4.4.2 *Nitrogen fixing symbiosis genes*. While being capable of biological nitrogen fixation, cultivated peanut utilizes a unique rhizobial infection mechanism distinct from model legumes and many other legume species[37]. The rhizobia enter peanut roots through cracks or openings where lateral roots were formed, in contrast to the root hair entry infection path in model legumes. The crack entry infection path was considered more ancient and less advanced than root hair entry, thus is more realistic to be transferred to non-legume species for

biological nitrogen fixation[38]. In total, 119 gene families were identified by BLASTp in all 13 species as nodulation-related, including the common symbiosis (SYM) signaling pathway genes: *NENA*, *POLLUX*, *NUP85*, *NUP133*, *SYMRK*, *CCaMK*, *NIN*, *ERN*, *NSP1*, and *NSP2* **(Supplementary Data Set 11d)**. Eight-one (68.07%) families of nodulation-related genes (including SYM signaling pathway genes) were found in all 13 species indicating the conservativeness of genetic mechanisms controlling nodulation across the legumes. The remaining genes were species-specific, which could be due to gene loss during speciation and evolution.

To compare the gene copy numbers of those putative orthologs between cultivated peanut and its two putative ancestors' genomes, a total of 79 gene families including 482 genes were obtained in cultivated peanut, versus 76 families of respectively 250 and 265 genes in *A. duranensis* and *A. ipaensis* (**supplementary Data Set 11e**). There were 36 gene families all containing single copy genes from both *A. duranensis* and *A. ipaensis* **(Supplementary Data Set 11e)**.

Three nodulation genes (*SYMRK*, *CCaMK*, *NSP1*) representing different steps in the SYM signaling pathway found in all 13 species were used to construct a phylogenetic tree **(Supplementary Fig. 14).** It was apparent that the SYM signaling pathway genes of *Arachis* species were distinct from those in other legume species. The SYM signaling pathway of the 10 non-*Arachis* species shared one common ancestor, which was the "sister" of *Arachis* species **(Supplementary Fig. 14)**. Based on the phylogenetic tree, the model species *M. truncatula*, *L. japonicus*, and *G. max* have all undergone more species divergence than *Arachis* species, indicating faster evolution. However, the SYM signaling pathway in *Arachis* species is more ancient among the 13 legume species. Thereby, the ancient nature and differential context of nodulation-related genes might provide an explanation of the specific nitrogen fixation mechanism in peanut[37,125].

Supplementary Note 5. Re-sequencing and phylogenetic analysis

S5.1 Plant material and Illumina sequencing

S5.1.1 Plant material. A total of fifty-two accessions from South America, China, India and the United States of America, were used for re-sequencing (**Supplementary Data Set 12**). They include three kinds of accessions: (1) 32 tetraploid peanut accessions representing original cultivars of two subspecies (*A. hypogaea subsp. hypogaea, A. h. subsp. fastigiata*) four varieties (var. hypogaea, var. hirsuta, var. fastigiata, and var. vulgaris) and bred cultivars derived from cross between *subspecies fastigiata var. vulgaris* only, such as

BS1016, Min6, SY27, Min8, M6-A, M8-B, EM7-10, or between *var. vulgaris* and *var. hypogaea*, such as HH1, LH11, ZH16, YZ9102, YH23; (2) 18 wild peanut accessions including *A. duranensis*, *A. stenosperma*, *A. hoehnei* and *A. cardenasii* with A-genome, *A. ipaensis* and *A. magna* with B-genomes, *A. batizocoi* of the K-genome, *A. pintoi* of the C-genome, *A. stenophylla* of the E genome, and *A. chiquitana* of the Pr genome and the unique tetraploid wild species *A. monticola*; and (3) Four synthetic tetraploid peanuts ISATGR5, ISATGR278, ISATGR1212 and ISATGR184 and their parents.

S5.1.2 Illumina sequencing. For genome sequencing of 52 cultivars, high quality genomic DNA was extracted from leaf tissue using a CTAB method[86] and 400-bp paired-end (PE) libraries were prepared using the NEBNext Ultra DNA Library Prep Kit for Illumina. Sequencing was performed by HiSeq X Ten machines at WuXi NextCODE (Shanghai, China). Raw data were trimmed through the NGS QC Toolkit (v2.3.3)[93] to remove adaptors, low quality bases, and reads containing more than 10% unknown bases ("N"). The cleaned reads were aligned to the peanut genome assembly using BWA software[88], and then the generated Sam files were used for variant calling by GATK software (version 3.6-0-g89b7209)[117.] Summary statistics for re-sequencing lines are shown in **Supplementary Table 11.**

S5.2 History of peanut origin

S5.2.1 Dating peanut history by collinear gene pairs

Colinear genes between (sub)genomes were retrieved and synonymous nucleotide substitution per synonymous site (Ks) was estimated for each colinear gene pair using the Nei-Gojobori approach[39].

Tetraploid peanut *A. hypogaea* originated much earlier than expected. By characterizing Ks values between colinear genes, based on a previous estimated mutation rate of 8.12 x 10^{-9} Ks/year, the divergence of Ad and At (peak Ks ~ 0.005), or Bd and Bt (0.0045) was dated to be around 420-470 thousand years ago **(Fig. 5b; Supplementary Note Table 5.2.2)**, which predated human habitation of the Americas, thus ruling out the possibility that human cultivation had been involved in producing *A. hypogaea*[1]. The split of At and Bt subgenomes (Ks ~ 0.028) was estimated to occur about 2.6 mya, as previously reported with A and B genomes (Ks ~ 0.029)[1]. These results conformed to the expansion times of LTRs of cultivated peanut and its diploid progenitors.

Supplementary Note Table 5.2.2 Ks estimation and the splitting dates for peanut (sub)genomes.

Colinear genes	Peak of Ks distribution	Standard error	Dates (MYA)
A. hypogaea A vs. *A. hypogaea B*	0.028	0.014	2.635+/-1.318
A. duranensis vs *A. ipaensis*	0.029	0.015	2.739+/-1.417
A. duranensis vs *A. hypogaea A*	0.005	0.004	0.471 +/-0.377
A. ipaensis vs *A. hypogaea B*	0.0045	0.004	0.424+/-0.377

S5.2.2 Estimation of peanut history with BEAST2

We have also performed exploration with BEAST2 using 41 single-copy genes from peanut subgenomes, Medicago, and *L. japonica* (Parameters: Gamma Category Count = 4, Subst Model = HKY, Frequencies = Empirical, Clock Model = Strict Clock), and found 27 genes to have an expected tree topology. However, we found there was a lot of variation among different gene trees. As to the rate used (8.12 x 10-9 Ks/year), the medians of inferred dates of splitting times of Ad_AhA, Ai_AhB, and Ad_Ai were 794 thousand years, 275 thousand years, and 3.01 million years, respectively. This analysis provides further support that humans did not contribute to the direct hybridization of tetraploid peanut. As a genomics exploration with all colinear genes may provide a relatively stable inference, dating time estimated using all colinear gene pairs was shown in the main manuscript.

Supplementary Note Table 5. 2. 2 Splitting dates inferred with 41 genes.

Group	*A. duranensis* vs *A. hypogaea* A	*A. ipaensis* vs *A. hypogaea* B	*A. duranensis* vs *A. ipaensis*
1	0.97	0.37	2.93
3	0.2451	0.2603	2.0319
5	4.3499	0.0908	7.7379
6	1.582	0.2476	3.2961
8	0.1899	0.1699	2.9492
9	1.2634	1.3013	3.2889
10	0.1969	0.226	3.4686
11	0.2822	0.2753	3.1083
12	0.1696	0.2072	2.0852
13	1.6454	0.4596	4.3032
15	0.8649	0.1068	2.326
16	1.0603	0.3746	2.6645
20	0.2027	0.2119	3.6221
22	0.4913	0.2958	2.1273
23	3.1135	0.2922	4.8952
24	0.3001	0.3961	2.0828
25	0.222	0.1008	2.081

26	0.4448	0.2338	8.2518
27	0.6039	0.0359	1.6657
28	1.2146	0.3808	6.1978
29	0.4497	0.1534	1.9273
30	0.7942	0.2766	3.0142
33	0.8462	0.2763	2.8684
34	0.3023	0.4709	2.2813
39	4.5491	0.2715	9.3492
40	0.8897	1.5616	3.3676
41	1.2061	1.7536	3.3177
Median	0.7942	0.2753	3.0142
Average	1.053696	0.400022	3.601452

S5.3 Phylogenetic analyses

S5.3.1 Methodology for alignment, SNP filtering and phylogenetic analysis

The raw reads from 52 accessions were filtered using Trimmomatic v 0.36 and mapped to the reference genome using BWA-MEM (https://arxiv.org/abs/1303.3997). The resulting alignment files were processed for removal of PCR duplicates using Picard Tools v2.17.10 (https://broadinstitute.github.io/picard/) and subjected to variant calling using HaplotypeCaller and GenotypeGVCFs of the Genome Analysis tool kit (GATK) v3.8[117]. The obtained SNPs were filtered using GATK filters (QD < 2.0 || FS > 60.0 || MQ < 40.0 || MQRankSum < -12.5 || ReadPosRankSum < -8.0) followed by HAPLOSWEEP v1.0[118] to remove homeologous SNPs. The identified InDels were filtered using GATK filters (QD < 2.0 || FS > 200.0 || ReadPosRankSum < -20.0). The filtered SNPs were used as an input to SNPhylo[74] (maximum likelihood method and 1000 bootstraps) to construct the phylogenetic tree **(Fig. 5c)**.

S5.3.2 Phylogeny of peanut accessions and related species

A total of 18.69 billion high quality reads from 52 genetically diverse peanut accessions **(Supplementary Data Set 12)** were mapped on the tetraploid genome, leading to identification of 17.16 million SNPs and 4.52 million InDels **(Supplementary Data Set 13; Supplementary Fig. 15)**. These variants can be deployed for developing better SNP arrays to perform high resolution genotyping in different genetic populations. Maximum variants were detected in intergenic regions followed by introns, missense synonymous variants, 3_prime UTR and 5_prime UTR variants.

Phylogenetic analysis grouped synthetic tetraploids with the diploids indicating high genetic distance

from the domesticated tetraploids **(Fig. 5c),** illustrating the value of synthetics as untapped diversity for diversifying the cultivated gene pool. Clustering also showed separate grouping of the two subspecies (*hypogaea* and *fastigiata*) of cultivated peanut as well as their progenitor genomes (AA and BB). The synthetic tetraploid, ISATGR 278-18 [*A. duranensis* (ICG 8138) × *A. batizocoi* (ICG 13160)] with AAKK genomes and ISATGR 5 [*A. magna* (ICG 8960) × *A. batizocoi* (ICG 8209)] with BBKK genomes perfectly grouped between their diploid progenitors, confirming the precise origin of these two synthetics. Similarly, the synthetic, ISATGR 1212, [*A. duranensis* (ICG 8123) × *A. ipaensis* (ICG 8206)] and ISATGR 184 [*A. ipaensis* (ICG 8206) × *A. duranensis* (ICG 8123)] with AABB genomes clustered between their progenitor species, however, showing unequal genome contributions (**Supplementary Table 11**).

S5.4 Circos and other analysis of peanut accessions

Circos analyses were used to visibly compare the SNP distribution patterns for three kinds of accessions: the tetraploid peanut accessions, the wild peanut accessions, and the synthetics and their parents as described above. All SNP information was gathered for statistics in a 1-Mb window along the reference genome. Within the window, three types of alleles were included: alternative alleles matching the reference alleles; alternative alleles that partly differed from the reference alleles (heterozygous); and alternative alleles that completely differed from the reference alleles (homozygous). These groups are marked by the scores 0, 1, and 2, respectively. The score of each position within the window was accumulated to a total score, as the variation density value in the window. All density values in all windows were used to draw the circus plot using the Circos software (v0.69)[67] (**Supplementary Fig. 16**). In addition, admixture, and principal component analysis (PCA) were used to analyzed the grouping and genetic relationship for the 52 accessions. PCA was performed using SNPRelate package of R[127] and Admixture package was used for estimating evolutionary ancestry[128]. Genetic distance of different groups were also analyzed by Fst using VCFtools[129]. All the analysis were performed with the 52 accessions according to the described procedures (**Supplementary Fig. 16**).

Supplementary Note 6 Linkage mapping, BSA and QTL discovery

S6.1 Yueyou 92 × Xinhuixiaoli populations

S6.1.1 Plant material. Two recombinant inbred line (RIL) populations for seed size and testa color were

created by crossing a parent with small seed with red testa color (Xinhuixiaoli) to one with bigger seed with pink testa color (Yueyou 92) in 2008 and 2010, respectively **(Fig. 6a,d,e)**. These two parents also contain other different characteristics. Yueyou 92 with pink seed color was used as female and this approach led to hybrids with red seed color as dominant inheritance from F1 selfing plants. Out of the 750 F2 plants, 198 (26.4%) showed pink seeds, indicating the monogenic recessive nature of the pink/red testa trait **(Supplementary Data Set 17a)**. From F2 plants, selfing and "single-seed descent" were performed to generate RIL populations. We developed 343 RILs of the population named "R" from 2008 crossing and 978 RILs of the population named "YX" from 2010 crossing. When the two RIL populations were respectively in F12 and F9 generations, they were used as mapping populations.

S6.1.2 SLAF library construction and high-throughput sequencing. DNA from 314 F12 and 267 F9 genotypes from the R and YX RILs populations were obtained and subjected to Slaf-seq strategy[55]. Published reference genomes of *A. duranensis* and *A. ipaensis* (http://peanutbase.org/) were used to design optimum marker discovery experiments by simulating different enzyme cuttings. Then, *Hae*III was used to digest genomic DNA. A nucleotide (A) was added to the digested fragments. Duplex tag-labeled sequencing adapters (PAGE-purified, Life Technologies, USA) were ligated to the A-tailed fragments using T4 DNA ligase. Polymerase chain reaction (PCR) was performed using primers (Forward primer: 5'-AATGATACGGCGACCACCGA-3', reverse primer: 5'-CAAGCAGAAGACGGCATACG-3') and the products were purified using Agencourt AMPure XP beads (Beckman Coulter, High Wycombe, UK), then pooled and separated by 2% agarose gel electrophoresis. Fragments ranging from 314-414 base pairs (with indexes and adaptors) for R population and 380-480 bp for YX were excised and purified using a QIAquick gel extraction kit (Qiagen, Hilden, Germany). Paired-end sequencing was performed on an HiSeq 2500 system (Illumina; San Diego, CA, USA). For YX population, we obtained 2,356.17M reads (470.55 Gb) with Q30 being 92.82% and the average sequence depth of SLAF loci covered 86.42X of parental genome size and 29.91X of each descendant. For the R population, we got 2868M reads with 27.04X coverage of each RIL.

S6.1.3 SLAF markers calling and genotyping. SLAF marker identification and genotyping were performed based on procedures of Sun *et al*[55]. Briefly, raw reads were sorted to individuals according to the barcode sequences. Low-quality reads (quality score < 20e) were filtered out and the barcodes and the terminal 5-bp were trimmed, then clean reads were mapped to the diploid A and B genome sequences using SOAP software[56]. Sequences mapping to the same position were defined as one SLAF locus[118]. Single nucleotide polymorphism (SNP) loci of each SLAF locus were then detected between parents, and SLAFs with more

than 3 SNPs were filtered out. Alleles of each SLAF locus were then defined according to parental reads with sequence depth >10, while for each offspring the reads with sequence depth cover 70% of progenies/RILs. For diploid species, one SLAF locus can contain at most 4 genotypes. Only SLAFs with two to four alleles were identified as polymorphic and considered potential markers. All polymorphic SLAFs loci were genotyped with consistency in the parental and offspring SNP loci. The polymorphic SLAFs were analyzed to screen for potential SLAF loci that consist of one segregation type (aa×bb) consistent with a RIL population. According to the procedure, for the YX population, we obtained a total of 790,026 SLAF loci with 64,753 polymorphic SNP, of which 12,990 were aaxbb type and 8019 were used for map construction. For the R population, we acquired 52,970 polymorphic SLAF loci, of which 9,612 were aaxbb genotype, and 5,344 used for map construction.

S6.1.4 Linkage mapping. The two sets of high quality SNP markers described above were used for constructing linkage maps for the R and YX population, respectively. Markers were assigned to 20 linkage groups on the basis of grouping by HighMap[57]. Firstly, we obtained a primary marker order by their location on chromosomes, according to relationships between ordered markers. Genotyping errors or deletion were corrected by the SMOOTH algorithm. After that, we used MSTmap to order the map, again again using SMOOTH to correct the new ordered genotypes. After 4 or more cycles, we have 20 high-quality linkage maps. Map distances were estimated using the Kosambi mapping function[119]. Finally, we constructed two high density linkage maps with 7183 and 5019 SNP markers, one (YX) spanning 2990.54 cM with 0.55 cM average marker distance, and the other spanning 2468.61 cM with 0.71 cM average marker distance **(Supplementary Data Set 16)**.

S6.1.5 Mapping and identification of seed color locus. Testa color was determined in F2 plants to be controlled by a single gene with red being dominant (552 red and 198 pink) **(Supplementary Data Set 17a)**. After we developed the high density SNP linkage map derived from F9 YX RIL population **(Supplementary Data Set 16)**, we performed linkage mapping and localized the seed color locus within a region of 0.905 cM on chromosome 3 which harbored 202 genes **(Supplementary Data Set 17b)**. This region contained several transcription factors such as 2 WRKY, 1 MYB and 2 bHLH families genes and 3 Cytochrome 450 genes which were known to regulate anthocyanidin biosynthesis[120,121], an anthocyanidin reductase and flavonoid 3'-monooxygenase of the anthocyanidin biosynthesis pathway that could directly control seed color[120]. Importantly, the Wrky 13 was identified with an SNP of G605A (R202Q) for the red color parents and RILs. We identified this variant by sequencing PCR products from the parents and 30 red testa RILs and 30 pink

testa Rils, and found 30 pink lines all having the “G” nucleotide at the 605 position in coding sequence, and 30 red lines show 29 “A” versus 1 “G”. This indicated that the Wrky 13 might be responsible for the seed testa color, although 1 RIL was not consistent, possibly due to heterozygosity **(Fig. 6a)**. Besides, we also performed RNA sequencing of the red and pink testa from the parents during seed development. As a result, we found that nearly all genes in the anthocyanin synthesis pathway show upregulation in the red seed color genotypes, including anthocyanidin reductase near the locus **(Supplementary Data Set 17c,d)**, which supports the relationship of these genes to red seed color.

S6.1.6 QTL mapping of seed size. We performed genome-wide QTL mapping of seed size for candidate gene discovery. Using the RIL population from Yueyou 92 and Xinhuixiaoli (YX) with a linkage map of more than 7000 SNP markers, six seed size related traits, including 100 pod weight, pod length, pod width, 100 kernel weight, kernel length, and kernel width were mapped using the QTL ICIMapping V3.3 software[84.] The threshold of logarithm of odds ratio (LOD) scores for evaluating the statistical significance of QTL effects was determined using 1,000 permutations. Intervals with a LOD value above 2.5 were detected as effective QTLs using the composite interval mapping (CIM) model of QTL IciMapping V3.3. According to the threshold, a total of 7 QTL regions were detected for the five traits using mean values from two years. Of the 7 QTLs, one stable and consistent QTL was found within 4.01 Mb on Chromosome 07 Mb with five of the six traits measured in 2016 and 2017 mapped in same region **(Supplementary Table 6.1.5).** By performing BLAST using the bordering primers, the genomic region was located on pseudomolecule Chr07 (0.87 - 1.95 Mb), (**Fig. 6a; Supplementary Data Set 18a,b**). Of the 99 genome-annotated genes in the salient region of chr07, 19 were identified to be related to seed development and seed size identity, including ABC transporters, oligopeptide transporter 5 (OPT5), Histidine kinase 2 (AHK2), Amino acid permease 3, transcriptional regulator STERILE APETALA (SAP), and others[45]. The AHK2 and SAP are orthologous genes in Arabidopsis that control shoot and seed growth and seed size [49], indicating the reference genome can promote trait mapping and candidate gene discovery.

S6.1.7 Transcriptome analysis of seed size and testa by RNAseq. To explain the mechanism of seed size and testa color, we also performed RNA extraction and Illumina sequencing as described above. Pod pericarps of 15 days after pegging (DAP) and embryos of 30 DAP were collected from the big and small seed parents Yueyou 92 and Xinhuixiaoli and big and small seed bulks of respective 30 RILs. The seed testa samples were collected just from the two parents. The sequencing and mapping results are provided as **Supplementary Note Table 6.1.7**.

Supplementary Note Table 6.1.7 Statistics of clean reads and the mapping for bulked-segregant groups for seed size and testa color.

Library	BE	BP	SE	SP	XE	XP	YE	YP
Total Reads	44,243,732	45,249,066	44,432,564	44,019,388	43,833,912	47,735,446	40,689,112	42,801,346
Mapped Reads	43,045,188	37,907,329	43,025,257	29,523,306	42,428,039	43,472,600	39,429,590	36,467,334
Mapping Rate	0.9729	0.8377	0.9683	0.6707	0.9679	0.9107	0.969	0.852
UnMapped Reads	1,198,544	7,341,737	1,407,307	14,496,082	1,405,873	4,262,846	1,259,522	6,334,012
MultiMap Reads	9,240,401	3,296,494	8,775,228	2,870,877	7,553,565	3,487,222	9,618,494	2,925,017
MultiMap Rate	0.2089	0.0729	0.1975	0.0652	0.1723	0.0731	0.2364	0.0683

Note: BE: embryo from RILs with big seed; BP: pericarp from RIL with big seed; SE: embryo from RILs with small seed; SP: pericarp from RIL with small seed; XE: embryo from Xinhuixiaoli; XP: pericarp from Xinhuixiaoli; YE: embryo from Yueyou 92; YP: pericarp from Yueyou 92.

S6.2 Pooled-sequencing based candidate gene discovery

S6.2.1 Plant material. The recombinant inbred lines (RILs) from Yueyou 92 × Xinhuixiaoli and TAG 24 × GPBD 4 were used for performing pooled-sequencing based discovery of associated genomic region and candidate gene discovery for pod size and foliar disease resistance (FDR), respectively. The resistance parent for FDR, GPBD 4, was derived from the cross KRG 1 × ICGV 86855 (CS 16) and the resistant parent, ICGV 86855 (CS 16), was an interspecific derivative of *A. cardenasii* i.e., the resistance source for both diseases.

S6.2.2 Methodology of QTL-seq approach. A total of 302 million high quality reads were generated from six bulk samples and four parental genotypes (**Supplementary Table 12**). The QTL-seq analysis was conducted from two RIL populations using the multi-season phenotyping data for three traits namely pod weight, rust resistance and late leaf spot (LLS) resistance. In brief, the bulks were made by pooling DNA from leaves of selected RILs with extreme phenotypes for these traits. For pod weight, the DNA from 54 RILs possessing low pod weight and 54 RILs with high pod weight were pooled from the population (Yueyou 92 × Xinhuixiaoli). Similarly, DNA from 25 RILs each for resistant and susceptible RILs (TAG 24 × GPBD 4) were pooled to constitute four bulks i.e., a resistant bulk for rust (Rust_Rbulk), susceptible bulk for rust (Rust_Sbulk), resistant bulk for LLS (LLS_Rbulk) and susceptible bulk for LLS (LLS_Sbulk). A total of 10 samples (2 each for pod weight, rust and LLS resistance, and 4 parents of the two RIL populations) were sequenced on an Illumina HiSeq 2500. The sequencing data was analyzed using the QTL-seq pipeline (http://genome-e.ibrc.or.jp/home/bioinformatics-team/mutmap) 120 for calculating SNP-index using the tetraploid genome assembly developed and reported in this manuscript. Initially, reference guided assembly for all four parental genotypes (TAG 24, GPBD 4, Yueyou 92 and Xinhuixiaoli) were made by substituting the

bases with confidence variant calls in the tetraploid genome. These reference guided assemblies were then used together with bulk samples for three traits and variants (SNP-index) were calculated. The ΔSNP-index for each trait was then calculated by subtracting SNP-index of one bulk from SNP-index of another bulk. Candidate gene discovery was performed in the genomic regions that were identified in this analysis.

S6.2.3 Discovery of genomic regions and candidate gene for seed size. Seed size is important for achieving higher productivity in peanut and we performed QTL mapping and sequence based candidate gene discovery using QTL-seq in the recombinant inbred line (RIL) population, Yueyou 92 and Xinhuixiaoli. From two parental genotypes (Yueyou 92 and Xinhuixiaoli) and two pools (big and small pods), a total of 1.29 billion high quality reads were used for carrying out QTL-seq analysis **(Supplementary Table 12)**. The QTL-seq analysis (**Supplementary Data Set 18d, e**) confirmed the two significant regions identified using genetic mapping. These two genomic regions are located on pseudomolecules Chr07 (0 - 2.525 Mb) and Chr12 (12.280-13.859 Mb), harboring a total of 99 and 97 candidate genes, respectively (**Supplementary Data Set 18b**). We could identify candidate genes mainly related to transport like ABC transporters, cytochrome P450, transcription factors like WRKY, F-Box, zinc-finger proteins, cell wall modifying enzymes like pectinesterase, signaling proteins and some disease resistance genes. The 248 significant SNPs identified in both the genomic regions included 143 from intergenic region, 66 intron variants, 17 missense variants, 13 synonymous variants, five 3_prime UTR variants, three 5_prime UTR variants and one splice region variant and intron variant.

S6.2.4 Discovery of genomic region and candidate gene for Rust and LLS resistance. Two foliar fungal diseases namely leaf rust (caused by *Puccinia arachidis*) and late leaf spot (LLS) (caused by *Cercosporidium personatum*) are of global importance due to huge yield loss and deterioration of fodder quality. A total of 1.73 billion high quality reads of two parents (TAG 24 and GPBD 4) and four pools (resistant and susceptible bulks) were used for performing QTL-seq analysis. The QTL-seq analysis for pooled samples from RIL, TAG 24 × GPBD 4, identified co-localized genomic regions on pseudomolecule_Chr13 for rust (140.405 - 144.882 Mb) and LLS (140.808 - 144.705 Mb). This region harbored 216 candidate genes for rust resistance **(Fig. 6c; Supplementary Data Set 19a, b)** and 171 for LLS resistance **(Supplementary Data Set 19c)** with their possible role in providing resistance to peanut plants against both foliar fungal diseases. The annotation of the candidate genes identified for rust and LLS resistance included TIR-NBS-LRR, PPR proteins, Glutathione-S-transferase, serine-threonine kinases, enzymes involved in MAPK and CDPK pathways and transcription factors like auxin response factor (ARF), WRKY and F-box. The sources of resistance for both

diseases were traced back to *A. cardenasii* to resistant variety, GPBD 4 (cultivated tetraploid), through ICGV 86855 (interspecific derivative). This genomic region seems to be translocated from Chr03 to Chr13 after tetraploidization **(Fig 6c and Fig 2c)** as previous QTL-seq analysis using genome assembly of a diploid progenitor (*A. duranensis*) identified a genomic region associated with rust and LLS resistance on Aradu.A03 [121]. However, as shown above chr03 and chr13 had undergone translocation of 10 Mb on the terminal regions between the chromosomes after tetraploidization, which may result in the SNPs and Indels within this region of chr03 mapping to chr13. Therefore, this tetraploid assembly provides opportunities for performing precise and rapid candidate gene discovery which can be validated and deployed in cultivated tetraploids through molecular breeding.

Supplementary Note Table 6.2.4 Summary of genome-wide SNPs identified and their functional annotation.

Functional annotation of SNPs	Rust resistance	LLS resistance	Pod size
Splice_region_variant&synonymous_variant	3	1	0
Synonymous_variant	131	52	13
Stop_gained	3	0	0
3_prime_UTR_variant	103	61	5
5_prime_UTR_premature_start_codon_gain_variant	6	3	0
5_prime_UTR_variant	29	9	3
Splice_acceptor_variant&intron_variant	2	1	0
Missense_variant&splice_region_variant	4	1	0
Missense_variant	122	51	17
Intron_variant	838	447	66
Intergenic_region	2010	981	143
Splice_donor_variant&intron_variant	3	0	0
Splice_region_variant&intron_variant	16	13	1
Total	3270	1620	248

S6.2.5 Discovery of genomic region and candidate gene for seed test color. Seed testa color is also an important economic trait for some usages and a mark trait in breeding. By the reference genome we also mapped the red testa locus in the cross of Yueyou92 and Xinhuixiaoli (**Fig. 6a**). The red color mapped in the F2 progeny was controlled by a single dominant gene (552 red and 198 pink; **Supplementary Data Set 17a**). By linkage mapping using recombinant inbred line (RIL) population (**Supplementary Data Set 16; Supplementary Data Set 17b**), the seed color was mapped within a region of 0.905 cM on chromosome 3, which covered 202 genes (**Fig. 6a; Supplementary Data Set 17c**). This region harbored 202 genes (**Fig. 6a;**

Supplementary Data Set 17c) including transcription factor WRKY, MYB and bHLH family and cytochrome 450 genes associated with regulation of anthocyanidin biosynthesis[42,43]. An anthocyanidin reductase and flavonoid 3'-monooxygenase of the anthocyanidin biosynthesis pathway were also found near the seed testa color locus[42]. Twenty-seven genes were significantly upregulated and 15 downregulated in the region of the red seeded parent. The expression of anthocyanidin reductase and two testa specific genes CYP716A1 and Ethylene response factor increased 3 to 10-fold (**Supplementary Data Set 17c, d**). Also, WRKY13 had a SNP mutation of G605A (causing R202Q) that co-segregated with red color. Upregulation of anthocyanin synthesis genes in the red seeded parent (**Supplementary Data Set 17e**) may cause red seed color.

S6.2.6 Locating genomic regions for important mapped QTLs. Several scores of agronomical important quantitative and qualitative traits have been genetically mapped in peanut (**Supplementary Data Set 15**), but the functional genes underlying the traits are unknown. Through blasting analysis using flanking DNA markers, 40 quantitative traits such as seed sizes, yield and quality, resistance, and plant characters were mapped to pseudomolecules in specific locations (**Supplementary Fig. 18; Supplementary Data Set 15**). By employing the nearby SSR, SNP or InDels for markers, it will permit fine mapping of these trait for the candidate genes. Apparently, the reference genome presented here will allow fine mapping of QTLs and the genome-wide selection of peanut with required traits.

Supplementary Note 7. Mutant analysis and candidate gene discovery for high oleic acid

S7.1 Plant material

The high oleate mutant Min6-A was derived from Minhua 6 treated by ethyl methanesulfonate (EMS), while high oleate mutant Min8-B was derived from Minhua 8 using physical mutagen, r-ray **(Supplementary Table 13).**

S7.2 Methodology of mutant analysis

From the identified variations as mentioned in Section **5.3.1. Methodology for alignment, SNP filtering and**

phylogenetic analysis, the polymorphic calls between Minhua 6 and Min6-A, Minhua 8 and Min8-B were identified using an in house perl script. These variations were further studied for their effect using SnpEff[122].

S7.3 Candidate gene discovery by analyzing high oleic acid mutants

High oleic acid in the seeds is a main target of peanut quality improvement as it contributes to better flavor and longer storage life of peanut products and also benefits human cardiovascular health. We created and resequenced two high oleic (>80% oleic acid) mutants namely Min6-A and Min8-A by EMS treatment or r-ray radiation of parents, Minhua 6 and Minhua 8, respectively **(Supplementary Table 13; Supplementary Data Set 20a).** Sequence analysis of two mutants together with their original parents detected mutation in microsomal oleoyl-PC desaturase genes, *ahFAD2A* (*AH09G33970* at 114779221bp of Chr09*)* and *ahFAD2B* (frameshift mutation of *AH19G43590* at 154464257bp of Chr19), which lead to high oleate[50] **(Supplementary Data Set 20b, c)**. The analysis further revealed the location of FAD2 genes (*ahFAD2A* and *ahFAD2B)* in pseudomolecules Chr09 and Chr19 of tetraploid genome assembly. It was intriguing that we used different cultivars and treated them separately by EMS and by r-ray radiation at different times but scores of high oleic acid mutant varieties showed exactly the same mutations, SNP and Indels. These further clarified not only SNP but also Indel hotspots during plant evolution. These results also explain that the mutations happened simultaneously on *FAD2* genes located on homeologous pseudomolecules (Chr09 and Chr19) of the tetraploid genome leading to high oleic peanut in the mutants.

Supplementary Note Table 7.3 Summary of genome-wide significant SNPs identified through mutant analysis and their functional annotation.

Functional annotation SNPs	Minhua 6 vs. Min6-A	Minhua 8 vs. Min8-B
3_prime_UTR_variant	516	514
5_prime_UTR_premature_start_codon_gain_variant	51	55
5_prime_UTR_variant	269	273
Conservative_inframe_deletion	7	4
Conservative_inframe_insertion	5	9
Disruptive_inframe_deletion	5	6
Disruptive_inframe_insertion	2	3
Frameshift_variant	87	137
Frameshift_variant&splice_acceptor_variant&splice_region_variant&intron_variant		1
Frameshift_variant&splice_donor_variant&splice_region_variant&intron_variant	2	1
Frameshift_variant&splice_region_variant	5	6

Frameshift_variant&start_lost	1	1
Frameshift_variant&start_lost&splice_region_variant	1	1
Frameshift_variant&stop_gained	3	2
Frameshift_variant&stop_lost&splice_region_variant	0	1
Initiaton_codon_variant	0	1
Intergenic_region	154,043	129,380
Intron_variant	15,004	14,041
Missense_variant	2,485	2,503
Missense_variant&splice_region_variant	71	76
Splice_acceptor_variant&intron_variant	41	36
Splice_donor_variant&intron_variant	26	42
Splice_region_variant	8	13
Splice_region_variant&intron_variant	228	212
Splice_region_variant&stop_retained_variant	2	2
Splice_region_variant&synonymous_variant	23	25
Start_lost	10	10
Start_lost&conservative_inframe_deletion	1	0
Stop_gained	87	79
Stop_gained&splice_region_variant	2	7
Stop_lost	5	6
Stop_lost&splice_region_variant	10	11
Synonymous_variant	1,101	1,118
Total	174,101	148,576

Supplementary Figures

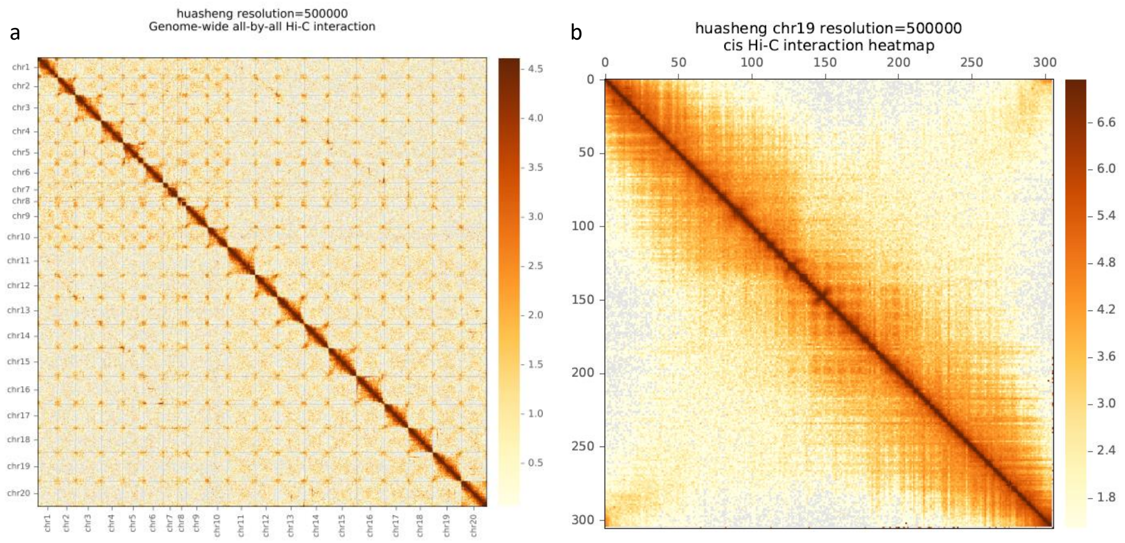

Supplementary Figure 1. Heatmap of chromosome conformation capture analysis. (a) Synthetic intra-chromosomal contact matrix, and **(b)** Heatmap of an intra-chromosomal contact matrix of chromosome 19. The intensity of interaction represents the normalized count of Hi-C links between 500 Kb bins on a logarithmic scale. The colored bar on the right side of the figure indicates the strength of interaction i.e., low (yellow) to high (red).

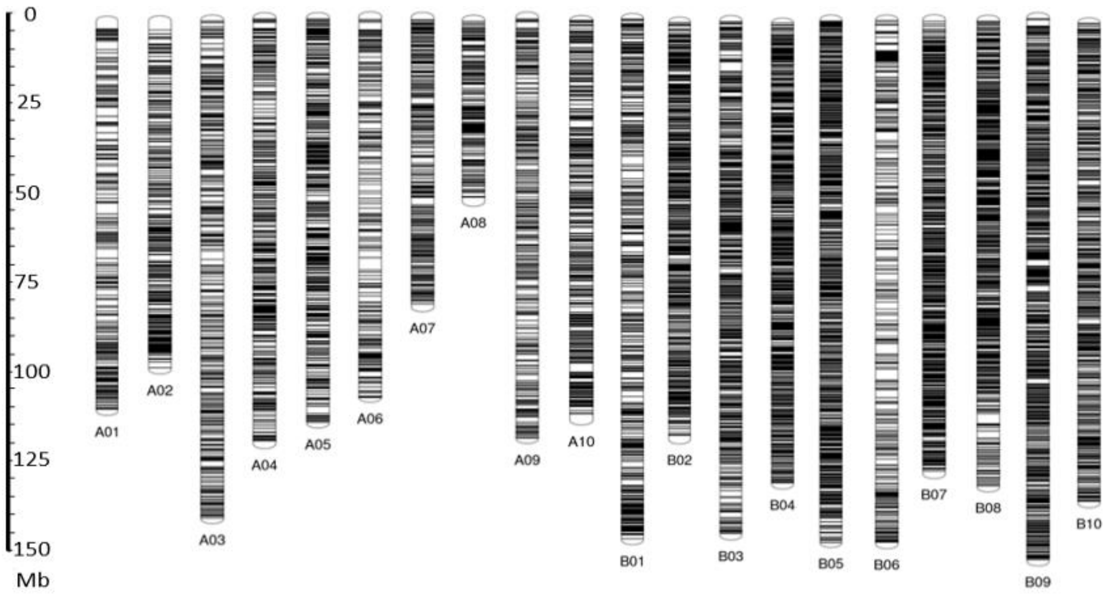

Supplementary Figure 2. Tetraploid peanut linkage map. This Linkage map with 14,619 mapped loci generated by integrating maps from four independent peanut populations using Allmap. Black bands in each linkage group represent mapped loci. Letters and the number next to each linkage group indicate the assigned linkage group names from the A or B sub-genome and the numbering of chromosomes, respectively (for example, A01 is equivalent to LGA01). Scale bar, Mb.

a

b

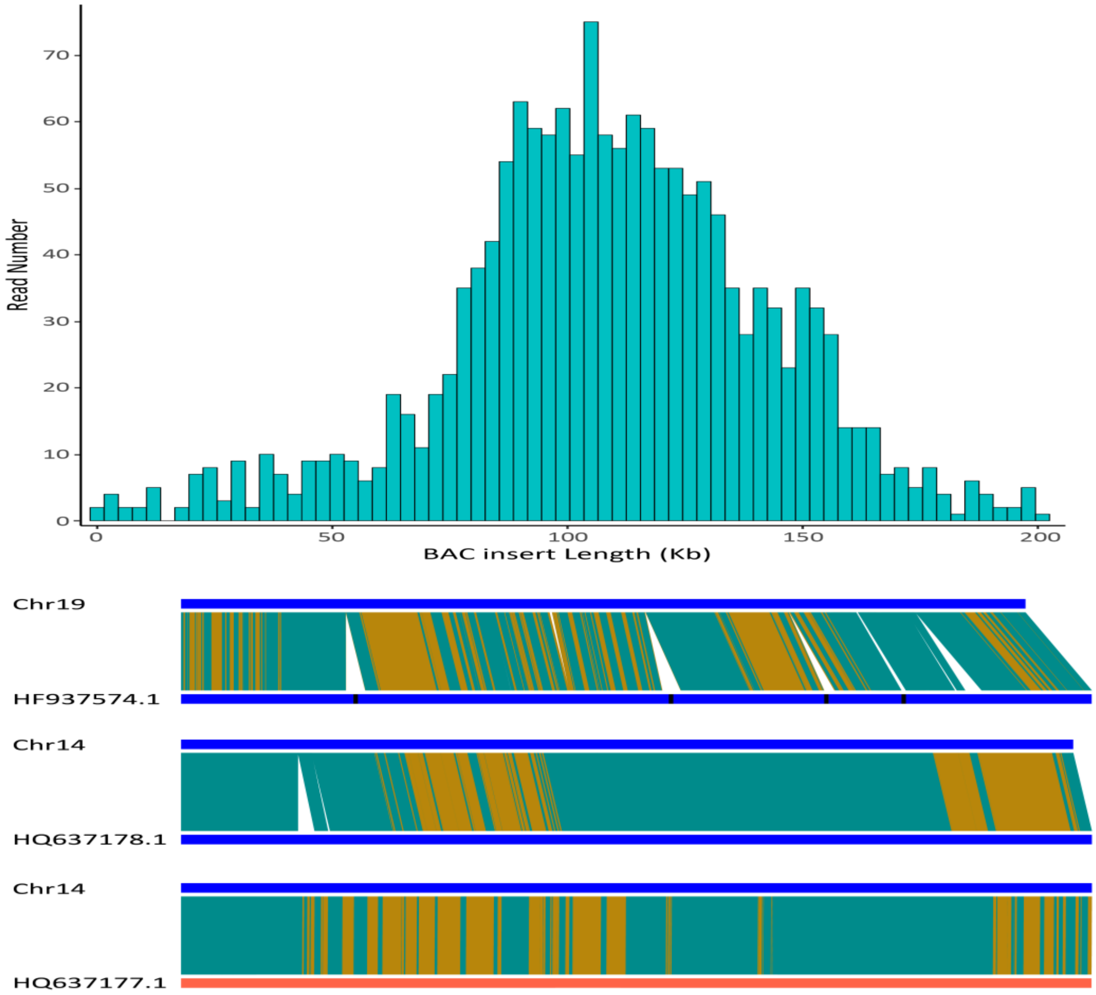

c

GenBank accession	Chr	Chr length (bp)	Start (bp)	End (bp)	BAC length (bp)	Gap length in BAC	Gap number in BAC	Coverage (bp)	Coverage (%)	Identity (%)
HF937574.1	19	159,154,999	157,603,801	157,697,993	101,591	2000	4	93,650	92.18	99.98
HQ637178.1	14	135,085,854	117,930,541	118,031,957	103,534	0	0	101,412	97.95	99.30
HQ637177.1	14	135,085,854	117,758,630	117,852,939	94,268	0	0	94,230	99.96	99.94

Supplementary Figure 3. Evaluation of peanut genome assembly with BAC.
(a) Distribution of gap length between pair-end sequences of 1,576 BACs matched to the reference genome. The average of length between matched BAC ends is near 110 kb on average. The available BACs of *A. hypogaea* are from NCBI. **(b)** Collinearity of the peanut genome assembly and three available BAC sequences. The yellow color lines indicate repeat sequences. It shows 100 % of coverage with over 99.3 % sequence identity for all three BACs. The arrows indicate the regions of BAC sequences and pseudomolecules which did not matched owing to polyN within the BAC sequences. **(c)** The blast results of three published BACs of *A. hypogaea* mapping to the reference genome.

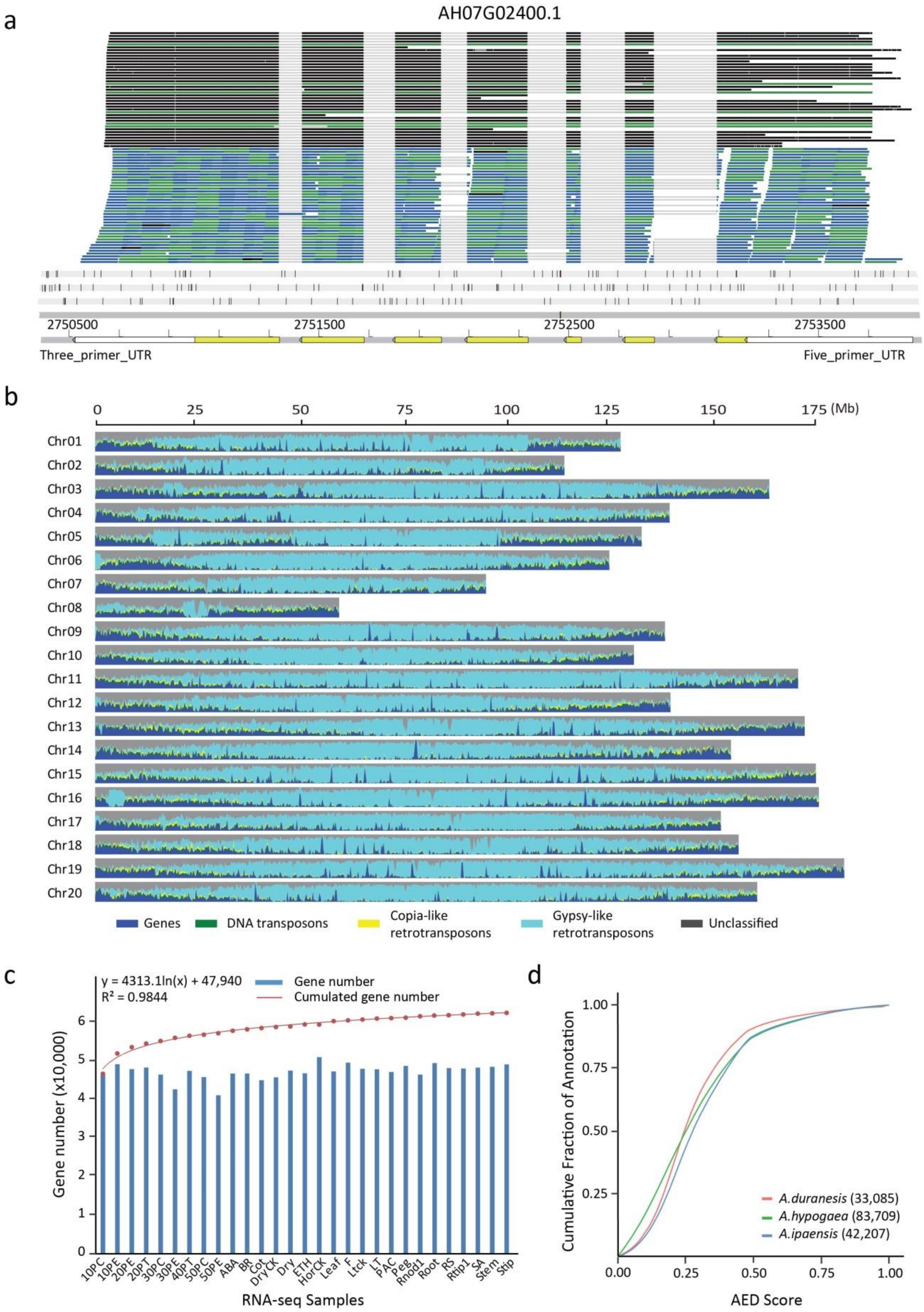
a
AH07G02400.1
2750500
2751500
2752500
2753500
Three_primer_UTR
Five_primer_UTR
b
0
25
50
75
100
125
150
175 (Mb)
Chr01
Chr02
Chr03
Chr04
Chr05
Chr06
Chr07
Chr08
Chr09
Chr10
Chr11
Chr12
Chr13
Chr14
Chr15
Chr16
Chr17
Chr18
Chr19
Chr20
Genes
DNA transposons
Copia-like retrotransposons
Gypsy-like retrotransposons
Unclassified
c
y = 4313.1ln(x) + 47,940
R² = 0.9844
Gene number
Cumulated gene number
Gene number (x10,000)
0
1
2
3
4
5
6
10PC
10PE
20PE
20PT
30PC
30PE
40PT
50PC
50PE
ABA
BR
Cot
DryCK
Dry
ETH
HorCK
Leaf
F
Ltck
LT
PAC
Peg
Rnod1
Root
RS
Rtip1
SA
Stem
Stip
RNA-seq Samples
d
Cumulative Fraction of Annotation
1.00
0.75
0.50
0.25
0.00
0.25
0.50
0.75
1.00
A.duranesis (33,085)
A.hypogaea (83,709)
A.ipaensis (42,207)
AED Score

Supplementary Figure 4. Illustration of peanut genome annotation.

(a) Representative gene model showing mapped RNA sequencing reads generated using Illumina or isoform sequencing technologies. The top and middle panels show isoform sequencing and RNA-seq reads, respectively, that have been mapped to the chromosomal location containing the AhA07G0390.1 gene model, which is shown on the bottom panel. Light grey lines in the top two panels indicate regions where reads were split to indicate positions of introns. Full-length isoform sequencing reads were able to span the 5′ untranslated region, all exons, and the 3′ untranslated region in a single read. **(b)** Gene density and repeat distribution on the 20 assembled peanut chromosomes. The components noted with different color at bottom demonstrate a differed distribution of density along chromosomes. Categories were determined with a 0.1-Mb shift. **(c)** Peanut gene saturation curve. The blue columns indicate the single locus gene number from RNA sequencing of 29 samples from different tissues and cross circumstances. The red curve indicates accumulation of single locus genes (for supporting gene model annotation) with increase of RNA-seq samples. **(d)** The frequency of annotation edit distance (AED) scores for the assemblies of tetraploid peanut (green), *A. duranensis* (red) and *A. ipaensis* (blue).

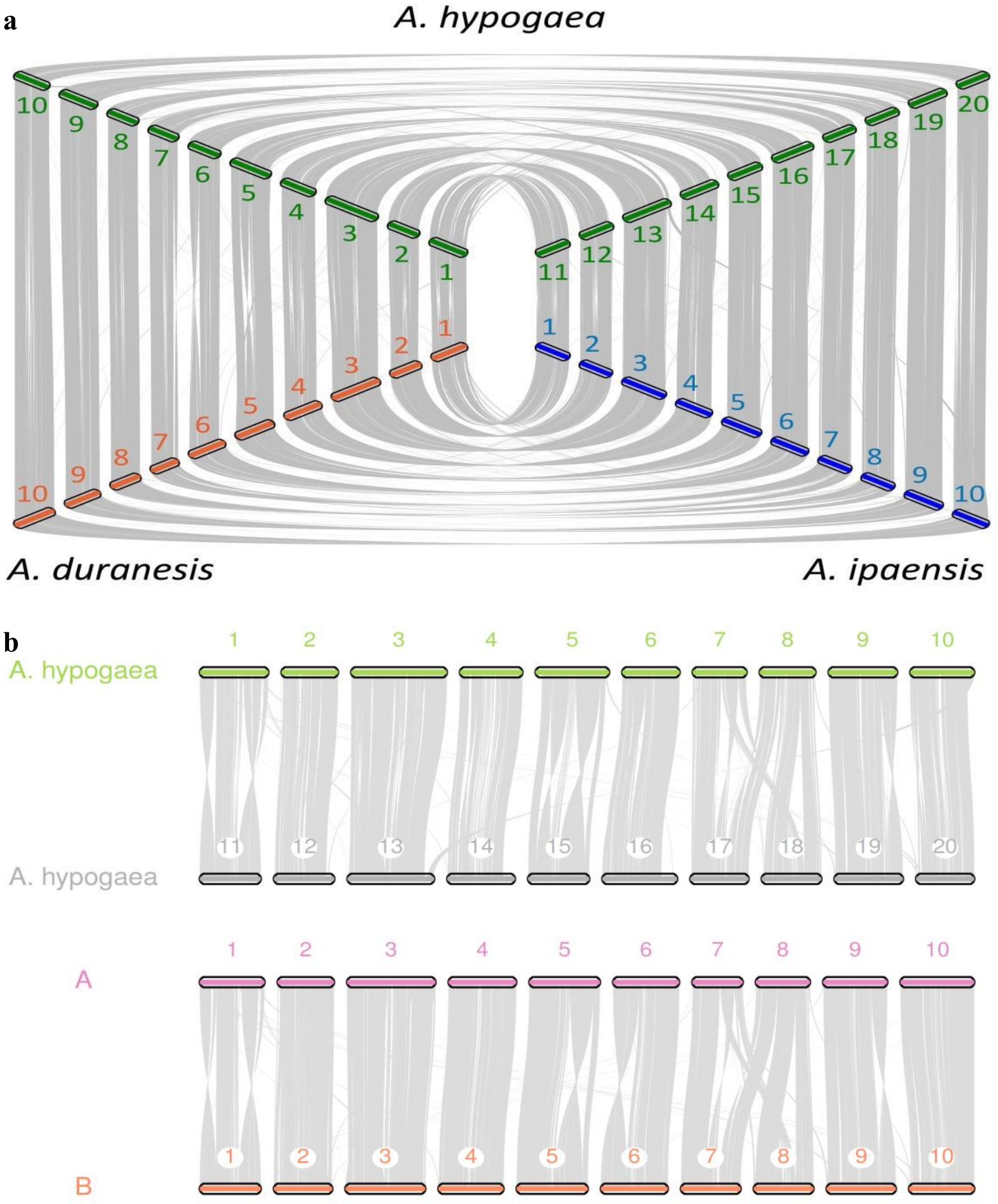

Supplementary Figure 5. Syntenic comparisons and recombination analysis. (a) Reciprocal syntenic comparison of peanut and its ancestral diploid genomes. There are more recombinations between A and B sub-genomes in tetraploid peanut than between A and B genome of diploid peanuts. (b) Collinearity between sub A- and B-genome in tetraploid peanut and A and B genome in diploid peanuts. It shows similar patterns of chromosome recombinations between sub A and B subgenome of cultivated peanut vs. A and B genomes of wild peanut, though cultivated peanut contains more inversion and recombinations.

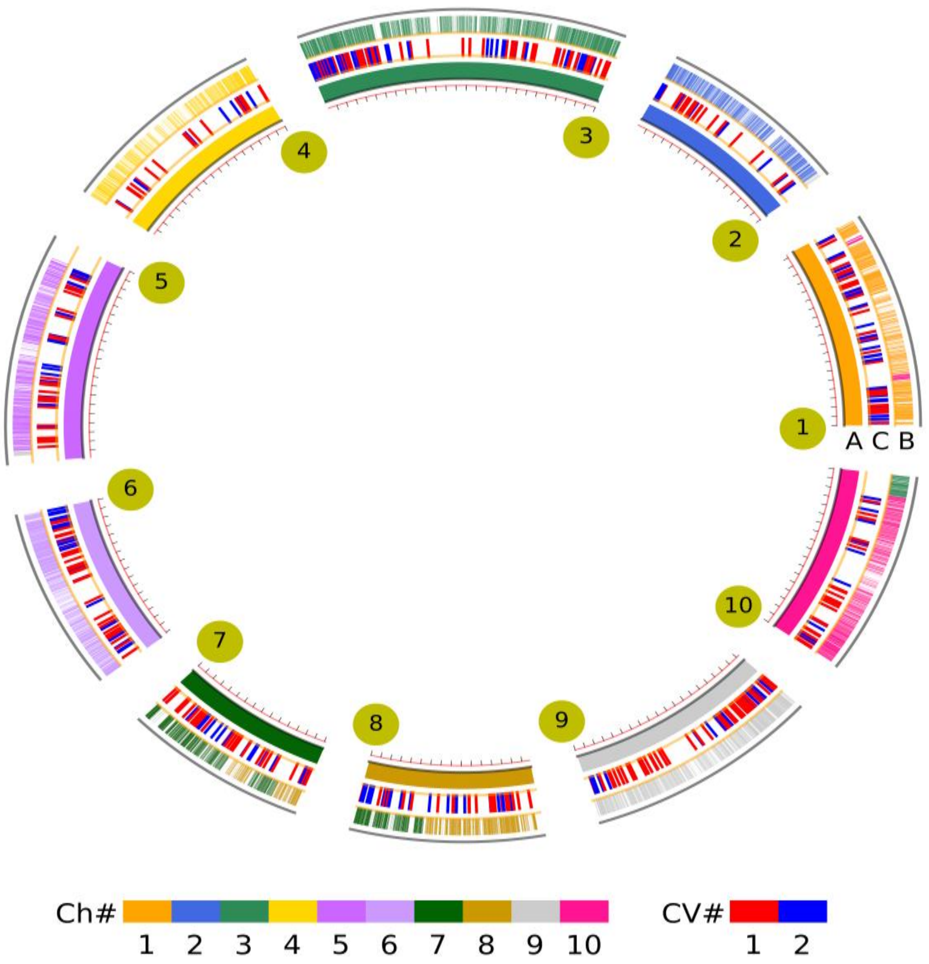

Supplementary Figure 6. Gene conversion in peanut.

Converted genes are marked out with short lines between subgenome A and B, with red ones showing donor genes from subgenome A, and blue ones showing those from subgenome B. Circles A, B and C indicate A subgenome chromosomes, B subgenome chromosomes, and the conversion positions, respectively.

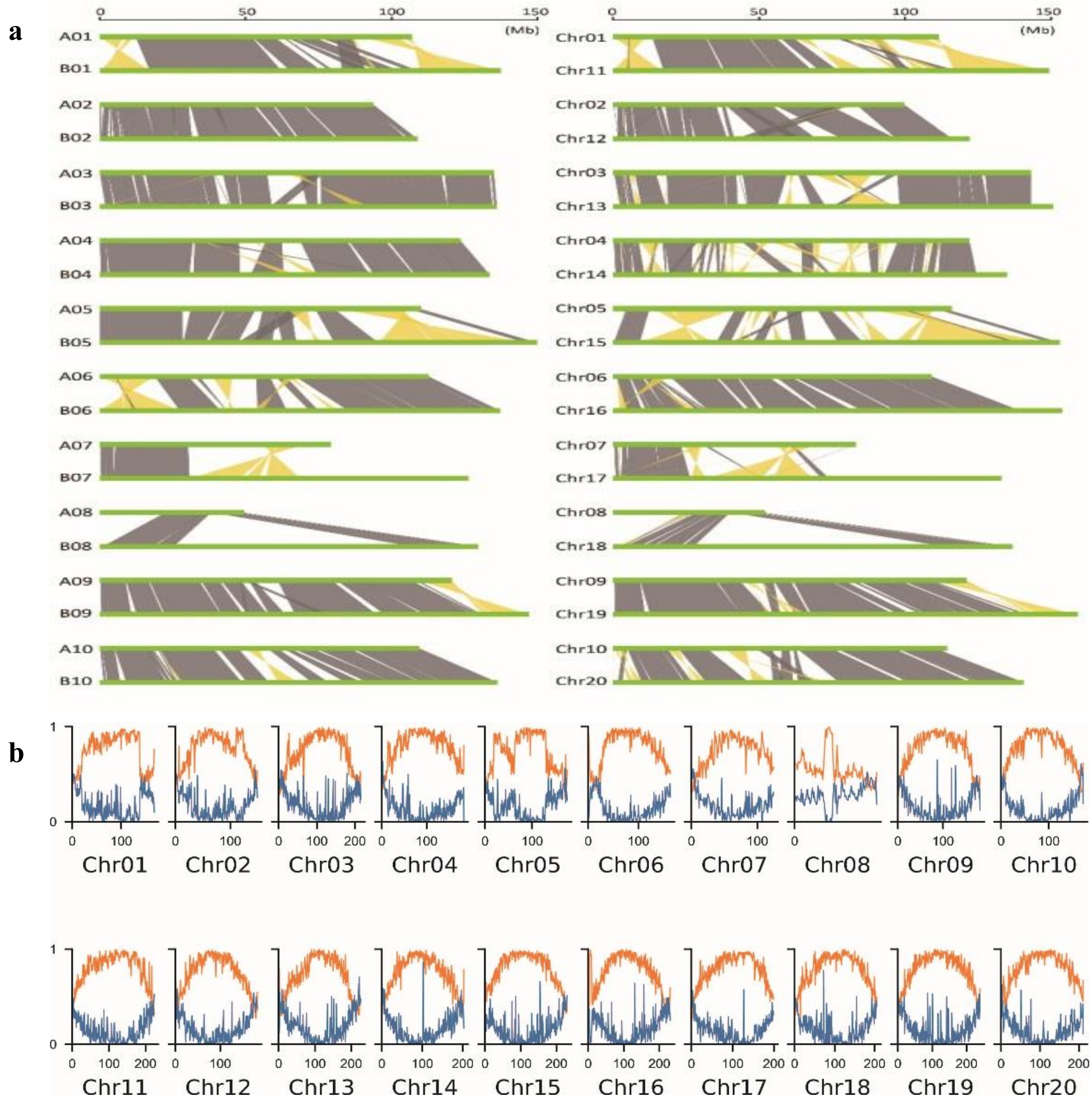

Supplementary Figure 7. Syntenic comparison and density of genes and main repeats analysis. (**a**) Syntenic comparison between AA and BB subgenome of *A. hypogaea*. Each syntenic block contains 30 or more genes, with sequence similarity of 80% or more. Besides chr02 and chr12 chromosome pairs, single to several inversions detected within each pair of chromosomes. **(b)** Densities of genes and Ty3-gypsy and non-autonomous mobile elements along pseudomolecules of subgenomes. The X-axis shows the bp coverage along the whole chromosomes within 500kb while the Y-axis in left are percentage of genes or repeat content per 500kb window. The orange color indicates repeat elements density, while blue color indicates genes.

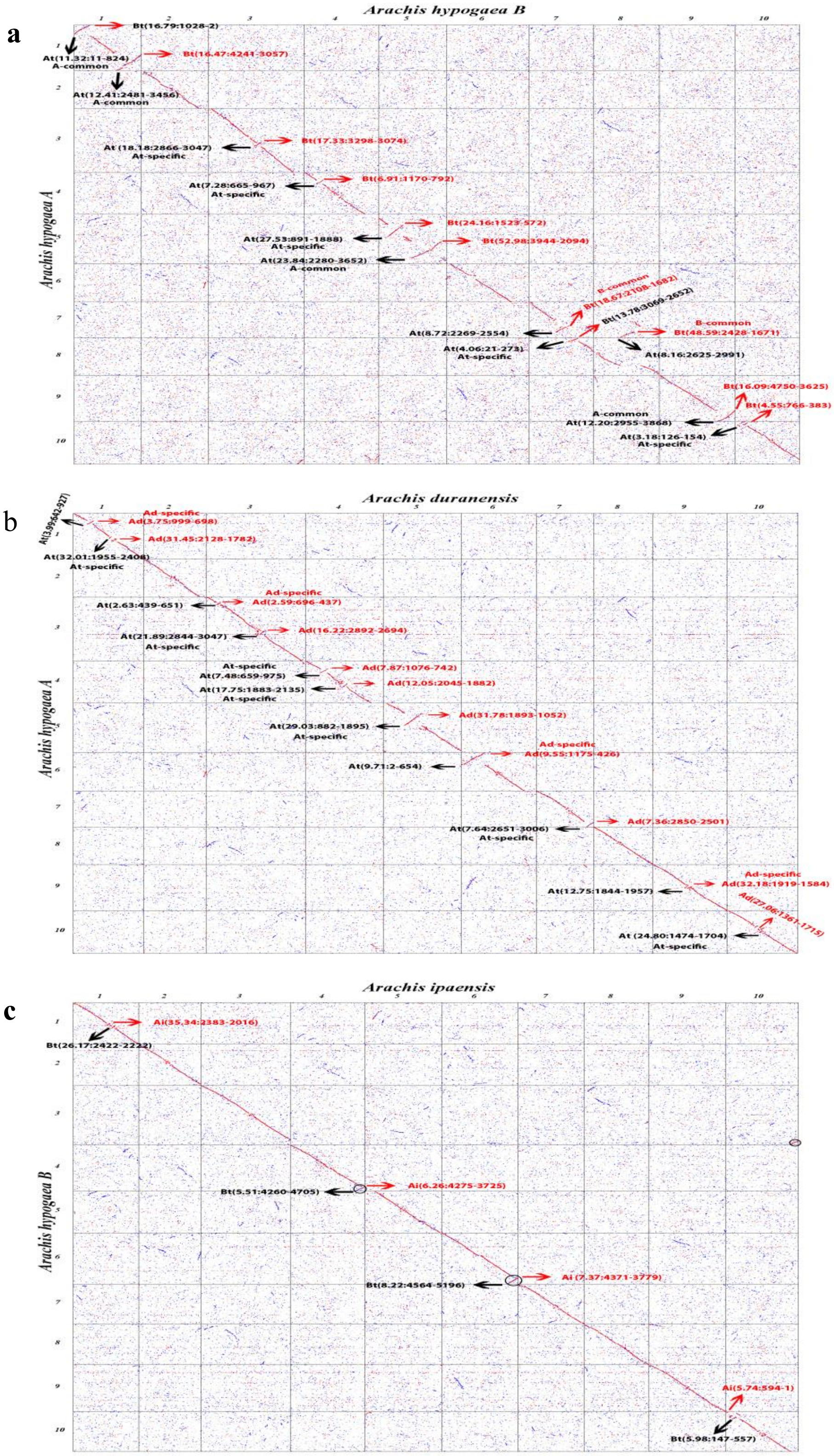

Supplementary Figure 8. Syntenic comparison of *A. hypogaea* itself versus *A. duranensis* and *A. ipaensis*. After tetraploidization, eleven genomic regions showed inversions between A subgenome and B subgenome of *A. hypogaea* (**a**), and also *A. hypogaea* and diploid *A. duranensis* (**b**), and only four smaller inversions happened between B subgenome of *A. hypogaea* and diploid B genome of *A. ipaensis* (**c**).

Supplementary Figure 9. Inference legume ancestral karyotypes and their evolutionary into peanut karyotype.

a

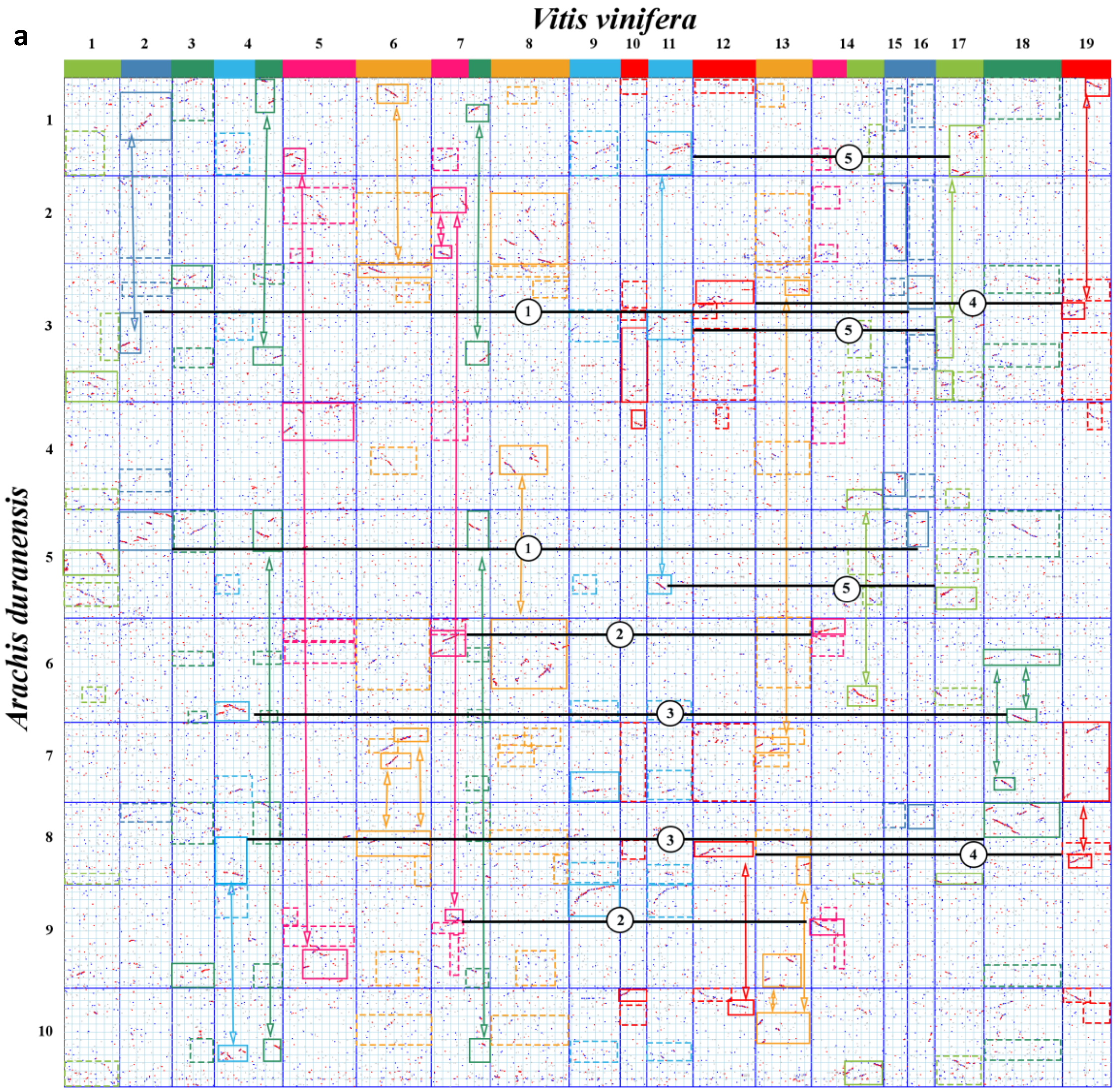

Supplementary Figure 9a. Dotplot between grape and *A. duranensis*.

By comparing the grape and peanut A genome by using homologous gene dotplot, we inferred ancestral chromosome fusions during evolution. A fusion between two chromosomes could be identified by the repetitive co-occurrence of their broken or duplicated segments in different extant legume chromosomes. For example, two ancestral chromosomes, corresponding to grape chromosomes 6 and 13, respectively, were inferred to have fused before the legume-common whole-genome duplication, in that their respective chromosomal segments were found to co-occur four times in peanut and other legume genomes. Eventually, 5 independent chromosome fusion events, including 3 nested chromosome fusions (NCF) and 2 chromosome end-end joining (CEJ) fusions were identified and therefore, the karyotype before the LCT was inferred to have 16 ancestral chromosomes, which were duplicated after the event. Grape chromosomes are shown in seven colors, corresponding to seven ancestral chromosomes before the eudicot-common hexaploidy, or ECH (see main text for information). Numbers in circles show fused chromosomes.

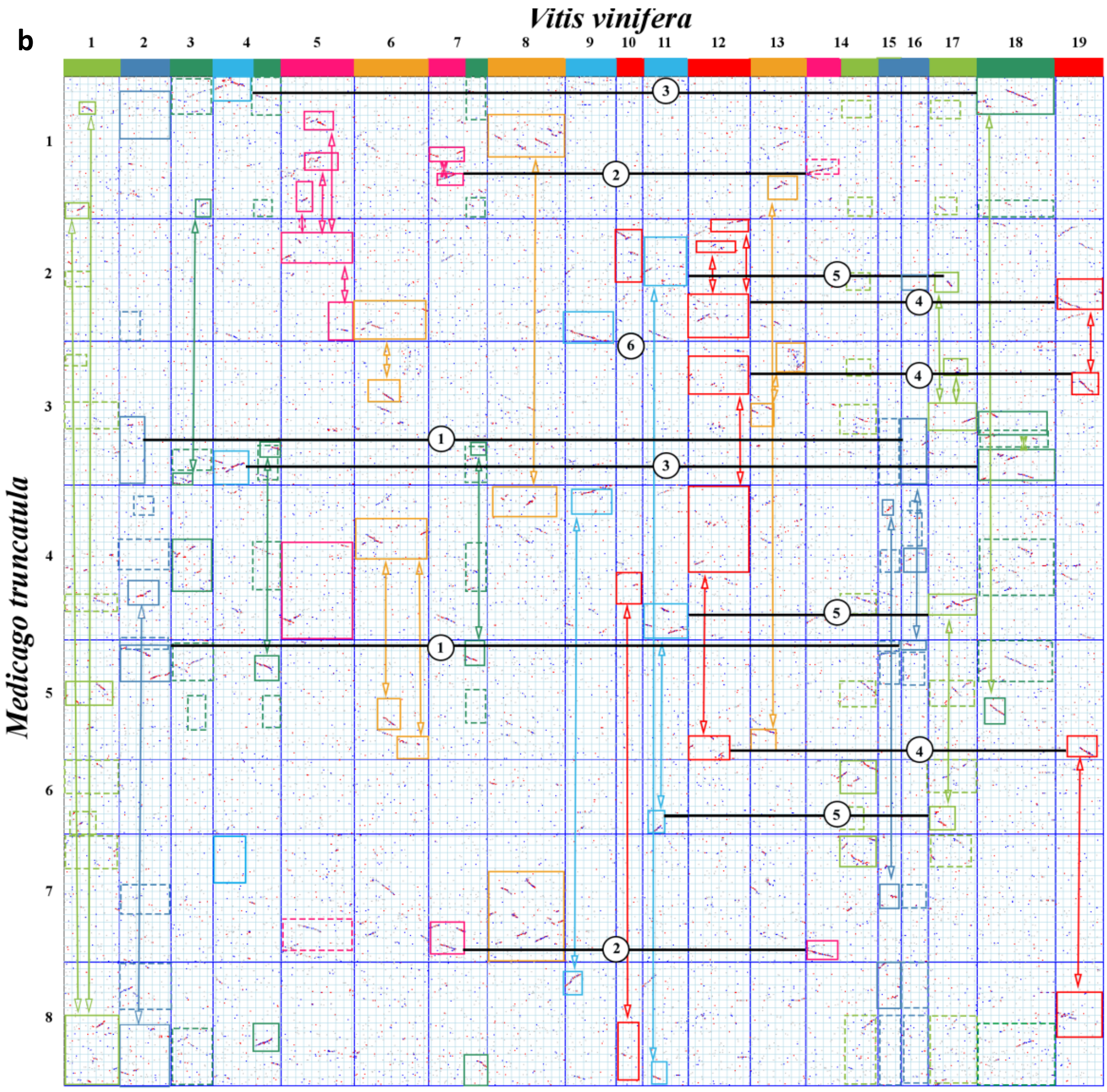

Supplementary Figure 9b. Dotplot between grape and barrel medic.
Please see legend for subfigure Extended Data Figure 10a for information.

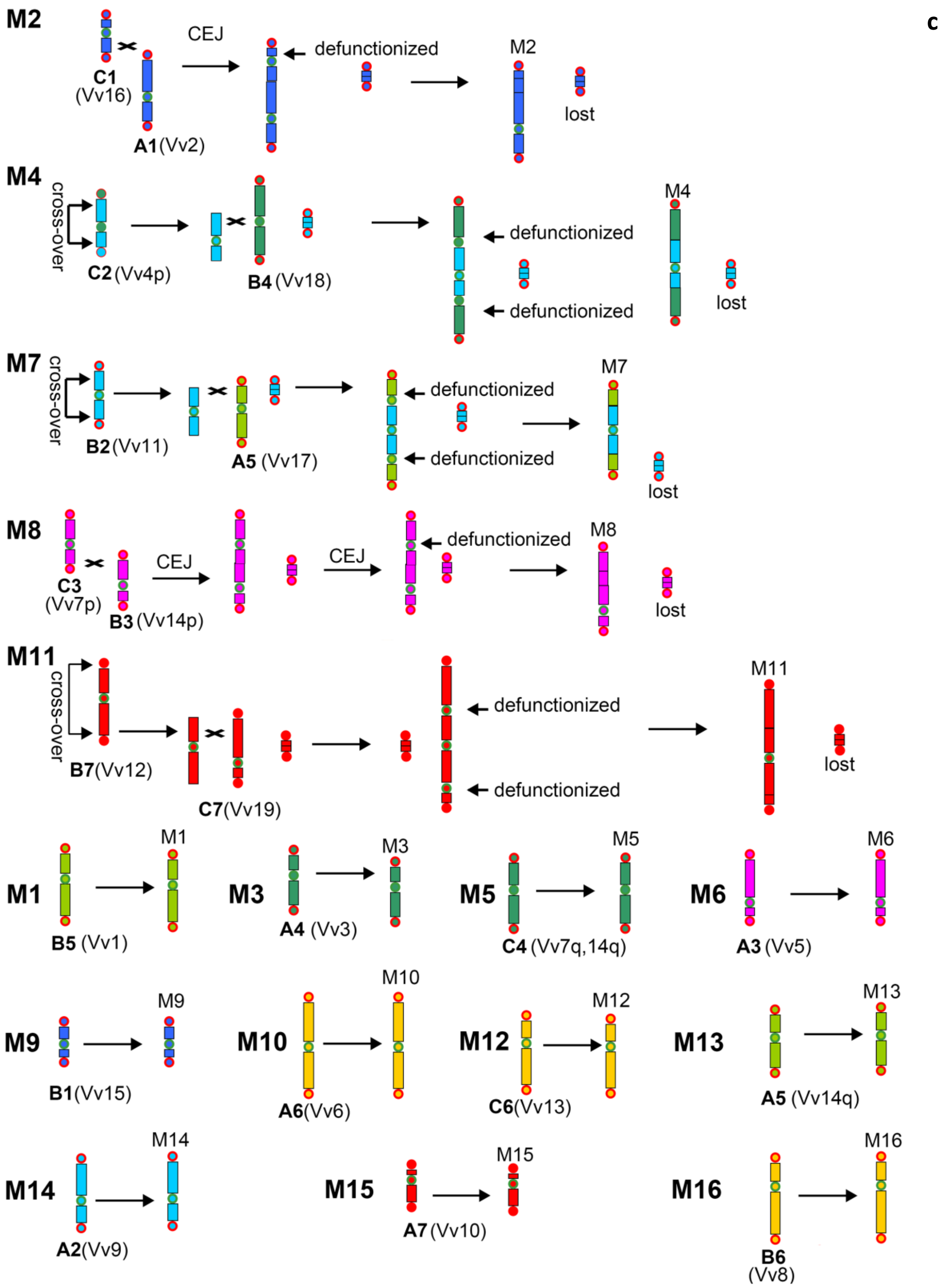

Supplementary Figure 9c. Production of 16 pre-LCT chromosomes.

From 21 post-ECH chromosomes, 16 pre-LCT chromosomes could be inferred. The LCT is an abbreviation of legume-common tetraploidy. Please see legend for subfigure Extended Data Figure 10a for information. CEJ: chromosome end-end joining; X: a crossing-over.

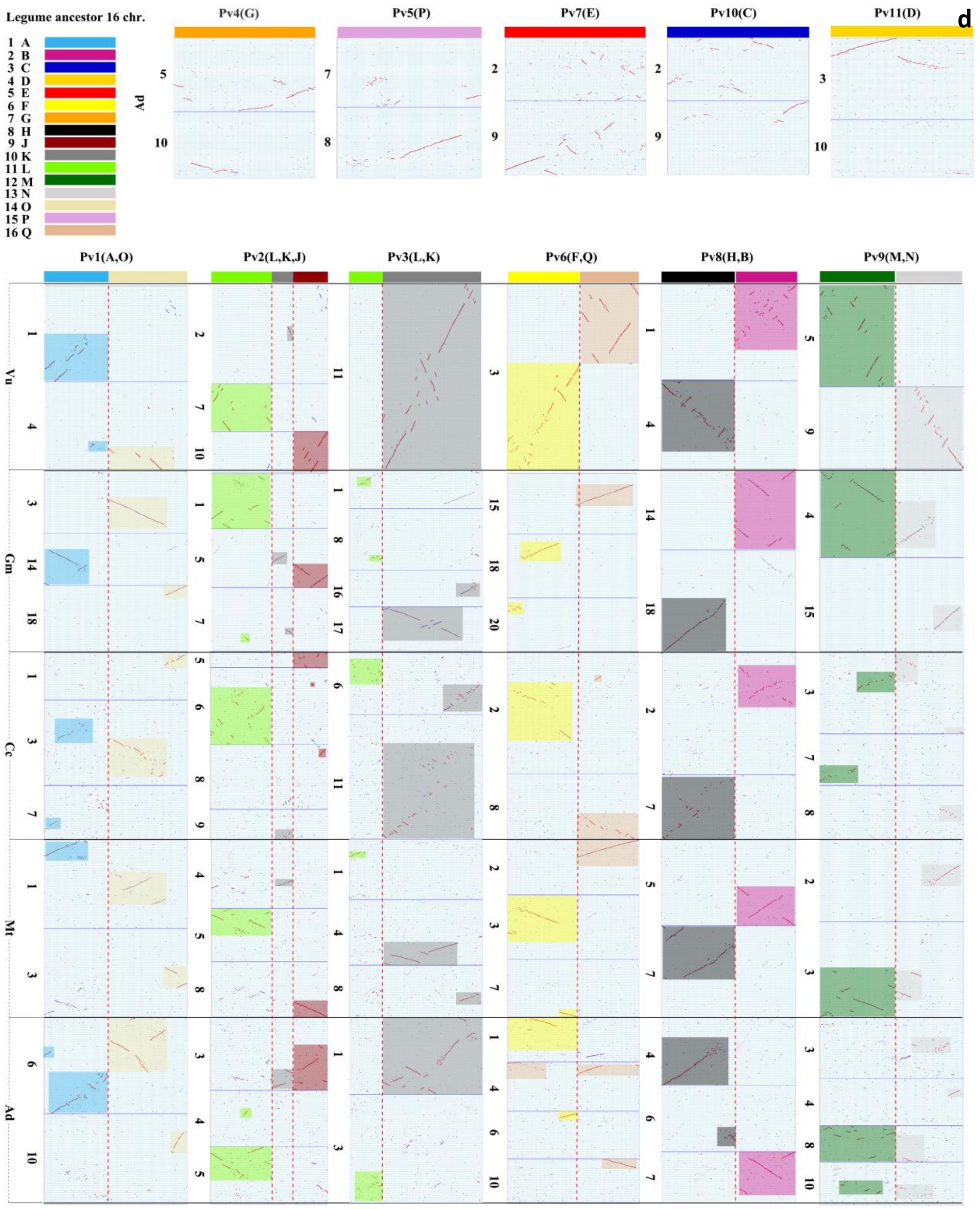

Supplementary Figure 9d. Dotplot between legume genomes.

By comparing common bean chromosomes to other legume chromosomes, 16 independent chromosomes, e.g. PV4 or G, or chromosomal blocks (A and O in Pv1) were identified.

e

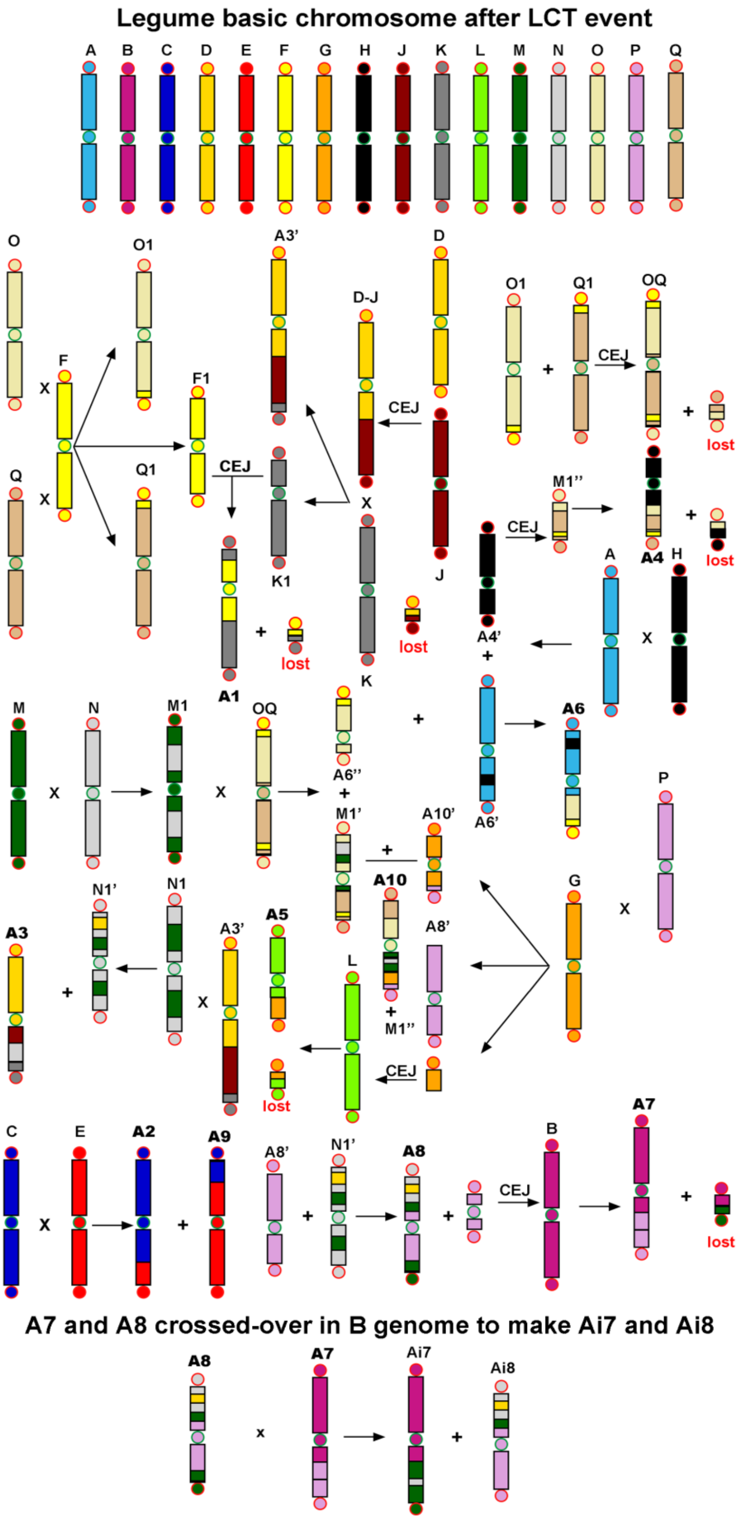

Supplementary Figure 9e. The inferred process of production of peanut chromosomes, each of which is shown in a red rectangle. CEJ: chromosome end-end joining; X: a crossing-over.

a

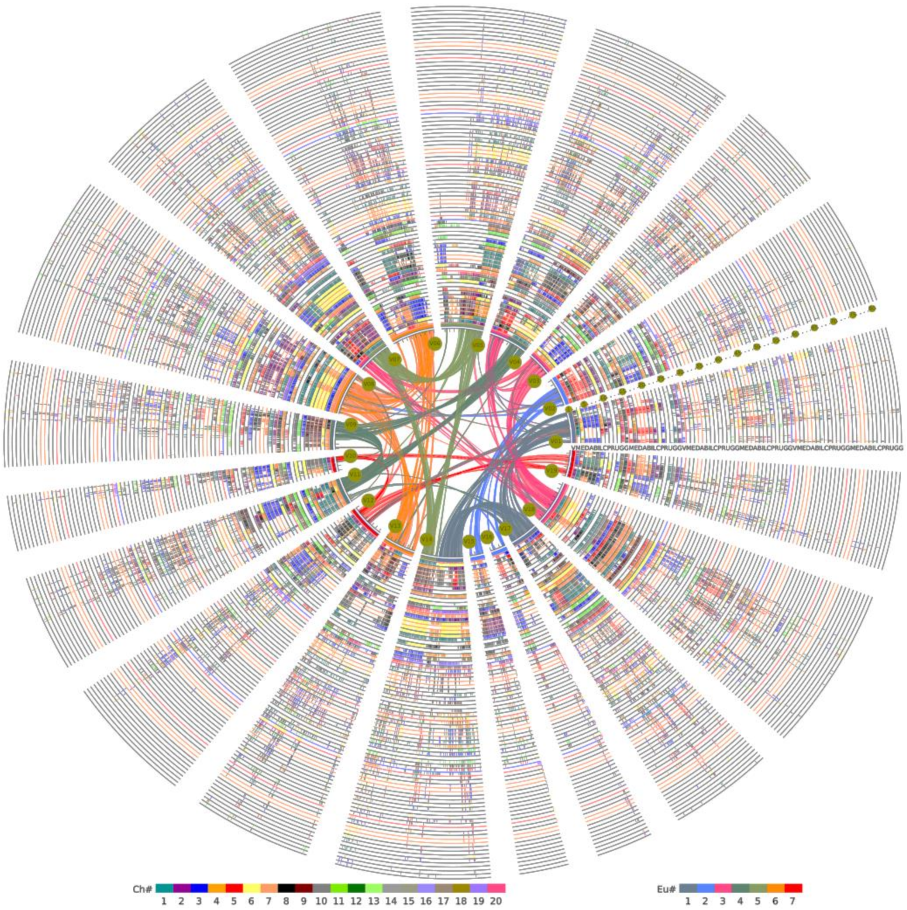
Ch#
1 2 3 4 5 6 7 8 9 10 11 12 13 14 15 16 17 18 19 20
Eu#
1 2 3 4 5 6 7

b

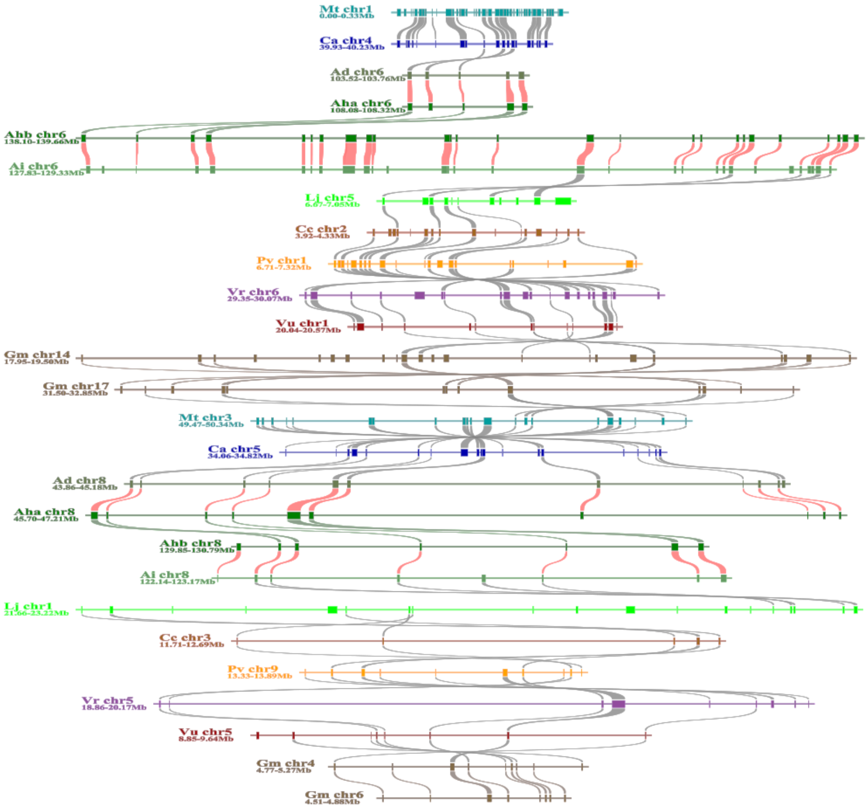
Mt chr1
0.00-0.33Mb
Ca chr4
39.93-40.23Mb
Ad chr6
103.52-103.76Mb
Aha chr6
108.08-108.32Mb
Ahb chr6
138.10-139.66Mb
Ai chr6
127.83-129.33Mb
Lj chr5
6.67-7.05Mb
Cc chr2
3.92-4.33Mb
Pv chr1
6.71-7.32Mb
Vr chr6
29.35-30.07Mb
Vu chr1
20.04-20.57Mb
Gm chr14
17.95-19.50Mb
Gm chr17
31.50-32.85Mb
Mt chr3
49.47-50.34Mb
Ca chr5
34.06-34.82Mb
Ad chr8
43.86-45.18Mb
Aha chr8
45.70-47.21Mb
Ahb chr8
129.85-130.79Mb
Ai chr8
122.14-123.17Mb
Lj chr1
21.66-23.22Mb
Cc chr3
11.71-12.69Mb
Pv chr9
13.33-13.89Mb
Vr chr5
18.86-20.17Mb
Vu chr5
8.85-9.64Mb
Gm chr4
4.77-5.27Mb
Gm chr6
4.51-4.88Mb

Supplementary Figure 10. Circular and local alignment with grape as reference. (a) Global alignment. Genomic paralogy, orthology, and outparalogy information within and among 10 legumes, with same name abbreviations as in Fig. 2, are displayed in 69 circles, each corresponding to an extant gene in Fig. 2b; the curved lines within the inner circle are formed by 19 grape chromosomes color-coded to correspond to the 7 ancestral chromosomes before the ECH. The short lines forming the innermost grape chromosome circles represent predicted genes, which have 2 sets of paralogous regions, forming another two circles. Each of the three sets of grape paralogous chromosomal regions had 2 orthologous copies in a legume with exception of soybean, which had 4 copies. The resulting 69 circles were marked according to species by a capital letter, as defined in Fig. 2. Each circle has an underline colored as to its source plant corresponding to the color scheme and each circle is formed by short vertical lines that denote homologous genes, colored as to chromosome number in their respective source plant as shown in the inset color scheme. Abbreviations: *V. vinifera* (V), *M. truncatula* (M), *C. arietium* (E), *A. duranensis* (D), *A. hypogaea* A (A), *A. hypogaea* B (B), *A. ipaensis* (I), *L. japonicus* (L), *C. cajan* (C), *P. vulgaris* (P), *V. radiata* (R), *V. angularis* (U), and *G. max* (G). (**b)** Local alignment with grape as the reference. Chromosome numbers are shown after the names of plants, along with locations on chromosomes. Genes are shown by rectangles with small arrows indicating their transcriptional direction. Homologous genes between neighboring chromosomal regions are linked with curvy lines. Abbreviations: *V. vinifera* (Vv), *M. truncatula* (Mt), *C. arietinum* (Ca), *A. duranensis* (Ad), *A. hypogaea* A (Aha), *A. hypogaea* B (Ahb), *A. ipaensis* (Ai), *L. japonicus* (Lj), *C. cajan* (Cc), *P. vulgaris* (Pv), *V. radiata* (Vr), *V. angularis* (Va), and *G. max* (Gm).

a

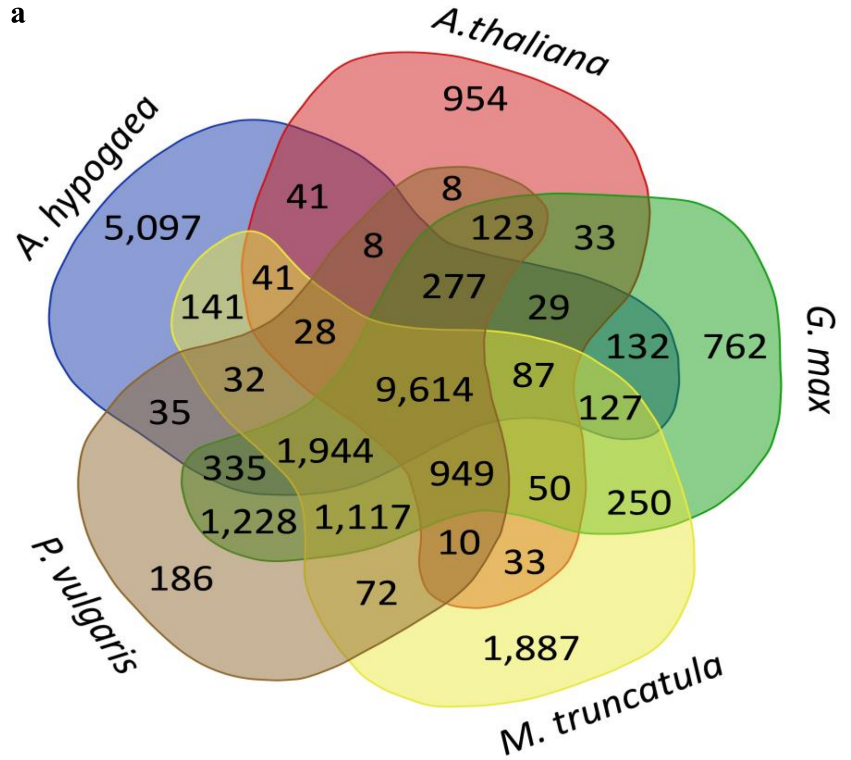

b

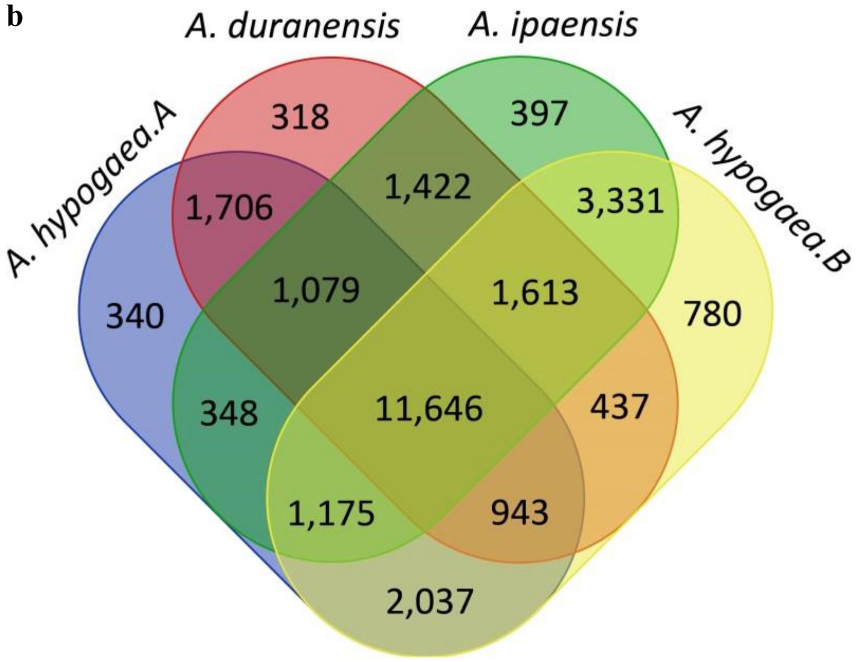

Supplementary Figure 11. Venn diagram showing the orthologous genes between peanut and other legume species.

The number of orthologous gene clusters in peanut and other legume species as well as *A. thaliana* (**a** and **b**). The numbers in the diagram representing the number of protein-coding gene clusters shared between or distinct to the indicated species.

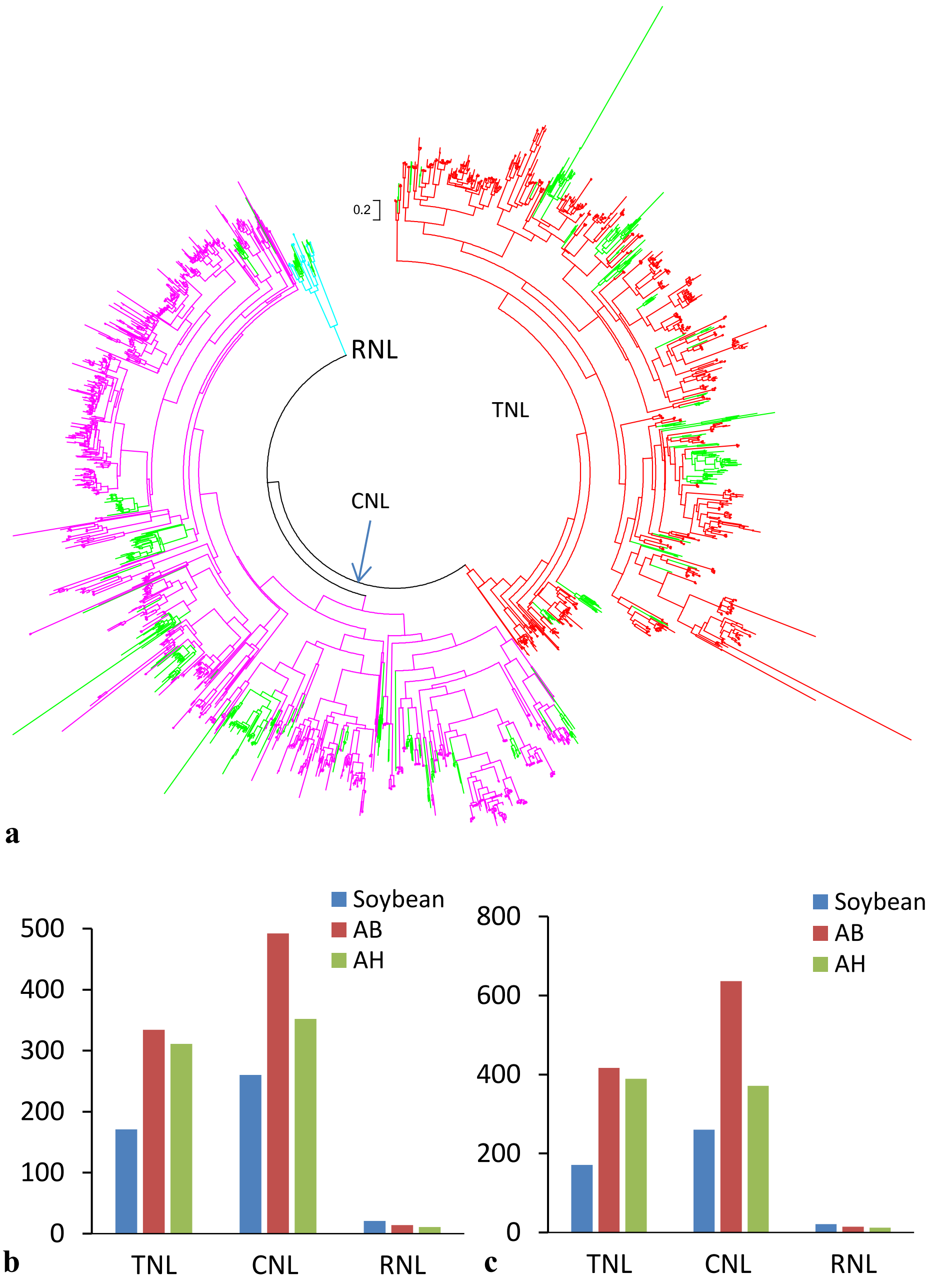

Supplementary Figure 12. Phylogenetic analysis and statistics of NB-LRR resistance genes. (a) Phylogenetic analysis divided NBS genes of peanut and soybean into three categories, TNL (TIR-NBS-LRR), CNL (CC-NBS-LRR) and RNL (RPW8-NBS-LRR). The red, purple, and cyan colors indicate TNL, CNL and RNL, respectively. The green color indicate soybean R gene. (**b)** Diagram characterizing the number of the three categories of R genes in soybean, two diploids peanut together (AB), and cultivated peanut (AH). The number of CNL genes **(b)** or domains **(c)** in both *A. duranensis* and *A. ipaensis* together was much more than that in cultivated peanut, *A. hypogaea,* due to loss genes in cultivated peanut. While the number of TNL between the wild and cultivated peanut species were comparable, indicating the adaptability of TNL to cultivated peanut.

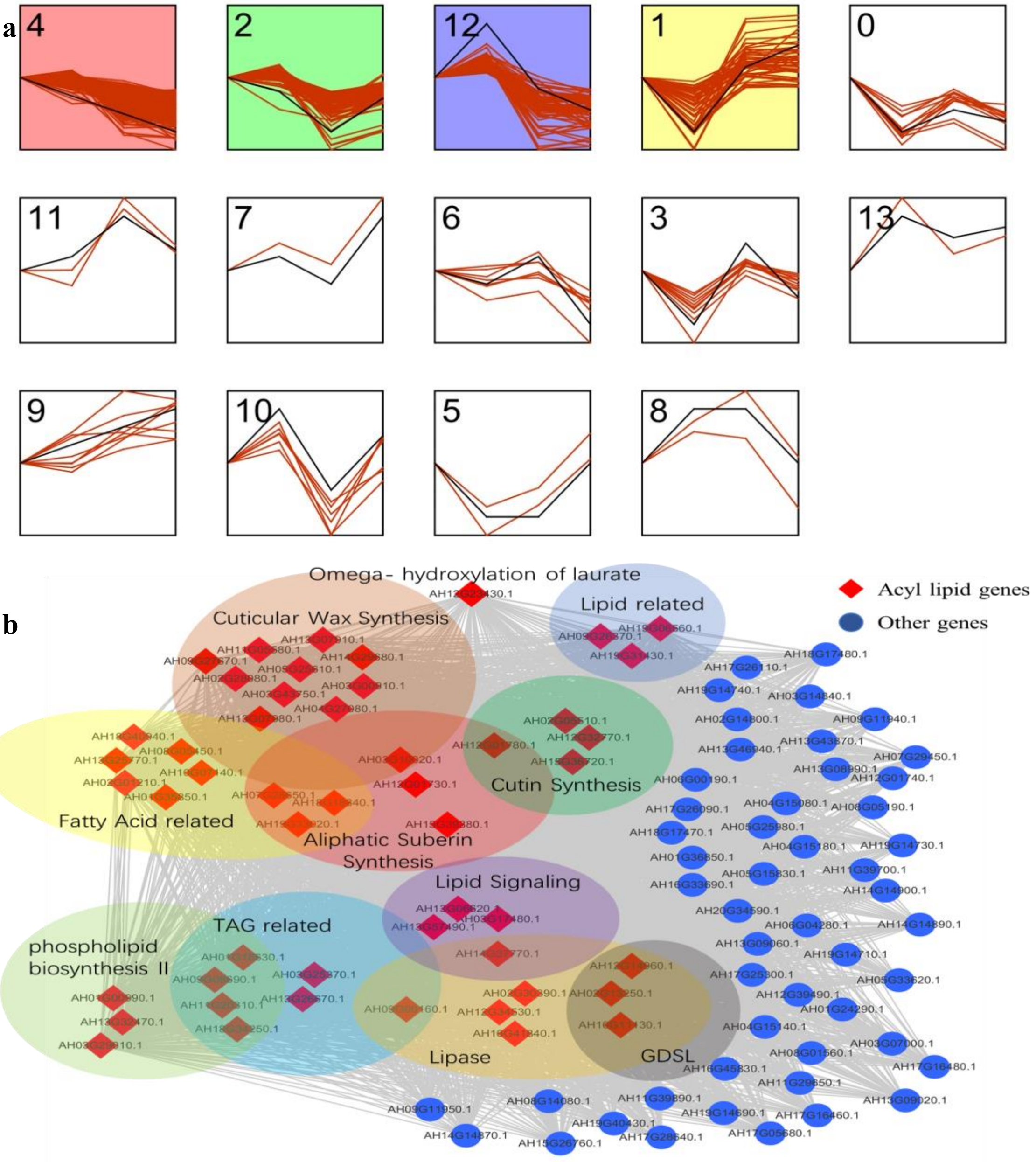

Supplementary Figure 13. Expression patterns and networks of acyl-lipid orthologue gene in developing embryos. (a) Expression patterns of cultivated peanut acyl-lipid genes during embryo growth. The number on each graph indicates the expression profile code; red curves indicate the expression value of assigned genes; black curves indicate the model pattern for each cluster; colored graphs indicate that the cluster has a statistically significant number of genes assigned. **(b)** Sub-network of co-expressed genes of orthologous acyl-lipid genes in the tan module. Red rhombus in the shadowed circles indicates the acyl-lipid genes and blue circular indicates other genes.

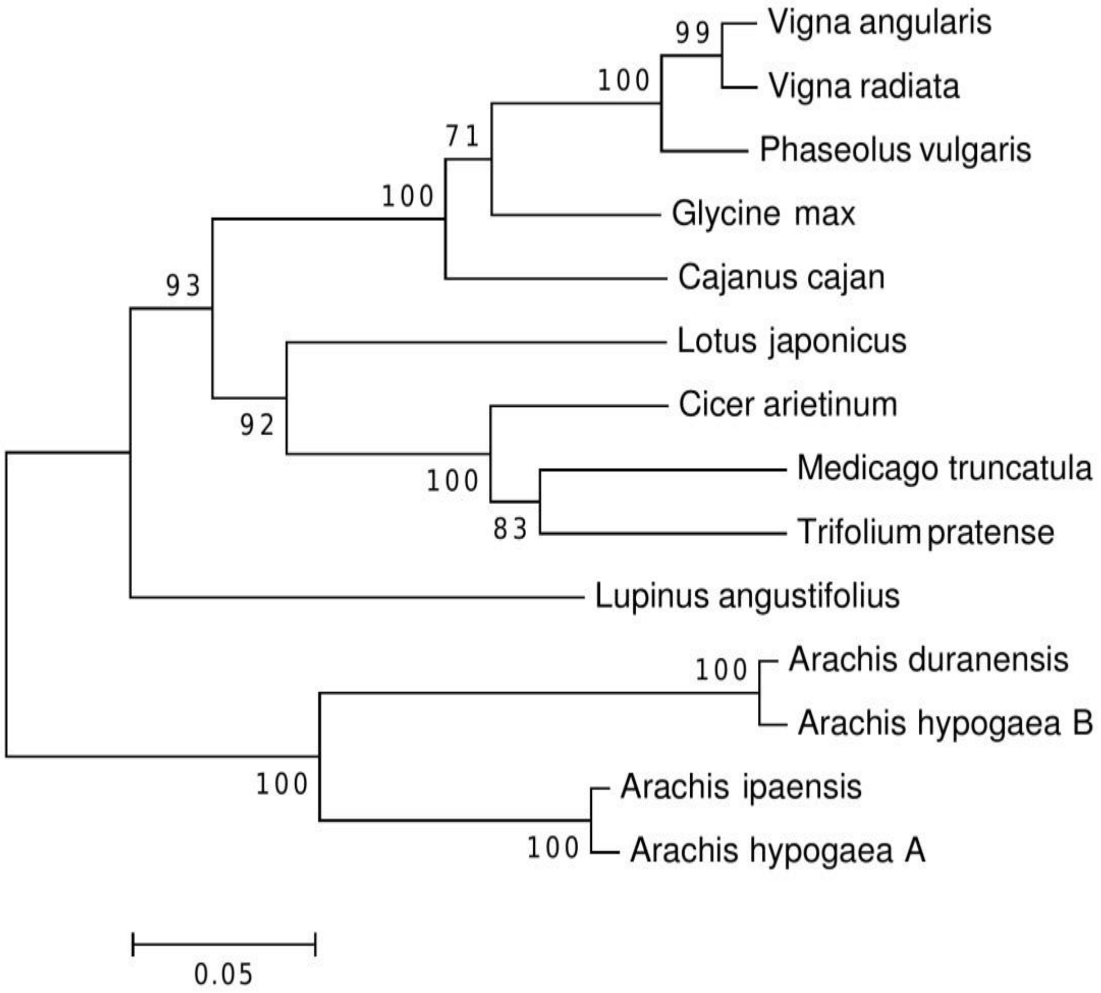

Supplementary Figure 14. Phylogenetic tree and coexpression network analysis of orthologue nodulation genes. A phylogenetic tree for legume species based on concatenated sequences of symbiosis signaling pathway genes *SYMRK*, *CCaMK*, and *NSP1*. Protein sequences were aligned using ClustalW in MEGA6 software. The tree was constructed based on the JTT model. The percentages of 1000 bootstrap replications were at the branch-points. The scale bar represents the branch length measured in the number of substitutions per site.

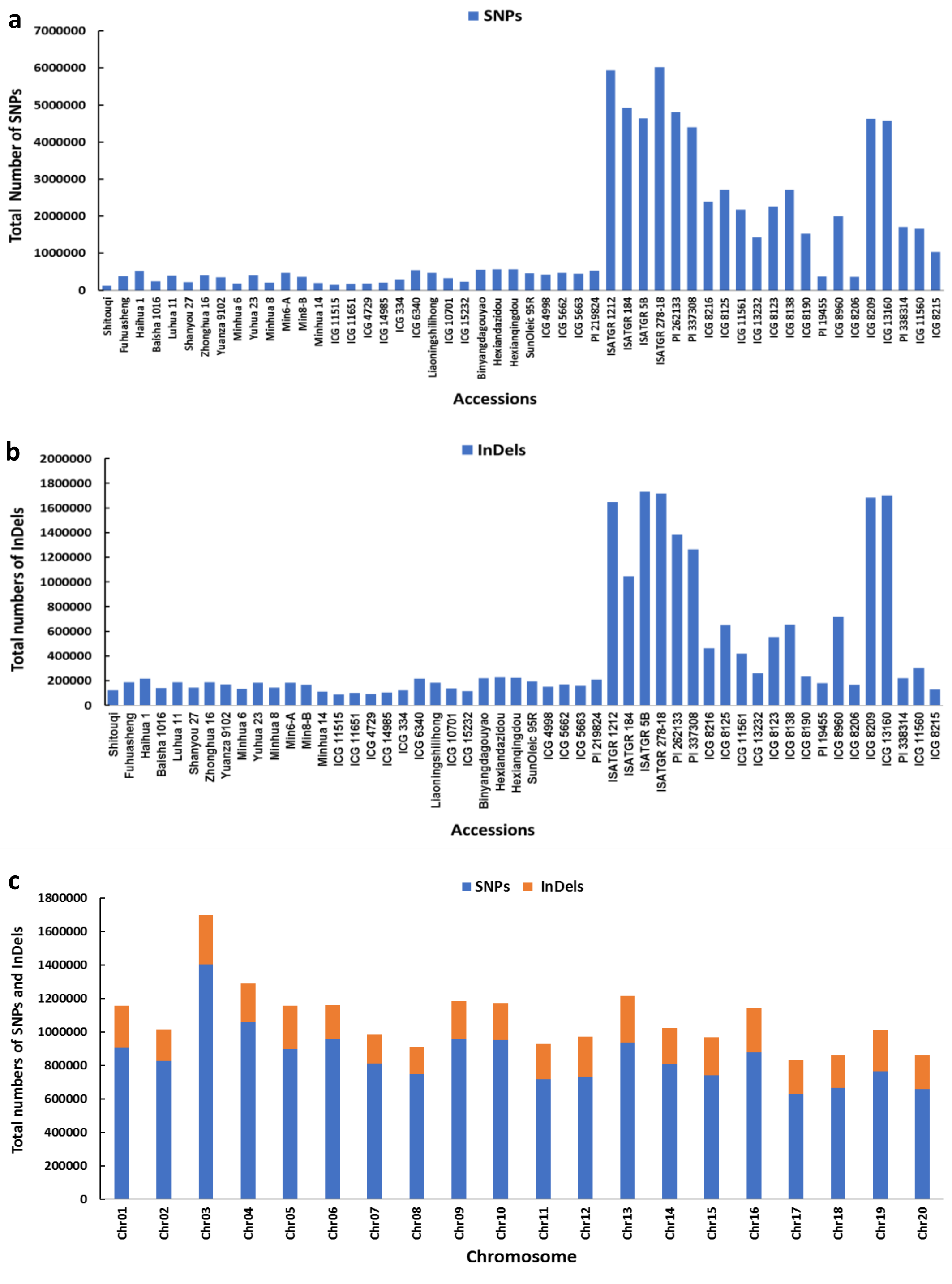

Supplementary Figure 15. Frequency of SNPs and InDels among accessions of resequencing panel and chromosome.

(a) Distribution of SNPs and InDels in each chromosome. The figure shows high number of SNPs **(b)** and InDels **(c)** in synthetic tetraploid peanuts (ISATGR 5B, ISATGR 1212, ISATGR 278-18 and ISATGR 184) as compared to domesticated and wild tetraploid. Among diploid species, *A. stenosperma* (PI 337308 and ICG 8125) and *A. batizocoi* (ICG 8209 and ICG 13160) are more diverse and can play an important role in diversification of the cultivated gene pool.

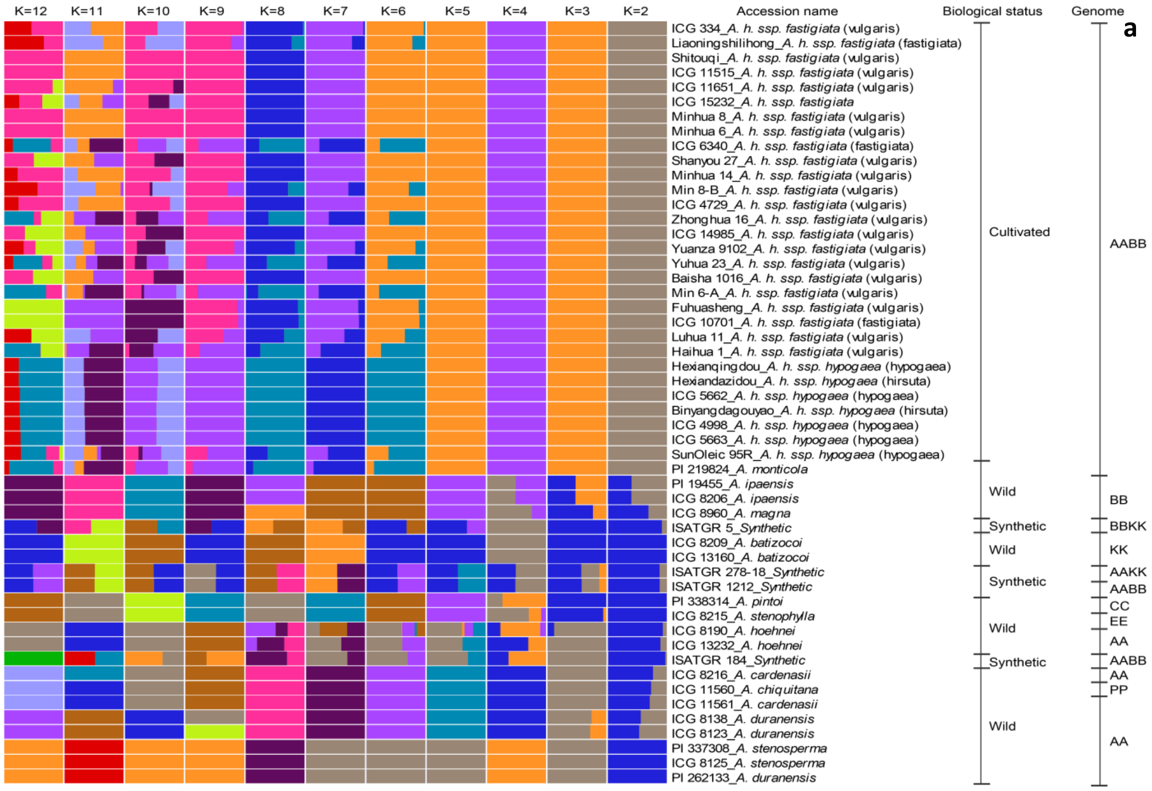

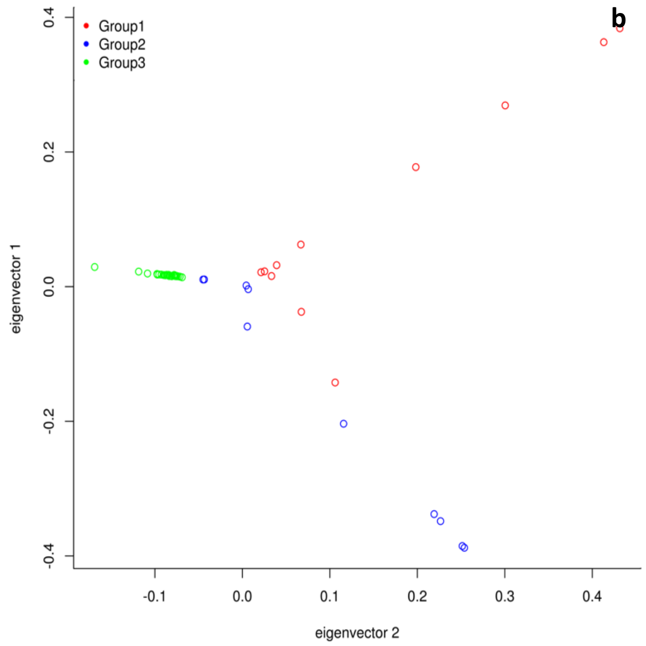

c

Weir and Cockerham mean Fst estimate			
	Group1	Group2	Group3
Group1	NA	0.16849	0.47669
Group2		NA	0.57584
Group3			NA

Weir and Cockerham weighted Fst estimate			
	Group1	Group2	Group3
Group1	NA	0.33513	0.76094
Group2		NA	0.79683
Group3			NA

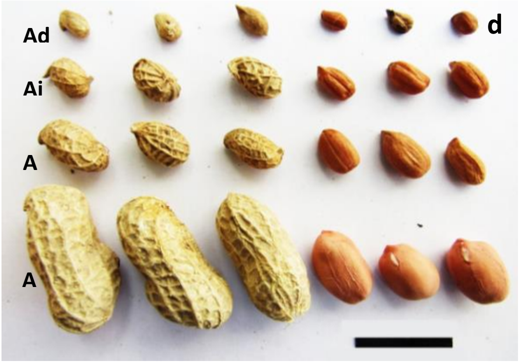

Supplementary Figure 16 Grouping and genetic relationship among 52 diverse peanut accessions and the phenotype evolution of peanut seed size. The grouping and genetic relationship for 52 accessions was analyzed using SNPhylo (Figure 3b), admixture **(a)** and principal component analysis (PCA) **(b)**. The grouping pattern observed through phylogenetic analysis was in agreement with the pattern admixture (k=3) and PCA analysis. The higher Fst value between Group1 and Group3 (0.76094) and between Group2 and Group3 (0.79683) confirm the higher genetic distance and low levels of genome exchange which is quite obvious due to lack of cross-ability because of ploidy difference **(c)**. Most importantly the synthetics with AABB genome have clustered with B-genome accessions showing higher similarity and genome dominance in these synthetics. **(d)** Pod and seed morphology evolution of *A. duranensis* (Ad), *A. ipaensis* (Ai), *A. monticol*a (Am), *A. hypogaea* (Ah). Bar indicates 1 cm.

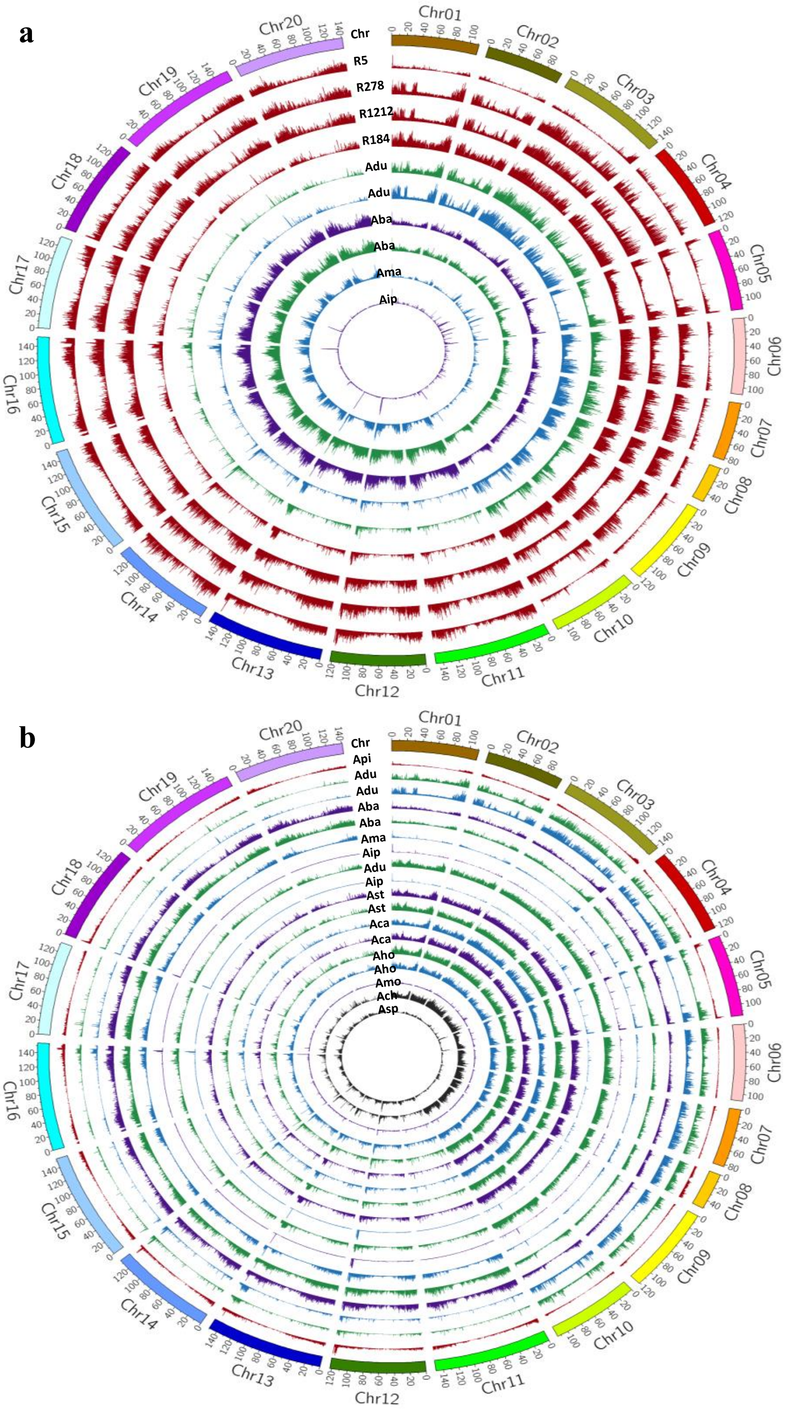
a
Chr
R5
R278
R1212
R184
Adu
Adu
Aba
Aba
Ama
Aip
Chr01
Chr02
Chr03
Chr04
Chr05
Chr06
Chr07
Chr08
Chr09
Chr10
Chr11
Chr12
Chr13
Chr14
Chr15
Chr16
Chr17
Chr18
Chr19
Chr20
b
Chr
Api
Adu
Adu
Aba
Aba
Ama
Aip
Adu
Aip
Ast
Ast
Aca
Aca
Aho
Aho
Amo
Ach
Asp
Chr01
Chr02
Chr03
Chr04
Chr05
Chr06
Chr07
Chr08
Chr09
Chr10
Chr11
Chr12
Chr13
Chr14
Chr15
Chr16
Chr17
Chr18
Chr19
Chr20

Supplementary Figure 17. Distribution patterns of SNPs of wild peanut in the reference genome. (a) Frequency distribution of SNPs in synthetics (dark red), ISATGR5 {ICG8960 (Ama) × ICG8206 (Aba)}, ISATGR278 {ICG8138 (Adu) × ICG13160 (Aba)}, ISATGR1212 {ICG812 (Adu) × ICG8206 (Aip)}, ISATGR184 {ICG8206 (Aip) × ICG8123 (Adu)}, and in the parents, ICG8123 (Adu), ICG8138 (Adu), ICG8209 (Aba), ICG13160 (Aba), ICG8960 (Ama), ICG8206 (Aip), and listed from outside to inside as the above order shown, in a 0.1 Mb window size. (**b)** Frequency distribution of SNPs in 18 wild species in the same bin. From outside to inside are diploids namely PI338314 (Api), ICG8123 (Adu), ICG8138 (Adu), ICG8209 (Aba), ICG13160 (Aba), ICG8960 (Ama), ICG8206 (Aip), PI262133 (Adu), PI19455 (Aip), PI337308 (Ast), ICG8125 (Ast), ICG8216 (Aca), ICG11561 (Aca) ICG13232 (Aho), ICG8190 (Aho), PI219824 (Amo), ICG11560 (Ach), and ICG8215 (Asp). The wild tetraploid A. monticola (Amo) showed less and comparative even SNP density distribution along each chromosome of A and B subgenomes. All accessions with A genomes such as three *A. duranensis* (Adu), two *A. stenosperma* (Ast), two A. *cardinasii* (Aca) and two *A. hoehnei* (Aho), including *A. chiquitana* (Ach, PrPr genome), all showed higher SNP density distributed on A subgenome than on B subgenome, with Adu contained less SNP density on B subgenome. Accessions with B subgenome such as two *A. ipaensis* (Aip), one *A. magna (Ama*) and accessions of two *A. batizocoi* (Aba; KK genome with over 75% reads mapped on B subgenome) showed higher SNP density on B subgenome than on A subgenome, except two Aip which showed the least SNP density on B subgenome among all accessions. Api (*A. pintoi, with CC genome*) and Asp (*A. stenophylla,* with EE gnomes), showed specific distribution patterns of SNP density on both A and B subgenomes with more SNPs mapped on B subgenome and also with a low rate of mapped reads (Supplementary Table 11). This indicated that the Api and Asp far diverged from the reference genome and relative to both A and B subgenomes.

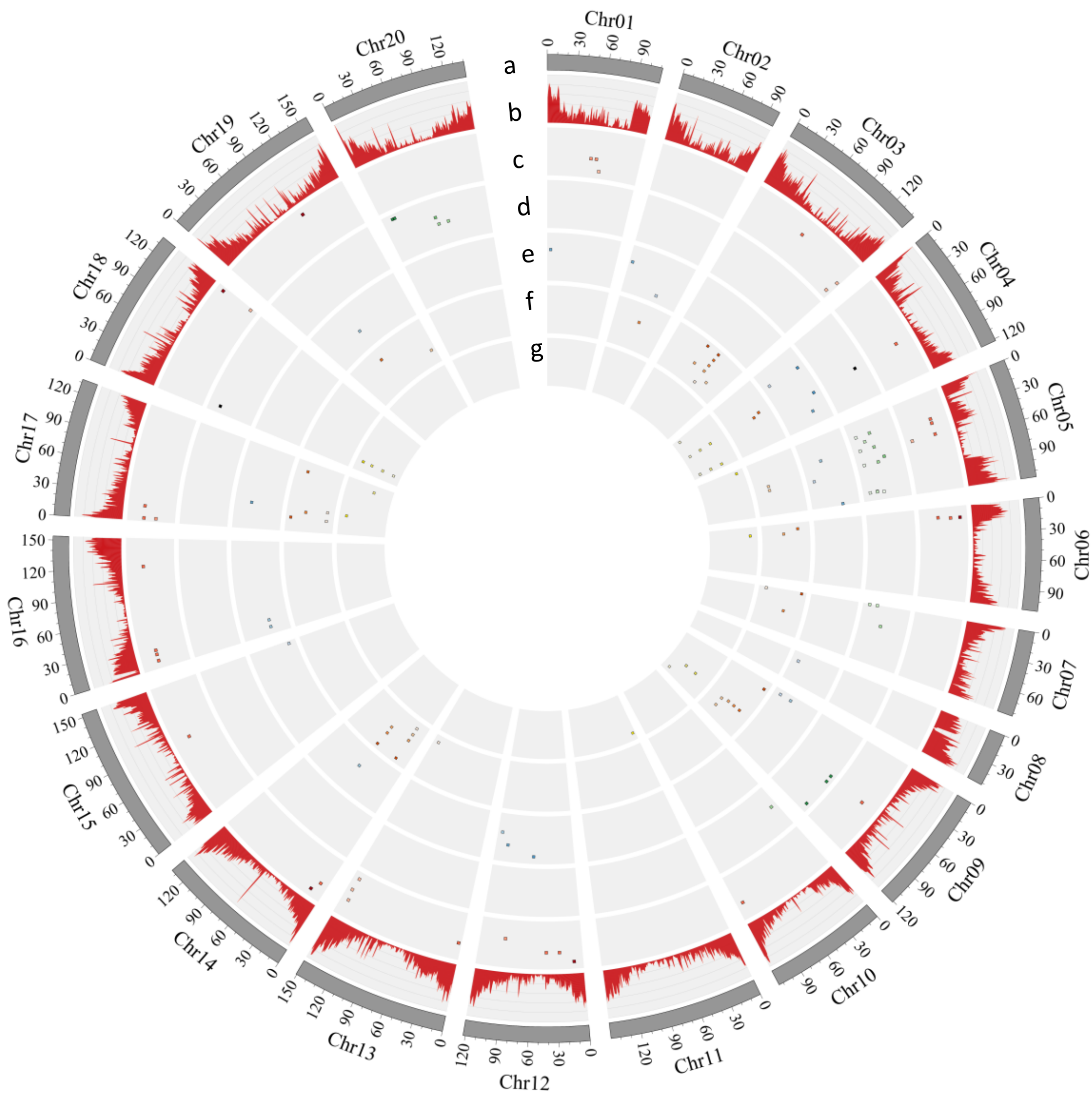

Supplementary Figure 18. The peanut genome assembly allows integration of genetics data. **(a)** Circular representation of the pseudomolecules (gray), (**b)** Density of genes distribution (red), (**c**) QTLs for 8 resistance traits. The color of red from light to dark indicating the traits rust, TSWV, later leaf spot and leaf spot disease, leaf spots, bacterial wilt, *Aspergillus flavus*, thrips resistance. (**d**) Ten economic traits of peanut i.e., the colors of black from light to dark indicates the traits of pod length, pod wide, seed length, seed wide, 100 pod weight, 100 seed weight, 10 seed weight, pod mass per plant. (**e**) QTLs for 12 plant growth traits. The color of blue indicates pod number of the stem-related, the branch related, the leaf related and the other related traits. (**f**) QTLs for 6 fatty acid component-content traits. the color of red from light to dark indicates the arachidic acid, behenic acid, gadoleic acid, lignoceric acid, palmitic acid, stearic acid. (**g**) QTLs for 4 oil quality traits. The color of yellow indicates the traits of oleic acid, linoleic acid, oleic / linoleic acid ratio, oil content. Dark indicating the traits of rust resistance, TSWV, early leaf spot, late leaf spot.

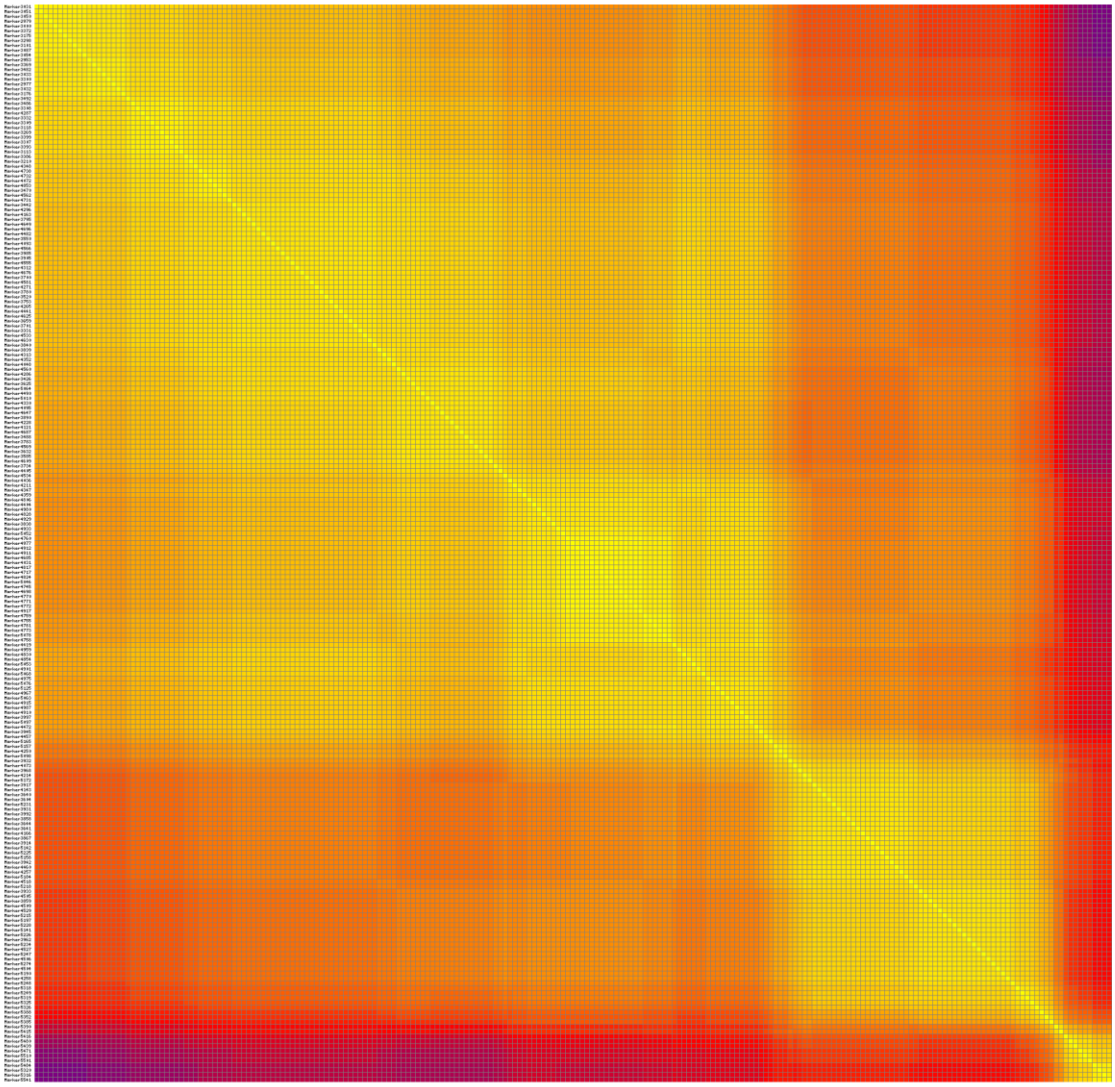

Supplementary Figure 19. Heatmap of genetic linkage map A02.

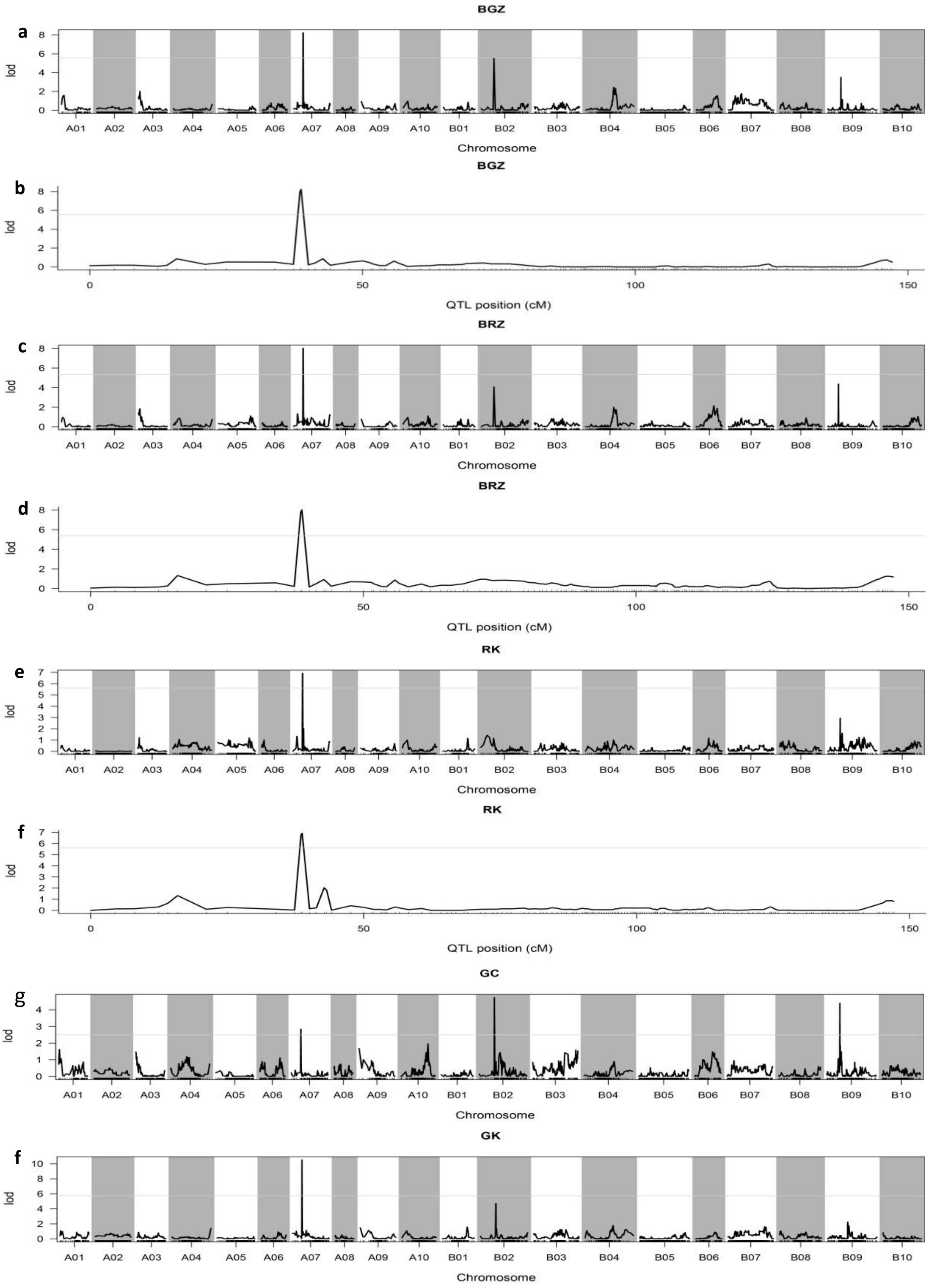

Supplementary Figure 20. Seed size QTL mapping result.

a-b, c-d, e-f, g and h are 100 pods weight, 100 kernels weight, kernel width, pod long and pod width, respectively. All the traits have a QTL localized on chromosome A07 at the same position.

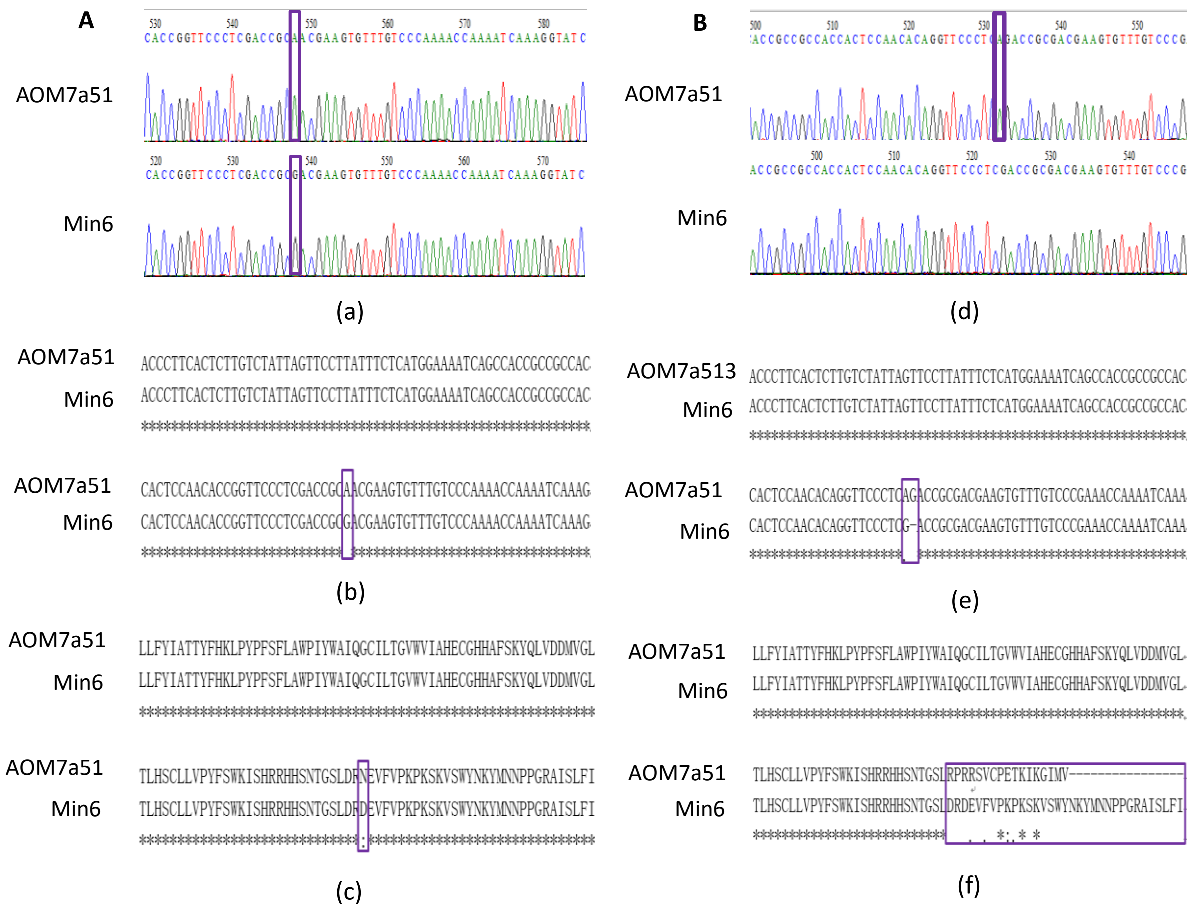

Supplementary Figure 21. Nucleotide and amino acids sequences comparison of mutated *ahFAD2A* and *ahFAD2B* genes between mutant line AOM7a513 and host mock Min6. An SNP at G448A (G>A) for *ahFAD2A* (a, b) mutated into asparaginate (N) (c) which leads to amino acid aspartic acid (d). A single-base adenine insertion at 442 bp for *ahFAD2B* (d, e) results in a frameshift (f).

Supplementary Tables

Supplementary Table 1 Summary table of integrated linkage map.

LG	Sub-genome	Markers	Contigs	Length of LG (cM)	Physical length (bp)	Average Nt/cM	Marker density (bp/loci)
A01	A	347	251	132.0056782	111624253	667,571	321,684
A02	A	560	333	150.1216913	99791824	594,331	178,200
A03	A	531	359	156.8284133	143206517	692,193	269,692
A04	A	656	328	157.4816629	122064722	606,765	186,074
A05	A	627	359	153.0999522	116166884	608,940	185,274
A06	A	307	324	129.4685947	109264827	686,164	355,911
A07	A	470	254	170.2305958	83117376	462,652	176,845
A08	A	273	96	126.2441775	51955169	490,746	190,312
A09	A	449	253	143.1300871	121058954	605,383	269,619
A10	A	606	383	181.2101415	114519869	609,021	188,977
B01	B	584	315	154.9715223	149379573	693,527	255,787
B02	B	912	281	191.513136	122213787	637,361	134,006
B03	B	784	305	182.0261796	150766190	630,032	192,304
B04	B	1106	325	198.9559338	135085854	568,538	122,139
B05	B	1224	380	203.4754189	153119099	599,488	125,097
B06	B	334	380	128.9386899	153789061	768,846	460,446
B07	B	1289	327	184.1041034	132998879	542,072	103,180
B08	B	1215	378	170.0462605	136776503	600,066	112,573
B09	B	1482	372	198.2153741	159154999	568,189	107,392
B10	B	863	286	152.3774658	140659116	569,528	162,989
Total		14,619	6,289	3,264	2,506,713,456	610,071	171,470

The contig column shows how many contigs that all markers on the linkage group can be mapped with. Marker density is calculated as physical length divided by markers.

Supplementary Table 2a Statistics of gene annotation for *A. hypogaea, A. duranensis*, and *A. ipaensis.*

	A. duranensis-NG	*A. ipaensis-NG*	*A. hypogaea*
Number of genes	36,734	41,840	83,709
Length of gene regions	123,534,791	136,789,374	424,985,486
Length of mRNA regions	52,182,755	56,816,416	133,056,128
Length of the coding regions	40,433,739	43,877,079	101,482,701
Length of intron regions	71,352,036	79,972,958	291,929,358
Length of largest gene	68,350	99,555	513,003
Mean length of genes	3362.96	3269.34	5076.94
Mean length of mRNAs	1420.56	1357.94	1589.51
Length of the largest CDS	16,629	19,380	46,410
Mean length of CDSs	1100.72	1048.7	1212.33
Number of exons per gene	5.81	5.66	6.82
Number of single-exon genes	1,817	1,923	29
Length of the largest intron	67,497	96,189	363,466
Mean length of introns	403.45	410.43	599.66

Note: all units of length are base pair (bp). -NG: results from the literature (doi:10.1038/ng.3517)

Supplementary Table 2b Comparison of protein coding genes in different plant species.

**Species**	**Total number of gene**	**Average gene length(bp)**	**Average CDS length(bp)**	**Average exons number per gene**	**Average exon length(bp)**	**Average intron length(bp)**
A. hypogaea	83,709	5,103,37	1,589.77	6.82	233.16	603.89
A. duranensis	36,734	3,362.96	1,100.72	5.38	204.59	399.95
A. ipaensis	41,840	3,269.34	1,048.69	5.23	200.59	406.57
B. napus	101,040	1,953.13	1,001.16	4.91	204.06	195.48
G. max	56,044	4,671.51	1,275.06	9.38	214.91	502.11
M. truncatula	50,894	3,064.99	1,059.51	5.60	231.70	437.64
P. vulgaris	27,433	3,911.92	1,258.76	5.24	240.11	492.02
V. vinifera	26,346	6,454.02	1,137.11	5.95	191.10	969.55

Supplementary Table 2c Statistics of RNA sequence assisted assembly (by Illumina and Isoform techniques).

Species	Gene Number	RNA-seq transcripts		Tifrunner (25177+27103)		IsoSeq		RNAseq Reads	
		Number	%	Number	%	Number	%	Number	%
A. hypogaea	83,709	64,179	76.67	33,904	40.49	33,807	40.37	64,179	76.67
A. duranensis	36,374	17,359	47.72	16,130	44.34	-	-	21,185	58.24
A. ipaensis	41,480	18,157	43.77	17,697	42.66	-	-	22,402	54.01

Supplementary Table 2d Functional annotation of predicted genes in *A. hypogaea.*

Type	Number	Percent (%)
Total Gene	83,744	100
Kegg	20,370	24.32
InterproScan	56,241	67.16
Go	33,711	40.25
SwissProt	48,523	57.94
TrEMBL	43,071	51.43
Annotated	64,105	76.55
Total Gene	83,709	100

Supplementary Table 3a Summary of predicted non-coding RNAs in peanut.

Type		Copy number	Average Length (bp)	Total Length (bp)	% of Genome
miRNA		480	127.62	60494	0.002382
tRNA		4723	73.06	345070	0.01359
rRNA		3107	861.06	2675311	0.105362
	18S	481	1763.83	848404	0.033413
	28S	403	3868.95	1559186	0.061406
	5.8S	299	153.69	45953	0.00181
	5S	1924	115.26	221768	0.008734
snRNA		30817	106.33	3276894	0.129054
	CD-box	30563	106.04	3240954	0.127639
	HACA-box	66	126.56	8353	0.000329
	Splicing	188	146.74	27587	0.001086

Supplementary Table 3b Repeat classification in *A. hypogaea, A. duranensis, and A. ipaensis*.

Repeat Class Description			*A.hypogaea*		*A.duranensis*		*A.ipaensis*	
**Repeat Types**	**Order**	**Superfamily**	**Length (bp)**	**Percent (%)**	**Length (bp)**	**Percent (%)**	**Length (bp)**	**Percent (%)**
Class I: Retrotransposon	LTR	Summary	1,623,072,933	63.94	555,993,041	59.15	816,659,305	64.97
		Caulimovirus	11,312,667	0.44	2,560,114	0.28	2,141,951	0.17
		Copia	77,519,828	3.05	34,581,437	3.68	39,804,181	3.17
		ERV1	472,208	0.02	206,460	0.02	431,521	0.03
		ERVK	12,125	0.00	145,189	0.02	9,232	0.00
		Gypsy	1,030,432,832	40.59	350,811,362	37.32	525,380,519	41.79
		Gypsy-Cigr	133,376	0.01	64,332	0.01	113,307	0.01
		Unclassified[a]	688,978,318	27.14	191,911,380	20.42	304,241,051	24.20
		Pao	14,325	0.00	7,587	0.00	644,170	0.05
	LINE	Summary	44,943,906	1.77	18,138,009	1.93	23,821,319	1.90
		L1	37,934,410	1.49	15,045,917	1.60	21,030,599	1.67
		L1-Tx1	228,211	0.01	125,065	0.01	173,734	0.01
		L2	1,039,582	0.04	136,021	0.01	192,280	0.02
		RTE-BovB	5,965,839	0.24	2,966,443	0.32	2,618,468	0.21
	SINE	Summary	612,482	0.02	75,983	0.01	126,605	0.01
		Alu	551,022	0.02	0	0.00	0	0.00
		tRNA-RTE	54,004	0.00	73,745	0.01	124,498	0.01
Class II: DNA transposon	DNA	Summary	113,888,023	4.49	40,020,963	4.26	51,404,078	4.09
		CMC-EnSpm	27,507,646	1.08	8,448,284	0.90	11,629,723	0.93
		Crypton-S	0	0.00	0	0.00	162,258	0.01
		Dada	255,406	0.01	33,849	0.00	372	0.00
		Ginger	338,871	0.01	408,552	0.04	270,890	0.02
		hAT	3,207	0.00	111,491	0.01	2,174	0.00
		hAT-Ac	14,378,710	0.57	4,854,055	0.52	4,648,612	0.37
		hAT-Charlie	3,960,886	0.16	42,815	0.00	41,853	0.00
		hAT-Tag1	11,823,830	0.47	2,870,158	0.31	4,231,692	0.34
		hAT-Tip100	6,096,090	0.24	1,859,654	0.20	2,037,759	0.16
		MULE-MuDR	51,289,021	2.02	21,763,991	2.32	29,375,344	2.34
		Unclassified	3,240,568	0.13	477,723	0.05	585,833	0.05
		DNA/P[b]	1,612	0.00	98,278	0.01	1,106	0.00
		PIF-Harbinger	4,354,773	0.17	2,241,112	0.24	2,591,692	0.20
		TcMar-Stowaway	189,183	0.01	4,262	0.00	10,078	0.00
RC (rolling-circle)		Summary	10,696,717	0.42	3,612,683	0.38	3,586,280	0.29
		Helitron	10,686,227	0.42	3,607,079	0.38	3,581,469	0.28
Simple_repeat			79,822,418	3.14	28,517,323	3.03	32,250,288	2.57
Satellite (Unclassfied)			3,736,095	0.15	836,163	0.09	1,409,761	0.11
Unknown[c]			90,643,361	3.57	42,520,332	4.52	39,561,433	3.15
Total content			1,971,141,540	77.65	689,708,893	73.37	968,814,258	77.08

Those repeats which take less than 0.01% in all the three species are not list in the table.

a: Unclassified: Repeats that have Order name but can not be attributed to any one Superfamily.

b: DNA/P: means Motif: P-1_CR, Motif: P-2_CR, and Motif: DNA-8-3_CR" in DNA transposons.

c: Unknown: Repeats that can not be attributed to any known class.

Supplementary Table 4 Summary of genome assembly and annotation of peanut.

Genome features	Measures of Index
Genome assembly	
Pacbio SMRT sequences	270.5 Gb (100x)
Number of contigs	7,232
Total size of contigs	2,538,408,906 bp
Longest contigs	8,550,813 bp
Contig N50 Length	1,509,423 bp
Contig N90 Length	342,540 bp
Contig L50 count	505
Contig L90 count	1,804
Number of scaffolds	1297
Total size of scaffolds	2,539,035,806 bp
Scaffold N50 Length	135,085,854 bp
Scaffold N90 Length	109,264,827 bp
Scaffold N50 count	9
Scaffold N90 count	17
Longest scaffold	159,154,999
GC content	36.53
Gene models	
Number of gene models	83,709
Mean gene length	5076.94 bp
Mean length of exon	233.21 bp
Mean length of intron	599.66 bp
Mean number of exons per gene	6.82
Mean gene density	33/M
Nonprotein coding genes/elements	
Number of miRNA genes	480
Mean length of miRNA genes	127.62 bp
miRNA genes share in genome	0.002382%
Number of rRNA fragments	2,808 3107
Mean length of rRNA fragments	861.06bp
rRNA fragment share in genome	0.105362%
Number of tRNA genes	4,723
Mean length of tRNA genes	73.06 bp
tRNA gene share in genome	0.01359%
Number of snRNA genes	30,817
Mean length of snRNA genes	106.33 bp
snRNA genes share in genome	0.129054%
Total transposable elements, bp	1,971,141,540bp
Percentage of transposable element in genome	77.65%

Supplementary Table 5 BUSCO analysis of Arachis genomes.

Type	Based on whole genome sequences			Based on annotated		
	A. hypogaea	***A. duranensi***	***A. ipaensis***	***A. hypogaea***	***A. duranensi***	***A. ipaensis***
Complete BUSCOs (C)	1,341(93.1%)	1,323 (91.9%)	1,314 (91.3%)	1,319 (91.6%)	1,251(86.9%)	1,218(84.5%)
Complete and single-copy BUSCOs (S)	344(23.9%)	1,204 (83.6%)	1,183 (82.2%)	341 (23.7%)	1,122(77.9%)	1,085(75.3%)
Complete and duplicated BUSCOs (D)	997(69.2%)	119 (8.3%)	131 (9.1%)	978 (67.9%)	129(9%)	133(9.2%)
Fragmented BUSCOs (F)	18(1.2%)	21 (1.5%)	30 (2.1%)	33 (2.3%)	57(4%)	78(5.4%)
Missing BUSCOs (M)	81(5.7%)	96 (6.6%)	96 (6.6%)	88 (6.1%)	132(9.1%)	144(10%)
Total BUSCO groups searched	1,440	1,440	1,440	1,440	1,440	1,440

Supplementary Table 6 Statistics of homeologs in a cultivated peanut genome.

Homeologous chromosome pairs	No. of homeolog pairs	No. of genes without homeologs	Non-homeolog gene without ortholog in ancestral diploids	No. of tandem duplicated genes	No. of dispersed duplicated genes
Chr01-Chr11	3281	464	142	330	5181
Chr02-Chr12	2371	607	134	306	3652
Chr03-Chr13	3873	849	201	380	4505
Chr04-Chr14	2253	812	144	268	2622
Chr05-Chr15	2482	691	129	233	2228
Chr06-Chr16	2115	911	150	168	1732
Chr07-Chr17	1717	485	111	179	1521
Chr08-Chr18	2004	572	135	143	1214
Chr09-Chr19	2166	560	149	224	1508
Chr10-Chr20	1946	437	105	171	1348
Total	24208	6388	2421	2402	25511
Genes with homeologs defined			30596		
No. of unanchored genes/homeologs			992		
No. of duplicated genes			27913		
Families with duplicated genes			10974		

Supplementary Table 7 Number of homologous blocks and gene pairs within or between genomes.

Homologous Blocks within and among genome	BL[a] > 4	BL > 10	BL > 20	BL > 50	ACGP[b]	LDB[c]	LDB on chromosomes
A. hypogaea_A	2067/301	646/36	302/9	0/0	6.87,17.94,33.56,0.00	49	Aha04-Aha01
A. hypogaea_B	2283/300	929/48	543/18	0/0	7.61,19.35,30.17,0.00	46	Ahb09-Ahb08
A. duranensis vs A. hypogaea_A	34266/2310	23793/352	21610/179	18611/72	14.83,67.59,120.73,258.49	1058	Ad03-Aha03
A. duranensis vs A. hypogaea_B	30398/2343	20020/386	17241/168	14274/64	12.97,51.87,102.63,223.03	990	Ad03-Ahb03
A. hypogaea_B vs A. hypogaea_A	35576/3539	19255/371	16454/158	13583/63	10.05,51.90,104.14,215.60	693	Ahb03-Aha03
A. ipaensis vs A. hypogaea_A	28845/2230	18819/341	16312/150	13597/66	12.93,55.19,108.75,206.02	691	Ai10-Aha10
B. ipaensis vs A. hypogaea_B	38417/2405	27912/404	25102/190	21439/68	15.97,69.09,132.12,315.28	1553	Ai07-Ahb07
C. arietinum vs A. hypogaea_A	22479/2212	12852/399	10096/190	6333/63	10.16,32.21,53.14,100.52	351	Ca2-Aha07
C. cajan vs A. hypogaea_A	14385/1534	7961/362	4958/137	1695/23	9.38,21.99,36.19,73.70	131	Cc02-Aha07
G. max vs A. hypogaea_A	37173/2572	27042/794	21713/394	14234/148	14.45,34.06,55.11,96.18	317	Gm02-Aha07
L. japonicus vs A. hypogaea_A	14947/1210	9930/304	7954/155	5007/54	12.35,32.66,51.32,92.72	309	Lj5-Aha09
M. truncatula vs A. hypogaea_A	20118/1572	13565/402	10950/199	7176/74	12.8,33.74,55.03,96.97	344	Mt1-Aha09
P. vulgaris vs A. hypogaea_A	25454/2314	15572/486	12087/222	7628/73	11,32.04,54.45,104.49	346	Pv07-Aha09
V. radiata vs A. hypogaea_A	21078/2138	11720/404	8624/174	5001/49	9.86,29.01,49.56,102.06	352	Vr06-Aha07
V. angularis vs A. hypogaea_A	15570/1579	8896/356	6210/150	2984/36	9.86,24.99,41.40,82.89	167	Vu06-Aha09
V. vinifera vs A. hypogaea_A	15678/1557	9271/406	6105/164	2324/35	10.07,22.83,37.23,66.40	138	Vv18-Aha08
D. arietinum vs A. hypogaea_B	22521/2088	13595/413	10743/195	6830/65	10.79,32.92,55.09,105.08	337	Ca4-Ahb09
C. cajan vs A. hypogaea_B	14541/1528	8215/380	4943/134	1808/25	9.52,21.62,36.89,72.32	96	Cc02-Ahb09
G. max vs A. hypogaea_B	39904/2698	29179/803	23818/401	16353/158	14.79,36.34,59.40,103.50	367	Gm12-Ahb03
L. japonicus vs A. hypogaea_B	15937/1242	10895/332	8668/160	5494/57	12.83,32.82,54.17,96.39	362	Lj3-Ahb03
M. truncatula vs A. hypogaea_B	21525/1659	14540/407	12094/218	8139/83	12.97,35.72,55.48,98.06	357	Mt4-Ahb03
P. vulgaris vs A. hypogaea_B	26980/2414	16777/508	13339/239	8637/80	11.18,33.03,55.81,107.96	441	Pv11-Ahb03
V. radiata vs A. hypogaea_B	21738/2075	12977/435	9732/188	5944/62	10.48,29.83,51.77,95.87	392	Vr02-Ahb03
V. angularis vs A. hypogaea_B	16187/1603	9572/389	6578/157	2791/32	10.10,24.61,41.90,87.22	182	Vu11-Ahb01
V. vinifera vs A. hypogaea_B	16262/1541	9899/418	6454/163	2741/39	10.55,23.68,39.60,70.28	139	Vv18-Ahb07

[a]BL: block_length; [b]ACGP: average colinear gene pairs respectively per block; [c]LDB: number of colinear gene pairs residing in longest duplicated block

Supplementary Table 8 Statistics of OrthoMCL analysis in peanut and related species.

	A. hypogaea	*A. duranensis*	*A. ipaensis*	*A. hypogaea*	*A. thaliana*	*G. max*	*P. vulgaris*	*M. truncatula*
Protein coding genes	83,709	36,734	41,840	83,709	27,416	56,044	27,433	50,894
Genes in clusters	62,794	28,532	31,535	72,036	22,929	44,785	25,034	37,375
% of genes in clusters	75.01	77.67	75.37	86.06	83.63	79.91	91.26	73.44
Clusters	25,153	19,003	20,831	17,986	12,376	17,114	15,992	16,460
% of clusters	91.72	69.29	75.96	70.21	48.31	66.80	62.42	64.25
Species-specific clusters	3,044	332	413	5,054	929	730	181	1,866
Genes in specific clusters	10,339	898	1068	28,393	3,554	2140	587	9,382
% of genes in specific clusters	12.35	2.44	2.55	33.92	12.96	3.82	2.14	18.43

Supplementary Table 9 Orthologous acyl-lipid genes in cultivated peanut, its progenitors and major oilseed crops.

Pathways	Species*					
	Ahyp	Aradu	Araip	Glym	Equi	Bnap
Plastidial Fatty Acid Synthesis	113	71	59	120	112	242
TAG Synthesis	100	50	51	111	91	297
Lipid Signaling	290	168	159	315	252	673
Mitochondrial Acyl-lipids Synthesis	36	23	23	43	55	99
Fatty Acid Elongation and Wax and Cutin Synthesis	401	221	225	504	277	1030
Degradation of TAG and long Fatty acid	93	56	55	108	95	298
Galactolipid degradation	16	8	6	19	12	27
Structure lipids Synthesis	192	110	107	175	209	464
Other lipid Synthesis	255	156	169	304	209	618
Lipid Trafficking	19	9	10	21	22	27
Metabolism of acyl-lipid	671	402	402	860	564	1801
Miscellaneous: lipid related	104	61	58	117	96	311
Total functional orthologous gene number**	2290	1335	1324	2697	1994	5887
Total orthologous gene number***	1944	1137	1143	1876	1409	4201

*Ahyp, Glym, Aradu, Araip, Equi and Bnap represent *A. hypogaea, G. max, A. duranensis, A. ipaensis, Elaeis guineensis* and *Brassica napus* respectively.

**Total functional gene number is the summary of genes annotated with a single pathway

***Total orthologous gene number is the total number of orthologs aligned to *A. thaliana* oil genes, they might be annotated with different pathway

Supplementary Table 10 Orthologous acyl-lipid genes in cultivated peanut that were duplicated more than once from single copy diploid orthologs and their annotation.

Cultivated peanut genes	*A. thaliana* pathway	*G. max* pathway
AH03G46800.1	Plastidial Fatty Acid Synthesis	
AH05G11330.1	Plastidial Fatty Acid Synthesis	
AH13G46440.1	Plastidial Fatty Acid Synthesis	
AH15G13100.1	Plastidial Fatty Acid Synthesis	
AH04G04320.1	Lipid Signaling	
AH14G05290.1	Lipid Signaling	
AH14G05720.1	Lipid Signaling	
AH09G26850.1		poly-hydroxy fatty acids biosynthesis
AH03G08130.1	Cutin Synthesis	poly-hydroxy fatty acids biosynthesis
AH13G10320.1	Cutin Synthesis	poly-hydroxy fatty acids biosynthesis
AH19G32070.1	Cutin Synthesis	poly-hydroxy fatty acids biosynthesis
AH19G32260.1	Cutin Synthesis	poly-hydroxy fatty acids biosynthesis
AH16G23420.1	Cutin Synthesis	omega- hydroxylation of laurate
AH06G17280.1		omega- hydroxylation of laurate
AH19G43870.1		omega- hydroxylation of laurate
AH06G13860.1	Cuticular Wax Synthesis	
AH09G35630.1	Cuticular Wax Synthesis	
AH16G19170.1	Cuticular Wax Synthesis	
AH19G41940.1	Cuticular Wax Synthesis	
AH10G34800.1		triacylglycerol biosynthesis
AH10G34820.1		triacylglycerol biosynthesis
AH13G57090.1		triacylglycerol biosynthesis
AH13G57110.1		triacylglycerol biosynthesis

Supplementary Table 11 Statistics of read mapping for 52 resequenced accessions.

Genotype	Abbreviated name	Total HQ reads	Total reads mapped	Mapping Rate (%)*	Reads mapped to A-genome	Reads mapped to B-genome	Mapping ratio of B/A genomes	Reads mapped to unanchored scaffolds
Shitouqi	STQ_4	468900579	468481789	99.91068682	193756327	262631214	1.355	12094248
Fuhuasheng	FHS_2	355170782	355028437	99.9599221	146146670	197722879	1.353	11158888
Haihua 1	HH1	399110874	398678957	99.8917802	163288732	219504280	1.344	15885945
Baisha1016	BS1016	343545458	343178333	99.89313641	141177908	188654584	1.336	13345841
Luhua 11	LH11	390007356	389468956	99.86195132	160771479	214351716	1.333	14345761
Shanyou 27	SY27	390984827	390271474	99.8175497	160289198	214414390	1.338	15567886
Zhonghua 16	ZH16	344262705	343830247	99.8743814	141089226	189462413	1.343	13278608
Yuanza 9102	YZ9102	374524236	374041613	99.87113704	154693314	206745443	1.336	12602856
Minhua 6	Min6	434937556	434243805	99.84049411	178529259	238215543	1.334	17499003
Yuhua 23	YH23	322010501	321548026	99.85637891	131648924	177256219	1.346	12642883
Minhua 8	Min8_1	550972717	550814605	99.97130312	226422153	305913878	1.351	18478574
Min6-A	M6_A	312944151	312266953	99.7836042	123653007	170822212	1.381	17791734
Min8-B	M8_B	330734971	330432510	99.90854883	135433274	182368762	1.347	12630474
Minhua 14	EM7_10	327734001	325739094	99.39130301	132915879	178797225	1.345	14025990
Hexianqingdou	PTX	352814791	352115578	99.80181868	144380031	193062649	1.337	14672898
SunOleic 95R	Sun	428625171	428095556	99.87643866	176039596	236182467	1.341	15873493
Binyangdagouyao	LSX	346993792	346412152	99.8323774	142289678	191403325	1.345	12719149
Hexiandazidou	HXD	378227452	377231243	99.73661113	157057224	205697492	1.310	14476527
ICG6340	ICG	421815129	420752727	99.74813563	171648267	232267257	1.353	16837203
Liaoningshilihong	DLX	367475914	366824233	99.82266021	150446059	201864641	1.342	14513533
ICG15232	ICG15232	249990932	249207716	99.68670224	103460758	136609966	1.320	9136992
ICG4998	ICG4998	162021999	161389029	99.60933083	66424154	89146679	1.342	5818196
ICG5662	ICG5662	209084026	208412020	99.67859525	85212197	113573691	1.333	9626132
ICG5663	ICG5663	183507583	182513303	99.45818043	75809180	99665455	1.315	7038668
ICG11515	ICG11515	211170582	210579301	99.7199984	87855919	116267527	1.323	6455855
ICG11651	ICG11651	214149776	213304503	99.60528887	88260609	116789925	1.323	8253969
ICG4729	ICG4729	183051606	182268074	99.57196114	75812005	100268795	1.323	6187274
ICG14985	ICG14985	197964351	197436168	99.73319287	81867933	108303984	1.323	7264251
ICG334	ICG334	203397239	202884577	99.74795036	84493925	111324268	1.317	7066384
ICG10701	ICG10701	214599247	213486592	99.48151962	88839622	117223842	1.319	7423128
PI219824	Mon	376802264	376495870	99.91868573	155030039	208919924	1.348	12545907
ISATGR5	ISATGR5	891664274	882701996	98.99488201	129772255	736599731	5.676	16330010
ISATGR278	ISATGR278-18	1101861075	1091757067	99.08300527	591080719	458389419	0.777	42286929
ISATGR1212	ISATGR1212	843574610	836837634	99.20137758	445797396	363529069	0.815	27511169
ISATGR184	ISATGR184	1136299258	1128829229	99.3426002	924409784	155806850	0.169	48612595
ICG8123	ICG8123	466371433	462934904	99.26313476	402016682	41064709	0.102	19853513
ICG8138	ICG8138	465822915	463095597	99.41451614	404923278	37407642	0.092	20764677
ICG8209	ICG8290	426110091	421979102	99.03053481	99521400	311440644	3.129	11017058

ICG13160	ICG13160	445197897	440588950	98.96474197	100856554	324995850	3.222	14736546
ICG8960	ICG8960	430236037	428580227	99.61513917	22448453	399407004	17.792	6724770
ICG8206	ICG8206	513139239	508763922	99.14734312	11157384	487727878	43.713	9878660
PI262133	Adu	310831263	308851463	99.36306278	250899674	46322894	0.185	11628895
PI19455	Aip	319541047	316389667	99.01377928	5872004	305093350	51.957	5424313
PI337308	Ast	325576016	323651836	99.40899209	267780108	45538902	0.170	10332826
ICG8125	ICG8125	83567808	83076898	99.41256088	66455827	12529125	0.189	4091946
ICG8216	ICG8216	89114566	87969623	98.71520106	72145308	10752071	0.1490	5072244
ICG11561	ICG11561	91026349	90516192	99.43955019	74740606	10318078	0.138	5457508
ICG13232	ICG13232	63806553	63443770	99.43143301	51240245	9553443	0.186	2650082
ICG8190	ICG8190	87575449	86761248	99.07028624	65878205	17265440	0.262	3617603
PI338314	Apin	379763108	311762544	82.09395211	111750378	192141441	1.719	7870725
ICG11560	ICG11560	67361431	66946041	99.38334149	54822711	8353036	0.152	3770294
ICG8215	ICG8215	103435921	92288756	89.22312008	31634151	58526972	1.850	2127633
*The mapping rate of several accessions of diploids are different from Nextomics								
With wild diploids, especially remote *Arachis* species, the mapping and analysis methods should be different from the tetraploid accessions.								

Supplementary Table 12 Whole genome re-sequencing data generated on BSA using Illumina HiSeq 2500.

Samples	Genotype/bulks	HQ Reads	Reads mapped	Genome coverage at >=2 x	Genome coverage at >= 5x	Average mapping depth
**Pod size**						
Parent with big seed	Yueyou 92	382,713,001	380,684,622	96.46	94.48	42.58
Parent with small seed	Xinhuixiaoli	344,100,662	342,681,899	95.49	90.23	38.69
Big seed bulk	Big seed	542,566,359	539,690,757	96.90	95.17	59.15
Small seed bulk	Small seed	646,204,840	643,749,261	97.17	96.13	71.06
**Foliar fungal disease resistance (rust and LLS resistance)**						
Resistant parent for rust and LLS	GPBD 4	293,508,707	291,610,791	96.58	91.33	11.36
Susceptible parent for rust and LLS	TAG 24	295,481,743	293,605,207	96.59	91.68	11.44
Resistant bulk (R-LLS)	R-LLS	282,206,463	278,734,251	96.34	90.71	10.70
Susceptible bulk (S-LLS)	S-LLS	288,765,162	286,908,255	96.78	96.78	11.18
Resistant bulk (R-Rust)	R-Rust	283,209,868	279,794,653	96.68	90.57	10.90
Susceptible bulk (S-Rust)	S-Rust	283,163,371	275,095,555	96.77	90.61	10.72

Late leaf spot: LLS.

Supplementary Table 13 Quality analysis of high oleate mutants by physical and chemical analysis.

Variety Name	Near infrared spectrum analysis results					Chemical analysis results				
	Protein (%)	Oil (%)	Oleic acid (%)	Linoleic Acid (%)	O/L	Protein (%)	Oil (%)	Oleic acid (%)	Linoleic Acid (%)	O/L
Minhua 6	28.24	49.46	40.17	35.67	1.13	29.5	50	42.1	35.9	1.17
Minhua 8	28.3	48.67	47.4	32.2	1.47	27.8	50.3	45.8	32.5	1.41
BOM7a112	29.19	49.61	88.34	-7.7	-11.47	25.2	47.8	78.4	4.3	18.16
AOM7a151	31.89	39.23	88.67	-0.82	-108.13	25.2	47.8	81.4	2.3	35.39
AOM7a513	31.91	42.38	80.37	6.66	12.07					
BOM7a115	26.86	53.98	82.11	0.74	110.96	23.2	49.8	78.4	4.3	18.23
BOM7a114	29.58	50.66	86.95	-1.44	-60.38	25.2	47.8	77.1	2.3	33.52
BOM7a113	28.1	49.23	86.93	-2.76	-31.5	25.2	46.8	78.4	4.3	18.23
BOM7a353	26.85	49.74	88.32	-2.84	-31.1	23.1	43.6	80.6	3.3	24.42
BOM7a313	31.61	48.21	72.16	7.06	10.22					
BOM7a111	30.15	46.72	97.34	-13.34	-7.3	25.2	47.8	78.8	4.3	18.33
BOM7b122	30.16	51.31	72.23	10.71	6.74					
BOM7b351	30.31	44.86	71.5	12.2	5.86					
BOM7b212	27.88	49.61	102.08	-17.66	-5.78	25.2	48.8	79.4	2.3	34.52

Note: High oleate varieties derived by EMS and r-ray treatment of Minhua 6 and Minhua 8, respectively.

Supplementary Data sets

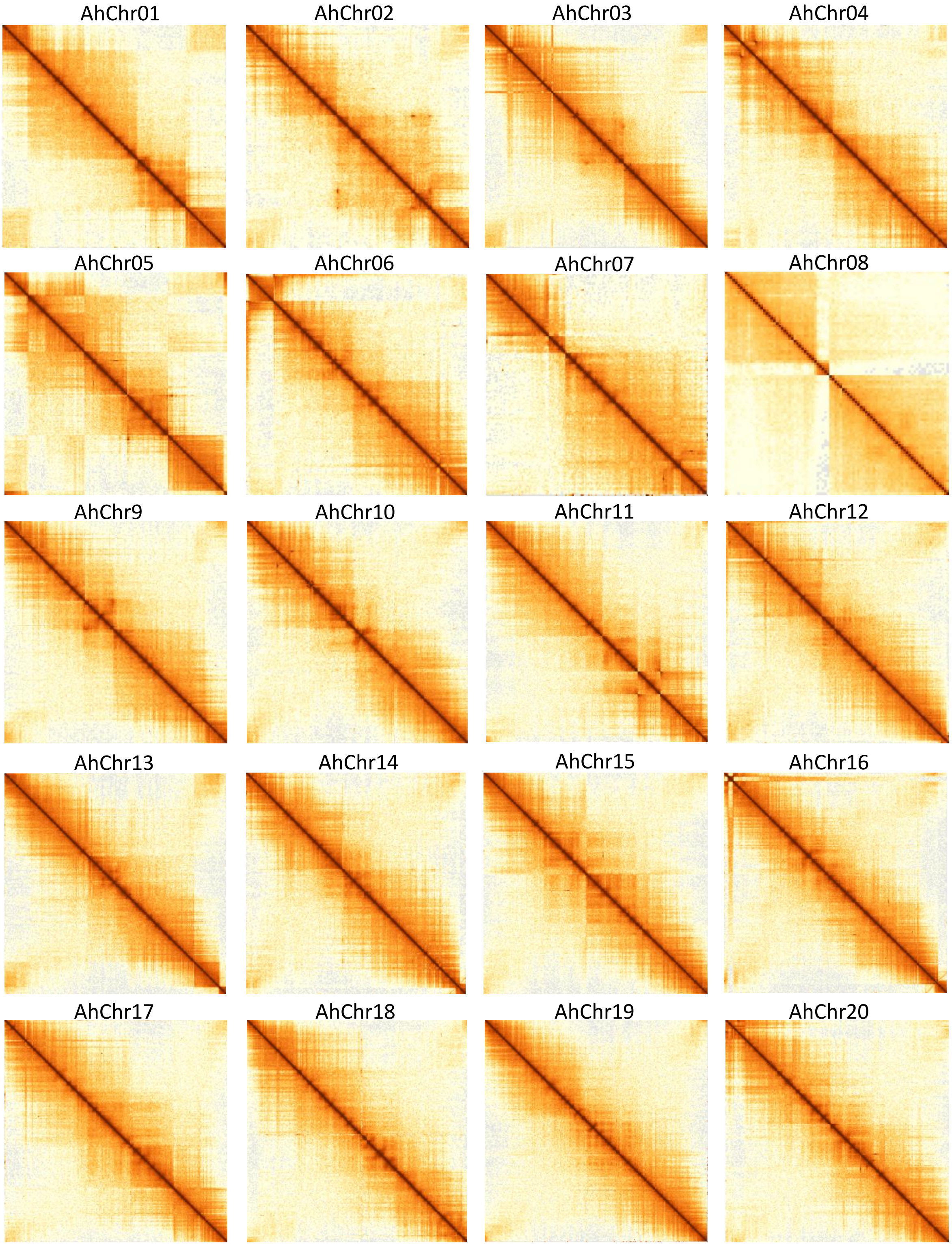

Supplementary Data Set 1 Heatmaps for HiC assembly in peanut chromosomes. The HiC data were used to map the *A. hypogaea* genome sequences. Each heatmap is shown at a resolution 500 Kb. The dots from dark red to light yellow show high to low probability of interactions. Heatmaps for chromosome 19 was shown in **Fig. 1b. All heatmaps were collectively shown in Fig. 1a.**

Supplementary Data Set 2 Genome map integration from four genetic maps.

The four input genetic maps included map 314 with 5,019 SSR markers, map 267 with 7,184 SNP markers, map 1623 with 1,592 SNP markers, and map 1954 with 1,836 SNP markers following the hierarchy priority level from high to low. The four maps were integrated into one map using Allmaps (Tang et al., 2015) with 7,566 contigs assembled from HiC sequencing as the reference. CMAP-style map of each chromosome is presented on the left. Different colored lines connecting the marker positions on each genetic map with physical positions on the reconstructed chromosome. On the left of each integrated chromosome are the scatter plots of each integrated map with different colored dots representing the physical position on the chromosome (x-axis) versus the map location (y-axis). Color codes: pink for map 314, blue map 267, green map 1623, and orange for map 1954. Each reconstructed chromosome is shown as a box at the bottom of scatter plots with alternating shades, marking the boundaries of the component scaffolds. The ρ-value on each scatter plot measures the Pearson correlation coefficient, with values in the range of -1 to 1 (values closer to -1 and 1 indicate near-perfect collinearity) (Tang et al., 2015).

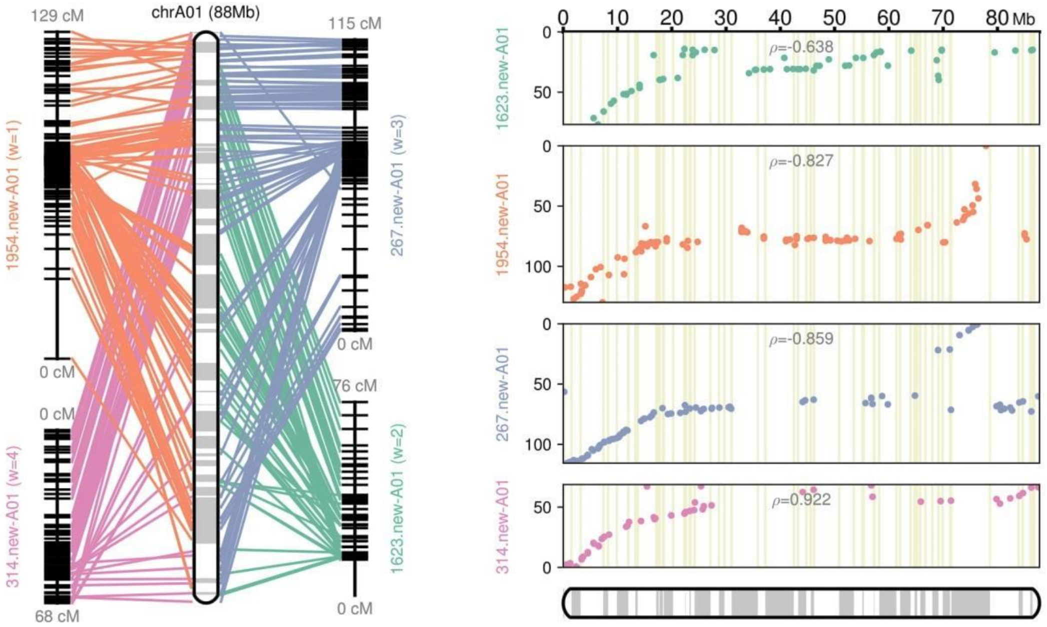
chrA01 (88Mb)
129 cM
115 cM
1954.new-A01 (w=1)
267.new-A01 (w=3)
0 cM
76 cM
0 cM
314.new-A01 (w=4)
1623.new-A01 (w=2)
68 cM
0 cM
Mb
1623.new-A01
ρ=-0.638
1954.new-A01
ρ=-0.827
267.new-A01
ρ=-0.859
314.new-A01
ρ=0.922

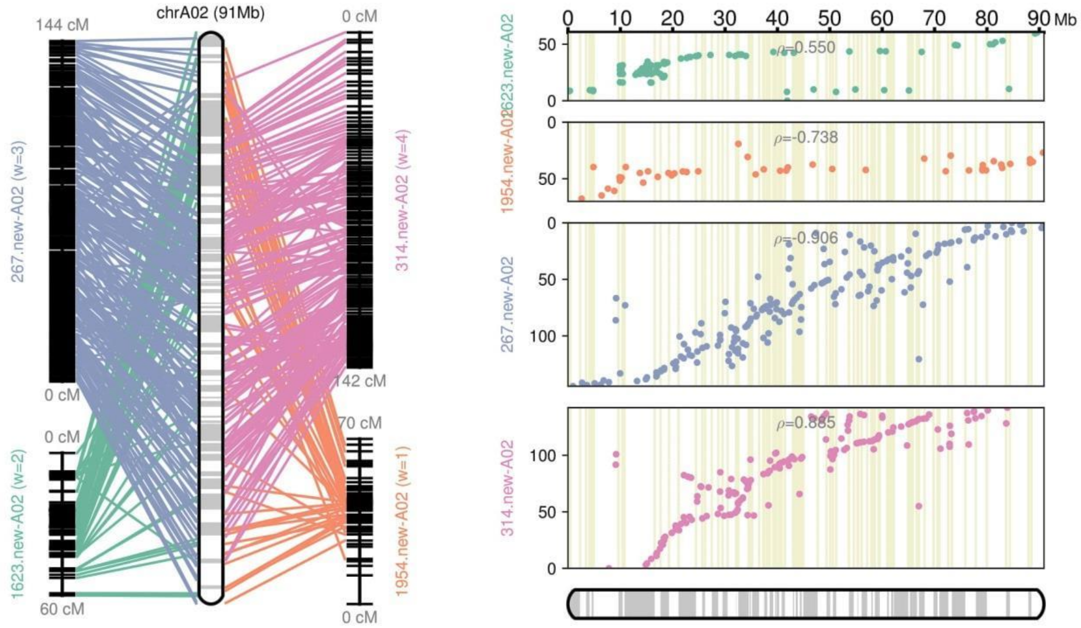
chrA02 (91Mb)
144 cM
0 cM
267.new-A02 (w=3)
314.new-A02 (w=4)
0 cM
142 cM
0 cM
70 cM
1623.new-A02 (w=2)
1954.new-A02 (w=1)
60 cM
0 cM
Mb
1623.new-A02
ρ=0.550
1954.new-A02
ρ=-0.738
267.new-A02
ρ=-0.906
314.new-A02
ρ=0.885

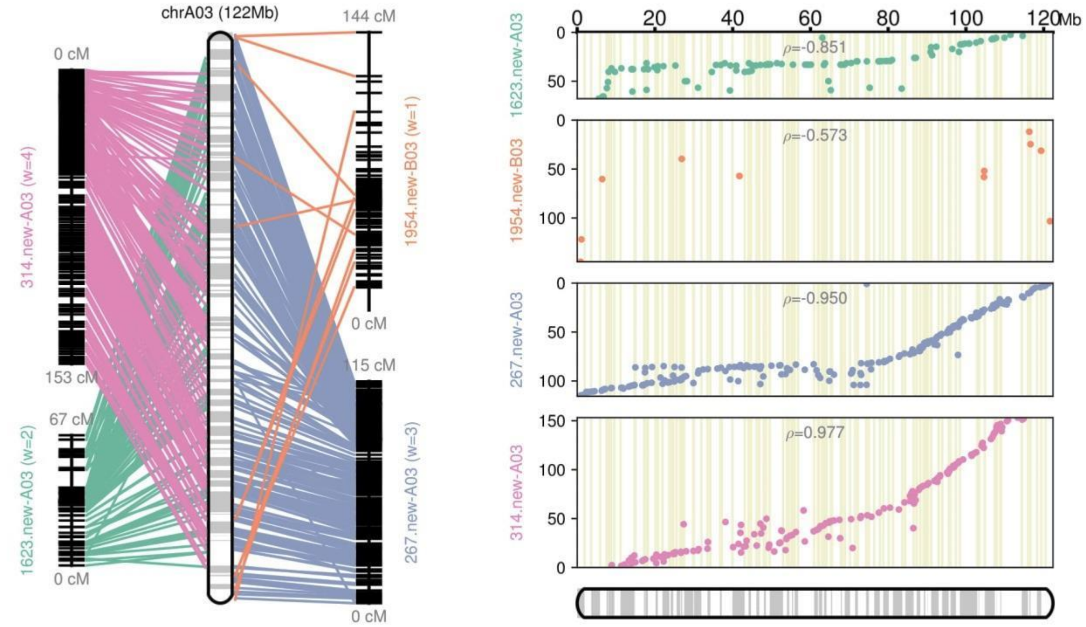
chrA03 (122Mb)
0 cM
153 cM
314.new-A03 (w=4)
67 cM
0 cM
1623.new-A03 (w=2)
144 cM
0 cM
1954.new-B03 (w=1)
115 cM
0 cM
267.new-A03 (w=3)
0
20
40
60
80
100
120Mb
1623.new-A03
ρ=-0.851
1954.new-B03
ρ=-0.573
267.new-A03
ρ=-0.950
314.new-A03
ρ=0.977

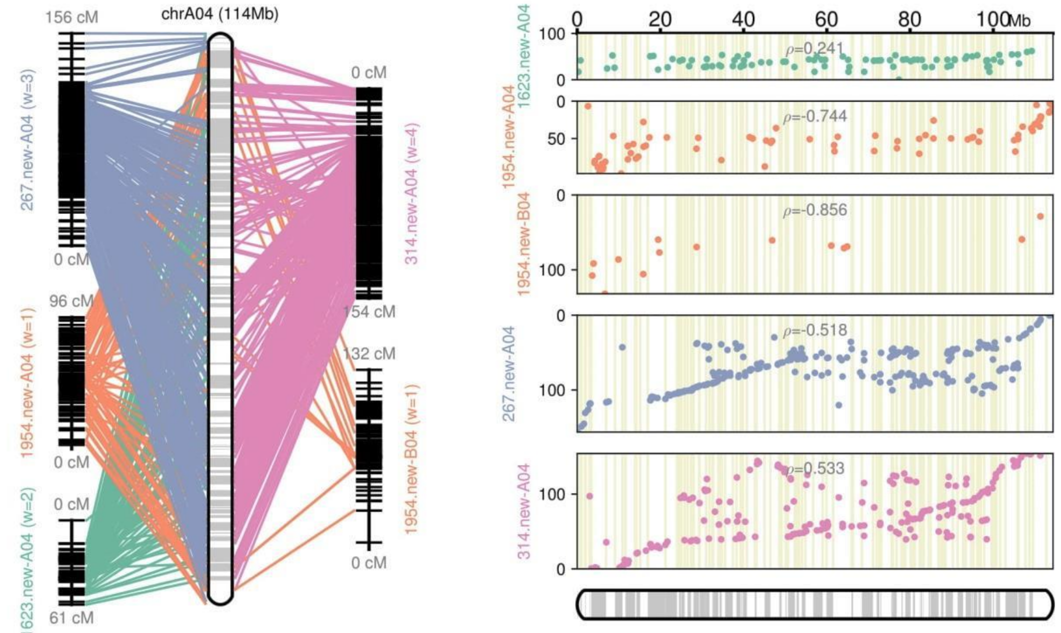
chrA04 (114Mb)
156 cM
0 cM
267.new-A04 (w=3)
96 cM
0 cM
1954.new-A04 (w=1)
0 cM
61 cM
1623.new-A04 (w=2)
0 cM
154 cM
314.new-A04 (w=4)
132 cM
0 cM
1954.new-B04 (w=1)
0
20
40
60
80
100Mb
1623.new-A04
ρ=0.241
1954.new-A04
ρ=-0.744
1954.new-B04
ρ=-0.856
267.new-A04
ρ=-0.518
314.new-A04
ρ=0.533

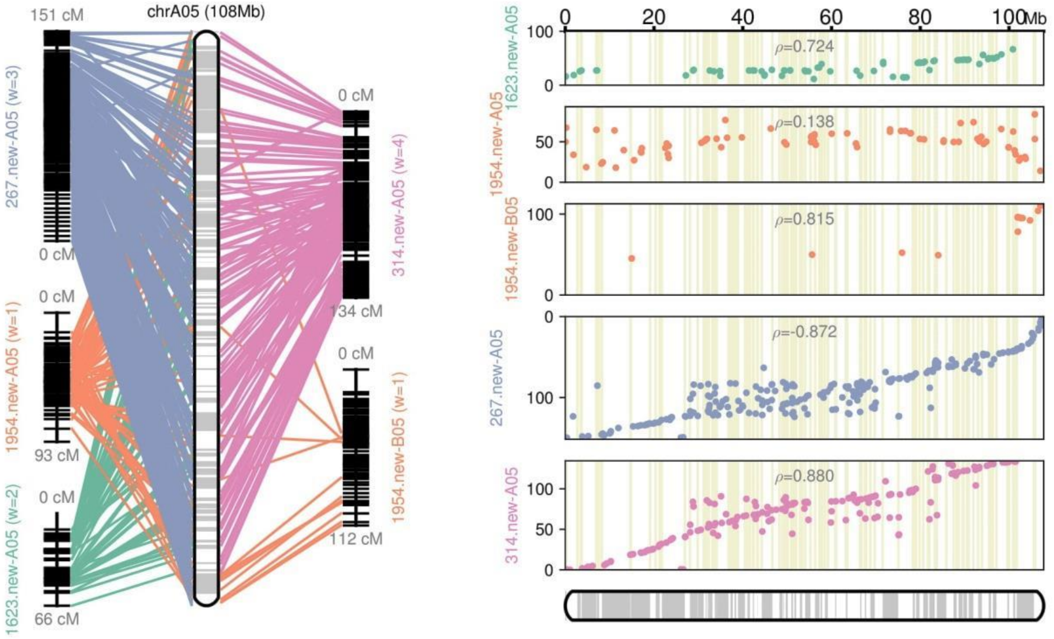
chrA05 (108Mb)
151 cM
267.new-A05 (w=3)
0 cM
0 cM
1954.new-A05 (w=1)
93 cM
0 cM
1623.new-A05 (w=2)
66 cM
0 cM
314.new-A05 (w=4)
134 cM
0 cM
1954.new-B05 (w=1)
112 cM
0
20
40
60
80
100
Mb
1623.new-A05
ρ=0.724
1954.new-A05
ρ=0.138
1954.new-B05
ρ=0.815
267.new-A05
ρ=-0.872
314.new-A05
ρ=0.880

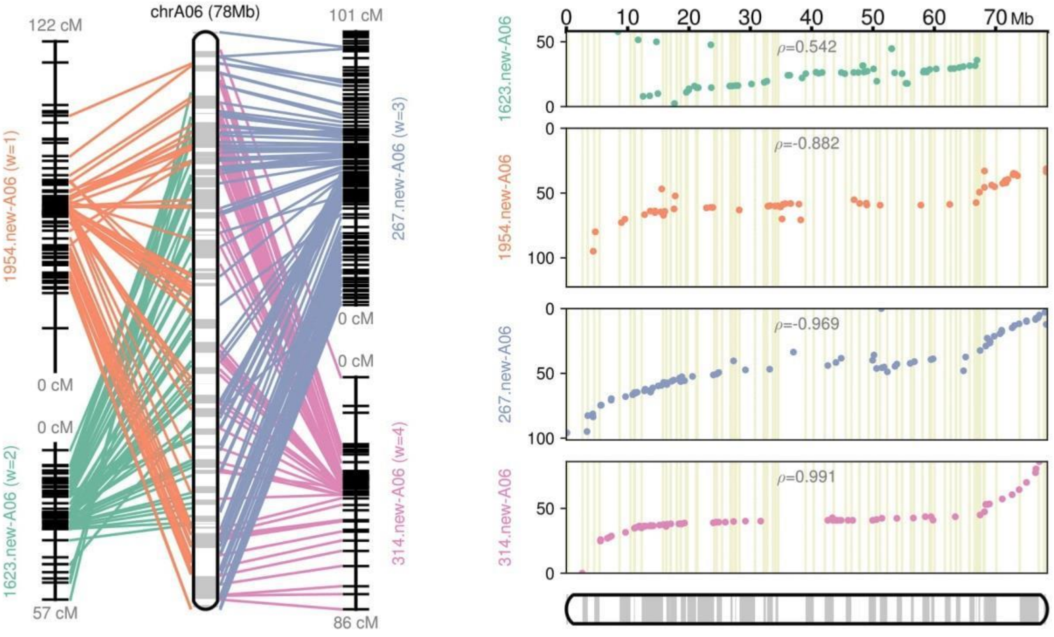
chrA06 (78Mb)
122 cM
1954.new-A06 (w=1)
0 cM
0 cM
1623.new-A06 (w=2)
57 cM
101 cM
267.new-A06 (w=3)
0 cM
0 cM
314.new-A06 (w=4)
86 cM
0
10
20
30
40
50
60
70
Mb
1623.new-A06
ρ=0.542
1954.new-A06
ρ=-0.882
267.new-A06
ρ=-0.969
314.new-A06
ρ=0.991

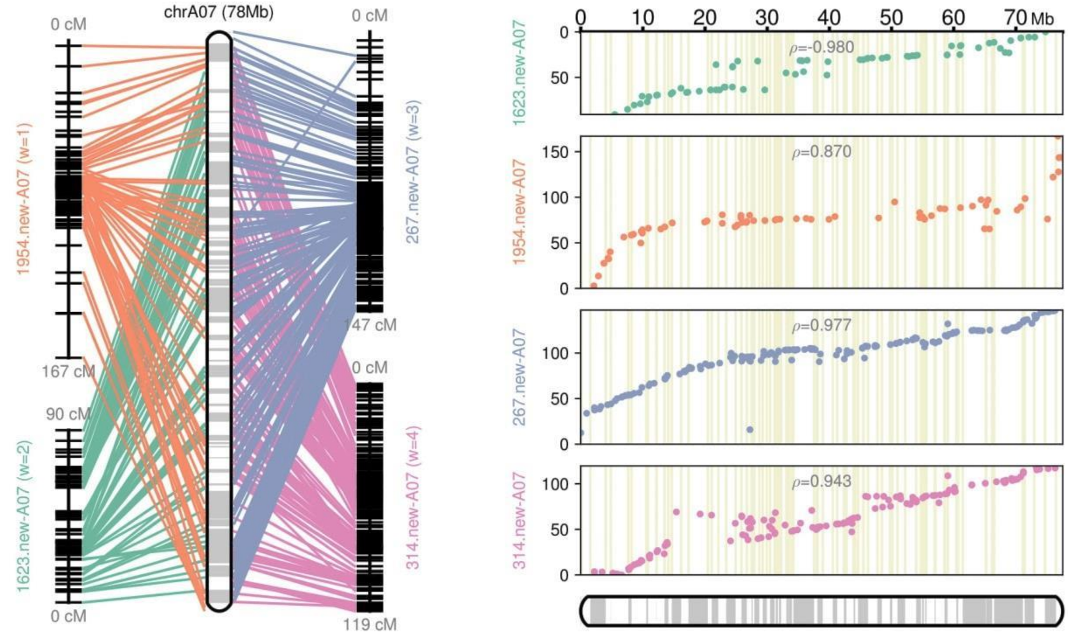
chrA07 (78Mb)
0 cM
167 cM
90 cM
0 cM
47 cM
119 cM
1954.new-A07 (w=1)
1623.new-A07 (w=2)
267.new-A07 (w=3)
314.new-A07 (w=4)
0 10 20 30 40 50 60 70 Mb
1623.new-A07
ρ=-0.980
1954.new-A07
ρ=0.870
267.new-A07
ρ=0.977
314.new-A07
ρ=0.943

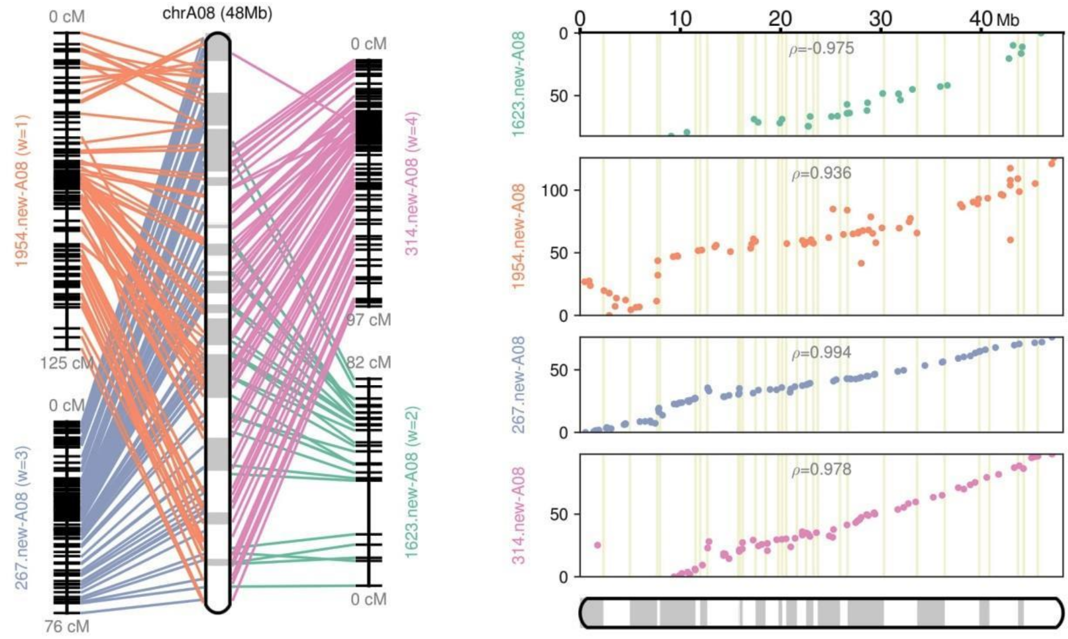
chrA08 (48Mb)
0 cM
125 cM
76 cM
97 cM
82 cM
1954.new-A08 (w=1)
267.new-A08 (w=3)
314.new-A08 (w=4)
1623.new-A08 (w=2)
0 10 20 30 40 Mb
1623.new-A08
ρ=-0.975
1954.new-A08
ρ=0.936
267.new-A08
ρ=0.994
314.new-A08
ρ=0.978

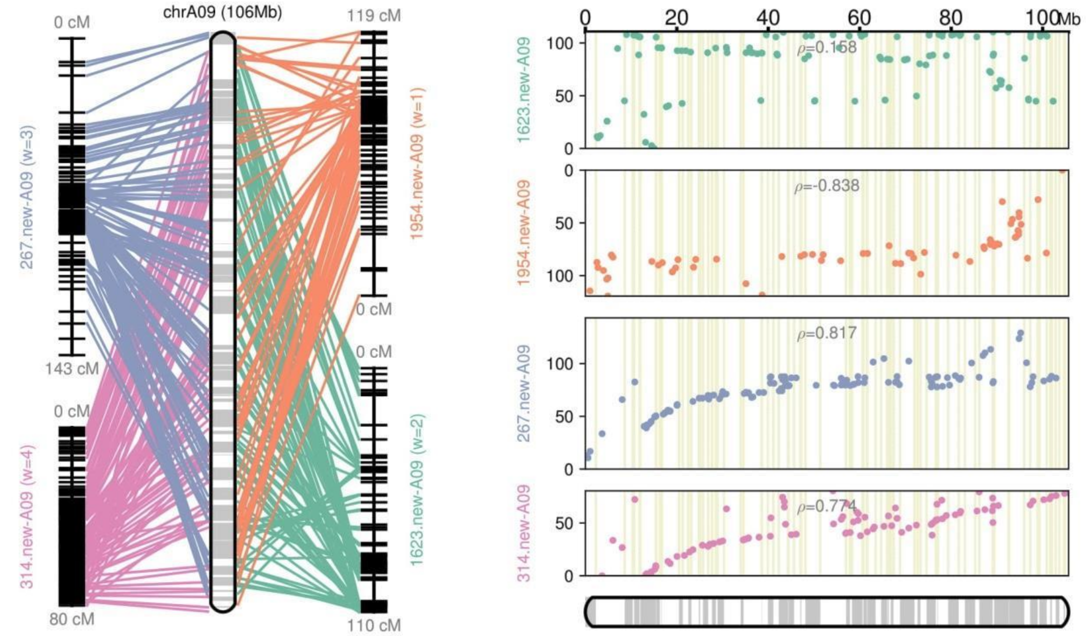
chrA09 (106Mb)
0 cM
143 cM
267.new-A09 (w=3)
0 cM
80 cM
314.new-A09 (w=4)
119 cM
0 cM
1954.new-A09 (w=1)
0 cM
110 cM
1623.new-A09 (w=2)
0
20
40
60
80
100
Mb
1623.new-A09
ρ=0.168
1954.new-A09
ρ=-0.838
267.new-A09
ρ=0.817
314.new-A09
ρ=0.774

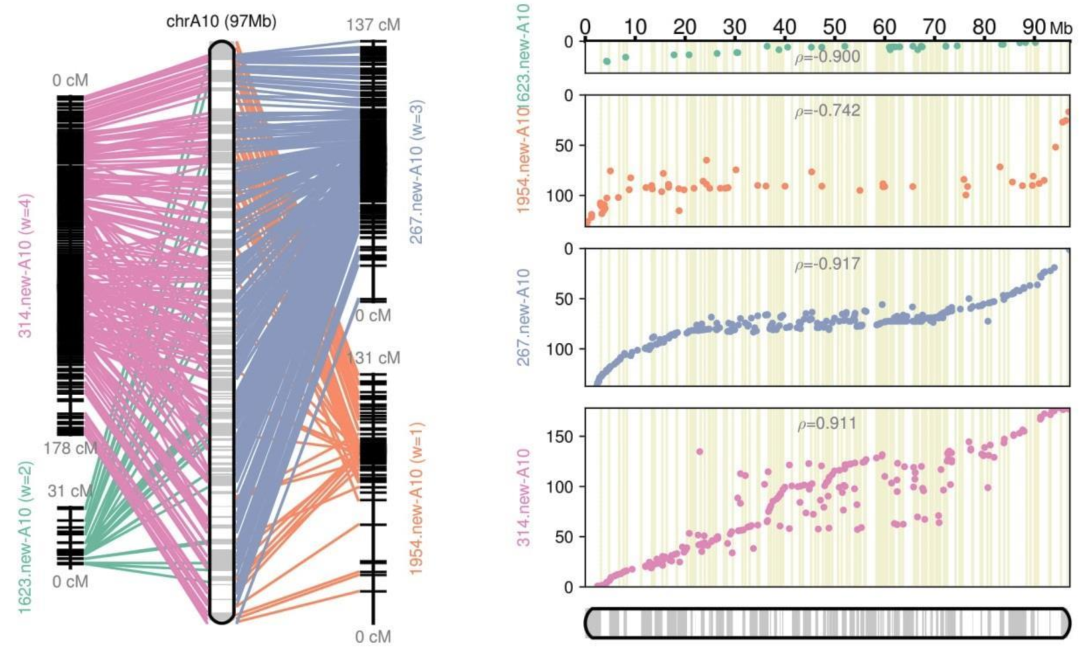
chrA10 (97Mb)
0 cM
178 cM
314.new-A10 (w=4)
31 cM
0 cM
1623.new-A10 (w=2)
137 cM
0 cM
267.new-A10 (w=3)
131 cM
0 cM
1954.new-A10 (w=1)
0
10
20
30
40
50
60
70
80
90
Mb
1623.new-A10
ρ=-0.900
1954.new-A10
ρ=-0.742
267.new-A10
ρ=-0.917
314.new-A10
ρ=0.911

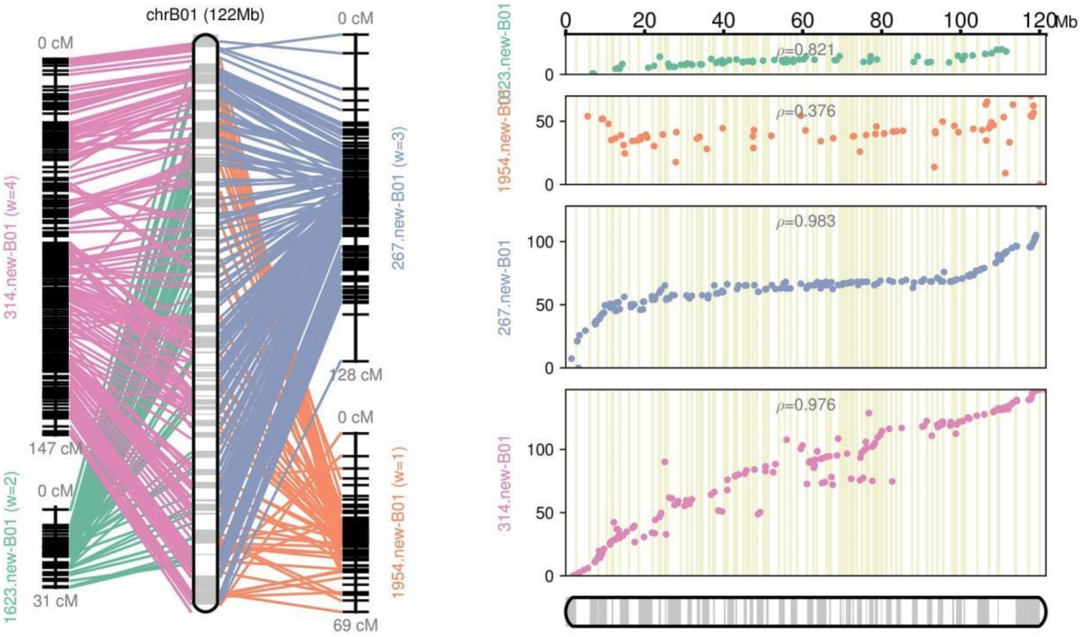
chrB01 (122Mb)
0 cM
147 cM
314.new-B01 (w=4)
0 cM
31 cM
1623.new-B01 (w=2)
0 cM
128 cM
267.new-B01 (w=3)
0 cM
69 cM
1954.new-B01 (w=1)
0
20
40
60
80
100
120
Mb
1623.new-B01
ρ=0.821
1954.new-B01
ρ=0.376
267.new-B01
ρ=0.983
314.new-B01
ρ=0.976

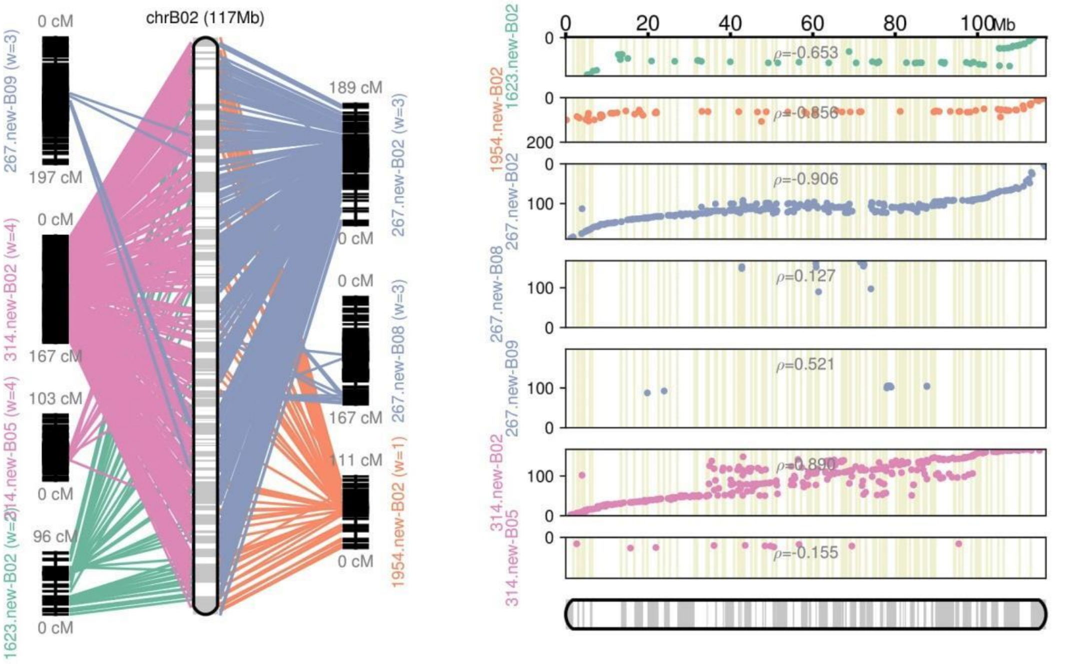
chrB02 (117Mb)
0 cM
197 cM
267.new-B09 (w=3)
0 cM
167 cM
314.new-B02 (w=4)
103 cM
0 cM
314.new-B05 (w=4)
96 cM
0 cM
1623.new-B02 (w=2)
189 cM
0 cM
267.new-B02 (w=3)
0 cM
167 cM
267.new-B08 (w=3)
111 cM
0 cM
1954.new-B02 (w=1)
0
20
40
60
80
100
Mb
1623.new-B02
ρ=-0.653
1954.new-B02
ρ=-0.856
267.new-B02
ρ=-0.906
267.new-B08
ρ=0.127
267.new-B09
ρ=0.521
314.new-B02
ρ=0.890
314.new-B05
ρ=-0.155

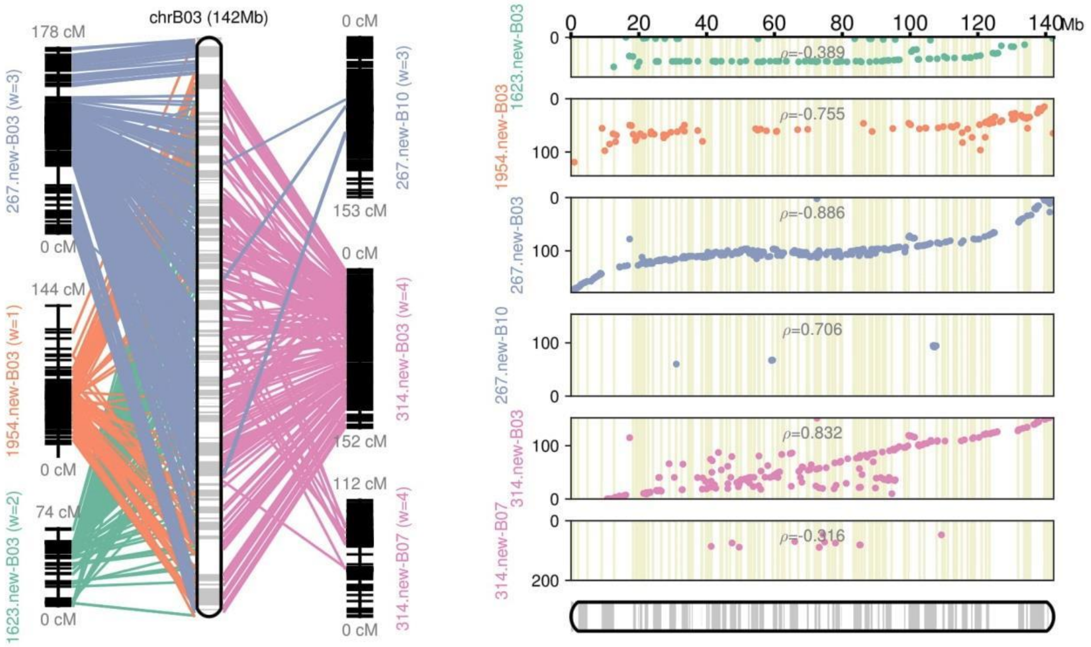
chrB03 (142Mb)
178 cM
267.new-B03 (w=3)
0 cM
144 cM
1954.new-B03 (w=1)
0 cM
74 cM
1623.new-B03 (w=2)
0 cM
0 cM
267.new-B10 (w=3)
153 cM
0 cM
314.new-B03 (w=4)
152 cM
112 cM
314.new-B07 (w=4)
0 cM
Mb
1623.new-B03
ρ=-0.389
1954.new-B03
ρ=-0.755
267.new-B03
ρ=-0.886
267.new-B10
ρ=0.706
314.new-B03
ρ=0.832
314.new-B07
ρ=-0.316

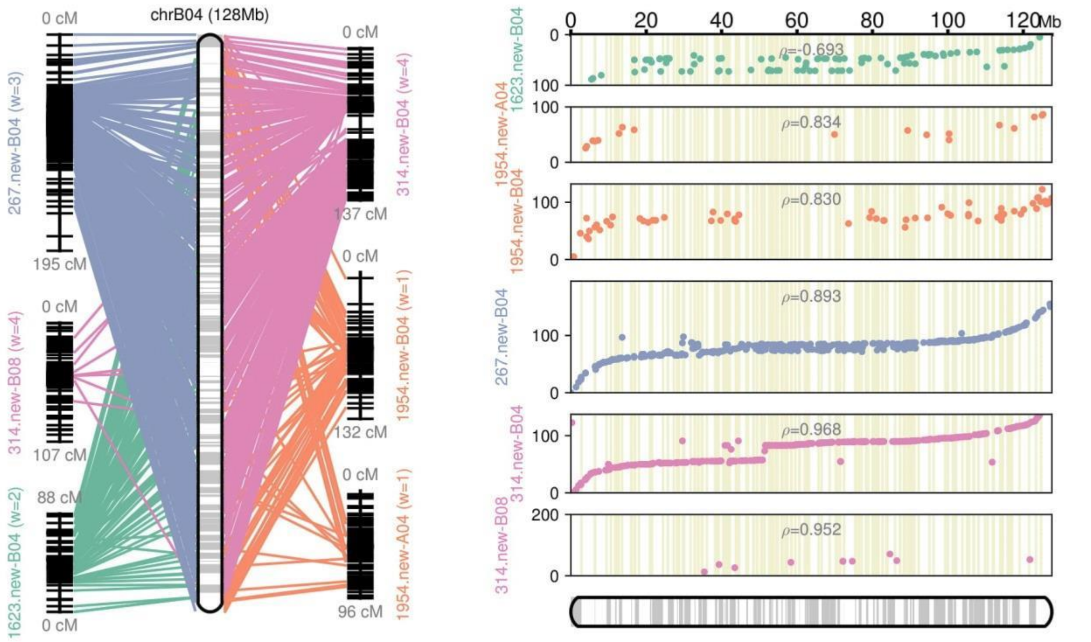
chrB04 (128Mb)
0 cM
267.new-B04 (w=3)
195 cM
0 cM
314.new-B08 (w=4)
107 cM
88 cM
1623.new-B04 (w=2)
0 cM
0 cM
314.new-B04 (w=4)
137 cM
0 cM
1954.new-B04 (w=1)
132 cM
0 cM
1954.new-A04 (w=1)
96 cM
Mb
1623.new-B04
ρ=-0.693
1954.new-A04
ρ=0.834
1954.new-B04
ρ=0.830
267.new-B04
ρ=0.893
314.new-B04
ρ=0.968
314.new-B08
ρ=0.952

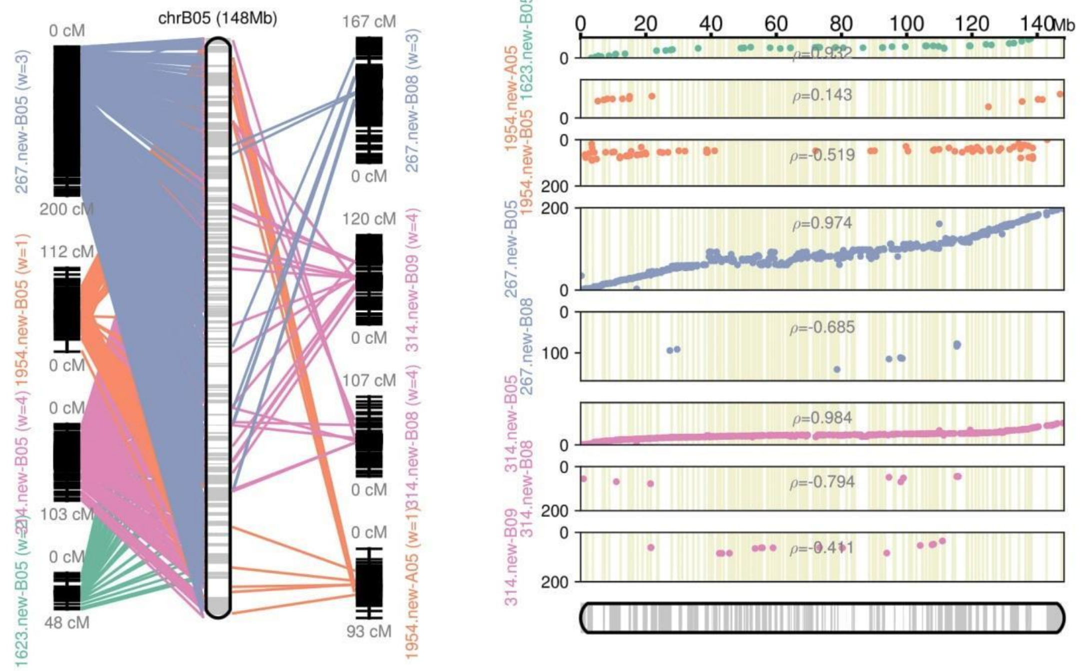
chrB05 (148Mb)
267.new-B05 (w=3)
1954.new-B05 (w=1)
314.new-B05 (w=4)
1623.new-B05 (w=2)
267.new-B08 (w=3)
314.new-B09 (w=4)
314.new-B08 (w=4)
1954.new-A05 (w=1)
0 cM
200 cM
112 cM
103 cM
48 cM
167 cM
120 cM
107 cM
93 cM
Mb
ρ=0.932
ρ=0.143
ρ=-0.519
ρ=0.974
ρ=-0.685
ρ=0.984
ρ=-0.794
ρ=-0.411

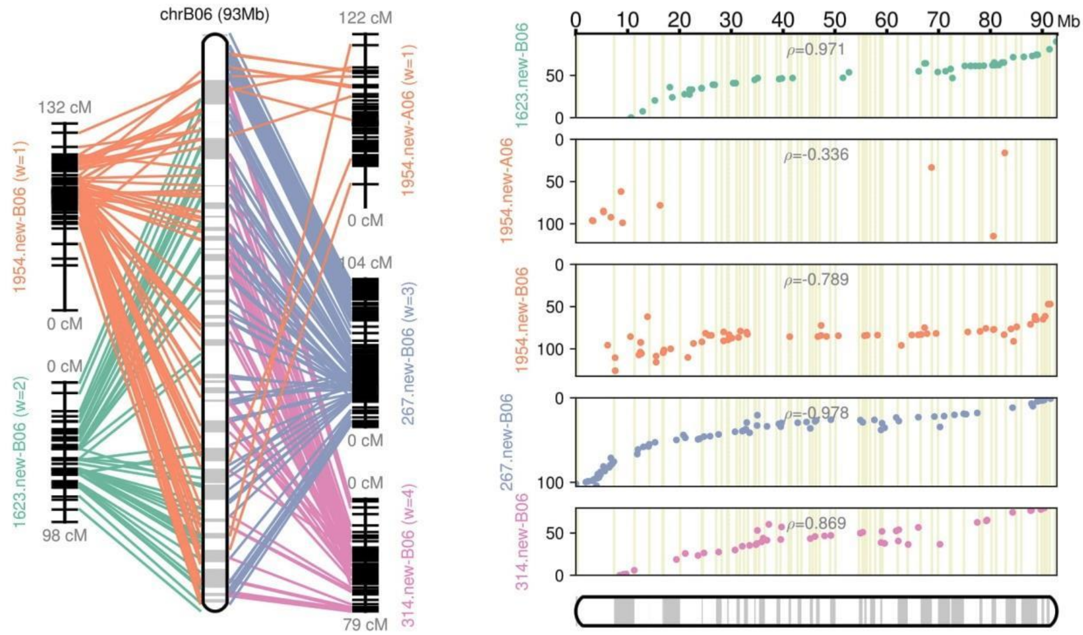
chrB06 (93Mb)
1954.new-B06 (w=1)
1623.new-B06 (w=2)
1954.new-A06 (w=1)
267.new-B06 (w=3)
314.new-B06 (w=4)
132 cM
0 cM
98 cM
122 cM
104 cM
79 cM
Mb
ρ=0.971
ρ=-0.336
ρ=-0.789
ρ=-0.978
ρ=0.869

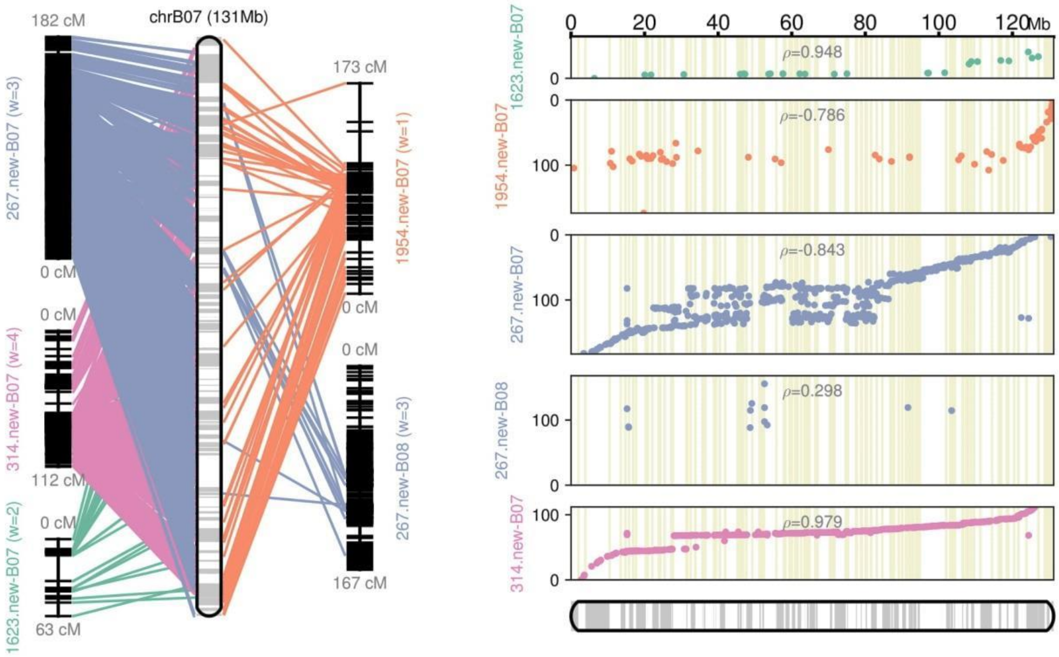

chrB07 (131Mb)
182 cM
0 cM
267.new-B07 (w=3)
0 cM
314.new-B07 (w=4)
112 cM
0 cM
1623.new-B07 (w=2)
63 cM
173 cM
1954.new-B07 (w=1)
0 cM
0 cM
267.new-B08 (w=3)
167 cM
0 20 40 60 80 100 120 Mb
1623.new-B07
ρ=0.948
1954.new-B07
ρ=-0.786
267.new-B07
ρ=-0.843
267.new-B08
ρ=0.298
314.new-B07
ρ=0.979

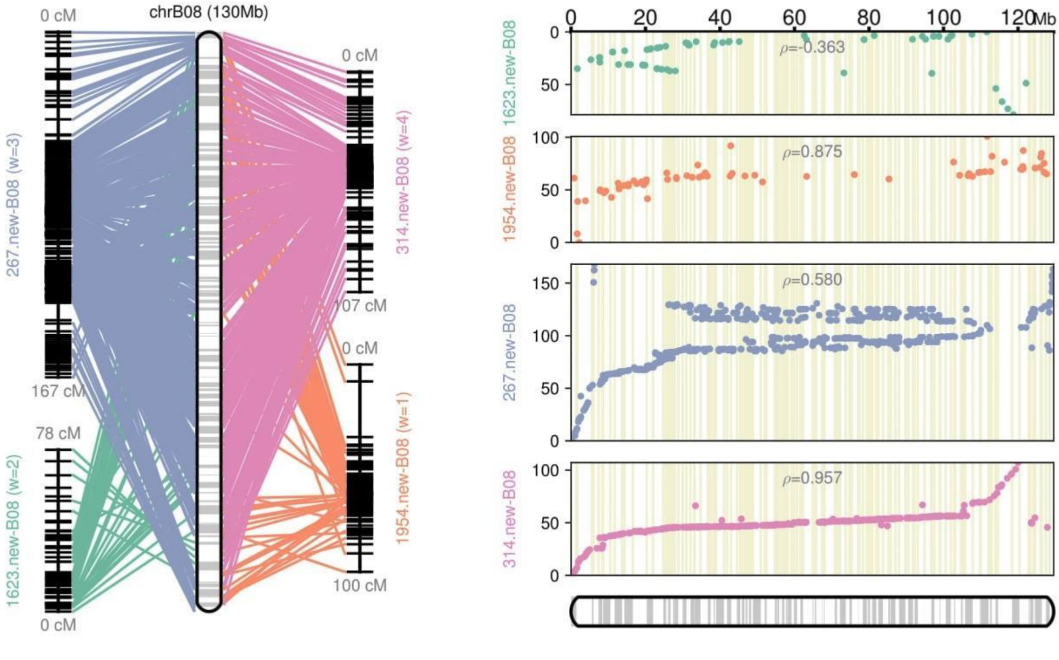

chrB08 (130Mb)
0 cM
267.new-B08 (w=3)
167 cM
78 cM
1623.new-B08 (w=2)
0 cM
0 cM
314.new-B08 (w=4)
107 cM
0 cM
1954.new-B08 (w=1)
100 cM
0 20 40 60 80 100 120 Mb
1623.new-B08
ρ=-0.363
1954.new-B08
ρ=0.875
267.new-B08
ρ=0.580
314.new-B08
ρ=0.957

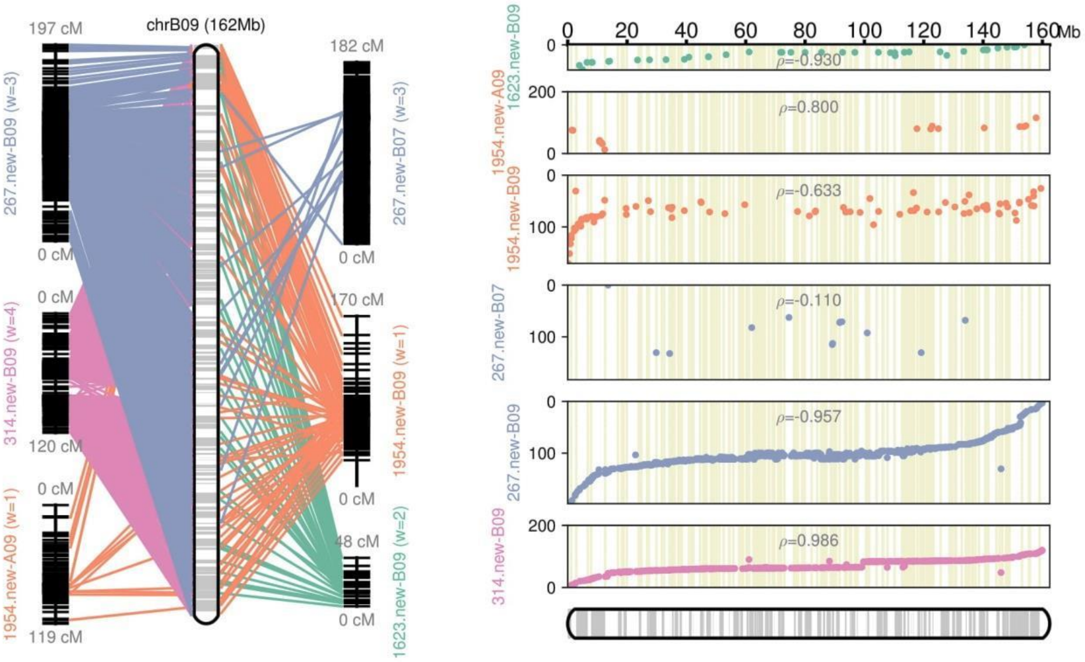
chrB09 (162Mb)
197 cM
0 cM
267.new-B09 (w=3)
0 cM
120 cM
314.new-B09 (w=4)
0 cM
119 cM
1954.new-A09 (w=1)
182 cM
0 cM
267.new-B07 (w=3)
170 cM
0 cM
1954.new-B09 (w=1)
48 cM
0 cM
1623.new-B09 (w=2)
Mb
1623.new-B09
ρ=-0.930
1954.new-A09
ρ=0.800
1954.new-B09
ρ=-0.633
267.new-B07
ρ=-0.110
267.new-B09
ρ=-0.957
314.new-B09
ρ=0.986

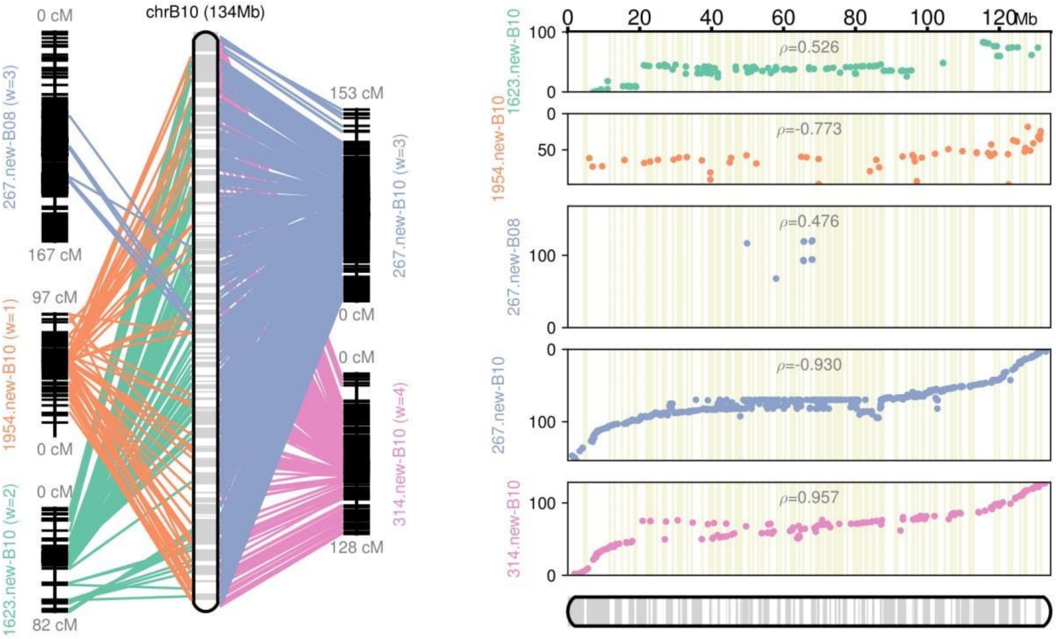
chrB10 (134Mb)
0 cM
167 cM
267.new-B08 (w=3)
97 cM
0 cM
1954.new-B10 (w=1)
0 cM
82 cM
1623.new-B10 (w=2)
153 cM
0 cM
267.new-B10 (w=3)
0 cM
128 cM
314.new-B10 (w=4)
Mb
1623.new-B10
ρ=0.526
1954.new-B10
ρ=-0.773
267.new-B08
ρ=0.476
267.new-B10
ρ=-0.930
314.new-B10
ρ=0.957

Supplementary Data Set 7. Dot plots showing sequencing identify of corresponding chromosomes between ancestor diploid and cultivated tetraploid peanut genomes. X-axis represents the physical position in base pair of the A. *hypogaea* chromosome stated on top of each subfigure. X-axis represents the sequence identify in percentage. Blue does represents the aligned sequence contigs from A. *ipaensis*. Orange dots represents the aligned sequences contigs from A. *duranensis*.

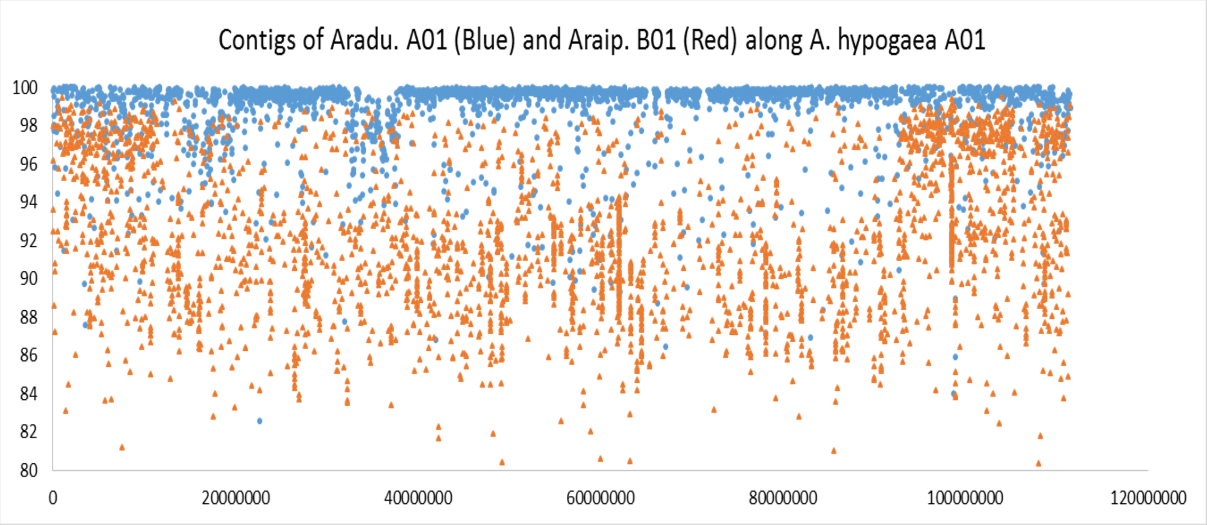

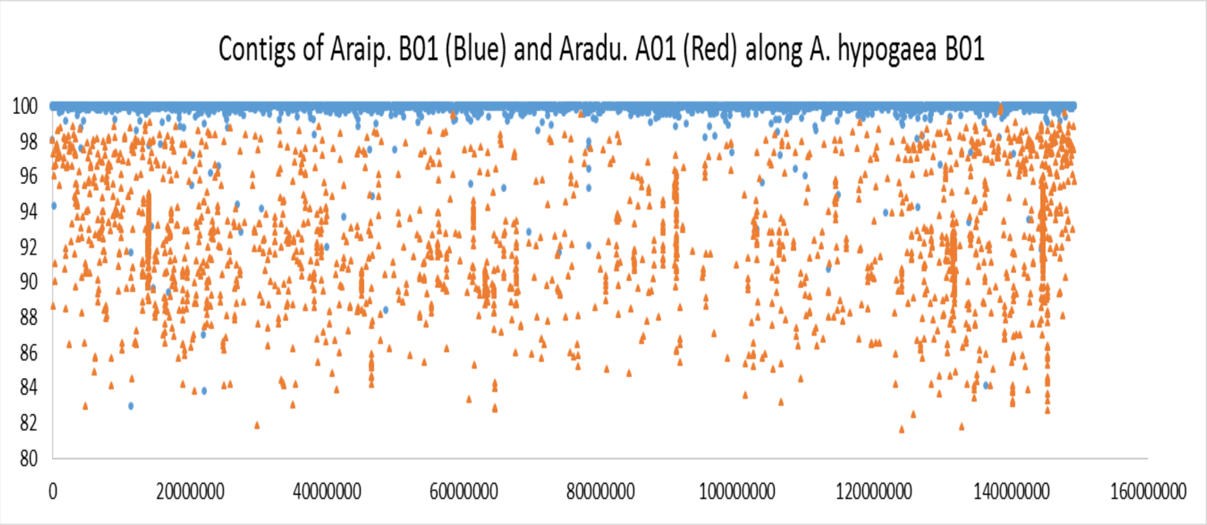

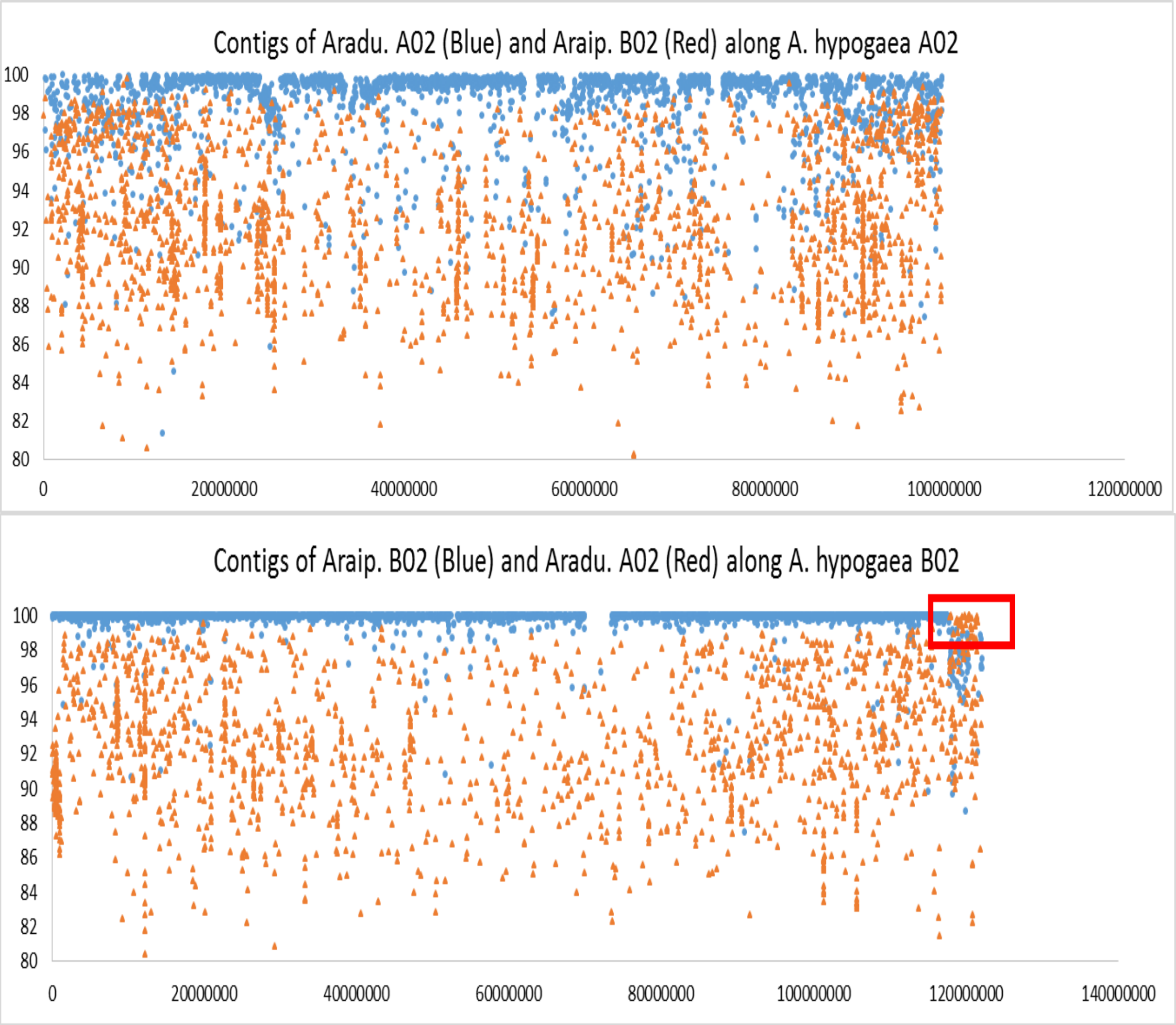
Contigs of Aradu. A02 (Blue) and Araip. B02 (Red) along A. hypogaea A02
100
98
96
94
92
90
88
86
84
82
80
0
20000000
40000000
60000000
80000000
100000000
120000000
Contigs of Araip. B02 (Blue) and Aradu. A02 (Red) along A. hypogaea B02
100
98
96
94
92
90
88
86
84
82
80
0
20000000
40000000
60000000
80000000
100000000
120000000
140000000

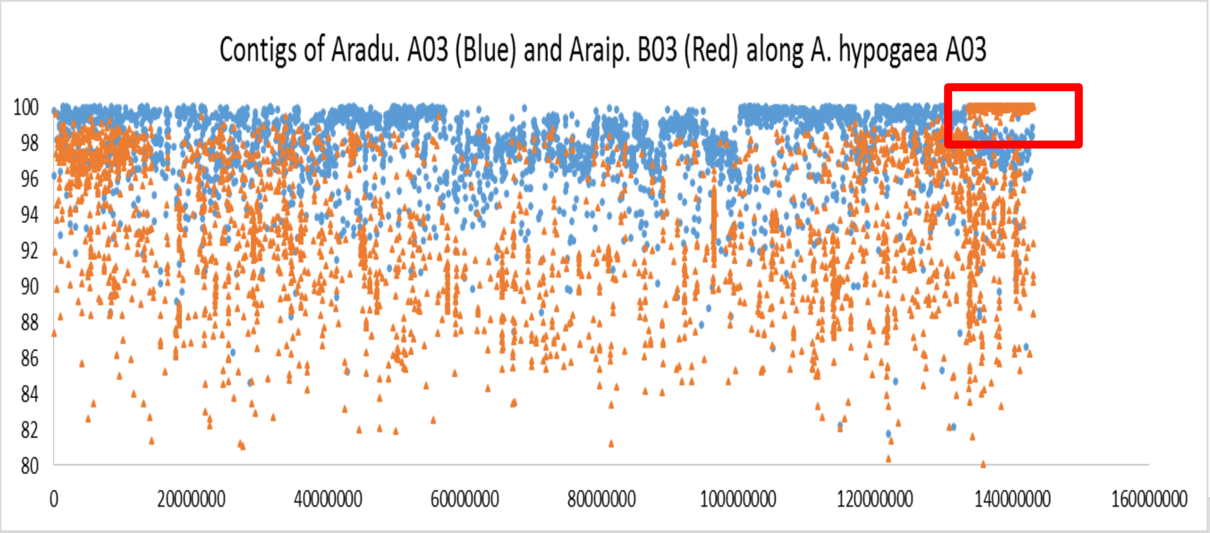
Contigs of Aradu. A03 (Blue) and Araip. B03 (Red) along A. hypogaea A03
100
98
96
94
92
90
88
86
84
82
80
0
20000000
40000000
60000000
80000000
100000000
120000000
140000000
160000000

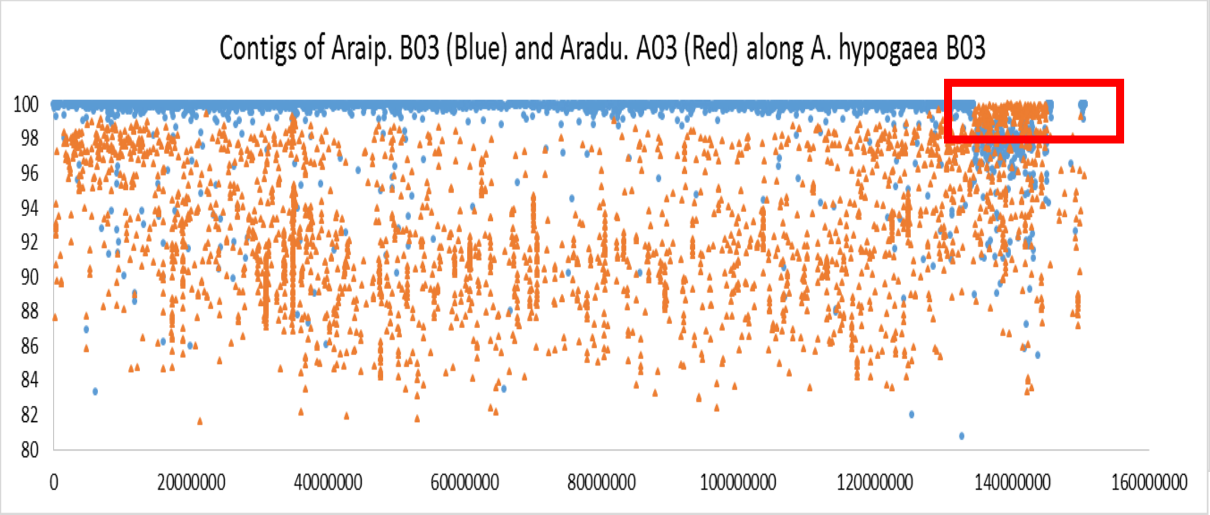
Contigs of Araip. B03 (Blue) and Aradu. A03 (Red) along A. hypogaea B03
100
98
96
94
92
90
88
86
84
82
80
0
20000000
40000000
60000000
80000000
100000000
120000000
140000000
160000000

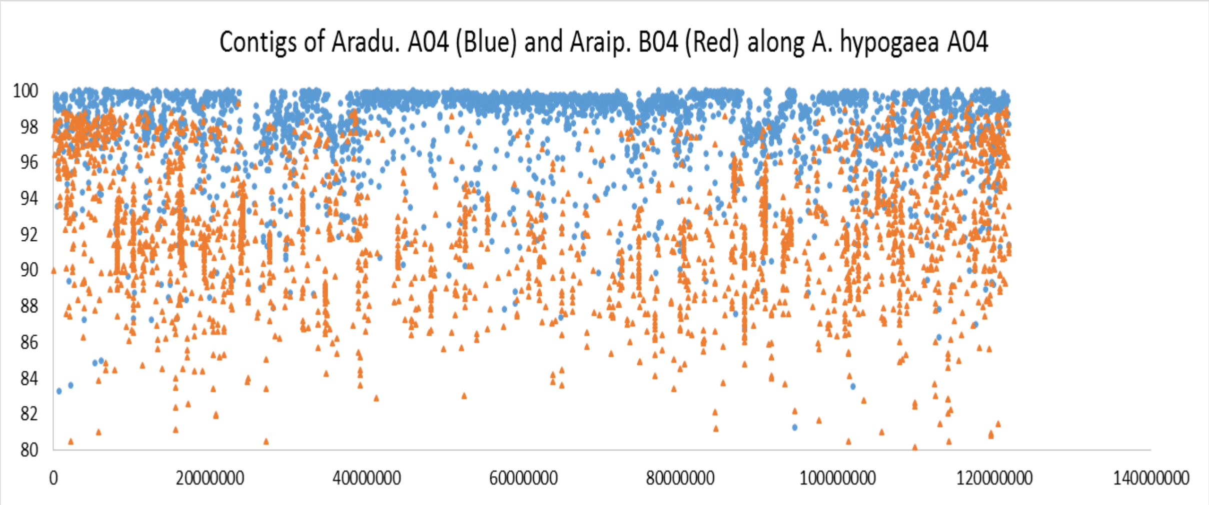

Contigs of Aradu. A04 (Blue) and Araip. B04 (Red) along A. hypogaea A04
100
98
96
94
92
90
88
86
84
82
80
0
20000000
40000000
60000000
80000000
100000000
120000000
140000000

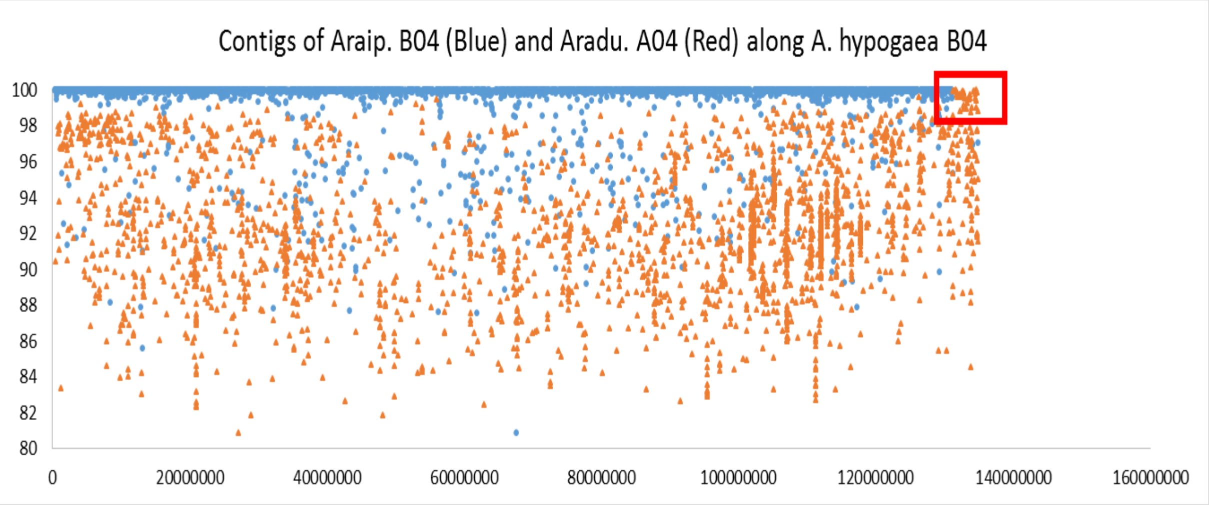

Contigs of Araip. B04 (Blue) and Aradu. A04 (Red) along A. hypogaea B04
100
98
96
94
92
90
88
86
84
82
80
0
20000000
40000000
60000000
80000000
100000000
120000000
140000000
160000000

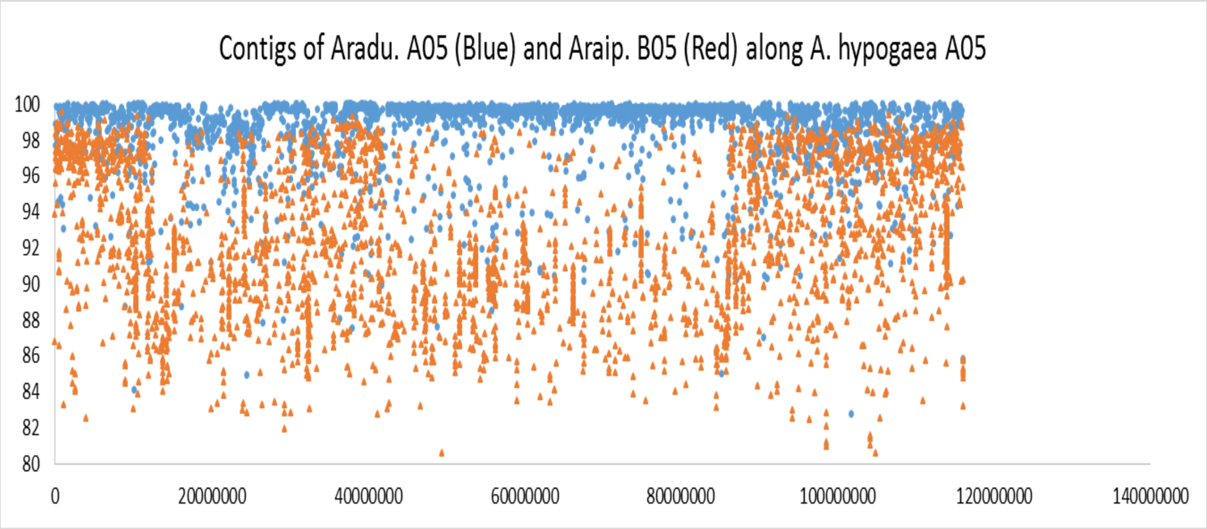
Contigs of Aradu. A05 (Blue) and Araip. B05 (Red) along A. hypogaea A05
100
98
96
94
92
90
88
86
84
82
80
0
20000000
40000000
60000000
80000000
100000000
120000000
140000000

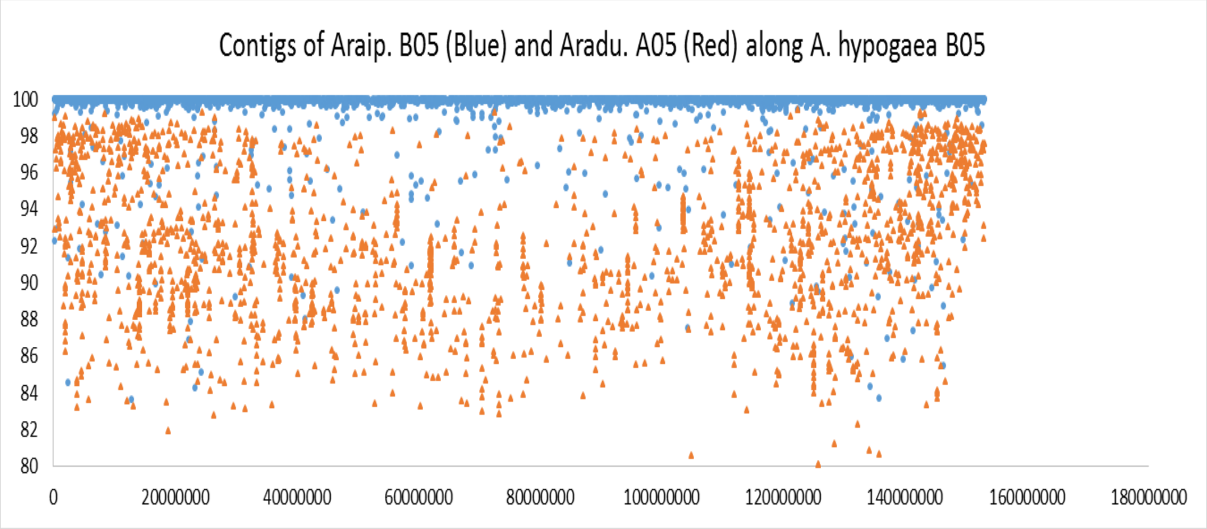
Contigs of Araip. B05 (Blue) and Aradu. A05 (Red) along A. hypogaea B05
100
98
96
94
92
90
88
86
84
82
80
0
20000000
40000000
60000000
80000000
100000000
120000000
140000000
160000000
180000000

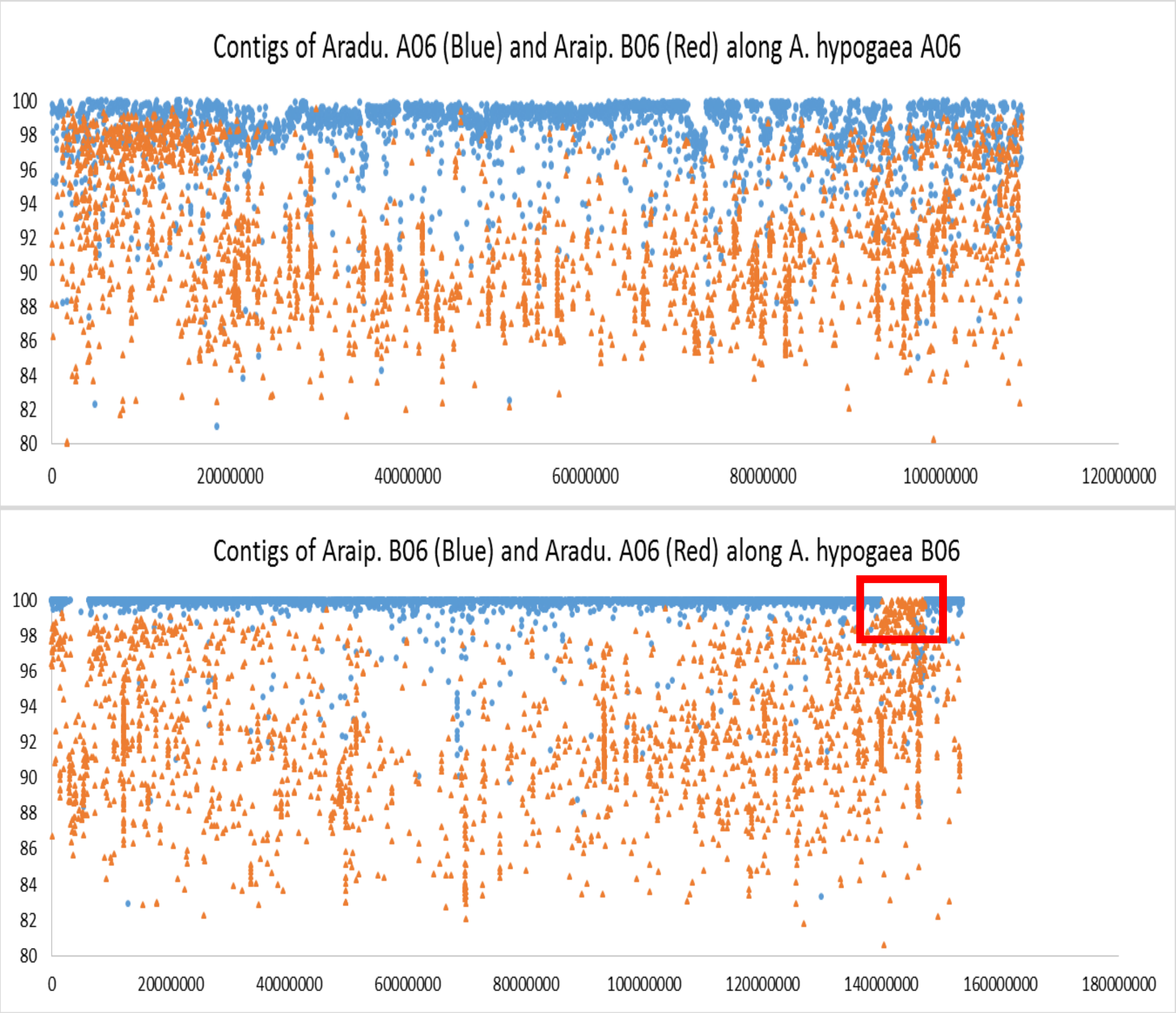
Contigs of Aradu. A06 (Blue) and Araip. B06 (Red) along A. hypogaea A06
100
98
96
94
92
90
88
86
84
82
80
0
20000000
40000000
60000000
80000000
100000000
120000000
Contigs of Araip. B06 (Blue) and Aradu. A06 (Red) along A. hypogaea B06
100
98
96
94
92
90
88
86
84
82
80
0
20000000
40000000
60000000
80000000
100000000
120000000
140000000
160000000
180000000

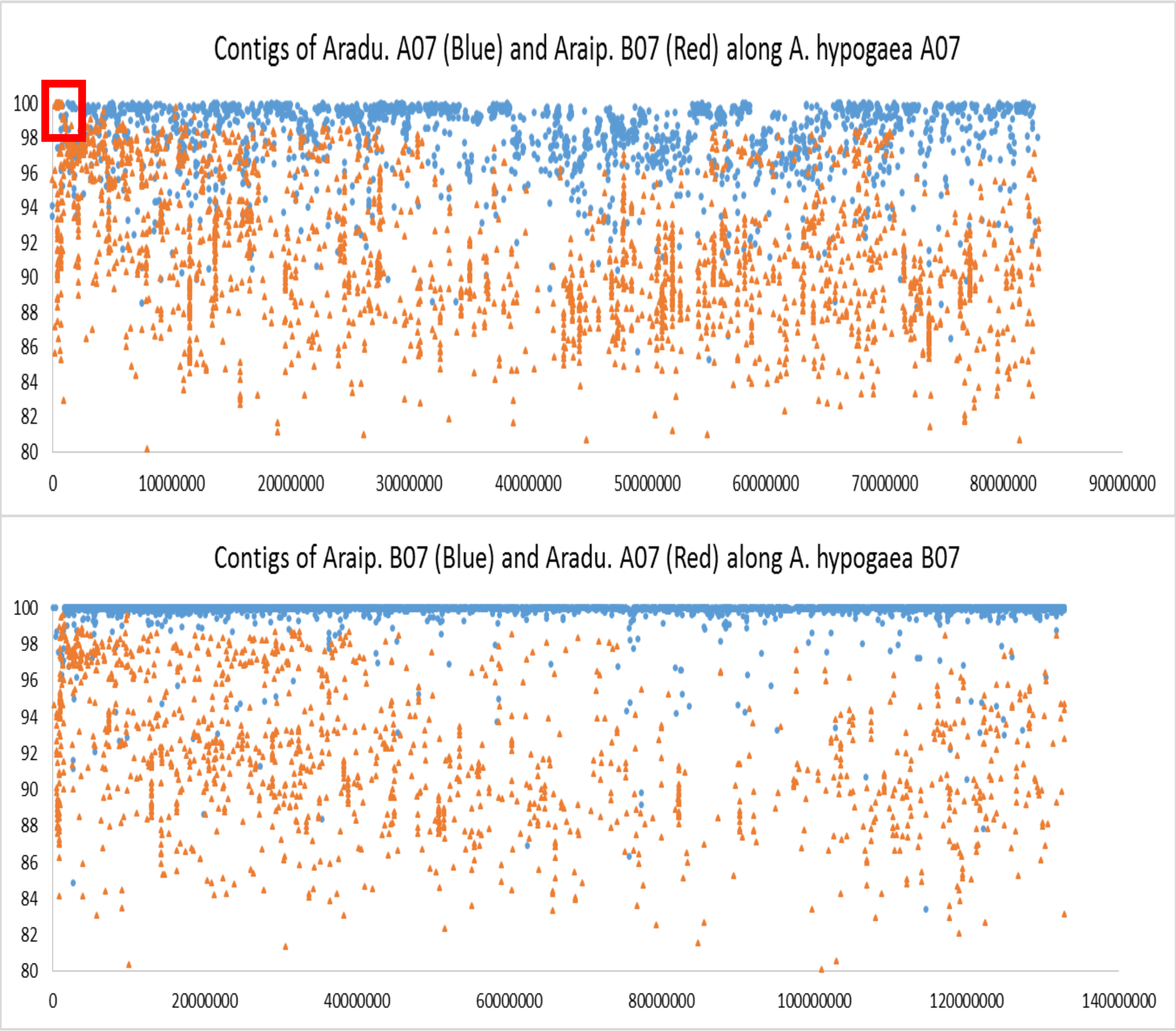
Contigs of Aradu. A07 (Blue) and Araip. B07 (Red) along A. hypogaea A07
100
98
96
94
92
90
88
86
84
82
80
0
10000000
20000000
30000000
40000000
50000000
60000000
70000000
80000000
90000000
Contigs of Araip. B07 (Blue) and Aradu. A07 (Red) along A. hypogaea B07
100
98
96
94
92
90
88
86
84
82
80
0
20000000
40000000
60000000
80000000
100000000
120000000
140000000

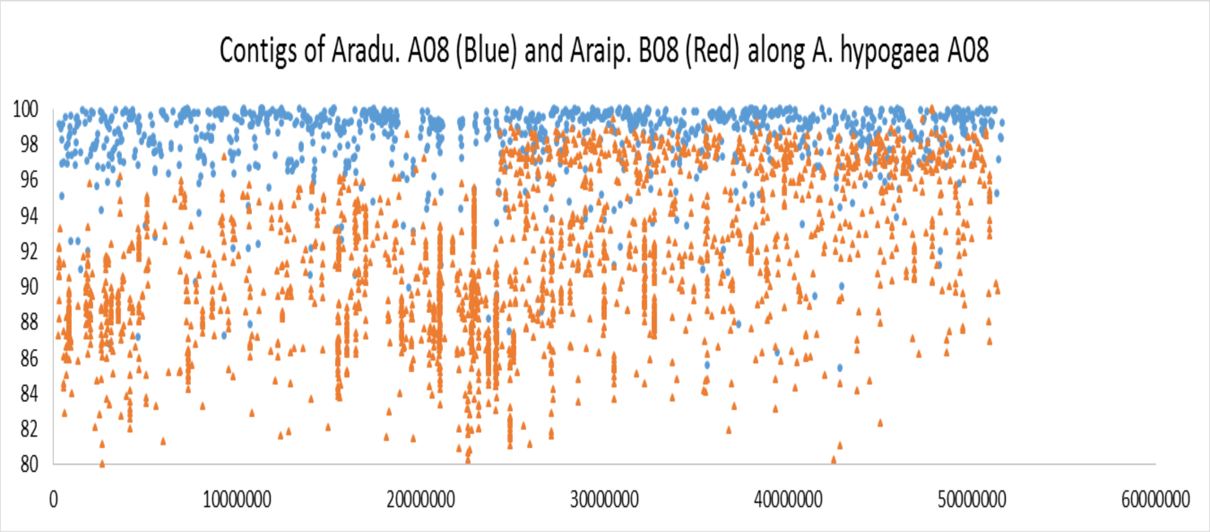
Contigs of Aradu. A08 (Blue) and Araip. B08 (Red) along A. hypogaea A08
100
98
96
94
92
90
88
86
84
82
80
0
10000000
20000000
30000000
40000000
50000000
60000000

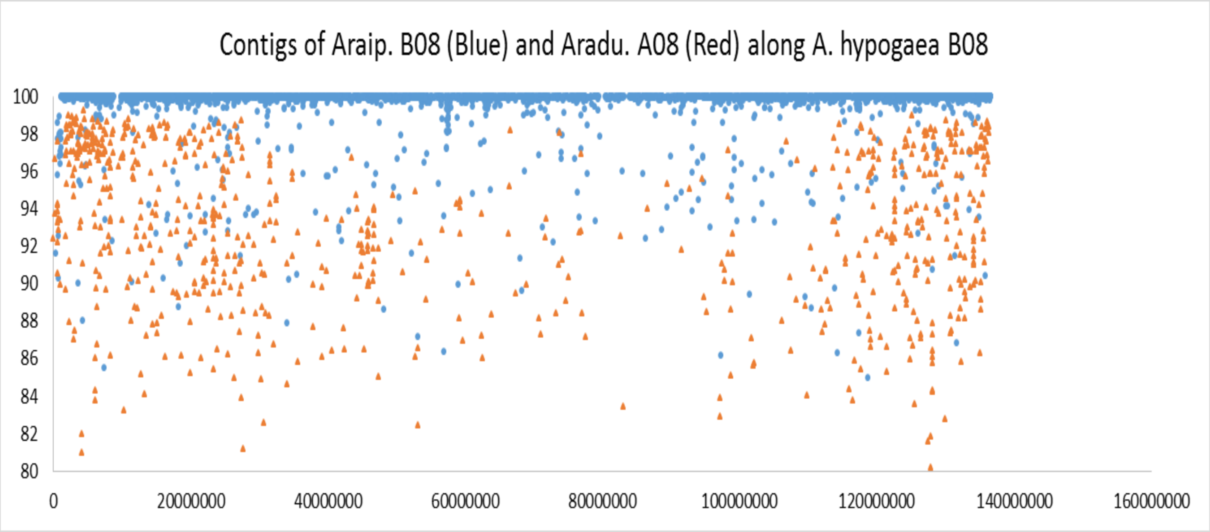
Contigs of Araip. B08 (Blue) and Aradu. A08 (Red) along A. hypogaea B08
100
98
96
94
92
90
88
86
84
82
80
0
20000000
40000000
60000000
80000000
100000000
120000000
140000000
160000000

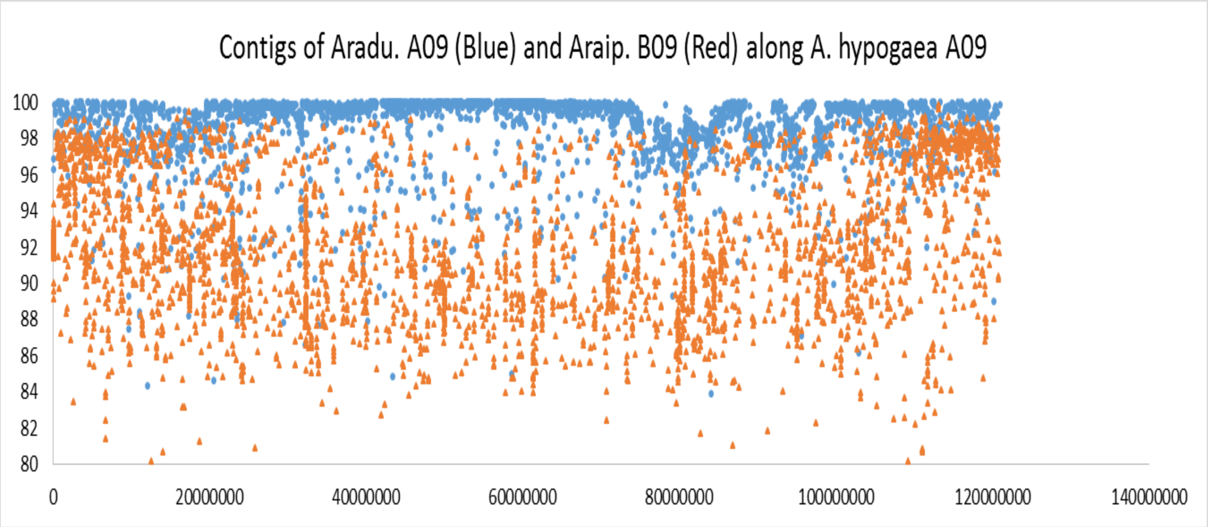
Contigs of Aradu. A09 (Blue) and Araip. B09 (Red) along A. hypogaea A09
100
98
96
94
92
90
88
86
84
82
80
0
20000000
40000000
60000000
80000000
100000000
120000000
140000000

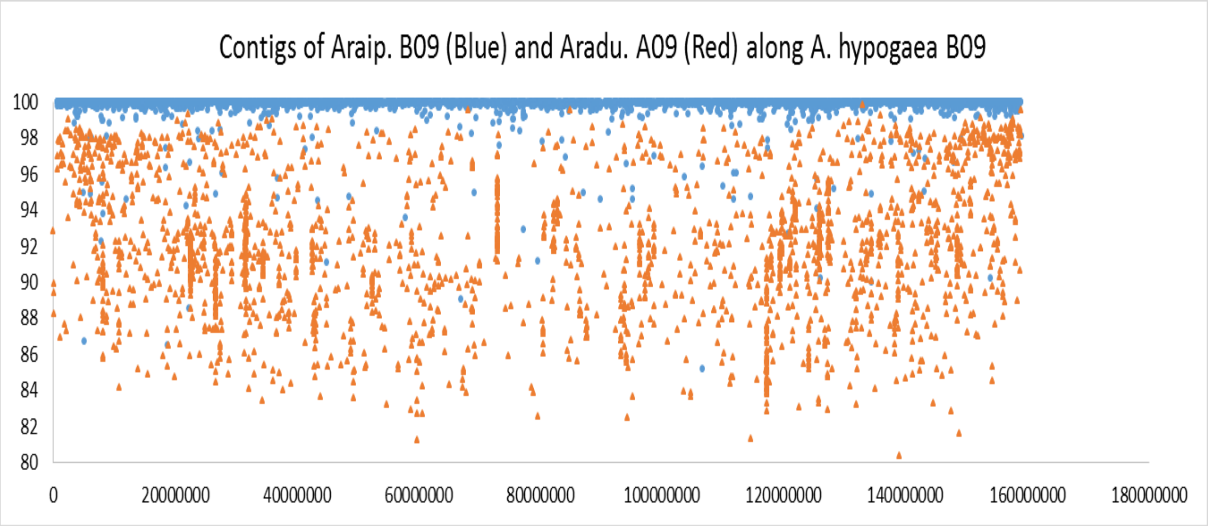
Contigs of Araip. B09 (Blue) and Aradu. A09 (Red) along A. hypogaea B09
100
98
96
94
92
90
88
86
84
82
80
0
20000000
40000000
60000000
80000000
100000000
120000000
140000000
160000000
180000000

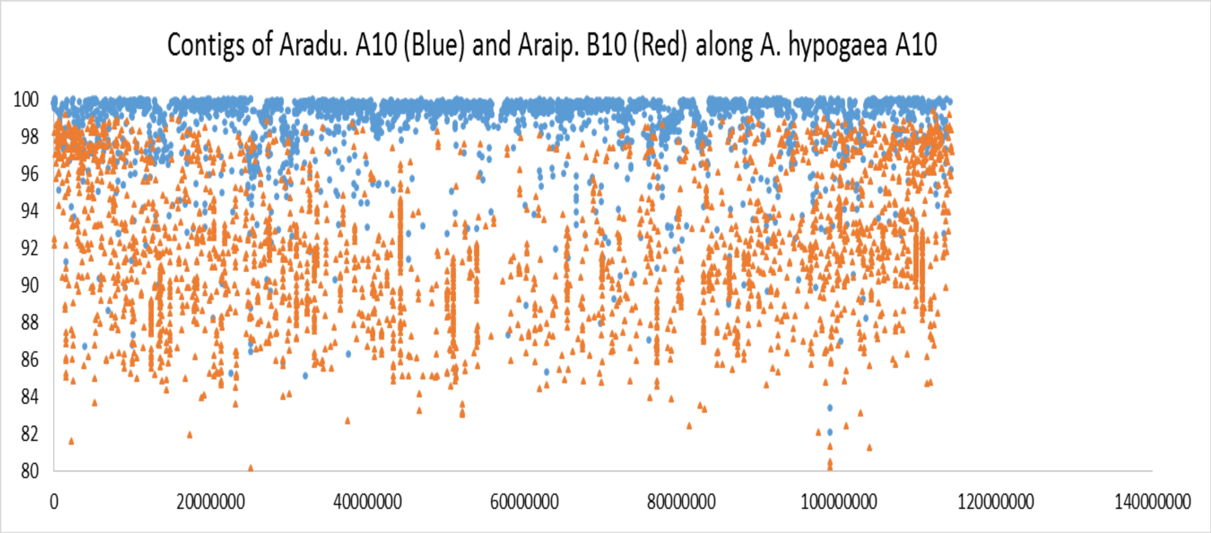
Contigs of Aradu. A10 (Blue) and Araip. B10 (Red) along A. hypogaea A10
100
98
96
94
92
90
88
86
84
82
80
0
20000000
40000000
60000000
80000000
100000000
120000000
140000000

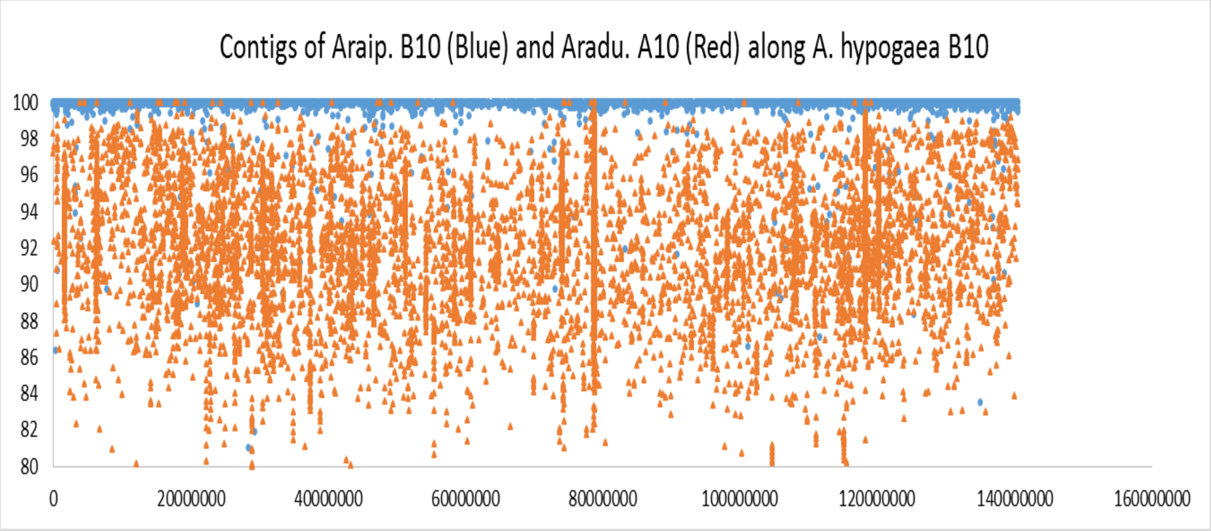
Contigs of Araip. B10 (Blue) and Aradu. A10 (Red) along A. hypogaea B10
100
98
96
94
92
90
88
86
84
82
80
0
20000000
40000000
60000000
80000000
100000000
120000000
140000000
160000000

Supplementary Data Set 8.   Karyotype evolution of peanut genome.

a

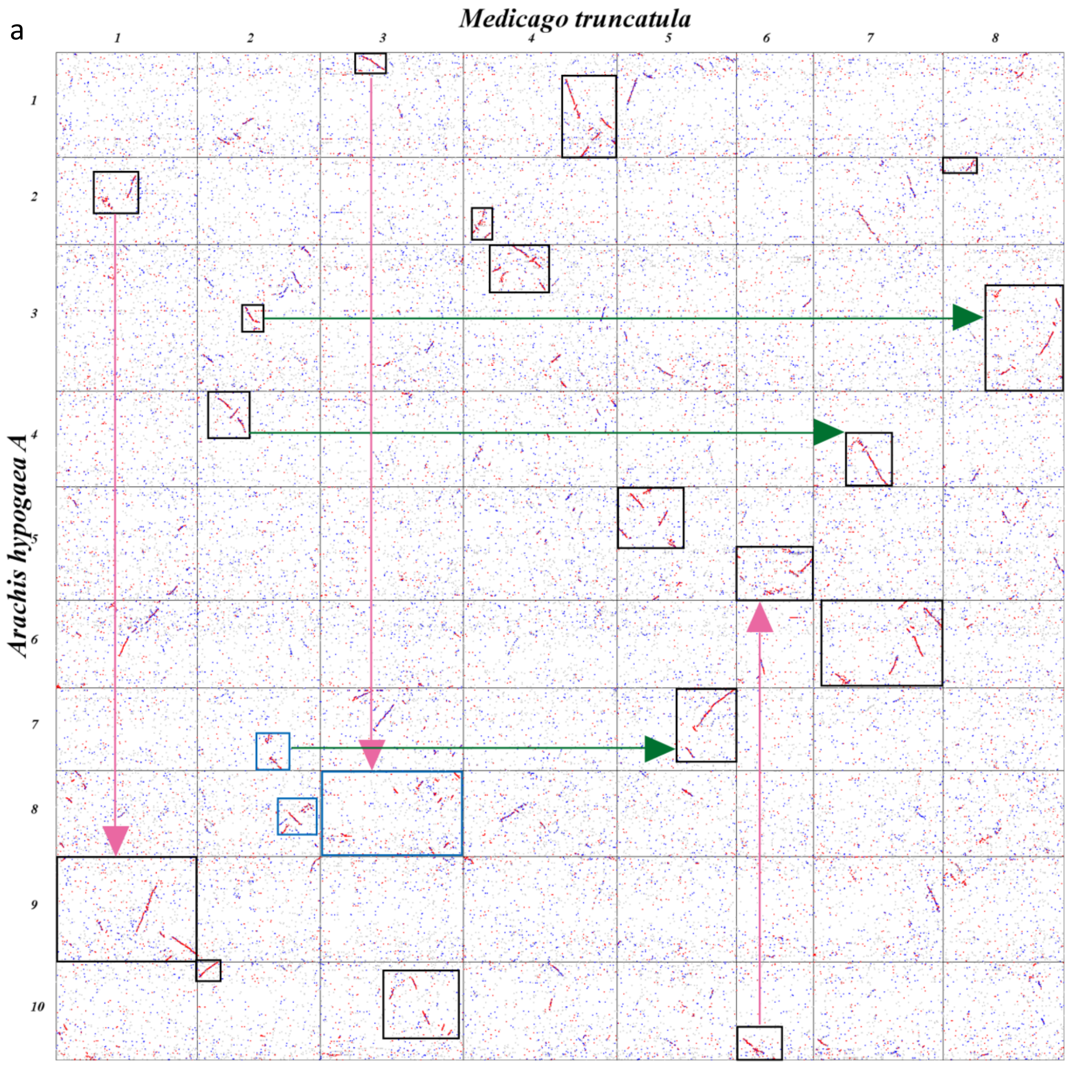

a. Dotplot between *Medicago truncatula* and peanut A sugenome.

Medicago and peanut A subgenome chromosomes are aligned horizontally and vertically, respectively. Homologous gene pairs are shown in red, blue, and gray to denote the best, second-best, and other matches, respectively. Best-matched or orthologous regions were marked out by solid-line rectangles. Arrows show complement correspondence produced by chromosome breakages during evolution.

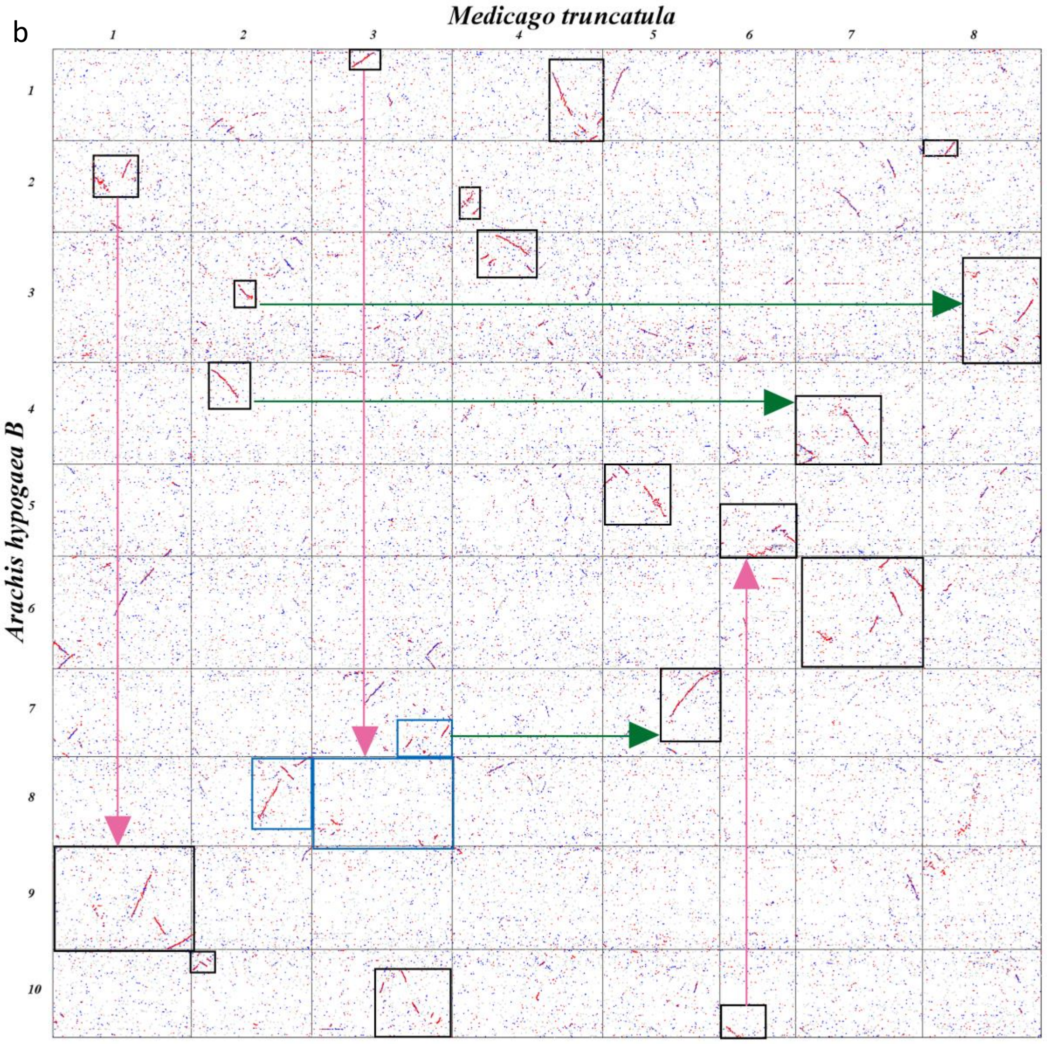

b. **Dotplot between *Medicago truncatula* and peanut B sugenome.**
Medicago and peanut B subgenome chromosomes are aligned horizontally and vertically, respectively. Homologous gene pairs are shown in red, blue, and gray to denote the best, second-best, and other matches, respectively. Best-matched or orthologous regions were marked out by solid-line rectangles. Arrows show complement correspondence produced by chromosome breakages during evolution.

c

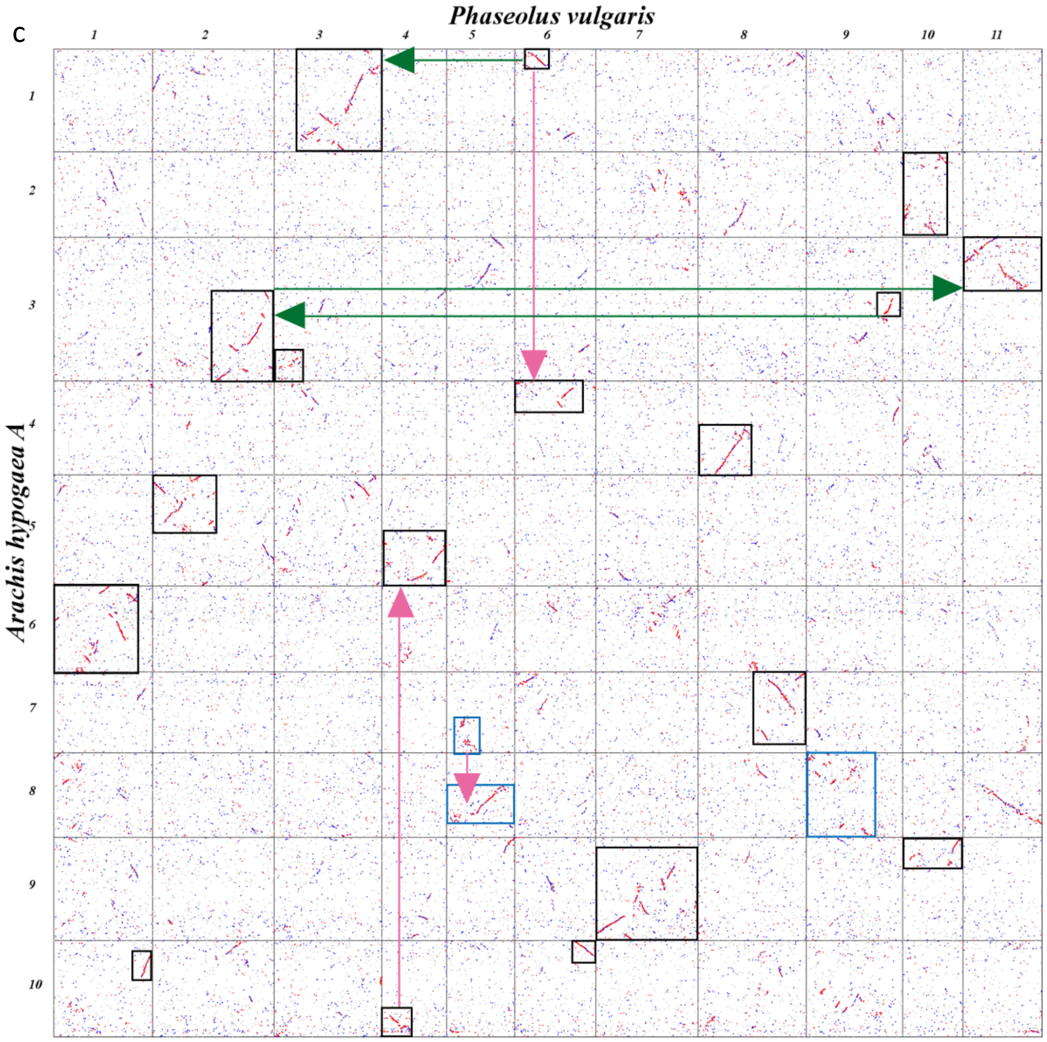

c. **Dotplot between *Phaseolus vulgaris* and peanut A sugenome.**
Medicago and peanut A subgenome chromosomes are aligned horizontally and vertically, respectively. Homologous gene pairs are shown in red, blue, and gray to denote the best, second-best, and other matches, respectively. Best-matched or orthologous regions were marked out by solid-line rectangles. Arrows show complement correspondence produced by chromosome breakages during evolution.

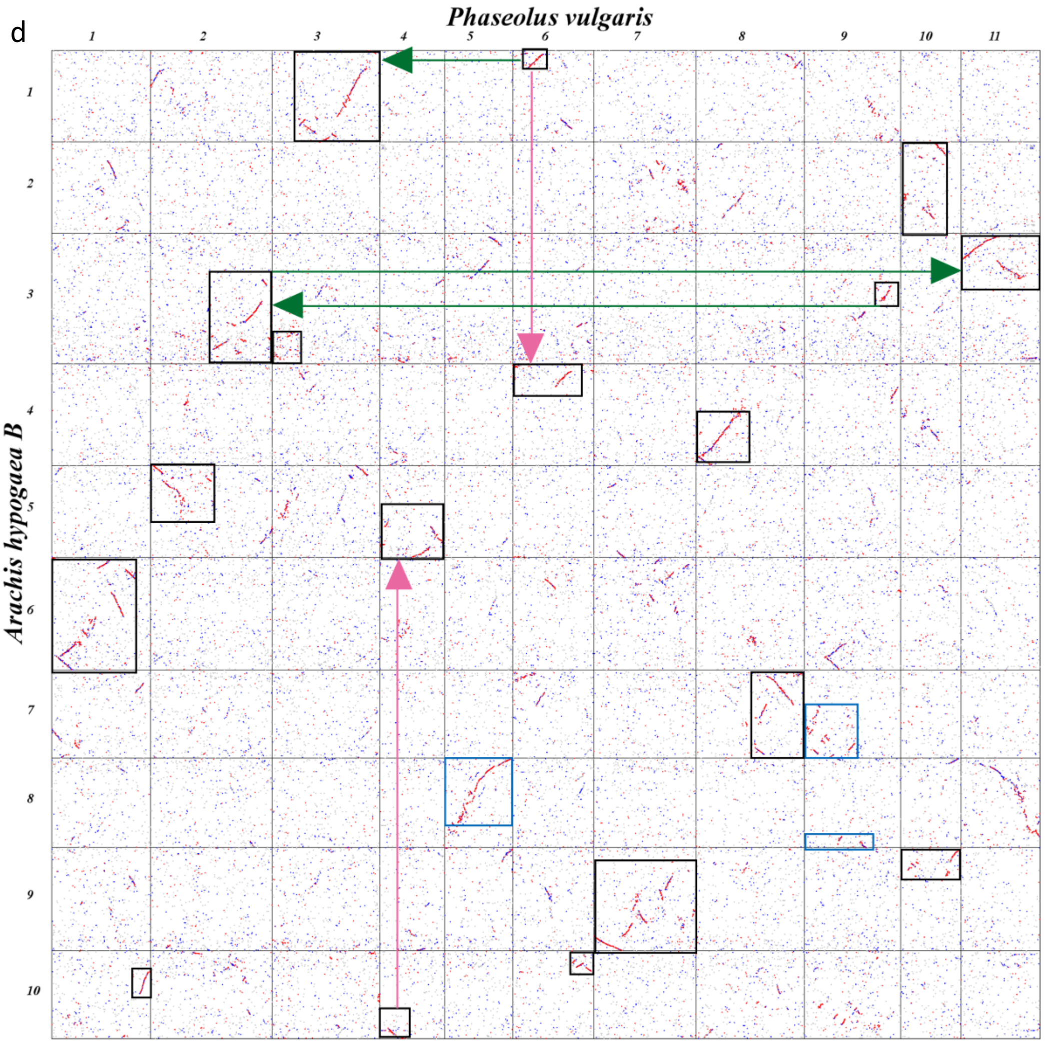

d. **Dotplot between *Phaseolus vulgaris* and peanut B sugenome.**

Medicago and peanut B subgenome chromosomes are aligned horizontally and vertically, respectively. Homologous gene pairs are shown in red, blue, and gray to denote the best, second-best, and other matches, respectively. Best-matched or orthologous regions were marked out by solid-line rectangles. Arrows show complement correspondence produced by chromosome breakages during evolution.

Evolution of peanut B genome

e

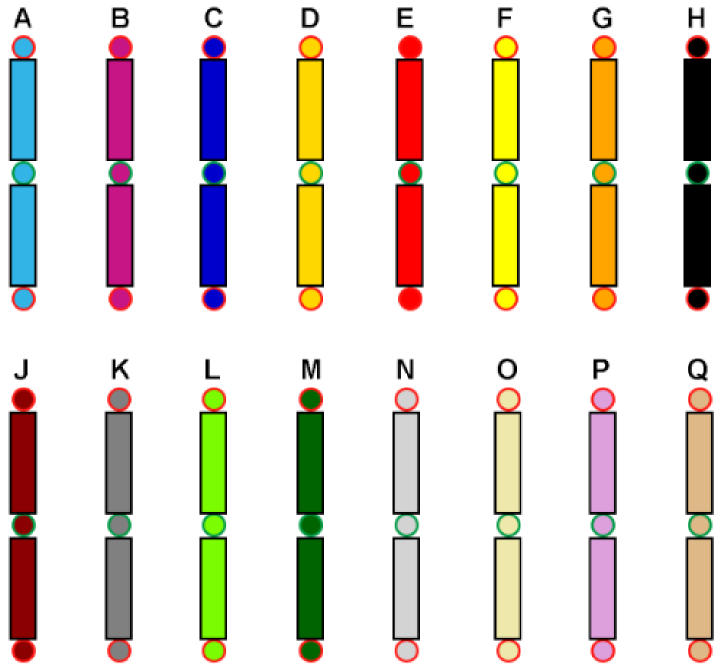

e. **Ancestral legume chromosomes before species differentiation.**
Color bars, chromosomes; red circles, telomeres; green circles, centromeres.

f

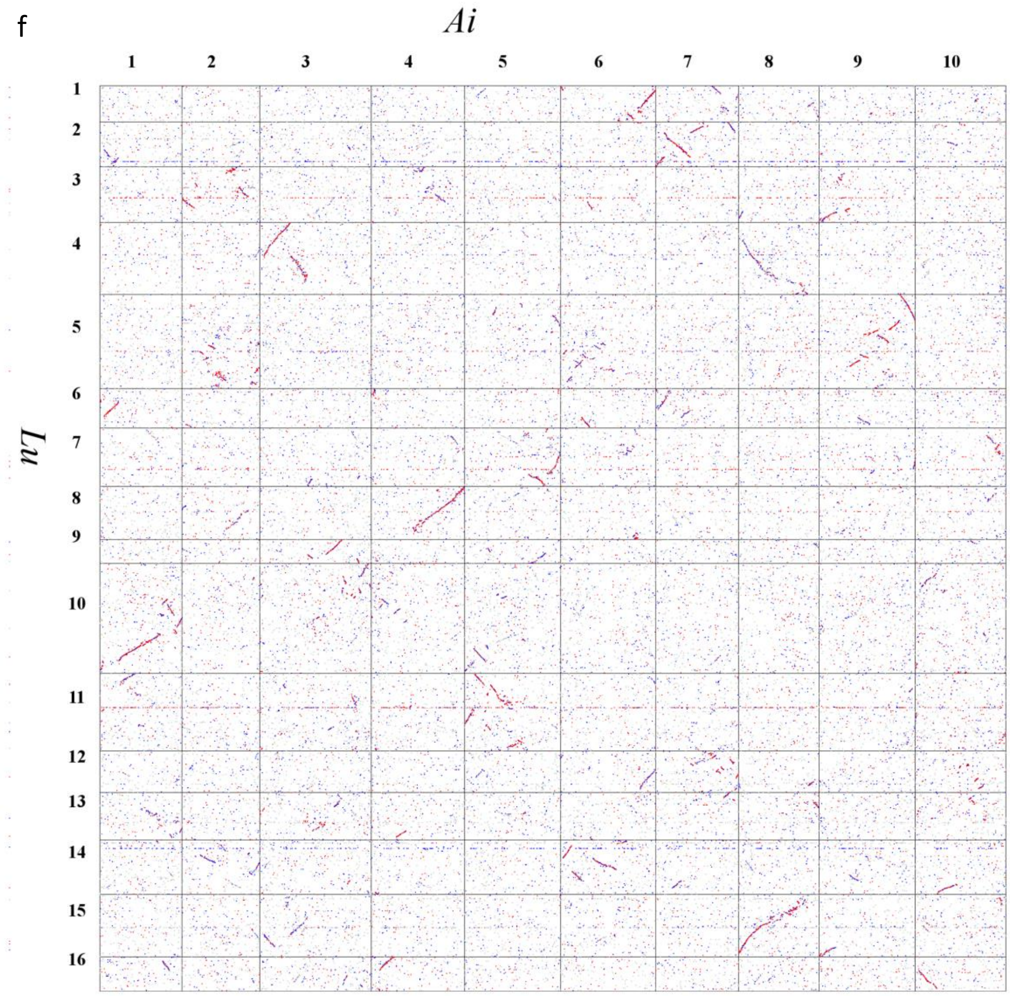

f. **Dotplots between *A. ipaensis* and Lu (legume basic chromosome after LCT).**
A. ipaensis and Lu chromosomes are aligned horizontally and vertically, respectively. Homologous gene pairs are shown in red, blue, and gray to denote the best, second-best, and other matches, respectively.

g

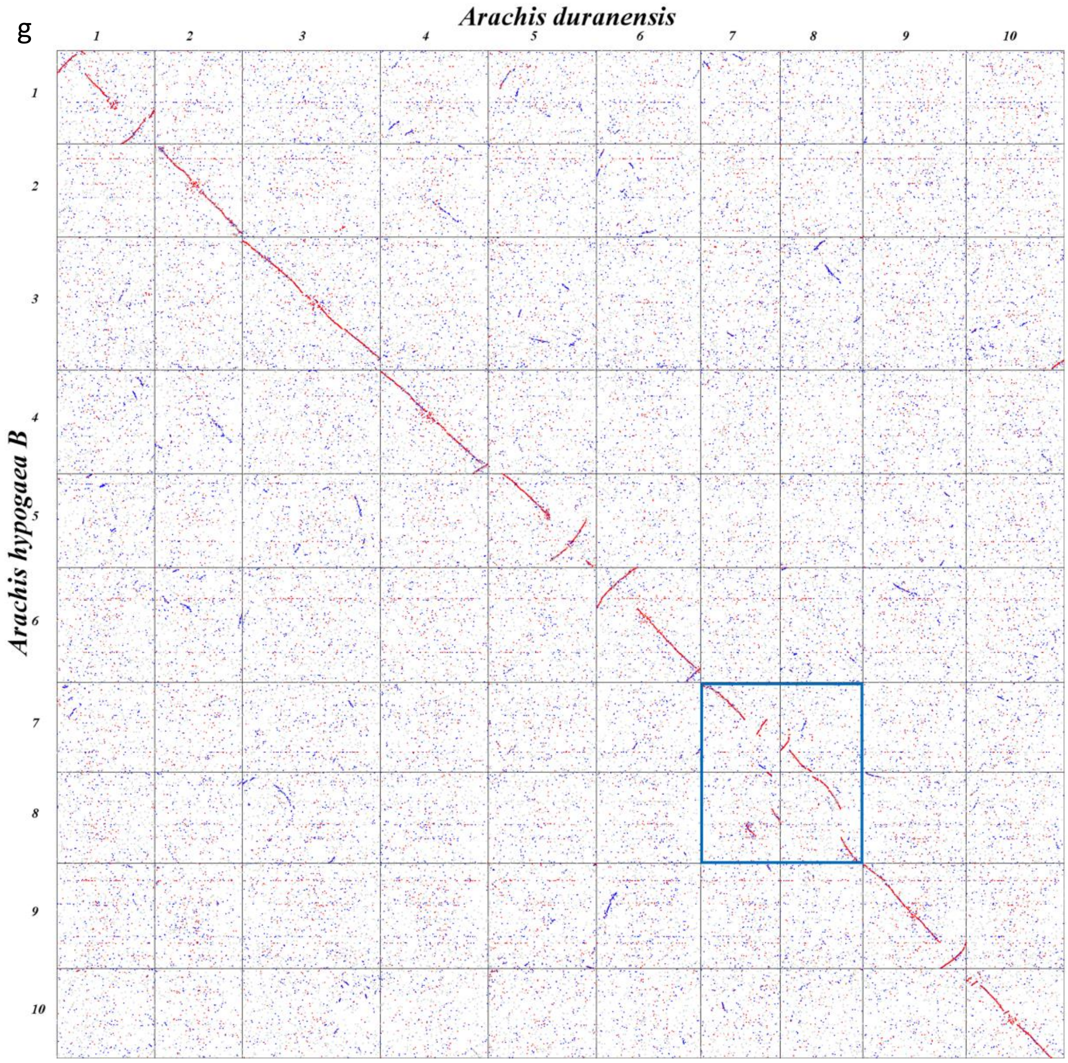

g. **Dotplot between *A. duranensis* and peanut B sugenome.**

A. duranensis and peanut B subgenome chromosomes are aligned horizontally and vertically, respectively. Homologous gene pairs are shown in red, blue, and gray to denote the best, second-best, and other matches, respectively.

h

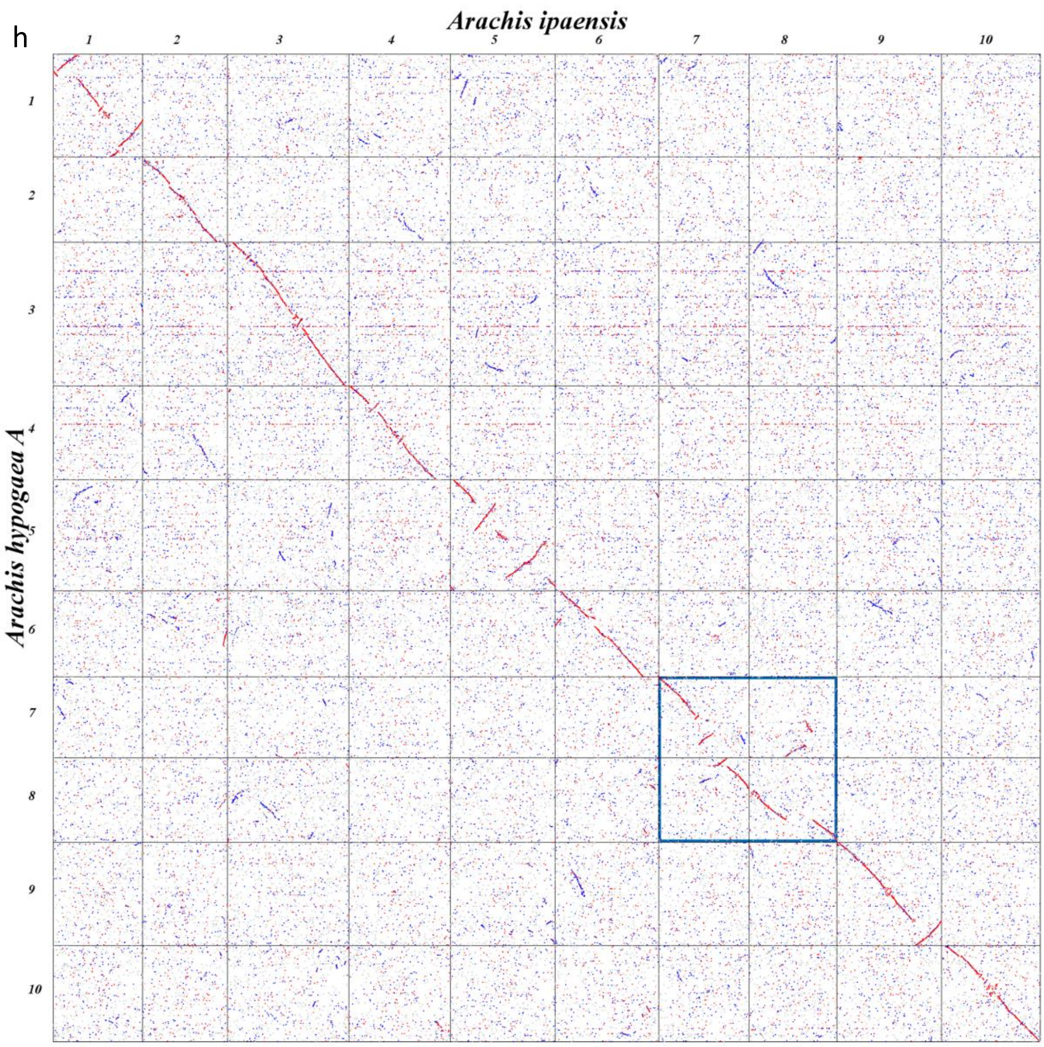

h. **Dotplot between *A. ipaensis* and peanut A sugenome.**

A. ipaensis and peanut A subgenome chromosomes are aligned horizontally and vertically, respectively. Homologous gene pairs are shown in red, blue, and gray to denote the best, second-best, and other matches, respectively.

i

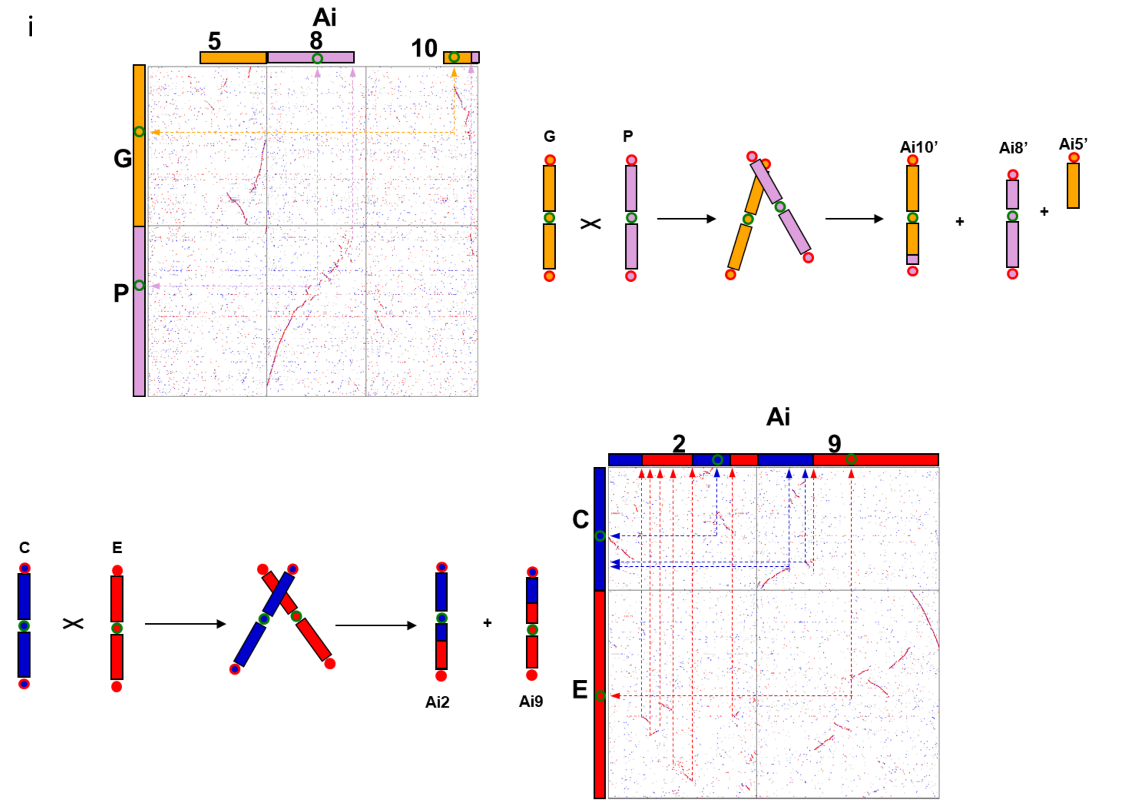

i. Partial chromosome evolution in *A. ipaensis*.
Chromosomes, shown as rectangular blocks, are arranged horizontally and vertically to the dot-plot. The color scheme for the chromosomes mainly follows ancestral legume chromosomes before species differentiation. Homologous gene pairs are shown in red, blue, and gray to denote the best, second-best, and other matches, respectively. Dashed lines, with colors corresponding to chromosome color schemes, help to show where the merging points were and whether centromeres were preserved or not.

j Evolution of Peanut genome

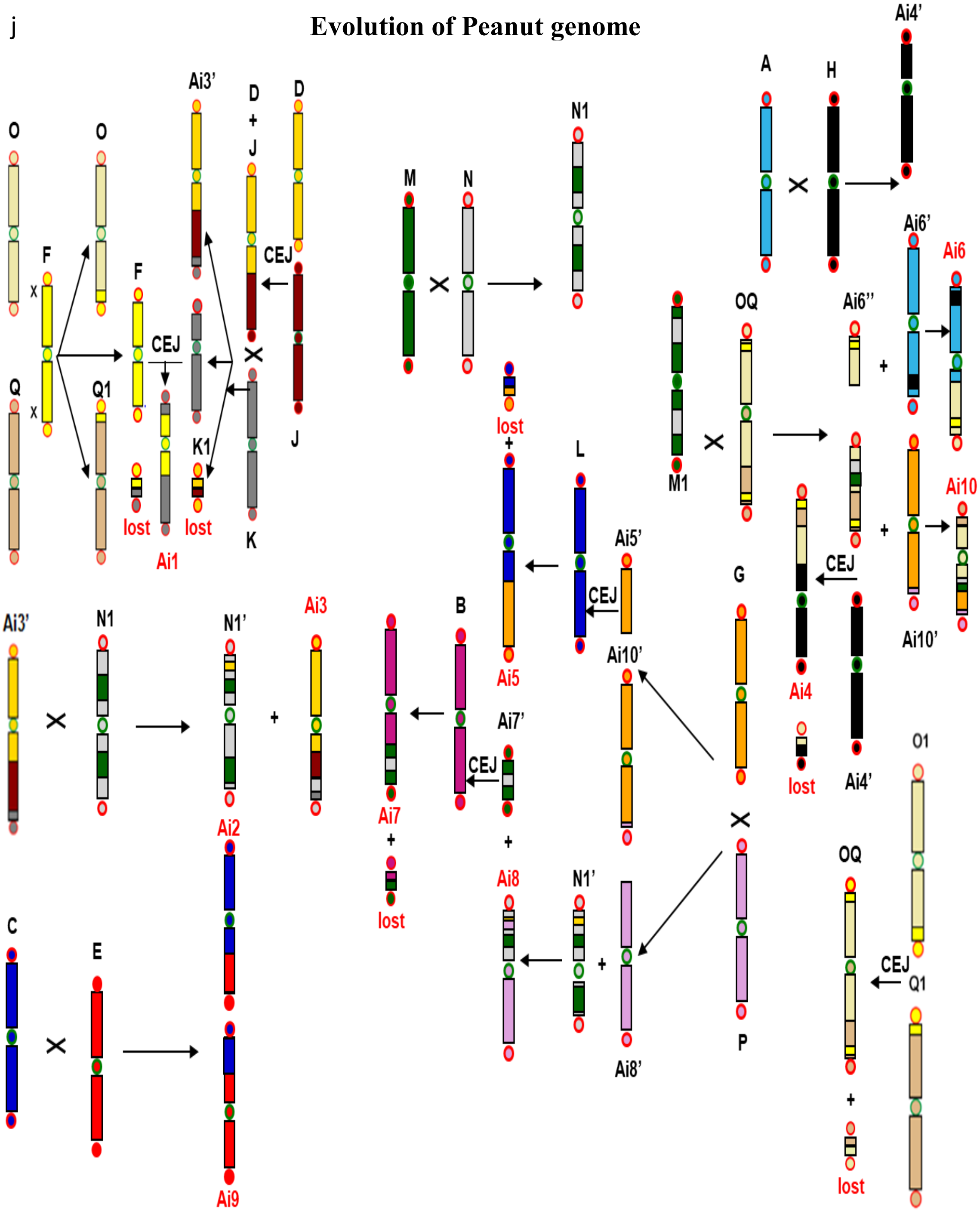

j. **Karyotype evolution of peanut genome.**
Color bars, chromosomes; red circles, telomeres; green circles, centromeres; CEJ, chromosome end-end joining; X: a crossing-over. The color scheme for the chromosomes mainly follows ancestral legume chromosomes before species differentiation named by A-Q.

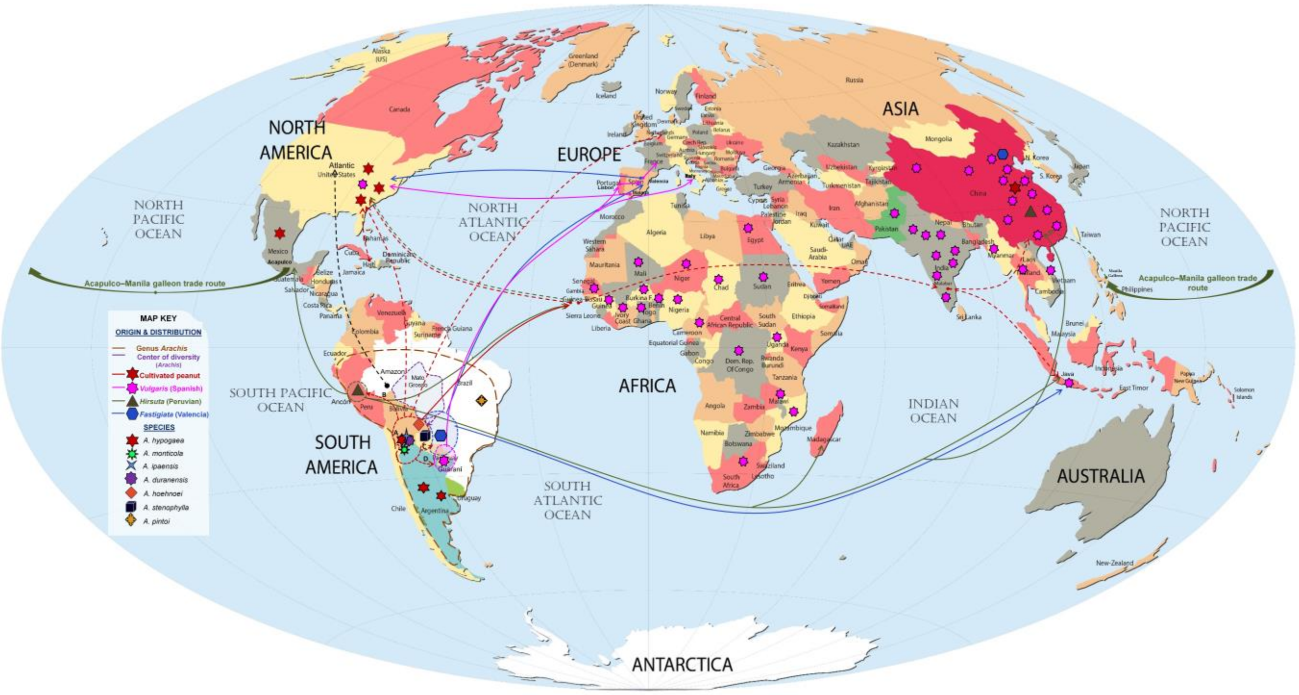

Supplementary Data Set 14. Worldwide distribution of peanut species and their dissemination routes. The genus Arachis originated in southwestern Mato Grosso do Sul region of Brazil (South America) and probably moved to the region southern Bolivia - northern Argentina. The putative A and B genome donors were found in this area, where hybridization and domestication occurred, and then further domesticated in different places and dispersed to other regions. Peanut Peruvian type (*hirsute*) originated in Ancon, Peru; Spanish type (*vulgaris*) in Guarani area while Valencia type (*var. fastigiata*) in Paraguay - central Brazil, from where major peanut types were distributed to different regions of the world. Distribution, abundance and cultivation of major peanut types and wild species are shown by arrows and symbols in their respective areas. The solid line shows documented distribution while a partial line represents hypothesized dispersal.

References

86. Zhang G., et al. Hybrid de novo genome assembly of the Chinese herbal plant danshen (*Salvia miltiorrhiza* Bunge). Gigascience 4, 62 (2015).
87. Bolger A.M., Lohse M.& Usadel B. Trimmomatic: a flexible trimmer for Illumina sequence data. Bioinformatics 15, 2114-2120 (2014).
88. Li H. & Durbin R: Fast and accurate short read alignment with Burrows-Wheeler transform. Bioinformatics 25, 1754-1760(2009).
89. Walker B.J. et al. Pilon: an integrated tool for comprehensive microbial variant detection and genome assembly improvement. PLoS One, 9,11:e112963 (2014).
90. Servant N. et al. HiC-Pro: an optimized and flexible pipeline for Hi-C data processing. Genome Biol 16,259 (2015).
91. Li H., et al. Genome Project Data Processing S: The sequence alignment/map format and SAMtools. Bioinformatics 25, 2078-2079 (2009).
92. Kent W.J. BLAT--the BLAST-like alignment tool. Genome Res 12, 656-664 (2002).
93. Patel R.K. & Jain M. NGS QC Toolkit: a toolkit for quality control of next generation sequencing data. PLoS One 7, 2:e30619 (2012).
94. Gordon S.P. et al. Widespread polycistronic transcripts in fungi revealed by single-molecule mRNA sequencing. PLoS One 10, 7:e0132628 (2015).
95. Salmela L. & Rivals E. LoRDEC: accurate and efficient long read error correction. Bioinformatics 30, 3506-3514 (2014).
96. Wu T. & Watanabe C. GMAP: a genomic mapping and alignment program for mRNA and EST sequences. Bioinformatics 21, 1859-1875 (2005).
97. Korf I. Gene finding in novel genomes. BMC Bioinformatics 5, 59 (2004).
98. Besemer J. & Borodovsky M. GeneMark: web software for gene finding in prokaryotes, eukaryotes and viruses. Nucleic Acids Res 33, 451-454 (2005).
99. Kim D., Langmead B. & Salzberg S.L. HISAT: a fast spliced aligner with low memory requirements. Nat Methods 12, 357-360 (2015).
100. Pertea M. et al. StringTie enables improved reconstruction of a transcriptome from RNA-seq reads. Nat Biotechnol 33, 290-295(2015).
101. Trapnell C. et al. Differential gene and transcript expression analysis of RNA-seq experiments with TopHat and Cufflinks. Nat Protoc 7, 562-578 (2012).
102. Campbell M.S., Holt C., Moore B. & Yandell M. Genome annotation and curation using MAKER and MAKER-P. Curr Protoc Bioinformatics 48, 11-39 (2014).
103. Ogata H. et al. KEGG: Kyoto Encyclopedia of Genes and Genomes. Nucleic Acids Res 27, 29-34 (1999).
104. Bairoch A.& Apweiler R.The SWISS-PROT protein sequence database and its supplement TrEMBL in 2000. Nucleic Acids Res 1, 45-48 (2000).
105. Zdobnov E.M.& Apweiler R. InterProScan--an integration platform for the signature-recognition methods in InterPro. Bioinformatics 9, 847-848 (2001).
106. Nawrocki E.P., Kolbe D.L.& Eddy S.R. Infernal 1.0: inference of RNA alignments. Bioinformatics 10, 1335-1337 (2009).
107. Lowe T.M.& Eddy S.R. tRNAscan-SE: a program for improved detection of transfer RNA genes in genomic sequence. Nucleic Acids Res 5, 955-964 (1997).
108. Wang X. et al. Statistical inference of chromosomal homology based on gene colinearity and applications to

Arabidopsis and rice. BMC Bioinformatics 7, 447(2006).
109. Bowers J.E., Chapman B.A., Rong J. & Paterson A.H. Unravelling angiosperm genome evolution by phylogenetic analysis of chromosomal duplication events. Nature 6930, 433-438 (2003).
110. Wang X. et al. Telomere-centric genome repatterning determines recurring chromosome number reductions during the evolution of eukaryotes. New Phytol 1, 378-389(2015).
111. Pandey M.K. et al. Identification of QTLs associated with oil content and mapping FAD2 genes and their relative contribution to oil quality in peanut (*Arachis hypogaea* L.). BMC Genet 15,133 (2014).
112. Langmead B., Trapnell C., Pop M. & Salzberg S.L. Ultrafast and memory-efficient alignment of short DNA sequences to the human genome. Genome Biol 10, R25 (2009).
113. Ernst J. & Bar-Joseph Z. STEM: a tool for the analysis of short time series gene expression data. BMC Bioinformatics 7,191(2006).
114. Qiao Z., Pingault L., Nourbakhsh-Rey M. & Libault M. Comprehensive comparative genomic and transcriptomic analyses of the legume genes controlling the nodulation process. Front Plant Sci 7,34 (2016).
115. Peng Z. et al. Transcriptome profiles reveal gene regulation of peanut (*Arachis hypogaea* L.) nodulation. Sci Rep 7, 40066 (2017).
116. Fischer S. et al. Using OrthoMCL to assign proteins to OrthoMCL-DB groups or to cluster proteomes into new ortholog groups. Curr Protoc Bioinformatics 6, Unit 6 12 11-19 (2011).
117. McKenna A. et al. The Genome Analysis Toolkit: a MapReduce framework for analyzing next-generation DNA sequencing data. Genome Res 9, 1297-1303 (2010).
118. Clevenger J.P., Korani W., Ozias-Akins P. & Jackson S. Haplotype-based genotyping in polyploids. Front Plant Sci 9, 564 (2018).
119. Zhang J. et al. High-density genetic map construction and identification of a locus controlling weeping trait in an ornamental woody plant (Prunus mume Sieb. et Zucc). DNA Res 22, 183-191(2015).
120. Vinod K.K. Kosambi and the genetic mapping function. Resonance 16, 540-550 (2011).
121. Takagi H. et al. QTL-seq: rapid mapping of quantitative trait loci in rice by whole genome resequencing of DNA from two bulked populations. Plant J 74, 174-183 (2013).
122. Pandey M.K. et al. Genetic dissection of novel QTLs for resistance to leaf spots and Tomato spotted wilt virus in peanut (*Arachis hypogaea* L.). Front Plant Sci 8, 25 (2017).
123. Cingolani P. et al. A program for annotating and predicting the effects of single nucleotide polymorphisms, SnpEff: SNPs in the genome of *Drosophila melanogaster* strain w1118; iso-2; iso-3. Fly (Austin) 6, 80-92 (2012).
124. Boogerd, F.C. and van Rossum, D. Nodulation of Groundnut by Bradyrhizobium: A Simple Infection Process by Crack Entry. FEMS Microbiology Reviews, 21, 5-27 (1997).
125. Ma, J. & Bennetzen, J.L. Rapid recent growth and divergence of rice nuclear genomes. Proc Natl Acad Sci U S A 101, 12404-10 (2004).
126. Phillip, S.M. et al. The paleontology of intergene retrotransposons of maize. Nature Genetics 20, 43-45 (1998).
127. Zheng, X. et al. A high-performance computing toolset for relatedness and principal component analysis of SNP data. Bioinformatics, 28, 3326-3328 (2012).
128. Alexander, D.H. et al. Fast model-based estimation of ancestry in unrelated individuals. Genome Research, 19, 1655–1664 (2009).
129. Danecek, P. et al. 2011. The variant call format and VCFtools. Bioinformatics, 27(15), pp.2156-2158.
