# Supplementary material for: The genome of cultivated peanut provides insight into legume karyotypes, polyploid evolution and crop domestication
Source: Nat Genet. 2019 May 1;51(5):865–76. doi: 10.1038/s41588-019-0402-2 (PMC7188672; doi:10.1038/s41588-019-0402-2)
Supplement: Supplementary file 2 — Reporting Summary [file 41588_2019_402_MOESM2_ESM.pdf]

nature research

Corresponding author(s): Weijian Zhuang; Rajeev K Varshney; Ray Ming; Xiyin Wang

Last updated by author(s): 05032019

Reporting Summary

Nature Research wishes to improve the reproducibility of the work that we publish. This form provides structure for consistency and transparency in reporting. For further information on Nature Research policies, see Authors & Referees and the Editorial Policy Checklist.

Statistics

For all statistical analyses, confirm that the following items are present in the figure legend, table legend, main text, or Methods section.

n/a	Confirmed	
☐	☒	The exact sample size (n) for each experimental group/condition, given as a discrete number and unit of measurement
☒	☐	A statement on whether measurements were taken from distinct samples or whether the same sample was measured repeatedly
☒	☐	The statistical test(s) used AND whether they are one- or two-sided<br>*Only common tests should be described solely by name; describe more complex techniques in the Methods section.*
☒	☐	A description of all covariates tested
☒	☐	A description of any assumptions or corrections, such as tests of normality and adjustment for multiple comparisons
☒	☐	A full description of the statistical parameters including central tendency (e.g. means) or other basic estimates (e.g. regression coefficient) AND variation (e.g. standard deviation) or associated estimates of uncertainty (e.g. confidence intervals)
☒	☐	For null hypothesis testing, the test statistic (e.g. F, t, r) with confidence intervals, effect sizes, degrees of freedom and P value noted<br>*Give P values as exact values whenever suitable.*
☒	☐	For Bayesian analysis, information on the choice of priors and Markov chain Monte Carlo settings
☒	☐	For hierarchical and complex designs, identification of the appropriate level for tests and full reporting of outcomes
☒	☐	Estimates of effect sizes (e.g. Cohen's d, Pearson's r), indicating how they were calculated

Our web collection on statistics for biologists contains articles on many of the points above.

Software and code

Policy information about availability of computer code

Data collection	No software used for collecting data.
Data analysis	Detailed description for all the softwares used for analysis have been provided in the Methods as well as Supplementary Note. The tools and software used in this study: Falcon, ALLMAP, AUGUSTUS, SNAP, BLAST v2.2.28, BLAT, Bowtie, Bowtie2, BWA-MEM v0.7.15, Celera Assembler v8.3rc2, BUSCO v2, Circos v0.69, ANNOVAR, Clustal Omega, Cufflinks, EuGene v4.2, GATK v3.8, GeneMark v4.38, HAPLOSWEEP v1.0, HiC-Pro software, ColinearScan, HighMap, HMMER v3, LACHESIS, MAKER, MCScanX, MEGA, MiRanda v3.0,TASSEL v 5.0, OrthoMCL, pbalign, Quiver tool in Genomic Consensus, PicardTools v2.17.10, Pilon, QTL-seq pipeline v1.4.4, RepeatMasker, RepeatModeler, SMRT-make, SNPhylo v20160204, PbcR v8.3rc2, TopHat, trimmomatic V0.35 and v0.36, WGCNA, and WGS v8.3.

For manuscripts utilizing custom algorithms or software that are central to the research but not yet described in published literature, software must be made available to editors/reviewers. We strongly encourage code deposition in a community repository (e.g. GitHub). See the Nature Research guidelines for submitting code & software for further information.

Data

Policy information about availability of data

All manuscripts must include a data availability statement. This statement should provide the following information, where applicable:

- Accession codes, unique identifiers, or web links for publicly available datasets
- A list of figures that have associated raw data
- A description of any restrictions on data availability

We have included a data availability statement in the MS.

Field-specific reporting

Please select the one below that is the best fit for your research. If you are not sure, read the appropriate sections before making your selection.

☒ Life sciences ☐ Behavioural & social sciences ☐ Ecological, evolutionary & environmental sciences

For a reference copy of the document with all sections, see nature.com/documents/nr-reporting-summary-flat.pdf

Life sciences study design

All studies must disclose on these points even when the disclosure is negative.

Sample size	Samples were selected to have enough representation from cultivated and wild species to derive meaningful and decisive conclusion. The genotype selected for developing reference genome belongs to cultivated peanut, A. hypogaea var. Shitouqi (zh.h0235, a well-known Chinese cultivar and breeding parent belonging to subspecies fastigiata, botanical type vulgaris and agronomic type Spanish. Such subspecies cover majority of the growing regions across world, specially Asia and Africa. Nevertheless, some related species were also sequenced for comparative genome analysis.
Data exclusions	No data was excluded from the analysis
Replication	We have performed all the experiments in replications/iterations and results are reproducible, for example, traits of pod and seed and resistance were characterize in three replication and performed in at least two years for correct QTL mapping and QTLseq.
Randomization	Not Applicable in case of genome sequencing work
Blinding	Not Applicable in case of genome sequencing work

Reporting for specific materials, systems and methods

We require information from authors about some types of materials, experimental systems and methods used in many studies. Here, indicate whether each material, system or method listed is relevant to your study. If you are not sure if a list item applies to your research, read the appropriate section before selecting a response.

Materials & experimental systems

n/a	Involved in the study	
☒	☐	Antibodies
☒	☐	Eukaryotic cell lines
☒	☐	Palaeontology
☒	☐	Animals and other organisms
☒	☐	Human research participants
☒	☐	Clinical data

Methods

n/a	Involved in the study	
☒	☐	ChIP-seq
☒	☐	Flow cytometry
☒	☐	MRI-based neuroimaging
